# Effect of monthly intermittent preventive treatment with dihydroartemisinin–piperaquine with and without azithromycin versus monthly sulfadoxine–pyrimethamine on adverse pregnancy outcomes in Africa: a double-blind randomised, partly placebo-controlled trial

**DOI:** 10.1016/S0140-6736(22)02535-1

**Published:** 2023-03-25

**Authors:** Mwayiwawo Madanitsa, Hellen C Barsosio, Daniel T R Minja, George Mtove, Reginald A Kavishe, James Dodd, Queen Saidi, Eric D Onyango, Kephas Otieno, Duolao Wang, Ulla Ashorn, Jenny Hill, Crispin Mukerebe, Samwel Gesase, Omari A Msemo, Victor Mwapasa, Kamija S Phiri, Kenneth Maleta, Nigel Klein, Pascal Magnussen, John P A Lusingu, Simon Kariuki, Jacklin F Mosha, Michael Alifrangis, Helle Hansson, Christentze Schmiegelow, Julie R Gutman, R Matthew Chico, Feiko O ter Kuile

**Affiliations:** a School of Global and Public Health, Kamuzu University of Health Sciences, Blantyre, Malawi; b Department of Clinical Sciences, Academy of Medical Sciences, Malawi University of Science and Technology, Thyolo, Malawi; c Kenya Medical Research Institute, Centre for Global Health Research, Kisumu, Kenya; d National Institute for Medical Research, Tanga Centre, Tanga, Tanzania; e Kilimanjaro Clinical Research Institute and Kilimanjaro Christian Medical University College, Moshi, Tanzania; f Department of Clinical Sciences, Liverpool School of Tropical Medicine, Liverpool, UK; g Centre for Child, Adolescent and Maternal Health Research, Faculty of Medicine and Health Technology, Tampere University, Tampere, Finland; h National Institute for Medical Research, Mwanza, Tanzania; i Great Ormond Street Institute of Child Health, University College London, London, UK; j Centre for Medical Parasitology, Department of Immunology and Microbiology, University of Copenhagen and Department of Infectious Diseases, Copenhagen University Hospital, Copenhagen, Denmark; k Malaria Branch, Division of Parasitic Diseases and Malaria, Center for Global Health, Centers for Disease Control and Prevention, Atlanta, GA, USA; l Department of Disease Control, London School of Hygiene & Tropical Medicine, London, UK

## Abstract

**Background:**

Intermittent preventive treatment in pregnancy (IPTp) with dihydroartemisinin–piperaquine is more effective than IPTp with sulfadoxine–pyrimethamine at reducing malaria infection during pregnancy in areas with high-grade resistance to sulfadoxine–pyrimethamine by *Plasmodium falciparum* in east Africa. We aimed to assess whether IPTp with dihydroartemisinin–piperaquine, alone or combined with azithromycin, can reduce adverse pregnancy outcomes compared with IPTp with sulfadoxine–pyrimethamine.

**Methods:**

We did an individually randomised, double-blind, three-arm, partly placebo-controlled trial in areas of high sulfadoxine–pyrimethamine resistance in Kenya, Malawi, and Tanzania. HIV-negative women with a viable singleton pregnancy were randomly assigned (1:1:1) by computer-generated block randomisation, stratified by site and gravidity, to receive monthly IPTp with sulfadoxine–pyrimethamine (500 mg of sulfadoxine and 25 mg of pyrimethamine for 1 day), monthly IPTp with dihydroartemisinin–piperaquine (dosed by weight; three to five tablets containing 40 mg of dihydroartemisinin and 320 mg of piperaquine once daily for 3 consecutive days) plus a single treatment course of placebo, or monthly IPTp with dihydroartemisinin–piperaquine plus a single treatment course of azithromycin (two tablets containing 500 mg once daily for 2 consecutive days). Outcome assessors in the delivery units were masked to treatment group. The composite primary endpoint was adverse pregnancy outcome, defined as fetal loss, adverse newborn baby outcomes (small for gestational age, low birthweight, or preterm), or neonatal death. The primary analysis was by modified intention to treat, consisting of all randomised participants with primary endpoint data. Women who received at least one dose of study drug were included in the safety analyses. This trial is registered with ClinicalTrials.gov, NCT03208179.

**Findings:**

From March-29, 2018, to July 5, 2019, 4680 women (mean age 25·0 years [SD 6·0]) were enrolled and randomly assigned: 1561 (33%; mean age 24·9 years [SD 6·1]) to the sulfadoxine–pyrimethamine group, 1561 (33%; mean age 25·1 years [6·1]) to the dihydroartemisinin–piperaquine group, and 1558 (33%; mean age 24·9 years [6.0]) to the dihydroartemisinin–piperaquine plus azithromycin group. Compared with 335 (23·3%) of 1435 women in the sulfadoxine–pyrimethamine group, the primary composite endpoint of adverse pregnancy outcomes was reported more frequently in the dihydroartemisinin–piperaquine group (403 [27·9%] of 1442; risk ratio 1·20, 95% CI 1·06–1·36; p=0·0040) and in the dihydroartemisinin–piperaquine plus azithromycin group (396 [27·6%] of 1433; 1·16, 1·03–1·32; p=0·017). The incidence of serious adverse events was similar in mothers (sulfadoxine–pyrimethamine group 17·7 per 100 person-years, dihydroartemisinin–piperaquine group 14·8 per 100 person-years, and dihydroartemisinin–piperaquine plus azithromycin group 16·9 per 100 person-years) and infants (sulfadoxine–pyrimethamine group 49·2 per 100 person-years, dihydroartemisinin–piperaquine group 42·4 per 100 person-years, and dihydroartemisinin–piperaquine plus azithromycin group 47·8 per 100 person-years) across treatment groups. 12 (0·2%) of 6685 sulfadoxine–pyrimethamine, 19 (0·3%) of 7014 dihydroartemisinin–piperaquine, and 23 (0·3%) of 6849 dihydroartemisinin–piperaquine plus azithromycin treatment courses were vomited within 30 min.

**Interpretation:**

Monthly IPTp with dihydroartemisinin–piperaquine did not improve pregnancy outcomes, and the addition of a single course of azithromycin did not enhance the effect of monthly IPTp with dihydroartemisinin–piperaquine. Trials that combine sulfadoxine–pyrimethamine and dihydroartemisinin–piperaquine for IPTp should be considered.

**Funding:**

European & Developing Countries Clinical Trials Partnership 2, supported by the EU, and the UK Joint-Global-Health-Trials-Scheme of the Foreign, Commonwealth and Development Office, Medical Research Council, Department of Health and Social Care, Wellcome, and the Bill-&-Melinda-Gates-Foundation.

**Research in context**

**Evidence before this study**

A 2018 meta-analysis of two trials (one from western Kenya and one from Uganda) of intermittent preventive treatment in pregnancy (IPTp) with dihydroartemisinin–piperaquine versus standard of care of IPTp with sulfadoxine–pyrimethamine concluded that in areas of high resistance to sulfadoxine–pyrimethamine, the fixed-dose combination of dihydroartemisinin–piperaquine is a promising candidate to replace sulfadoxine–pyrimethamine as IPTp. Dihydroartemisinin–piperaquine was well tolerated and associated with greater reductions in the incidence of moderate-to-severe maternal anaemia, clinical malaria, and the prevalence of maternal peripheral malaria infection at delivery, and placental malaria infections than sulfadoxine–pyrimethamine. In addition, the risk of fetal loss was lower in the IPTp in the dihydroartemisinin–piperaquine group. However, no difference was reported in adverse morbidity outcomes among livebirths (low birthweight, preterm birth, or small for gestational age). WHO recommended that more research was needed to evaluate the effect of IPTp with dihydroartemisinin–piperaquine on adverse pregnancy outcomes. Before the final design of this trial, we did an initial electronic literature search for trials comparing IPTp with sulfadoxine–pyrimethamine with IPTp with dihydroartemisinin–piperaquine using the search terms (“intermittent” OR “IPT”) AND (“sulfadoxine–pyrimethamine” OR “sulphadoxine–pyrimethamine”) AND “dihydroartemisinin–piperaquine” AND (“malaria” OR “Plasmodium”) AND (“pregnan*” OR “antenatal”) AND “trial”. We searched MEDLINE and the Malaria in Pregnancy Library, which consists of references from Web of Knowledge, Scopus, CINAHL, Bioline, the Cochrane Library databases, and WHO Global Health Library, from inception of the database to Dec 1, 2016. The Malaria in Pregnancy Library also contains grey literature and conference abstracts. No language restrictions were used. No new trials were identified. We updated our literature search on June 20, 2022, and identified two additional trials, one from Uganda (n=782) and one from southern Tanzania (n=956). In both trials, monthly IPTp with dihydroartemisinin–piperaquine was superior to sulfadoxine–pyrimethamine for reducing the risk of malaria infections during pregnancy. In the trial in Tanzania, dihydroartemisinin–piperaquine was also superior with regards to mean birthweight and low birthweight, but this was not observed in the Ugandan trial. No trials were identified that combined azithromycin with IPTp with dihydroartemisinin–piperaquine.

**Added value of this study**

None of the four previous trials were powered to assess the effect of dihydroartemisinin–piperaquine on adverse pregnancy outcomes, and the results were conflicting. Our trial in 4680 women was designed to address the recommendation by WHO for larger definitive trials to investigate the effect of monthly IPTp with dihydroartemisinin–piperaquine compared with IPTp with sulfadoxine–pyrimethamine on adverse pregnancy outcomes. We found that the standard of care of monthly IPTp with sulfadoxine–pyrimethamine was associated with fewer adverse pregnancy outcomes (primary composite endpoint) than monthly dihydroartemisinin–piperaquine, and that this difference was driven by better maternal gestational weight gain and fewer low-birthweight and small-for-gestational-age births. However, compared with monthly IPTp with sulfadoxine–pyrimethamine, monthly IPTp with dihydroartemisinin–piperaquine was associated with reductions in clinical malaria, malaria infection detected by microscopy during pregnancy, and placental malaria at delivery. To our knowledge, this is the first trial to assess the effect of IPTp with dihydroartemisinin–piperaquine and azithromycin on sexually transmitted infections and bacterial vaginosis. We showed that adding a single 2 g dose of azithromycin at the initial antenatal visit between 16 weeks and 28 weeks gestation did not enhance the effect of monthly dihydroartemisinin–piperaquine on adverse pregnancy outcomes (primary composite endpoint). IPTp with sulfadoxine–pyrimethamine was associated with a lower prevalence of *Chlamydia trachomatis* at term than IPTp with dihydroartemisinin–piperaquine plus azithromycin or dihydroartemisinin–piperaquine alone. This finding is important because azithromycin is the WHO-recommended first-line treatment for chlamydia.

**Implications of all the available evidence**

Replacing IPTp with sulfadoxine–pyrimethamine with dihydroartemisinin–piperaquine will probably result in marked and clinically relevant reductions in clinical malaria and maternal malaria infection during pregnancy in areas with high sulfadoxine–pyrimethamine resistance. However, countries that choose to do so need to be aware that replacing sulfadoxine–pyrimethamine with dihydroartemisinin–piperaquine may increase the absolute number of adverse pregnancy outcomes compared with IPTp with sulfadoxine–pyrimethamine, even in areas with very high sulfadoxine–pyrimethamine resistance. We hypothesise that sulfadoxine–pyrimethamine has potent non-malarial effects on fetal growth that outweigh any improvements in birthweight associated with better prevention from malaria by dihydroartemisinin–piperaquine. IPTp with dihydroartemisinin–piperaquine combined with sulfadoxine–pyrimethamine warrants investigation to evaluate the potential benefits of combining the non-malarial effects of sulfadoxine–pyrimethamine on fetal growth with the superior antimalarial effects of dihydroartemisinin–piperaquine. Moreover, additional studies of the mechanisms by which sulfadoxine–pyrimethamine promotes maternal gestational weight gain and fetal growth, independent of its antimalarial effects, are also warranted.

## Introduction

The adverse consequences of *Plasmodium falciparum* malaria infection during pregnancy on maternal and pregnancy outcomes are well recognised.^1^ WHO recommends intermittent preventive treatment in pregnancy (IPTp) with sulfadoxine–pyrimethamine for the control of malaria in pregnant women in malaria-endemic countries,^2^ but the efficacy of IPTp with sulfadoxine–pyrimethamine is being reduced by high-grade sulfadoxine–pyrimethamine resistance.^3^ Over the past 15 years, several trials have evaluated potential alternatives to sulfadoxine–pyrimethamine for IPTp; amodiaquine, sulfadoxine-pyrimethamine-amodiaquine mefloquine, and chloroquine alone, and chloroquine–azithromycin were deemed unsuitable because of their poor tolerability as IPTp.^4^ Of the long-acting antimalarials, dihydroartemisinin–piperaquine is the only candidate with the potential to replace sulfadoxine–pyrimethamine for IPTp.^4^, ^5^, ^6^ A meta-analysis^4^ of the first two trials^5^, ^6^ showed that IPTp with dihydroartemisinin–piperaquine was well tolerated and associated with 42% (95% CI 4–65) fewer episodes of moderate-to-severe maternal anaemia, 75% (60–84) fewer episodes of clinical malaria, and 65% (30–83) fewer placental malaria infections than IPTp with sulfadoxine–pyrimethamine. In addition, the risk of fetal loss was 61% (95% CI 13–83) lower in women who received IPTp with dihydroartemisinin–piperaquine .^4^

However, the effect of IPTp with dihydroartemisinin–piperaquine on adverse newborn baby outcomes (low birthweight, preterm birth, or small for gestational age) varied from 18% (95% CI –36 to 50) in Uganda to –9% (–66 to 29) in Kenya (pooled 2%; 95% CI –36 to 29).^4^ These data were presented to WHO in July, 2015, and WHO recommended that more research was needed to evaluate the effect of IPTp with dihydroartemisinin–piperaquine on adverse pregnancy outcomes.^7^ Since then, two further trials—one in Uganda^8^ and one in southern Tanzania^9^—have confirmed the superior effect of monthly IPTp with dihydroartemisinin–piperaquine compared with sulfadoxine–pyrimethamine on malarial infections during pregnancy. A superior effect of dihydroartemisinin–piperaquine on low birthweight was reported in the trial in Tanzania (51%; 95% CI 22 to 73),^9^ but not reported in the trial in Uganda (19%; 95% CI –36 to 52).^8^

Sexually transmitted infections (STIs) and other reproductive tract infections (RTIs) in pregnancy are also prevalent causes of poor birth outcomes in sub-Saharan Africa, especially in eastern and southern Africa.^10^ Similar to malaria infections, STIs and other RTIs remain mostly asymptomatic and, therefore, often undetected and untreated. Azithromycin, a broad-spectrum antibiotic, has activity against curable STIs and other RTIs associated with adverse pregnancy outcomes, including syphilis, gonorrhoea, chlamydia and, potentially, trichomoniasis.^11^ Combining dihydroartemisinin–piperaquine with azithromycin might improve birth outcomes, potentially paving the way for integrated strategies for controlling malaria and curable STI and other RTI coinfections in pregnancy.

We aimed to assess whether IPTp with dihydroartemisinin–piperaquine, alone or combined with azithromycin, could reduce adverse pregnancy outcomes compared with IPTp with sulfadoxine–pyrimethamine.

## Methods

### Study design and participants

This three-arm, individually randomised, double-blind, partly placebo-controlled, trial was done in 12 antenatal clinics in southern Malawi (n=5), northeastern Tanzania (n=3), and western Kenya (n=4) in areas with high-grade sulfadoxine–pyrimethamine resistance and perennial malaria transmission. Women of any age with a viable singleton pregnancy between 16 weeks and 28 weeks gestation confirmed by ultrasound were eligible for inclusion if they were residents of the study area and were willing to adhere to the follow-up schedule and give birth in a study clinic. Women with multiple pregnancies, a known heart ailment, a history of receiving IPTp with sulfadoxine–pyrimethamine during the current pregnancy, a known allergy or contraindication to any of the study drugs, or women living with HIV were excluded. All study participants provided written informed consent. The study was approved by the ethics committees of the Kenya Medical Research Institute, Nairobi, Kenya; the College of Medicine, Blantyre, Malawi; the Medical Research Coordinating Committee, Dar Es Salaam, Tanzania; and the Liverpool School of Tropical Medicine (LSTM), Liverpool, UK.

### Randomisation and masking

The comparison between IPTp with sulfadoxine–pyrimethamine and IPTp with dihydroartemisinin–piperaquine was open label. The use of azithromycin was placebo controlled to ensure masking between the two arms containing IPTp with dihydroartemisinin–piperaquine. Women were randomly assigned (1:1:1) to receive monthly IPTp with sulfadoxine–pyrimethamine (sulfadoxine–pyrimethamine group), monthly IPTp with dihydroartemisinin–piperaquine plus a single treatment course of placebo at enrolment (dihydroartemisinin–piperaquine group), or monthly IPTp with dihydroartemisinin–piperaquine combined with a single dose of azithromycin at enrolment (dihydroartemisinin–piperaquine plus azithromycin group). Randomisation sequences were computer generated centrally at LSTM by the study statistician (DW), using permuted block randomisation (block size of three), stratified by site and gravidity (first or second *vs* more than two previous pregnancies). Before study onset, a set of sequentially numbered, opaque sealed envelopes containing the allocation group was prepared and stored in two boxes per site, one for each gravidity stratum. A second statistician at LSTM and a lead pharmacist in Kenya who were not otherwise involved in the study were responsible for treatment allocation and the preparation of study drugs. In each clinic, study staff were responsible for participant enrolment. The allocation of eligible participants occurred in the order of their study identification number by drawing the next sequentially numbered sealed envelope. Outcome assessors in the delivery units, laboratory staff, data managers, and study statisticians were masked to treatment group.

In addition, a subgroup of a third of all participants in each group and study site was selected randomly for additional assessments. These included serial ultrasound scans to assess fetal growth, and extra samples taken for the assessment of STIs and other RTIs in the third trimester. This was based on computer-generated lists prepared centrally by the statistician in Liverpool (DW) before the start of the study. The same statistician also prepared another computer-generated list to select 10% of participants in each group and study site for home visits to assess tolerance and adherence to study drugs administered at home. The information on whether the participant was eligible for these additional assessments and home visits was included in the same sealed envelopes as the study allocation.

### Procedures

At enrolment, demographic, socioeconomic, and educational information was collected from the participant on standardised case report forms. Participants had their medical and obstetric history recorded, a venous blood and urine sample collected, and they underwent a routine medical examination and an ultrasound scan to assess their gestational age. The randomly selected subgroup of a third of participants had vaginal swabs taken to assess for the presence of curable STIs and bacterial vaginosis. All participants received a long-lasting insecticide-treated net as part of routine antenatal care.

Each sulfadoxine–pyrimethamine course consisted of three tablets containing 500 mg of sulfadoxine and 25 mg of pyrimethamine (unbranded generic sulfadoxine–pyrimethamine, Medopharm, Chennai, India; quality controlled by Durbin, Hayes, UK) given as a single oral dose for 1 day (appendix p 2). Each dihydroartemisinin–piperaquine course was dosed according to the bodyweight of each participant and consisted of three to five tablets containing 40 mg of dihydroartemisinin and 320 mg of piperaquine (Alfasigma, Bologna, Italy), given orally once a day for 3 consecutive days. Each dose of azithromycin consisted of two tablets containing 500 mg (Universal Corporation, Nairobi, Kenya) given orally once daily for 2 consecutive days (cumulative dose of 2 g) at the same time as the first and second daily dose of dihydroartemisinin–piperaquine at enrolment. The placebo tablets were also provided by Universal Corporation and had the same appearance as active azithromycin (appendix p 2). The first daily dose was administered in the study clinic under the direct supervision of the study staff, combined with a slice of dry bread or a biscuit. The daily doses on the second and third days were self-administered at home at approximately the same time of the day and in a similar manner as the first dose taken under observation in the clinic. Tolerance and adherance to study drugs administered at home were assessed during unannounced home visits in a randomly selected subset of 10% of participants in each group and during telephone calls to all participants on days 2 and 3 of each course.

In Kenya and Tanzania, women were screened for malaria at enrolment using malaria rapid diagnostic tests (RDTs) as per national guidelines. All women who tested negative in all groups received their first course of IPTp at enrolment; women who tested positive in all groups in Kenya and those who tested positive in the IPTp with sulfadoxine–pyrimethamine group in Tanzania received a standard 3-day treatment course with artemether–lumefantrine. The first dose of IPTp was delayed until the next scheduled visit 4 weeks later. In Tanzania, women who tested positive for malaria in both the groups that included dihydroartemisinin–piperaquine received their first course of IPTp with dihydroartemisinin–piperaquine or IPTp with dihydroartemisinin–piperaquine plus azithromycin at enrolment instead of artemether-lumefantrine (appendix p 2).

Participants attended study clinics every 4 weeks, during which a clinical history of recent illness and drug use was taken and a clinical and obstetric examination was done. A blood sample was taken for malaria microscopy, quantitative PCR (qPCR), and targeted next-generation sequencing for molecular markers of sulfadoxine–pyrimethamine resistance for later examination, not to inform patient care. Haemoglobin and routine urinalysis were assessed during the last scheduled visit in the third trimester. Women were encouraged to make unscheduled visits if they felt ill or were concerned about their pregnancy. Women with documented fever or a history of fever during or between scheduled visits were tested for malaria by RDT. Women who tested positive for malaria in all groups received a 3-day course of artemether–lumefantrine. If this treatment overlapped with a scheduled IPTp visit, the IPTp course was delayed for 4 weeks, until the next scheduled visit. In the random sample of a third of women from each site, fetal weight was estimated by ultrasound scan at enrolment if the gestational age was at least 22 weeks and converted into Z scores using the INTERGROWTH-21 reference (appendix p 4). Two additional ultrasound scans were done to estimate fetal weight at scheduled visits at around 25–28 and 32–35 weeks gestation. In these women, repeat vaginal swabs and microscopy slides of genital secretions were taken before dosing at enrolment and again at the 32–36-week visit to assess the effect of exposure to IPTp on STIs and bacterial vaginosis (all countries).

In all participants, a maternal venous blood sample for the same malaria metrics was taken at delivery (during labour where possible), combined with placental and cord-blood samples for placental histology, malaria RDT, microscopy, and qPCR. qPCR samples at enrolment and delivery were analysed for all participants. The qPCR samples taken between enrolment and delivery were analysed in the third of all participants selected for serial ultrasound and third trimester assessment of STIs and bacterial vaginosis described earlier. Newborn babies were weighed as soon as study staff were notified of birth (appendix p 3). In addition, congenital abnormalities and jaundice were assessed at delivery, day 7, and at the final visit at 6–8 weeks, coinciding with the infants' childhood vaccination visit. Between scheduled visits, infants were followed up passively, when parents or guardians came to the study clinic because they were concerned about the health of the baby.

In a subgroup of 189 women from Malawi and Kenya who were in the two IPTp groups containing dihydroartemisinin–piperaquine, three sets of electrocardiograms were done to assess the heart-rate corrected QT intervals (QTc) before the first daily dose of dihydroartemisinin–piperaquine and 4 h after the third daily dose at enrolment, and twice more during pregnancy (approximately 1–2 and 3–4 months later; appendix p 3). The 189 women were selected through convenient sampling based on travel time to the facility and their availability to attend the extra clinic visits. Adverse events and serious adverse events were assessed up to 6 weeks postpartum and graded using the Common Terminology Criteria for Adverse Events (version 5.0) at every scheduled monthly clinic visit and unscheduled visit. Events were coded using Medical Dictionary for Regulatory Activities (MedDRA) coding. Tolerability was assessed by comparing the rates of vomiting within 30 min of drug intake in the clinic and other adverse events at each scheduled monthly visit (all women) and during random home visits in 10% of participants (appendix p 6).

### Outcomes

The primary endpoint was adverse pregnancy outcome, defined as a composite of either fetal loss (miscarriage or stillbirth), adverse newborn baby outcomes (low birthweight, preterm, or small for gestational age), or neonatal death (appendix p-4). Key secondary efficacy endpoints included the individual components of the composite primary endpoint, maternal haemoglobin concentrations and anaemia (haemoglobin <11 g/dL or <9 g/dL), clinical malaria, plasmodium infection during pregnancy and at delivery, placental inflammation or chorioamnionitis, maternal gestational weight gain, maternal mid-upper-arm circumference, mean birthweight, mean gestational age, and mean Z score for fetal growth and birthweight by gestational age, cord haemoglobin concentrations and fetal anaemia (haemoglobin <12·5 g/dL), and congenital malaria infection. For a full list of all endpoints see the appendix (pp 4–6).

### Statistical analysis

This study was designed to achieve 80% power to detect at least a 20% reduction in the composite primary endpoint of adverse pregnancy outcomes from 21·3% with IPTp with sulfadoxine–pyrimethamine^5^, ^12^, ^13^ to 17·0% in the IPTp with dihydroartemisinin–piperaquine group or IPTp with dihydroartemisinin–piperaquine plus azithromycin group (risk ratio [RR] 0·80; two-sided α=0·05), which required 4680 participants (1560 per group), allowing for 13·7% loss to follow-up. Statistical analyses were done using Stata (version 17). For the primary and dichotomous secondary endpoints, we used log-binomial regression to obtain RRs and corresponding 95% CIs, and modified Poisson regression in case of non-convergence (appendix pp 7–8). We used linear regression for continuous variables, and results were expressed as mean difference and 95% CIs. The mean differences for estimated fetal weight Z scores were obtained by linear mixed models for repeated measures. We used Poisson regression with a log-link function and follow-up time as an offset for count variables to obtain incidence rate ratios and 95% CIs. The unadjusted (crude) analysis was the primary analysis and included the stratification factors study site and gravidae group in all models. Secondary, covariate-adjusted analyses were done using the following other prespecified baseline covariates in addition to gravidity and site: malaria status (positive *vs* negative) at enrolment; gestational age (continuous) at enrolment; socioeconomic status (calculated using principal component analysis; continuous); malaria transmission season (based on average rainfall in the last 6 months of pregnancy; continuous); malaria transmission intensity by study site (based on the prevalence of malaria at enrolment); and the degree of sulfadoxine–pyrimethamine resistance by study site (very high *vs* high based on the prevalence of the Ala581Gly substitutions in the *dhps* gene >40% *vs* ≤40%; appendix p 7). The unadjusted linear mixed models contained visit, study arm, the interaction between visit and study arm, and the stratification factors site and gravidity as fixed effect and participant as random effects to account for the clustering within subject. The adjusted linear mixed models also included the six additional prespecified covariates as fixed effect and participant as random effects. Missing covariates were imputed using simple imputation. These same covariates were also included in subgroup analyses. We used a two-sided p value less than 0·05 to define statistical significance. p values and the widths of the 95% CIs for the primary and secondary endpoints have not been adjusted for multiplicity, so the values should not be used to infer definitive treatment effects. The modified intention-to-treat (ITT) population (ie, all randomised participants who contributed to the endpoint) was used for primary analyses. We did a sensitivity analysis using non-responder imputation^14^ to assess the effect of attrition bias (appendix p 8). Per-protocol analyses were done for the composite primary endpoint and its individual components. The per-protocol population included participants who attended every scheduled visit, took all scheduled IPTp courses, did not use prohibited medication, and contributed to the endpoint. For the safety analyses, women were included if they received at least one dose of study drug. All analyses were prespecified (unless otherwise indicated as post hoc) in a statistical analysis plan approved by the data and safety monitoring board. Post-hoc analyses in women were any malaria at delivery (a composite of malaria infection detected by any diagnostic test in either maternal or placental blood); gestational weight gain; and a composite of placental inflammation or chorioamnionitis. Post-hoc analyses in infants were a composite of fetal and neonatal death, early neonatal death, perinatal death, neonatal wasting, and a composite of neonatal wasting or stunting. This trial is registered with ClinicalTrials.gov, NCT03208179.

### Role of the funding source

The funders of the study had no role in study design, data collection, data analysis, data interpretation, or writing of the report.

## Results

**Figure 1:**
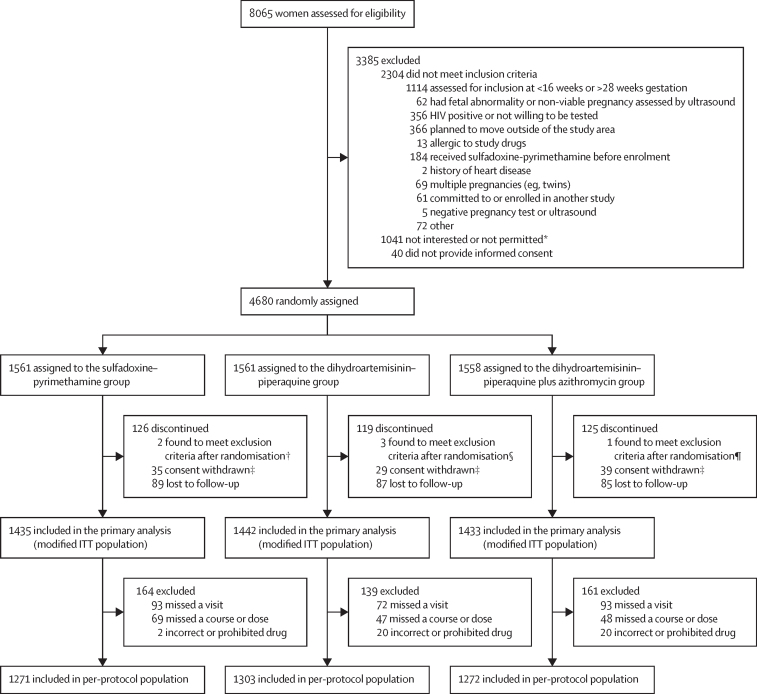
Trial profile ITT=intention to treat. *Full eligibility criteria could not be assessed in these women, who either expressed hesitation to join the study or whose partner or spouse or another family member discouraged them from joining the research study. †Non-viable pregnancy detected by ultrasound at screening, but erroneously issued a study number (n=1); allergic to sulfadoxine–pyrimethamine based on medical history after randomisation (n=1). ‡Participant withdrew consent or was withdrawn for safeguarding reasons (ie, partner or spouse requested participant to withdraw consent). §HIV positive (n=1); twin pregnancy detected by ultrasound at screening, but erroneously issued a study number (n=1); not pregnant, but erroneously enrolled because of a mix-up with another (non-participant) with identical first and last name (n=1). ¶Non-viable pregnancy detected by ultrasound at screening, but erroneously issued a study number (n=1).

From March 29, 2018, to July 5, 2019, 8065 women were screened for inclusion. Recruitment was stopped when 4680 participants (mean age 25·0 years [SD 6·0]; range 14–48 years) had been randomly assigned: 1561 (33%; mean age 24·9 years [SD 6·1]) assigned to the sulfadoxine–pyrimethamine group, 1561 (33%; mean age 25·1 years [6·1]) assigned to the dihydroartemisinin–piperaquine group, and 1558 (33%; mean age 24·9 years [6·0]) assigned to receive dihydroartemisinin–piperaquine plus azithromycin (figure 1). 1490 women were from Kenya, 1404 from Malawi, and 1786 from Tanzania. Baseline characteristics are reported in table 1. At baseline, 1057 (23%) of 4680 women were infected with malaria parasites (any diagnostic test): 343 (22%) of 1561 in the sulfadoxine–pyrimethamine group, 339 (22%) of 1561 in the dihydroartemisinin–piperaquine group, and 375 (24%) of 1558 in the dihydroartemisinin–piperaquine plus azithromycin group; of those infected, 407 (27%) of 1490 were from Kenya, 342 (24%) of 1404 from Malawi, and 308 (17%) of 1786 from Tanzania. 27 (21%) of the 127 parasite samples tested expressed the sextuple *dhfr*/*dhps* haplotype containing the *dhps* Ala581Gly mutation: 20 (40%) of 50 in Tanzania, three (8%) of 40 in Malawi, and four (11%) of 37 in Kenya (appendix p 10). 778 (16·9%) of 4597 women had an STI or other RTI at enrolment and this was similar across all three groups (table 1; appendix p 12).

**Table 1:** Demographics and baseline characteristics

		**Sulfadoxine–pyrimethamine group (n=1561)**	**Dihydroartemisinin–piperaquine group (n=1561)**	**Dihydroartemisinin–piperaquine plus azithromycin group (n=1558)**
**Maternal characteristics**				
Maternal age (years)		24·9 (6·1)	25·1 (6·1)	24·9 (6·0)
Residence				
	Rural	1154/1561 (73·9%)	1134/1560 (72·7%)	1139/1557 (73·2%)
	Semi-urban or urban	407/1561 (26·1%)	426/1560 (27·3%)	418/1557 (26·8%)
Marital status				
	Single*	208/1561 (13·3%)	184/1560 (11·8%)	180/1557 (11·6%)
	Married or cohabiting	1353/1561 (86·7%)	1376/1560 (88·2%)	1377/1557 (88·4%)
Used bednet last night				
	Yes	1204/1561 (77·1%)	1250/1560 (80·1%)	1206/1557 (77·5%)
	No	357/1561 (22·9%)	310/1560 (19·9%)	351/1557 (22·5%)
Attended school				
	Yes	1478/1560 (94·7%)	1471/1559 (94·4%)	1461/1553 (94·1%)
	No	82/1560 (5·3%)	88/1559 (5·6%)	92/1553 (5·9%)
Schooling level completed				
	None	135/1560 (8·7%)	140/1559 (9·0%)	137/1553 (8·8%)
	Primary school	890/1560 (57·1%)	852/1559 (54·7%)	870/1553 (56·0%)
	Secondary school	424/1560 (27·2%)	472/1559 (30·3%)	463/1553 (29·8%)
	Higher	111/1560 (7·1%)	95/1559 (6·1%)	83/1553 (5·3%)
SES index score (terciles)				
	Low	527/1561 (33·8%)	503/1561 (32·2%)	530/1558 (34·0%)
	Medium	516/1561 (33·1%)	536/1561 (34·3%)	508/1558 (32·6%)
	High	518/1561 (33·2%)	522/1561 (33·4%)	520/1558 (33·4%)
Pregnancy number (gravidity)				
	First	493/1558 (31·6%)	473/1557 (30·4%)	435/1553 (28·0%)
	Second	373/1558 (23·9%)	393/1557 (25·2%)	429/1553 (27·6%)
	Third or higher	692/1558 (44·4%)	691/1557 (44·4%)	689/1553 (44·4%)
Weight (kg)		61 (11·5)	61 (11·1)	61 (11·1)
Height (cm)		159 (7·1)	158 (7·0)	159 (7·4)
MUAC (cm)		27·0 (3·6)	26·9 (3·5)	26·9 (3·5)
**Infant characteristics**				
Gestational age (days)		146 (24·3)	146 (23·8)	147 (24·5)
Estimated fetal weight by ultrasound (Z score)		−0·65 (1·16)	−0·45 (1·24)	−0·62 (1·03)
**Maternal laboratory findings**				
Haemoglobin (g/dL)		11·0 (1·5)	11·0 (1·5)	11·0 (1·4)
Malaria infection				
	mRDT	192/1078 (17·8%)	178/1074 (16·6%)	197/1080 (18·2%)
	Microscopy	151/1554 (9·7%)	150/1553 (9·7%)	184/1552 (11·9%)
	PCR	196/1410 (13·9%)	204/1404 (14·5%)	218/1410 (15·5%)
	Any†	343/1561 (22·0%)	339/1561 (21·7%)	375/1558 (24·1%)
**STIs or other RTIs**				
Any STI or other RTI		272/1535 (17·7%)	241/1531 (15·7%)	265/1531 (17·3%)
Any STI		183/1531 (12·0%)	158/1526 (10·4%)	168/1527 (11·0%)
	Chlamydia trachomatis	62/453 (13·7%)	59/462 (12·8%)	72/456 (15·8%)
	Neisseria gonorrhoea	10/453 (2·2%)	16/462 (3·5%)	11/456 (2·4%)
	Treponema pallidum	51/1524 (3·3%)	49/1524 (3·2%)	45/1524 (3·0%)
	Trichomonas vaginalis	73/453 (16·1%)	63/462 (13·6%)	64/456 (14·0%)
Bacterial vaginosis		124/460 (27·0%)	134/467 (28·7%)	133/461 (28·9%)

Data are mean (SD) or n/N (%), unless otherwise specified. Some percentages do not add up to 100 because of rounding. SES=socioeconomic status. MUAC=mid-upper arm circumference. mRDT=malaria rapid diagnostic test. STI=sexually transmitted infection. RTI=reproductive tract infection.

* Divorced, separated, widowed, or not cohabiting.

† Any malaria infection detected by mRDT, microscopy, or PCR*.*

The median follow-up time until delivery was 4·3 months (IQR 3·5–5·0), and the median number of IPTp courses was five (IQR four to six; appendix p 14). 19 349 (91·2%) of 21 215 scheduled monthly antenatal follow-up visits were attended (sulfadoxine–pyrimethamine 6420 [90·9%] of 7066 visits, dihydroartemisinin–piperaquine 6528 [92·0%] of 7096 visits, dihydroartemisinin–piperaquine plus azithromycin 6401 [90·8%] of 7053 visits; appendix p 14). For 802 (98·3%) of 816 doses of dihydroartemisinin–piperaquine and 166 (97·6%) of 170 doses of placebo or azithromycin, the correct number of tablets was adhered to in a subgroup of women visited at home during random spot checks, with no differences between the two dihydroartemisinin–piperaquine groups (appendix p 15).

**Figure 2:**
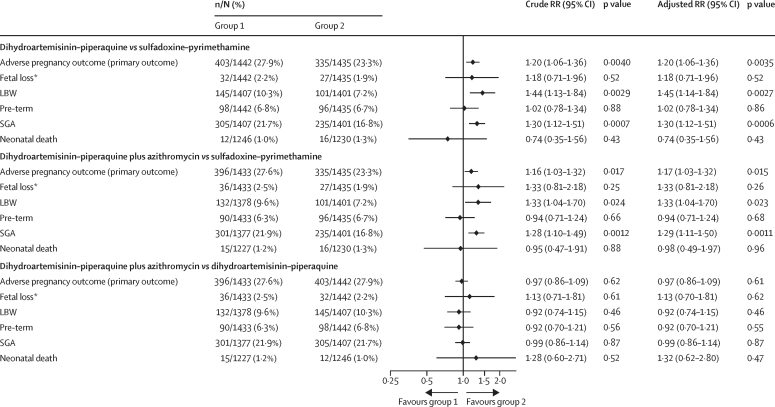
Composite primary endpount and its components (modified intention-to-treat population) LBW=low birthweight. RR=risk ratio. SGA=small for gestational age. *Fetal loss included miscarriage and stillbirth.

Of the 4680 participants randomly assigned, 4310 (92·1%) contributed to the primary endpoint analysis (figure 1). The proportion of participants with missing primary endpoint data was equally distributed across study groups (appendix p 15). Compared with the 335 (23·3%) of 1435 women in the sulfadoxine–pyrimethamine group who had adverse pregnancy outcomes (composite primary endpoint), more adverse pregnancy outcomes were reported in the dihydroartemisinin–piperaquine (403 [27·9%] of 1442; RR 1·20, 95% CI 1·06–1·36; p=0·0040) and in the dihydroartemisinin–piperaquine plus azithromycin (396 [27·6%] of 1433; 1·16, 1·03–1·32; p=0·017; figure 2) groups. The primary endpoint did not differ significantly between the dihydroartemisinin–piperaquine plus azithromycin and dihydroartemisinin–piperaquine groups (RR 0·97, 95% CI 0·86–1·09; p=0·62; figure 2). Results were similar for the prespecified covariate-adjusted analyses (figure 2), the per-protocol population analysis (appendix p 24), and the sensitivity analysis using non-responder imputation (appendix p 25).

Prespecified subgroup analyses of the comparison between the dihydroartemisinin–piperaquine and sulfadoxine–pyrimethamine groups showed evidence of a dose response, with the greatest difference in favour of sulfadoxine–pyrimethamine in women who had received six or more IPTp courses (RR 1·38, 95% CI 1·02–1·88) and no statistical difference in women who had received three or fewer courses (RR 1·03, 0·82–1·31; p_interaction_<0·0001; appendix p 26). This dose response was also observed in the comparison between dihydroartemisinin–piperaquine plus azithromycin and sulfadoxine–pyrimethamine, although the difference between subgroups was not statistically significant (p_interaction_<0·29; appendix p 27). The effect in favour of sulfadoxine–pyrimethamine was independent of the level of sulfadoxine–pyrimethamine resistance in the study area and occurred in areas with very high (Tanzania; RR 1·25, 95% CI 1·05–1·49) and high sulfadoxine–pyrimethamine parasite resistance (Kenya and Malawi; RR 1·16, 0·97–1·38; p_interaction_=0·52; appendix p 26). However, the effect in favour of sulfadoxine–pyrimethamine was not apparent in areas with very high resistance in the comparison between dihydroartemisinin–piperaquine plus azithromycin and sulfadoxine–pyrimethamine (Tanzania RR 1·01, 95% CI 0·84–1·22), only in areas with high resistance (Kenya and Malawi 1·30, 95% CI 1·10–1·54; p_interaction_=0·064; appendix p 27). Subgroup analyses of the comparison between dihydroartemisinin–piperaquine and sulfadoxine–pyrimethamine suggested that the effect in favour of sulfadoxine–pyrimethamine occurred in paucigravidae (RR 1·16, 95% CI 1·00–1·36) and multigravidae (1·27, 1·04–1·56; p_interaction_ 0·61; appendix p 26). For the comparison between dihydroartemisinin–piperaquine plus azithromycin and sulfadoxine–pyrimethamine, this effect in favour of sulfadoxine–pyrimethamine was only evident in multigravidae (RR 1·38, 95% CI 1·13–1·66), but not in paucigravidae (1·05, 0·89–1·23; p_interaction_=0·036; appendix p 27).

The prespecified secondary outcome analysis of the composite primary endpoint components showed that the lower risk of adverse pregnancy outcomes with sulfadoxine–pyrimethamine mainly reflected a difference in low birthweight and small for gestational age (figure 2). Preterm birth did not differ significantly between groups. Similarly, mean birthweight and Z scores for birthweight for gestational age were significantly higher in the sulfadoxine–pyrimethamine group than in the other two groups, yet mean gestational age did not differ significantly between groups (table 2). Serial assessment of fetal weight was available for 1440 (30·8%) of 4680 women: 479 in the sulfadoxine–pyrimethamine group, 482 in the dihydroartemisinin–piperaquine group, and 479 in the dihydroartemisinin–piperaquine plus azithromycin group. Z scores for estimated fetal weight obtained by ultrasound were significantly higher in the sulfadoxine–pyrimethamine group than in the other two groups (table 2). Risk of neonatal wasting was also lower in the sulfadoxine–pyrimethamine group than in the other two groups, but risk of neonatal stunting was similar across groups (appendix p 29). Perinatal, early neonatal, or neonatal death did not differ significantly between groups (appendix p 29).

**Table 2:** Secondary efficacy endpoints (continuous) in the modified ITT population

	**Mean (SD)**	**Mean difference (95% CI); p value**	
		Unadjusted	Adjusted
**Birthweight, g**			
SP (n=1401)	3121 (500)	..	..
DP (n=1407)	3033 (507)	..	..
DPAZ (n=1378)	3042 (480)	..	..
DP *vs* SP	..	−89 (−125 to −53); p<0·0001	−90 (−126 to −54); p<0·0001
DPAZ *vs* SP	..	−78 (−114 to −42); p<0·0001	−78 (−114 to −42); p<0·0001
DPAZ *vs* DP	..	11 (−25 to 47); p=0·55	12 (−24 to 48); p=0·52
**Birthweight for gestational age, Z score**			
SP (n=1371)	−0·32 (1·02)	..	..
DP (n=1388)	−0·50 (1·05)	..	..
DPAZ (n=1352)	−0·50 (1·02)	..	..
DP *vs* SP	..	−0·18 (−0·26 to −0·11); p<0·0001	−0·18 (−0·26 to −0·11); p<0·0001
DPAZ *vs* SP	..	−0·18 (−0·25 to −0·10); p<0·0001	−0·18 (−0·25 to −0·10); p<0·0001
DPAZ *vs* DP	..	0·00 (−0·07 to 0·08); p=0·95	0·01 (−0·07 to 0·08); p=0·88
**Gestational age at birth, weeks**			
SP (n=1395)	39·5 (2·0)	..	..
DP (n=1417)	39·4 (2·1)	..	..
DPAZ (n=1395)	39·5 (2·0)	..	..
DP *vs* SP	..	−0·1 (−0·3 to 0·0); p=0·18	−0·1 (−0·3 to 0·0); p=0·17
DPAZ *vs* SP	..	−0·1 (−0·2 to 0·1); p=0·49	−0·1 (−0·2 to 0·1); p=0·48
DPAZ *vs* DP	..	0·1 (−0·1 to 0·2); p=0·51	0·1 (−0·1 to 0·2); p=0·51
**Neonatal length, cm**			
SP (n=1327)	48·6 (3·2)	..	..
DP (n=1309)	48·4 (3·1)	..	..
DPAZ (n=1270)	48·4 (3·2)	..	..
DP *vs* SP	..	−0·2 (−0·4 to 0·0); p=0·11	−0·2 (−0·4 to 0·0); p=0·094
DPAZ *vs* SP	..	−0·2 (−0·4 to 0·0); p=0·062	−0·2 (−0·4 to 0·0); p=0·054
DPAZ *vs* DP	..	−0·0 (−0·3 to 0·2); p=0·76	−0·0 (−0·2 to 0·2); p=0·79
**Maternal MUAC, cm**			
SP (n=1345)	26·6 (3·3)	..	..
DP (n=1339)	26·3 (3·1)	..	..
DPAZ (n=1299)	26·3 (3·2)	..	..
DP *vs* SP	..	−0·3 (−0·6 to −0·1); p=0·0042	−0·4 (−0·6 to −0·1); p=0·0020
DPAZ *vs* SP	..	−0·3 (−0·5 to −0·1); p=0·016	−0·3 (−0·5 to −0·0); p=0·018
DPAZ *vs* DP	..	0·0 (−0·2 to 0·3); p=0·68	0·1 (−0·1 to 0·3); p=0·48
Estimated fetal weight by US 32–35 weeks, Z-score*			
SP (n=396)	0·19 (0·84)	..	..
DP (n=407)	−0·01 (0·92)	..	..
DPAZ (n=407)	0·05 (0·95)	..	..
DP *vs* SP	..	−0·16 (−0·27 to −0·05); p=0·0037	−0·16 (−0·26 to −0·05); p=0·0055
DPAZ *vs* SP	..	−0·18 (−0·29 to −0·07); p=0·0012	−0·17 (−0·28 to −0·06); p=0·0022
DPAZ *vs* DP	..	−0·02 (−0·13 to 0·09); p=0·74	−0·02 (−0·13 to 0·09); p=0·77
**Gestational weight gain per week, kg**†			
SP (n=1443)	0·31 (0·20)	..	..
DP (n=1454)	0·27 (0·21)	..	..
DPAZ (n=1434)	0·27 (0·20)	..	..
DP *vs* SP	..	−0·04 (−0·06 to −0·03); p<0·0001	−0·04 (−0·06 to −0·03); p<0·0001
DPAZ *vs* SP	..	−0·04 (−0·05 to −0·02); p<0·0001	−0·04 (−0·05 to −0·02); p<0·0001
DPAZ *vs* DP	..	0·00 (−0·01 to 0·02); p=0·67	0·00 (−0·01 to 0·02);p=0·52
**Maternal Hb third trimester, g/dL**			
SP (n=1375)	10·9 (1·4)	..	..
DP (n=1390)	10·9 (1·3)	..	..
DPAZ (n=1350)	10·9 (1·3)	..	..
DP *vs* SP	..	−0·0 (−0·1 to 0·1); p=0·87	−0·0 (−0·1 to 0·1); p=0·88
DPAZ *vs* SP	..	−0·0 (−0·1 to 0·1); p=0·62	−0·0 (−0·1 to 0·1); p=0·67
DPAZ *vs* DP	..	−0·0 (−0·1 to 0·1); p=0·74	−0·0 (−0·1 to 0·1); p=0·78
**Maternal Hb delivery, g/dL**			
SP (n=431)	11·2 (1·8)	..	..
DP (n=415)	11·3 (1·6)	..	..
DPAZ (n=410)	11·3 (1·8)	..	..
DP *vs* SP	..	0·1 (−0·2 to 0·3); p=0·55	0·1 (−0·2 to 0·3); p=0·60
DPAZ *vs* SP	..	0·1 (−0·1 to 0·4); p=0·30	0·1 (−0·1 to 0·4); p=0·30
DPAZ *vs* DP	..	0·1 (−0·2 to 0·3); p=0·66	0·1 (−0·2 to 0·3); p=0·62
**Cord blood Hb, g/dL**			
SP (n=1182)	14·4 (2·0)	..	..
DP (n=1185)	14·3 (2·1)	..	..
DPAZ (n=1130)	14·5 (2·0)	..	..
DP *vs* SP	..	−0·1 (−0·2 to 0·1); p=0·41	−0·1 (−0·2 to 0·1); p=0·36
DPAZ *vs* SP	..	0·1 (−0·0 to 0·3); p=0·12	0·1 (−0·0 to 0·3); p=0·10
DPAZ *vs* DP	..	0·2 (0·0 to 0·4); p=0·017	0·2 (0·0 to 0·4); p=0·012

The crude models include the stratification factors site and gravidity (paucigravidae and multigravidae) as covariates. ITT=intention to treat. SP=sulfadoxine–pyrimethamine. DP= dihydroartemisinin–piperaquine. DPAZ=dihydroartemisinin–piperaquine plus azithromycin. MUAC=mid-upper arm circumference. US=ultrasound scan. Hb=haemoglobin.

* If no ultrasound scan was available during that time window, the closest ultrasound scan taken in the third trimester was used. The mean Z-scores for estimated fetal weight represent the scores measured around 32–35 weeks gestation. The mean difference represents the overall difference obtained by linear mixed models for repeated measures that also include the measures taken around 25–28 weeks gestation and included 479, 482, and 479 participants in the SP, DP, and DPAZ groups, respectively.

† Post-hoc analysis.

**Figure 3:**
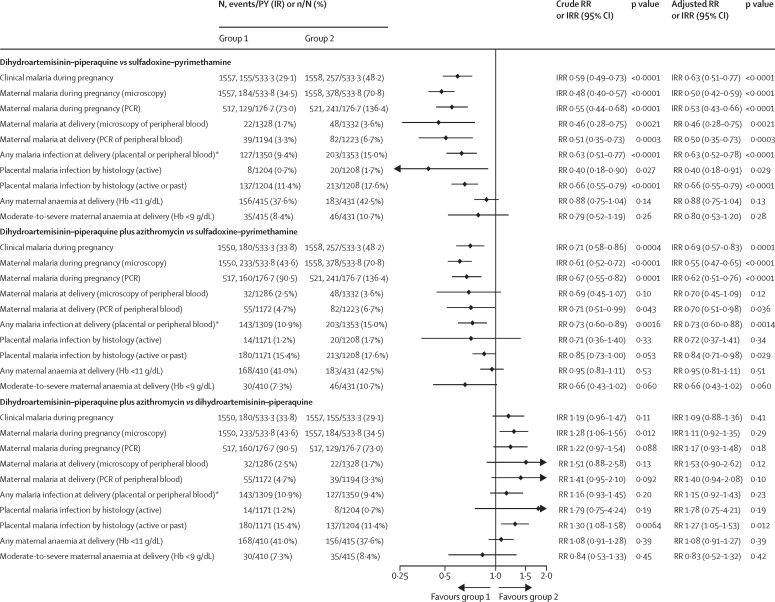
Malaria infection and maternal anaemia outcomes (modified intention-to-treat population) The crude RR values were obtained from log-binomial regressions with the stratification factors site and gravidity (paucigravidae and multigravidae) included as covariates. Hb=haemoglobin concentration. IR=incidence rate. IRR=incidence rate ratio. ITT=intention-to-treat. PCR=polymerase chain reaction. PY=person-year. RR=risk ratio. *Data are N, events/person-years (incidence rate) or n/N (%). †Post-hoc analysis.

Analysis of maternal endpoints showed that compared with the sulfadoxine–pyrimethamine group, women in the dihydroartemisinin–piperaquine group had a 41% (95% CI 27–51) reduction in clinical malaria, a 52% (43–60) reduction in malaria infection detected by microscopy during pregnancy, and a 34% (21–45) reduction in placental malaria at delivery (figure-3, appendix pp-30–31); these reductions for the dihydroartemisinin–piperaquine plus azithromycin group were 29% (95% CI 14–42), 39% (28–48), and 15% (0–27). RRs for maternal anaemia did not differ significantly between groups (figure-3, appendix p-32). Participants in the sulfadoxine–pyrimethamine groups had higher maternal gestational weight gain during pregnancy and higher mean mid-upper arm circumference (MUAC) at the time of birth (table 2). This effect on gestational weight was observed in all three countries (appendix pp 17–18).

The proportion of women with STIs or bacterial vaginosis at enrolment was similar across groups (table 1). 193 (14%) of 1371 participants had chlamydia at enrolment. At term, compared with ten (2%) of 410 women in the sulfadoxine–pyrimethamine group, chlamydia infections were reported in 40 (10%) of 416 in the dihydroartemisinin–piperaquine group (RR 4·11, 95% CI 2·09–8·08; p<0·0001) and 30 (7%) of 421 in the dihydroartemisinin–piperaquine plus azithromycin group (2·90, 1·44–5·85; p=0·0028; appendix p 33). In a post-hoc analysis, there was a non-statistically significant higher risk of adverse pregnancy outcomes (composite primary endpoint) in participants with chlamydia infection compared with those without (RR 0·65, 95% CI 0·39–1·07; p=0·092). The proportion of women with other STIs or bacterial vaginosis in the third trimester did not differ significantly between groups (appendix p 33).

Few women vomited after drug administration. 12 (0·2%) of 6685 sulfadoxine–pyrimethamine, 19 (0·3%) of 7014 dihydroartemisinin–piperaquine, and 23 (0·3%) of 6849 dihydroartemisinin–piperaquine plus azithromycin treatment courses were vomited within 30 min (table 3). One (0·1%) of 1552 women in the sulfadoxine–pyrimethamine group, two (0·1%) of 1558 in the dihydroartemisinin–piperaquine group, and four (0·3%) of 1556 in the dihydroartemisinin–piperaquine plus azithromycin group vomited after their first course of treatment (the only course when azithromycin was coadministered with dihydroartemisinin–piperaquine; table 3). All three regimens were well tolerated (table 3; appendix pp 20–21), but vomiting, nausea, and dizziness were more common in the first 3 days after dihydroartemisinin–piperaquine (13 [3·2%], 14 [3·4%], and 15 [3·7%] of 410 women visited at home, respectively) than sulfadoxine–pyrimethamine (one [0·3%], one [0·3%], and zero [0%] of 384 women visited at home, respectively; appendix pp 20–21). The addition of azithromycin to dihydroartemisinin–piperaquine was associated with significantly more vomiting than with dihydroartemisinin–piperaquine alone (p=0·0033; table 3).

**Table 3:** Safety and tolerability endpoints (safety population)

		**Sulfadoxine–pyrimethamine**	**Dihydroartemisinin–piperaquine**	**Dihydroartemisinin–piperaquine plus azithromycin**	**p value**		
					Dihydroartemisinin–piperaquine *vs* sulfadoxine–pyrimethamine	Dihydroartemisinin–piperaquine plus azithromycin *vs* sulfadoxine–pyrimethamine	Dihydroartemisinin–piperaquine plus azithromycin *vs* dihydroartemisinin–piperaquine
**Prevalence measures, n/N (%)**							
Vomiting within 30 min after study drug administration							
Vomiting dose (first course), events/participants (%)		1/1552 (0·1%)	2/1558 (0·1%)	4/1556 (0·3%)	0·57	0·18	0·41
	Vomiting dose at least once (any course), events/participants (%)	12/1553 (0·8%)	19/1561 (1·2%)	23/1557 (1·5%)	0·21	0·063	0·53
	Vomiting dose (any course), events/number of courses (%)	12/6685 (0·2%)	19/7014 (0·3%)	23/6849 (0·3%)	0·26	0·073	0·49
QTc >500 ms on ECG after the third dose*							
	First course	NA	2/97 (2·1%)	3/84 (3·6%)	NA	NA	0·56
	Middle course	NA	0/80	0/75	NA	NA	1·00
	Last course	NA	1/77 (1·3%)	0/62	NA	NA	1·00
**Continuous measures, mean (SD)**							
Change in QTc (after third dose–before first dose), ms*							
	All measurements	NA	18 (21·4)	23 (37·0)	NA	NA	0·15
	First course	NA	27 (21·0)	36 (25·9)	NA	NA	0·011
	Middle course	NA	16 (19·2)	22 (20·4)	NA	NA	0·13
	Last course	NA	9 (20·1)	6 (54·4)	NA	NA	0·49
**Incidence measures, events (incidence per person-year at risk) or prevalence measure (n, N [%])**							
Adverse events							
	Dizziness	4 (0·8)	51 (9·5)	38 (7·2)	<0·0001	<0·0001	0·16
	Vomiting	4 (0·8)	41 (7·7)	71 (13·5)	<0·0001	<0·0001	0·0033
	Nausea	2 (0·4)	44 (8·2)	35 (6·7)	<0·0001	0·0001	0·30
	Abdominal pain	6 (1·1)	11 (2·1)	14 (2·7)	0·27	0·092	0·52
	Diarrhoea	2 (0·4)	5 (0·9)	6 (1·1)	0·29	0·19	0·75
	Headache	12 (2·3)	17 (3·2)	18 (3·4)	0·42	0·32	0·84
	Rash	0 (0·0)	2 (0·4)	0 (0·0)	0·97	1·00	0·97
SAEs and grade 3–4 AEs (pregnant woman)							
	Any	95 (17·7)	79 (14·8)	92 (16·9)	0·34	0·96	0·32
	Maternal mortality	1/1553 (0·1%)	2/1561 (0·1%)	3/1557 (0·2%)	0·57	0·34	0·66
By System Organ Class							
	Blood and lymphatic system disorders	2 (0·4)	0 (0·0)	2 (0·4)	0·99	0·82	0·99
	Cardiac disorders	0 (0·0)	0 (0·0)	2 (0·4)	1·00	0·99	0·99
	Infections and infestations	10 (1·9)	14 (2·6)	12 (2·3)	0·35	0·44	0·88
	Injury, poisoning, and procedural complications	2 (0·4)	1 (0·2)	0 (0·0)	0·58	0·99	0·99
	Investigations	0 (0·0)	0 (0·0)	2 (0·4)	1·00	0·99	0·99
	Neoplasms benign, malignant, and unspecified (including cysts and polyps)	1 (0·2)	0 (0·0)	0 (0·0)	0·99	0·99	1·00
	Nervous system disorders	1 (0·2)	0 (0·0)	0 (0·0)	0·99	0·99	1·00
	Pregnancy, puerperium, and perinatal conditions	74 (13·7)	53 (9·9)	71 (13·3)	0·084	0·93	0·070
	Psychiatric disorders	0 (0·0)	2 (0·4)	0 (0·0)	0·99	1·00	0·99
	Respiratory, thoracic, and mediastinal disorders	1 (0·2)	2 (0·4)	3 (0·2)	0·44	0·92	0·52
	Surgical and medical procedures	3 (0·6)	6 (1·1)	0 (0·0)	0·31	0·99	0·99
	Vascular disorders	1 (0·2)	1 (0·2)	0 (0·0)	0·98	0·99	0·99
SAEs and grade 3–4 AEs (infant)							
	Any	75 (49·2)	70 (42·4)	76 (47·8)	0·95	0·88	0·93
By System Organ Class							
	Congenital, familial, and genetic disorders	19/1397 (1·4%)	21/1399 (1·5%)	15/1383 (1·1%)	0·42	0·45	0·13
	Gastrointestinal disorders	0 (0·0)	0 (0·0)	1 (0·7)	1·00	1·00	1·00
	General disorders and administration site conditions	1 (0·7)	2 (1·3)	4 (2·0)	0·57	0·54	0·97
	Infections and infestations	24 (16·2)	14 (9·3)	17 (11·4)	0·24	0·31	0·85
	Pregnancy, puerperium, and perinatal conditions	22 (14·2)	24 (13·3)	21 (12·8)	0·86	0·94	0·80
	Respiratory, thoracic, and mediastinal disorders	9 (5·4)	8 (5·3)	18 (12·1)	0·85	0·12	0·18
	Skin and subcutaneous tissue disorders	0 (0·0)	1 (0·7)	0 (0·0)	0·99	1·00	0·99

AE=adverse event. ECG=electrocardiogram. NA=not applicable. QTc=corrected QT interval. SAE=serious adverse event.

* ECGs were only conducted in the two dihydroartemisinin-piperaquine groups.

Maternal mortality, congenital anomalies, or other serious adverse events did not differ significantly between treatment groups among pregnant women or their infants, overall or by MedDRA System Organ Class (table 3). The incidence of serious adverse events in mothers was 17·7 per 100 person-years in the sulfadoxine–pyrimethamine, 14·8 per 100 person-years in the dihydroartemisinin–piperaquine group, and 16·9 per 100 person-years in the dihydroartemisinin–piperaquine plus azithromycin group; in infants the incidence of serious adverse events was 49·2 per 100 person-years in the sulfadoxine–pyrimethamine group, 42·4 per 100 person-years in the dihydroartemisinin–piperaquine group, and 47·8 per 100 person-years in the dihydroartemisinin–piperaquine plus azithromycin group. Mean change in QTc interval after the first course was significantly longer with dihydroartemisinin–piperaquine plus azithromycin (36 ms [SD 25·9]) than with dihydroartemisinin–piperaquine alone (27 ms [21·0]; mean difference 8·8 ms, 95% CI 2·0–15·7; p=0·011; table 3; appendix p 22). Prolongation of the QTc interval of more than 60 ms after the first course occurred in five (5%) of 97 women in the dihydroartemisinin–piperaquine group compared with ten (11·9%) of 84 with dihydroartemisinin–piperaquine plus azithromycin group (RR 2·21, 95% CI 0·80–6·16; p=0·13), and two (2%) of 97 compared with three (3%) of 84 women in the dihydroartemisinin–piperaquine plus azithromycin group had QTc values greater than 500 ms (RR 1·69, 95% CI 0·29–9·76; p=0·56). All these events were asymptomatic. Mean QTc prolongation decreased significantly with each subsequent course of dihydroartemisinin–piperaquine (last *vs* first, paired *t* test; p=0·0002).

## Discussion

In this trial, which compared standard of care with monthly IPTp with sulfadoxine–pyrimethamine with monthly IPTp with dihydroartemisinin–piperaquine and monthly IPTp with dihydroartemisinin–piperaquine plus azithromycin, the use of dihydroartemisinin–piperaquine was associated with reductions in clinical malaria, malaria infection detected by microscopy during pregnancy, and placental malaria at the time of birth. However, despite these reductions in malaria, the risk of adverse pregnancy outcomes (composite primary endpoint) was significantly lower in the sulfadoxine–pyrimethamine group than in both dihydroartemisinin–piperaquine groups. Newborn babies in the sulfadoxine–pyrimethamine group had a lower risk of low birthweight and of being small for gestational age compared with the dihydroartemisinin–piperaquine groups. Birthweight for gestational age Z scores at birth, a marker of fetal growth, were higher in the sulfadoxine–pyrimethamine group, as were Z scores for estimated fetal weight obtained by ultrasound. No statistical differences were reported in mean gestational age at birth, preterm delivery, fetal loss, or neonatal mortality. The results were similar in all three countries, including northeastern Tanzania, where 40% of parasites carried the highly resistant sextuple *dhfr*/*dhps* mutant haplotype containing the *dhps* Ala581Gly mutation. Adding one dose of azithromycin at enrolment to monthly IPTp with dihydroartemisinin–piperaquine did not result in a significant difference in the composite primary endpoint of adverse pregnancy outcomes, yet, it increased the incidence of vomiting and the mean change in QTc interval after the first course. These results suggest that replacing sulfadoxine–pyrimethamine with dihydroartemisinin–piperaquine for IPTp, alone or combined with azithromycin, might reduce the risk of malaria infection during pregnancy, but may result in an increase in adverse pregnancy outcomes, even in areas with very high sulfadoxine–pyrimethamine resistance.

In a previous meta-analysis^4^ a modest, but non-significant, reduction of 17% in the number of adverse pregnancy outcomes favouring dihydroartemisinin–piperaquine versus sulfadoxine–pyrimethamine was reported. This reduction was driven by a 61% reduction in fetal loss (miscarriage or stillbirth), but adverse livebirth outcomes did not differ significantly between groups.^4^ These findings led WHO to recommend larger definitive studies to determine whether IPTp with dihydroartemisinin–piperaquine improves adverse pregnancy outcomes compared with sulfadoxine–pyrimethamine in areas with high sulfadoxine–pyrimethamine resistance. Our results are consistent with those from a third trial in eastern Uganda, which showed that monthly IPTp with dihydroartemisinin–piperaquine did not decrease the number of adverse pregnancy outcomes among livebirths or reduce fetal loss compared with monthly IPTp with sulfadoxine–pyrimethamine.^8^ A potential explanation for the difference could be the use of monthly courses in the later trials^8^ instead of the three to four courses used in the first two trials.^5^, ^6^ All of these trials, including ours, show that dihydroartemisinin–piperaquine is the superior antimalarial, resulting in 40–90% fewer malaria infections during pregnancy.^5^, ^6^, ^8^, ^9^

The observed 31% lower risk of low birthweight with sulfadoxine–pyrimethamine versus dihydroartemisinin–piperaquine (the reciprocal of the 44% increase in risk, relative to sulfadoxine–pyrimethamine) and 25% lower risk versus dihydroartemisinin–piperaquine plus azithromycin (the reciprocal of the 33% increase in risk, relative to sulfadoxine–pyrimethamine) are similar to the 18% reduction in low birthweight associated with daily iron supplementation in high-quality trials,^15^ and the 23% reduction in low birthweight associated with insecticide-treated bednets.^16^ The reductions in low birthweight and small for gestational age in the sulfadoxine–pyrimethamine group were reported even though these newborn babies were born to mothers at nearly double the risk of malaria infection during pregnancy. Our results suggest that the effect of sulfadoxine–pyrimethamine on the composite primary endpoint of adverse pregnancy outcomes and on birthweight is driven by the effect on intrauterine fetal growth rather than the duration of gestation. The effect of sulfadoxine–pyrimethamine on the mean birthweight were observed across all three countries, and thus was independent of the degree of malaria transmission and sulfadoxine–pyrimethamine resistance in an area. Findings from subgroup analyses suggested that the effect of sulfadoxine–pyrimethamine on the composite primary endpoint of adverse pregnancy outcomes might be greatest in multigravidae, who are at lower risk of malaria-associated adverse pregnancy outcomes than paucigravidae.^1^

The mechanisms by which sulfadoxine–pyrimethamine might stimulate fetal growth are not fully understood. A potent non-malarial effect of sulfadoxine–pyrimethamine might exist that outweighed any improvements in birthweight associated with better malaria prevention in the dihydroartemisinin–piperaquine group.^17^ The non-malarial effect of sulfadoxine–pyrimethamine might involve the antibacterial properties of sulfadoxine, either directly by preventing or suppressing bacterial infections or indirectly by altering the intestinal or vaginal microbiome.^18^ Sulfadoxine–pyrimethamine might also have immunomodulatory effects, similar to those described for antifolate co-trimoxazole,^19^ or a combination of these mechanisms. Although the differences were small, women in the sulfadoxine–pyrimethamine group had significantly higher MUAC scores at delivery and better gestational weight gain during pregnancy. Thus, our results support findings from a study of 105 women from Malawi that the effect on fetal growth might,^18^ at least in part, be mediated by the promotion of maternal weight gain during the second and third trimesters of pregnancy. In this study,^18^ the prevalence of enteroaggregative *Escherichia coli* was dose-dependently reduced by sulfadoxine–pyrimethamine. After three or more doses, women who received IPTp with sulfadoxine–pyrimethamine were 90% less likely to be infected with enteroaggregative *E coli* than those who received dihydroartemisinin–piperaquine.^18^ This finding coincided with higher maternal weight gain and other nutritional indicators, such as MUAC and BMI.^18^ An alternative explanation for the observed differences in birthweight between the sulfadoxine–pyrimethamine and dihydroartemisinin–piperaquine groups could be an unexplained negative effect of dihydroartemisinin–piperaquine on fetal growth or maternal weight gain during pregnancy. However the Z scores for fetal weight gain in the dihydroartemisinin–piperaquine arms were close to zero, suggesting the fetal growth was close to the average of the INTERGROWTH reference population used.

The number of serious adverse events was similar across all three groups in mothers and infants. IPTp with dihydroartemisinin–piperaquine was well tolerated, which is essential for drugs used for chemoprevention when most recipients are asymptomatic. Although dizziness, vomiting, and nausea were more frequently reported in the dihydroartemisinin–piperaquine groups than in the sulfadoxine–pyrimethamine group, these adverse events were reported by less than 5% of participants and did not affect adherence or dropouts. However, the addition of azithromycin resulted in significantly more vomiting than with dihydroartemisinin–piperaquine alone.

Furthermore, adding azithromycin to dihydroartemisinin–piperaquine was not associated with a reduction in the composite primary endpoint of adverse pregnancy outcomes, any of the individual components of the composite primary endpoint, or a reduction in STIs and bacterial vaginosis. The high prevalence of curable STIs and bacterial vaginosis at enrolment (16·9%) in this study, which is similar to that reported in a meta-analysis,^10^ highlights the importance of accurate diagnosis and treatment of curable STIs and other RTIs as part of antenatal care in sub-Saharan Africa. The evidence base of the benefits of adding azithromycin to IPTp in malaria-endemic settings is contradictory.^19^, ^20^, ^21^

We considered more frequent doses of azithromycin during pregnancy at the design stage. However, after discussion with WHO, this idea was disregarded in favour of a single dose of azithromycin because of concerns about the potential effect of azithromycin on macrolide resistance when given presumptively several times in pregnancy to all pregnant women even when asymptomatic. None of the pregnancy studies summarised, including our trial, involved partner screening and treatment for STIs; therefore, reinfection cannot be ruled out. The risk of reinfection is an important consideration, which might explain the superior effect of sulfadoxine–pyrimethamine on chlamydia infection. Women in the dihydroartemisinin–piperaquine plus azithromycin group received 2 g of azithromycin at the enrolment visit, double the WHO-recommended first-line treatment for chlamydia (1 g on 1 day). By contrast, participants in the IPTp with sulfadoxine–pyrimethamine group received a median of five courses of 1·5 g of sulfadoxine throughout pregnancy. Sulfadoxine is a sulfonamide derivative. Sulfisoxazole, another sulfonamide, which inhibits *Chlamydia trachomatis*,^22^ was used to treat chlamydia in the 1980s.^23^

Because both dihydroartemisinin–piperaquine and azithromycin can cause QT prolongation,^24^, ^25^ nested cardiac monitoring was completed in a subgroup of women. Adding azithromycin at the enrolment visit resulted in significantly longer QT prolongation compared with dihydroartemisinin–piperaquine alone (36 ms *vs* 27 ms), and a greater proportion of women had QTc values exceeding 500 ms. All QTc prolongation was asymptomatic. QTc prolongation progressively declined with subsequent IPTp courses, including in the dihydroartemisinin–piperaquine group that did not receive azithromycin, consistent with previous chemoprevention trials with dihydroartemisinin–piperaquine in pregnant women^26^, ^27^ or children,^24^, ^28^ suggesting tolerance to dihydroartemisinin–piperaquine-associated QT prolongation with monthly dosing.

The strengths of this trial are that it was powered to detect differences in the composite primary endpoint of adverse pregnancy outcomes and used ultrasound to determine gestational age for the accurate assessment of preterm delivery, small for gestational age, and estimated fetal weight gain during pregnancy. It also contained serial measures of gestational weight and assessment of malaria infection and common STIs and bacterial vaginosis. Furthermore, adherence to the follow-up schedule was good, with 91% of scheduled visits attended and 92% of participants included in the primary analysis, and the trial was done in areas with both high and very high sulfadoxine–pyrimethamine resistance.

A limitation of this study is that only the first-day dose of each course was given as directly observed therapy in the clinic: although all sulfadoxine–pyrimethamine courses were given under direct supervision, the second and third day doses of the 3-day dihydroartemisinin–piperaquine course and the second day dose of the 2-day azithromycin course were self-administered at home. Even though the self-reported adherence to all doses of dihydroartemisinin–piperaquine exceeded 97·5%, this was verified in less than 10% of IPTp courses by unannounced home visits. Furthermore, 9% of monthly courses were skipped. Incomplete adherence or spacing beyond 1 month between some of the courses could have resulted in inadequate piperaquine drug concentrations,^29^ which might partly explain the 10% malaria prevalence at delivery in the two groups receiving dihydroartemisinin–piperaquine. Another explanation is that pharmacokinetic and pharmacodynamic modelling suggests that monthly dosing, although effective, does not provide complete protection from malaria infection to all women, even when fully adhered to.^29^ A second limitation is the lower than usual levels of malaria transmission in all three countries, resulting in less placental malaria and a lower population-attributable risk of malaria than in previous studies at these sites. This disparity could partly explain the reduced effect of IPTp with dihydroartemisinin–piperaquine on adverse pregnancy outcomes relative to the non-significant reduction of 17% observed in a meta-analysis^4^ of the first two trials,^5^, ^6^ and the 12% in the subsequent trial in Uganda,^8^ and thus a larger difference in favour of the malaria-independent effect of sulfadoxine–pyrimethamine. Third, a surprising finding was that malaria RDTs detected more malaria infections at enrolment than qPCR. Some of these might have been false-positive infections because malaria RDTs can detect parasite circulating antigens for several weeks after clearance of *P falciparum* infections, for example after treatment. Also, our qPCR method, which used 50 μL of whole blood obtained from dried blood spots, will not be as sensitive as can be achieved with larger volumes obtained from whole blood pellets.^30^ A fourth limitation is the open-label comparison between sulfadoxine–pyrimethamine and dihydroartemisinin–piperaquine, which could have biased the assessment of tolerance and safety outcomes. Finally, there might have been selection bias in our modified ITT analysis due to the omission of participants with missing outcome data and in our per-protocol estimates in the presence of imperfect adherence, and measurement bias in our ITT analysis might affect the generalisability of our findings.

In conclusion, replacing IPTp with sulfadoxine–pyrimethamine with dihydroartemisinin–piperaquine will probably result in marked and clinically relevant reductions in clinical malaria and maternal malaria infection during pregnancy in areas with high sulfadoxine–pyrimethamine resistance. However, countries that choose to do so need to be aware that replacing sulfadoxine–pyrimethamine with dihydroartemisinin–piperaquine might increase the absolute number of adverse pregnancy outcomes compared with IPTp with sulfadoxine–pyrimethamine, even in areas with very high sulfadoxine–pyrimethamine resistance. We hypothesise that in these high resistance areas, sulfadoxine–pyrimethamine is likely to continue to provide benefits through malaria-independent effects on fetal growth, which might have masked any reductions in malaria-associated adverse pregnancy outcomes with dihydroartemisinin–piperaquine. Combining the superior antimalarial effects of dihydroartemisinin–piperaquine and the non-malarial effect of sulfadoxine–pyrimethamine on fetal growth merits future investigation. Further studies of how IPTp with sulfadoxine–pyrimethamine promotes gestational weight gain and fetal growth are also warranted.

## Data sharing

The protocol is available in the appendix (p 34) to this publication. Individual participant data will be available from the Worldwide Antimalarial Resistance Network data repository approximately 3 months after publication.

## Declaration of interests

We declare no competing interests.
