# Supplementary material for: Effect of monthly intermittent preventive treatment with dihydroartemisinin–piperaquine with and without azithromycin versus monthly sulfadoxine–pyrimethamine on adverse pregnancy outcomes in Africa: a double-blind randomised, partly placebo-controlled trial
Source: Lancet. 2023 Mar 25;401(10381):1020–36. doi: 10.1016/S0140-6736(22)02535-1 (PMC10063957; doi:10.1016/S0140-6736(22)02535-1)
Supplement: Supplementary appendix [file mmc1.pdf]

THE LANCET

Supplementary appendix

This appendix formed part of the original submission and has been peer reviewed. We post it as supplied by the authors.

Supplement to: Madanitsa M, Barsosio HC, Minja DTR, et al. Effect of monthly intermittent preventive treatment with dihydroartemisinin–piperaquine with and without azithromycin versus monthly sulfadoxine–pyrimethamine on adverse pregnancy outcomes in Africa: a double-blind randomised, partly placebo-controlled trial . *Lancet* 2023; published online March 10. https://doi.org/10.1016/S0140-6736(22)02535-1.

Supplementary appendix

Supplement to: Madanitsa M, Barsosio H, Minja, DTR, et al. Monthly intermittent preventive treatment with dihydroartemisinin-piperaquine with and without azithromycin versus monthly sulfadoxine-pyrimethamine to reduce adverse pregnancy outcomes in Africa: a double-blind randomised partially placebo-controlled superiority trial

Table of Content

Supplemental methods 2

Supplement 1: Details of study interventions 2

Supplement 2: Screening for malaria infection at enrolment 2

Supplement 3: Procedures for cardiac monitoring 3

Supplement 4: Methods used to determine birthweight, gestational age and gestational age z-scores 3

Supplement 5: Definition of endpoints 4

Supplement 6: Deviations from study protocol 6

Supplement 7: Laboratory procedures 7

Supplement 8: Statistical methods 7

Supplement 9: Supplemental details of trial profile 8

Supplemental references 9

Supplemental tables 10

Supplemental figures 24

Supplemental methods

Supplement 1: Details of study interventions

IPTp-SP

A standard single-day stat course of 3 quality-assured SP tablets (500 mg of sulfadoxine and 25 mg of pyrimethamine) was provided at enrolment and each subsequent monthly ANC visit in the 2nd and 3rd trimester. Each tablet contained 500 mg of sulfadoxine and 25 mg of pyrimethamine (unbranded generic SP, Medopharm, Chennai, India, and imported by Durbin PLC, Hayes, United Kingdom). Medopharm provided the certificate of analysis.

IPTp-DP

Participants in the IPTp-DP arm received 3 to 5 tablets of DP daily for three days based on bodyweight and provided at enrolment and each subsequent monthly ANC visit in the 2nd and 3rd trimester. The weight at enrolment was used to define the dose at subsequent scheduled visits. At the enrolment visit, participants also received 2 gr of placebo-azithromycin, given as two tablets of 500 mg (Universal corporation Ltd, Nairobi, Kenya) daily (1 gram) for two consecutive days (cumulative dose 2 grams), given at the same time as the first and second daily dose of DP on enrolment.

Each tablet of DP (Eurartesim, Alfasigma, Bologna, Italy) contained 40 mg of dihydroartemisinin and 320 mg of piperaquine. The dose of DP is equivalent to the standard treatment (case-management) dose for malaria in adults recommended by WHO with a target dose (range) of 4 (2-10) mg/kg bodyweight per day dihydroartemisinin and 18 (16-27) mg/kg bodyweight per day piperaquine given once a day for 3 days for adults and children weighing>= 25 kg (36-<60 kg: 3 tablets, 60-<80 kg:4 tablets, >=80 kg 5 tablets).[1]

IPTp-DPAZ

Participants in the IPTp-DP arm received the same dose of DP as in the IPTp-DP arm at enrolment and each scheduled monthly visit. This was combined with 2 gr of azithromycin at the enrolment visit, given orally as two tablets of 500mg azithromycin once daily for two consecutive days (Troza, Universal corporation Ltd, Nairobi, Kenya). The dose of AZ is commonly used for the treatment of STIs/RTIs.[2]

Drug vomiting

The full dose was repeated in case of vomiting within 30–60 min for any of the three drugs. If the repeat dose was vomited, the IPTp dose was withheld.

Supplement 2: Screening for malaria infection at enrolment

In Kenya and Tanzania, women are routinely screened for malaria infection at the first antenatal booking visit, which is part of the national guidelines in Tanzania and is common practice in the malaria-endemic counties in Kenya, but not in Malawi. Therefore, in Kenya and Tanzania, all participants were screened for malaria infection at enrolment using histidine-rich protein 2 (HRP2)/plasmodium lactate dehydrogenase (pLDH) combination mRDTs (CareStart™ Malaria Pf/PAN (HRP2/pLDH) Ag Combo mRDT). All mRDT-positive women in Kenya and those in the SP-arm in Tanzania received a standard 3-day treatment dose of artemether-lumefantrine, with the first dose provided as directly observed therapy in the clinic and the remaining doses self-administered at home. The first dose of IPTp was delayed until the next scheduled visit four weeks later for women who received artemether-lumefantrine at enrolment. In Tanzania, mRDT positive women in the IPTp-DP or IPTp-DPAZ arm received their first course of DP or DPAZ at enrolment instead of artemether-lumefantrine.

Supplement 3: Procedures for cardiac monitoring

Procedures

Nested cardiac monitoring was conducted in a sub-group of 189 participants in Malawi and Kenya. ECGs were taken before the first dose and then again 4 hours after the 3rd dose of the first course among women randomised to one of the two IPTp arms containing DP. ECGs were repeated approximately 1-2 and 3-4 courses later after the end of the second to the early third trimester and mid to end of the third trimester, respectively. ECGs were taken by dedicated staff at the study clinic, and the results were read by local clinicians. Half of the ECGs were transferred to a central ECG laboratory (Cardiabase, Nancy, France) for further reading using a computer-assisted, semi-automatic, on-screen measurement of the digital ECG waveforms (ECG Manager©, Cardiabase, Nancy, France), as described in detail by Funck-Brentano et al.[3]

Sample size

This nested study was designed to detect a difference of 10 milliseconds (ms) in QTc prolongation observed between the first and last course of DP with 90% power, which required 68 women per arm (standard deviation 25 ms, two-sided α=0.05, paired test). The study would also have approximately 80% power to detect a 10 ms difference in the mean QTc prolongation between the DP alone arm and the DPAZ arm with a standard deviation of approximately 20ms (two-sided α=0.05, two-sample t-test, assuming equal variance between arms).

Analysis

To obtain QTc values that are independent of the RR intervals, three different heart-rate correction methods were used, based on the generic formula generic $QTc = {QT}/{RR^{\beta}}$, where β is a correction exponent, the value of which depends on the method used;[3] for the Bazett method (QTcB), the value of β is 0.5; for Fridericia's method (QTcF), the value of β is 0.33. With the third method, the value of β was study-derived (QTcD), where β was calculated based on the value of the slope from the linear regression as determined using the log-transformed values of QT and RR and the equation log(QTc)=log(QT)-log(RR)*β. QTcD was calculated using the pre-dose ECGs at baseline (i.e. before the first dose of the first course of DP). We then applied the same study-derived correction factor to all subsequent ECGs.

To QTc values obtained by the three correction methods were then plotted against the corresponding RR values, and the slopes calculated for the three linear regressions lines based on the equation QTc = a + b*RR + e, where a is the intercept, b is the slope, and e is the residual error. The method with a slope closest to zero, i.e. a horizontal regression line, was then chosen as the best correction method and used as the primary method in the subsequent analyses.

The mean QTc values on day 3 and changes from baseline on day 3 were compared using linear regression. The results were expressed as mean difference (95% CI) between study arms with stratification factors gravidity and site entered as covariates. The proportion of participants with QTc values >480 ms, >500 ms for absolute values and >60 ms change from baseline were compared using log-binomial regression with study and baseline QTc values as covariates.

Supplement 4: Methods used to determine birthweight, gestational age and gestational age z-scores

Corrected birthweights

All birthweights in the analyses refer to corrected birthweights taken within seven days (168 hours) after birth. Birthweights taken more than 1 hour after delivery were corrected for the physiological fall in birth weight in breastfed infants occurring in the first days following delivery using daily

birthweight data collected from participants in one of the two sites in Tanzania during the trial. For newborn weight >1 hour after delivery, the corrected birthweight was imputed using a table of correction factors by Mtove et al. based on the sex of the infant, its measured weight (increasing from 2kg – 4kg in increments of 10g), and time from birth to weighing (in hourly increments from 1hr to 168hrs).[4] For newborns where sex was unavailable, the midpoint between girls and boys for that time point was used. For infants for which the exact hour of birth and /or measurement of (birth)weight was unavailable, the weighing timepoint was assumed to be 0 hours (if birth and measurement on the same day), then 24, 48, 72, etc. hours if the child was weighed 1, 2, 3, etc. days after delivery.

Gestational age at delivery

Gestational age in days at delivery was defined as the number of days between delivery and the date that gestational age was assessed at enrolment, plus the gestation age in days assessed at enrolment. The following methods were used in order of priority: Ultrasound data were used where available (99.8% [4671/4680]). The following biometric measures were used to define gestational age at enrolment: crown-rump length (CRL) was used for participants in the first trimester. In the second trimester, the combination of head circumference (HC) and femur length (FL) was used if both were available; otherwise, only the head circumference was used if femur length measures were unavailable, or only femur length if head circumference measures were unavailable. For the remaining 9 participants (0.2%), the date of the last menstrual period was used if the exact date was known, and otherwise, the gestational age was defined by fundal height measurement.

Z-scores for maternal and fetal growth monitoring

Z-scores for birthweight for gestational age for newborns with gestational ages at birth ranging between 168 and 300 days were calculated using the INTERGROWTH reference population, which is derived from a multi-ethnic cohort of low-risk, well-nourished mothers with uncomplicated pregnancies.[5] Z-scores for gestational weight gain were calculated using the INTERGROWTH reference population for Gestational Weight Gain [6] Z-scores for estimated fetal weight to monitor fetal growth were calculated for pregnancies with a gestational age between 22+0 and 40+0 weeks using the INTERGROWTH reference population for fetal growth.[7,8] Z-scores for newborn size at birth were calculated using the INTERGROWTH reference population for newborn size.[5]

Supplement 5: Definition of endpoints

Efficacy endpoints

Primary endpoints

- Adverse pregnancy outcome: A composite of either fetal loss (spontaneous miscarriage or stillbirth) or adverse live birth (defined as either low birth weight [<2500 grams], small-for-gestational-age [SGA, <10th percentile relative to INTERGROWTH-21st gender-specific chart], or preterm delivery [<37 weeks gestation]), or neonatal death.

Secondary maternal endpoints

- Clinical malaria, defined as a positive malaria mRDT (HRP2 or pLDH) or malaria smear in conjunction with documented fever (>37.5 ^{o}C) or reported fever in the past 48 hours.
- Any malaria infection during pregnancy, defined as the composite of malaria detected in the peripheral blood by mRDT (for point of care), or microscopy (not for point of care), or by PCR (not for point of care), from 2 weeks after enrolment and excluding delivery.
- Patent malaria infection during pregnancy: Malaria infection during pregnancy detected by microscopy and/or mRDT, from 2 weeks after enrolment and excluding delivery.

- Sub-patent malaria infection during pregnancy: Malaria infection during pregnancy detected by PCR, with negative microscopy and/or mRDT, from 2 weeks after enrolment and excluding delivery.
- Any malaria infection at delivery: Any *Plasmodium* infection detected in peripheral or placental blood by PCR, microscopy, mRDT or histopathology (active infection, i.e., acute or chronic infections)
- The individual components of the composite malaria infection endpoints at delivery
- Any placental malaria by histology (active or past infection)
- Placental Inflammation or chorioamnionitis detected by placental histology
- Mean haemoglobin (Hb in g/dL) in the third trimester, excluding delivery, using Hb measured in either venous or capillary blood.
- Maternal anaemia in the third trimester (any anaemia [Hb<11.0g/dL], moderate-severe anaemia [Hb<9.0g/dL], severe anaemia [Hb<7.0g/dL])
- Mean haemoglobin (Hb in g/dL) at delivery, measured just before delivery of the baby in either venous or capillary blood just before delivery.
- Maternal anaemia at delivery (any anaemia [Hb<11.0g/dL], moderate-severe anaemia [Hb<9.0g/dL], severe anaemia [Hb<7.0g/dL])
- Maternal mid-upper arm circumference (MUAC) at delivery
- Low maternal MUAC at delivery, defined as less than 23 cm measured at delivery or at the last time point in the third trimester if MUAC at delivery is not available
- Maternal MUAC gain in cm per week, defined as the maternal MUAC at the last measured time point at or before delivery (e.g., in the third trimester or at delivery) minus the MUAC enrolment, divided by the number of weeks between these dates
- Gestational weight gain in kilogram per week, defined as the maternal weight measured at the last time point before delivery (e.g., in the third trimester) minus the maternal weight at enrolment, divided by the number of weeks between these dates
- Z-scores for gestational weight gain using the INTERGROWTH reference

Secondary newborn endpoints

- Z-scores for femur length, head circumference and crown-rump length using the INTERGROWTH-21st reference
- Z-score for fetal growth using the INTERGROWTH-21st reference
- Mean birthweight in kilogram (for methods, see Corrected birthweights, above)
- Mean gestational age at delivery in days (for methods, see Gestational age at delivery, above)
- Z-score for birthweight using the INTERGROWTH-21st reference
- Z-scores for birthweight for gestational age using the INTERGROWTH-21st reference
- Low birth weight (LBW): A corrected birthweight < 2,500g measured
- Preterm birth (PTD): A gestational age of < 37 weeks at birth.
- Small-for-gestational-age: A birthweight <10th percentile of the INTERGROWTH-21st reference
- Adverse live birth, defined as either LBW, preterm or SGA (SGA-LBW-PT composite)
- Birth length in cm, defined as a length measured within 24 hours of birth
- Z-score for birth length at birth (cm) using the INTERGROWTH-21st reference
- Stunting at birth, defined as <3rd centile of birth length for gestational age using the INTERGROWTH-21st reference.

- Wasting at birth, defined as <3rd centile of corrected birthweight for gestational age using the INTERGROWTH-21st reference.
- Both stunting and wasting at birth
- Spontaneous miscarriage: Loss of foetus before 28 weeks gestational age
- Stillbirth: Loss of foetus at or after 28 weeks gestation or birth of foetus showing no signs of life
- Foetal loss: Stillbirth or miscarriage
- Early neonatal death, defined as the death of a live-born baby within the first week of life
- Perinatal death: stillbirths and deaths in the first week of life
- Neonatal death: death within 28 days of birth
- Composite of fetal loss and neonatal mortality
- Congenital malaria infection, defined as malaria infection detected in fetal cord blood or peripheral blood of the newborn at birth or within 7 days (168 hours) after birth by either mRDT (pLDH only) or microscopy
- Mean cord blood haemoglobin in g/dL, measured in umbilical cord blood at birth
- Fetal anaemia in g/dL, defined as Hb<12.5 g/dL in umbilical cord blood at birth
- Congenital anomalies: Physical abnormality of live-born baby detected at delivery or newly noted anomaly during the infant follow-up visits (7 days or 6-8 weeks post-natal).

Safety endpoints

- QTc-prolongation, defined as mean QTc, mean delta QTc, and QTc values >480ms and >500ms, or delta QTc >60ms, alone or combined with cardiac symptoms (e.g. syncope)
- Maternal mortality, defined as death at any time from enrolment onward until 42 days after delivery or termination of pregnancy
- Other Serious Adverse Events (SAEs) and Adverse Events (AEs), defined according to the Medical Dictionary for Regulatory Activities (MedDRA), overall and by system organ class and preferred term

Tolerance endpoints

- Vomiting of study drug, defined as vomiting within 30 minutes of taking the study drug at any scheduled administration
- Later vomiting at any point within 96hrs (4 days) of the first drug administered during a treatment cycle, excluding drug vomiting within 30 minutes
- Nausea at any point within 96hrs (4 days) of the first drug administered during a treatment cycle
- Dizziness at any point within 96hrs (4 days) of the first drug administered during a treatment cycle
- Gastrointestinal complaints are defined as nausea or vomiting at any point within 96hrs (4 days) of the first drug administered during a treatment cycle, excluding drug vomiting within 30 minutes

Supplement 6: Deviations from study protocol

The study protocol included two outcomes that will be reported in subsequent publications, including 1) the impact of azithromycin on macrolide resistance in Pneumococcus detected in maternal nasopharyngeal samples, and 2) the impact on colony composition of the maternal vaginal microbiota and intestinal microbiota of mother and infant.

Supplement 7: Laboratory procedures

Blood smears

Blood smears were stained with Giemsa and read in duplicate by certified microscopists. A blood smear was considered negative when the examination of 100 high-power fields did not reveal the presence of asexual parasites. A third microscopist would read any discrepancies between the first and second reader. Blood smear results were considered discrepant if one reader scored them as negative and another as positive.

PCR

Dried blood spots for PCR were taken at enrolment and delivery in all women and in a random sample of one-third of all participants in each site during the scheduled follow-up visits between enrolment and delivery (i.e. approximately every four weeks). DNA was extracted from dried blood spots (50 microL) using a commercial DNA extraction kit (E Z 96 Tissue DNA Kit®, Omega BIO-TEK, VWR, Denmark, vacuum protocol) described in detail previously.[9] The resulting DNA extracts were tested for the presence of *P. falciparum* DNA on a VIIA 7 Applied Biosystems Real-Time PCR System (Applied Biosystems, CA) using the qPCR method and conditions as described by Hofmann et al.[10] targeting the high-copy telomere-associated repetitive element 2 (TARE-2,*250 copies/genome) of *P. falciparum.* DNA of 3 mL was added into 15 mL of PowerUpTM SYBRTM Green master mix (Applied Biosystems, CA).[9] The method has a lower limit of detection of 0.03 to 0.15 parasites per µL of blood.[10]

SP resistance markers

Illumina targeted amplicon sequencing of *P. falciparum* genes *Pfdhfr* and *Pfdhps* was used to assess molecular markers of SP resistance in *Plasmodium*-positive samples as described previously.[11] Only full haplotype profiles at codons 51, 59, 108 and 164 of *Pfdhfr* and codons 431, 436, 437, 540, 581 and 613 of *Pfdhps* were used for analysis. Mixed haplotype infections were omitted.

Supplement 8: Statistical methods

Sample size calculations

The study was designed to achieve 80% power trial to detect a ≥20% reduction in the primary endpoint from 21.3% with IPTp-SP to 17.0% (RR=0.80) in any of the intervention arms containing DP (α=0.05) and required a sample size of 4,680 participants (1,560 per arm). The 21.3% primary endpoint prevalence in the control arm was the observed mean prevalence in the IPTp-SP arms of our previous trials in the same sites in Kenya[12] and Malawi,[13] which also provided LLINs on enrolment, and in observational cohort studies in Tanzania.[14]

The sample size allowed for 13.7% loss to follow-up was approximately double the loss to follow-up rate for this primary composite endpoint in our recently completed malaria in pregnancy trials in Malawi (N=1873, across two arms[13]) and Kenya (N=1546, across three arms[12]). These previous trials involved similar sample sizes per country as in the proposed study. However, the higher rate of loss to follow-up was used for the sample size calculations since not all hospitals in each country were part of the previous studies.

No alpha adjustment for multiplicity was made in the sample size calculations for making two comparisons as part of the primary objective (DP v SP and DP+AZ v SP).

Methods used for non-convergence

Log-binomial regression was used for the primary and dichotomous secondary endpoints and compared using risk ratios (RR) and corresponding 95% confidence intervals (CIs). In the case of non-

convergence of the log-binomial model, modified Poisson regression with robust standard errors was used.

Methods for imputations of missing primary endpoint

A sensitivity analysis of the primary endpoint was conducted using non-responder imputation of missing endpoints to determine the impact of attrition bias.[15] Multiple imputations were conducted using Markov chain Monte Carlo algorithms using the 'mi impute logit' command to impute missing primary endpoint values based on covariates. Fifty imputations were performed. We then used the 'mi estimate' command to fit the log-binomial regression models to estimate the crude and adjusted risk ratios based on the imputed data. The following covariables were used in the imputation model: Study arm, the stratification factors site and gravidity (paucigravidae and multigravidae), and the covariables used in the adjusted models, namely patent malaria status at enrolment (positive/negative), gestational age in days at enrolment (continuous), socioeconomic status (socioeconomic index calculated using principal component analysis) (continuous), season (based on average ranked rainfall during the last 6 months of pregnancy) (continuous), malaria transmission intensity by site (based on the prevalence of malaria at enrolment) (continuous)and the degree of SP resistance by site (very high vs high based on the prevalence of the dhps-A581G mutation >40% vs <= 40%).

Methods used for linear mixed models to analyse the effect on estimated fetal weight

To assess the treatment effect on Z-scores for estimated fetal weight (EFW), a linear mixed-effects model was fitted using the Stata command mixed. The unadjusted linear mixed models contained visit number (post-enrolment, around 25-28 and 32-35 weeks, as a categorical variable), arm, the interaction between visit number and study arm, the stratification factors site and gravidity (paucigravidae and multigravidae) as fixed effect and participant as random effects to account for the clustering within subject. The adjusted linear mixed models also included the same six additional prespecified covariates (listed above under Methods for imputations of missing primary endpoint) as fixed-effect, and participant as random effects. The mixed models used the "variance component" variance-covariance structure, which assumes that between-participant variation and within-participant variation have independent normal distributions. Between-group and within-group differences, together with their 95%CI in EFW and its z-scores, were obtained using the Stata command lincom.

The linear mixed model does not include the baseline Z-score for estimated EFW because the international estimated fetal weight standards of the INTERGROWTH-21st Project only start at 22 weeks.[7] Only 40% (624/1,560) of the participants randomised to be included in the nested serial ultrasound study were recruited at 22 weeks gestation or later. Thus baseline EFW Z-scores are unavailable for 60%. The results of a sensitivity analysis that includes baseline EFW Z-scores as a covariate following multiple imputations of missing baseline observations among those enrolled before 22 weeks gestation are shown in Table-S8 (Appendix, p 23).[7]

Supplement 9: Supplemental details of trial profile

Reasons for not meeting the eligibility criteria

The reasons for not meeting the eligibility criteria included: <16 or >28 weeks pregnant (n=1114); fetal abnormality or non-viable pregnancy (US) (n=62); plans to relocate outside study area/resides outside study area (n=366); history of allergy to study drugs (n=13); HIV positive or not willing to be tested (n=356); received SP prior to enrolment (n=184); history of heart disease (n=2); multiple pregnancies (e.g. twins) (n=69); committed/enrolled in another study (n=61); negative pregnancy

test or ultrasound (n=5); took prohibited medication (high-dose folic acid) (n=2); other (but no further details provided) (n=70).

Supplemental references

1. World Health Organization. Guidelines for the treatment of malaria, third edition: World Health Organization; 2015.
2. Chico RM, Hack BB, Newport MJ, Ngulube E, Chandramohan D. On the pathway to better birth outcomes? A systematic review of azithromycin and curable sexually transmitted infections. *Expert review of anti-infective therapy* 2013; **11**(12): 1303-32.
3. Funck-Brentano C, Ouologuem N, Duparc S, et al. Evaluation of the effects on the QT-interval of 4 artemisinin-based combination therapies with a correction-free and heart rate-free method. *Sci Rep* 2019; **9**(1): 883.
4. Mtove G, Abdul O, Kullberg F, et al. Weight change during the first week of life and a new method for retrospective prediction of birthweight among exclusively breastfed newborns. *Acta Obstet Gynecol Scand* 2022; **101**(3): 293-302.
5. Villar J, Cheikh Ismail L, Victora CG, et al. International standards for newborn weight, length, and head circumference by gestational age and sex: the Newborn Cross-Sectional Study of the INTERGROWTH-21st Project. *Lancet* 2014; **384**(9946): 857-68.
6. Cheikh Ismail L, Bishop DC, Pang R, et al. Gestational weight gain standards based on women enrolled in the Fetal Growth Longitudinal Study of the INTERGROWTH-21st Project: a prospective longitudinal cohort study. *Bmj* 2016; **352**: i555.
7. Stirnemann J, Villar J, Salomon LJ, et al. International estimated fetal weight standards of the INTERGROWTH-21(st) Project. *Ultrasound Obstet Gynecol* 2017; **49**(4): 478-86.
8. Papageorghiou AT, Ohuma EO, Altman DG, et al. International standards for fetal growth based on serial ultrasound measurements: the Fetal Growth Longitudinal Study of the INTERGROWTH-21st Project. *Lancet* 2014; **384**(9946): 869-79.
9. Saidi Q, Minja D, Njau J, Hansson H, Kavishe R, Alifrangis M. Ultrasensitive qPCR-Based Detection of Plasmodium falciparum in Pregnant Women Using Dried Blood or Whole Blood Pellet Samples Processed through Different DNA Extraction Methods. *The American journal of tropical medicine and hygiene* 2021; **106**(3): 846-9.
10. Hofmann N, Mwingira F, Shekalaghe S, Robinson LJ, Mueller I, Felger I. Ultra-sensitive detection of Plasmodium falciparum by amplification of multi-copy subtelomeric targets. *PLoS Med* 2015; **12**(3): e1001788.
11. Nag S, Ursing J, Rodrigues A, et al. Proof of concept: used malaria rapid diagnostic tests applied for parallel sequencing for surveillance of molecular markers of anti-malarial resistance in Bissau, Guinea-Bissau during 2014-2017. *Malaria journal* 2019; **18**(1): 252.
12. Desai M, Gutman J, L'Lanziva A, et al. Intermittent screening and treatment or intermittent preventive treatment with dihydroartemisinin-piperaquine versus intermittent preventive treatment with sulfadoxine-pyrimethamine for the control of malaria during pregnancy in western Kenya: an open-label, three-group, randomised controlled superiority trial. *Lancet* 2015; **386**(10012): 2507-19.
13. Madanitsa M, Kalilani L, Mwapasa V, et al. Scheduled Intermittent Screening with Rapid Diagnostic Tests and Treatment with Dihydroartemisinin-Piperaquine versus Intermittent Preventive Therapy with Sulfadoxine-Pyrimethamine for Malaria in Pregnancy in Malawi: An Open-Label Randomized Controlled Trial. *PLoS Med* 2016; **13**(9): e1002124.
14. Schmiegelow C, Minja D, Oesterholt M, et al. Malaria and fetal growth alterations in the 3(rd) trimester of pregnancy: a longitudinal ultrasound study. *PloS one* 2013; **8**(1): e53794.
15. Dossing A, Tarp S, Furst DE, et al. Interpreting trial results following use of different intention-to-treat approaches for preventing attrition bias: a meta-epidemiological study protocol. *BMJ Open* 2014; **4**(9): e005297.

Supplemental tables

Table S1: Sulfadoxine-pyrimethamine (SP) resistance markers at enrolment

**Number of mutations *Pfdhfr+Pfdhps***	***Pfdhfr* 51-59-108-164**	***Pfdhps* 431-436-437-540-581-613**	**Kenya (n=37)**	**Malawi (n=40)**	**Tanzania (n=50)**	**Overall (n=127)**
3+1	**I**-**R**-**N**-I	I-S-A-**E**-A-A	0	0	1 (2.0%)	
2+2	**I**-C-**N**-I	I-S-**G**-**E**-A-A	1 (2.7%)	0	1 (2.0%)	
2+3	N-**R**-**N**-I	I-S-**G**-**E**-**G**-A	0	0	1 (2.0%)	
3+2	**I**-**R**-**N**-I	I-S-**G**-K-A-**S**	0	1 (2.5%)	1 (2.0%)	
Quintuple*	**I**-**R**-**N**-I	I-S-**G**-**E**-A-A	24 (64.9%)	36 (90%)	26 (52.0%)	86 (67.8%)
Sextuple†	**I**-**R**-**N**-I	I-S-**G**-**E**-**G**-A	4 (10.8%)	3 (7.5%)	20 (40.0%)	27 (21.3%)
3+3‡	**I**-**R**-**N**-I	I-**H**-**G**-**E**-A-A	8 (21.6%)			

*Pf*dhfr=*Plasmodium falciparum* dihydrofolate reductase. *Pf*dhps= *Plasmodium falciparum* dihydropteroate synthase
* Quintuple mutant haplotype containing the *Pfdhps*-540E mutation associated with high-grade SP resistance. † Sextuple mutant haplotype containing the *Pfdhps*-540E and the *Pfdhps*-581G mutation associated with very high-grade SP resistance. ‡ Sextuple mutant containing the *Pfdhps*-540E and the new *Pfdhps*-436H mutation, but not the 581G mutation. The clinical significance of this sextuple mutant haplotype containing the *Pfdhps*-436H is not yet known.

TABLE S2: BASELINE CHARACTERISTICS BY STUDY ARM AND COUNTRY

	Kenya			Malawi			Tanzania		
	SP (n=495)	DP (n=497)	DP+AZ (n=498)	SP (n=469)	DP (n=469)	DP+AZ (n=466)	SP (n=597)	DP (n=595)	DP+AZ (n=594)
**Maternal characteristics**									
Maternal age (years)	23.8 (5.36)	23.8 (5.34)	24.0 (5.19)	24.2 (5.77)	24.7 (5.90)	24.2 (5.54)	26.5 (6.58)	26.5 (6.47)	26.3 (6.63)
Residence: Rural vs Semi-urban/Urban	81.6% (404/495)	83.1% (413/497)	79.9% (398/498)	80.8% (379/469)	76.7% (359/468)	79.2% (369/466)	62.1% (371/597)	60.8% (362/595)	62.7% (372/593)
Marital status: Single* vs Married/Cohabiting	23.8% (118/495)	21.7% (108/497)	23.5% (117/498)	7.5% (35/469)	5.6% (26/468)	4.7% (22/466)	9.2% (55/597)	8.4% (50/595)	6.9% (41/593)
Used bednet last night	89.9% (445/495)	91.5% (455/497)	91.8% (457/498)	62.5% (293/469)	65.0% (304/468)	63.7% (297/466)	78.1% (466/597)	82.5% (491/595)	76.2% (452/593)
Attended school	99.6% (493/495)	100.0% (497/497)	99.8% (497/498)	96.4% (452/469)	94.0% (441/469)	94.4% (439/465)	89.4% (533/596)	89.9% (533/593)	89.0% (525/590)
Schooling level									
None	5.7% (28/495)	5.8% (29/497)	5.6% (28/498)	9.0% (42/469)	10.2% (48/469)	9.2% (43/465)	10.9% (65/596)	10.6% (63/593)	11.2% (66/590)
Primary school	44.0% (218/495)	47.5% (236/497)	45.2% (225/498)	65.2% (306/469)	59.7% (280/469)	62.4% (290/465)	61.4% (366/596)	56.7% (336/593)	60.2% (355/590)
Secondary school	34.3% (170/495)	33.0% (164/497)	36.3% (181/498)	22.8% (107/469)	27.5% (129/469)	26.2% (122/465)	24.7% (147/596)	30.2% (179/593)	27.1% (160/590)
Higher	16.0% (79/495)	13.7% (68/497)	12.9% (64/498)	3.0% (14/469)	2.6% (12/469)	2.2% (10/465)	3.0% (18/596)	2.5% (15/593)	1.5% (9/590)
SES index score (terciles)									
Low	3.4% (17/495)	5.4% (27/497)	4.8% (24/498)	71.2% (334/469)	68.2% (320/469)	70.4% (328/466)	29.5% (176/597)	26.2% (156/595)	30.0% (178/594)
Medium	48.7% (241/495)	46.9% (233/497)	46.8% (233/498)	16.2% (76/469)	18.6% (87/469)	17.4% (81/466)	33.3% (199/597)	36.3% (216/595)	32.7% (194/594)
High	47.9% (237/495)	47.7% (237/497)	48.4% (241/498)	12.6% (59/469)	13.2% (62/469)	12.2% (57/466)	37.2% (222/597)	37.5% (223/595)	37.4% (222/594)
Pregnancy number (gravidity)									
First	36.0% (178/495)	35.6% (177/497)	34.3% (171/498)	32.2% (151/469)	29.1% (136/467)	26.1% (121/464)	27.6% (164/594)	27.0% (160/593)	24.2% (143/591)
Second	26.5% (131/495)	27.6% (137/497)	28.3% (141/498)	23.9% (112/469)	27.0% (126/467)	30.2% (140/464)	21.9% (130/594)	21.9% (130/593)	25.0% (148/591)
Third or higher	37.6% (186/495)	36.8% (183/497)	37.3% (186/498)	43.9% (206/469)	43.9% (205/467)	43.8% (203/464)	50.5% (300/594)	51.1% (303/593)	50.8% (300/591)

TABLE S2: BASELINE CHARACTERISTICS BY STUDY ARM AND COUNTRY

	Kenya			Malawi			Tanzania		
	SP (n=495)	DP (n=497)	DP+AZ (n=498)	SP (n=469)	DP (n=469)	DP+AZ (n=466)	SP (n=597)	DP (n=595)	DP+AZ (n=594)
Gestational age (days)	153 (25.9)	149 (24.1)	150 (25.1)	149 (21.5)	149 (21.9)	150 (22.1)	140 (23.4)	141 (24.2)	143 (25.0)
Estimated FW by US (Z-score)†	-0.47 (1.03)	-0.34 (1.25)	-0.51 (1.08)	-1.03 (1.55)	-0.56 (1.62)	-1.02 (1.16)	-0.60 (0.93)	-0.51 (0.92)	-0.57 (0.87)
Weight (kg)	66 (11.0)	64 (11.1)	65 (12.1)	58 (8.8)	59 (9.6)	59 (8.7)	59 (12.4)	59 (11.8)	59 (10.9)
Height (cm)	163 (6.7)	162 (6.9)	163 (7.2)	157 (6.1)	157 (6.6)	158 (7.3)	156 (6.0)	156 (6.3)	155 (5.8)
MUAC (cm)	27.2 (3.23)	26.7 (3.17)	27.0 (3.55)	26.1 (3.09)	26.4 (3.15)	26.3 (2.88)	27.5 (4.00)	27.5 (3.85)	27.4 (3.68)
**Laboratory findings**									
Haemoglobin (g/dL)	10.9 (1.50)	10.8 (1.58)	10.9 (1.38)	11.1 (1.48)	11.3 (1.37)	11.2 (1.42)	11.1 (1.37)	11.0 (1.43)	11.0 (1.47)
Malarial infection									
mRDT	24.4% (118/484)	22.1% (107/485)	23.7% (117/494)	NA	NA	NA	12.5% (74/594)	12.1% (71/589)	13.7% (80/586)
Microscopy	12.4% (61/490)	13.4% (66/491)	16.0% (79/494)	11.1% (52/467)	11.1% (52/467)	15.3% (71/465)	6.4% (38/597)	5.4% (32/595)	5.7% (34/593)
PCR	14.1% (61/434)	15.3% (66/431)	14.3% (62/433)	18.2% (79/433)	18.4% (80/434)	21.1% (91/431)	10.3% (56/543)	10.8% (58/539)	11.9% (65/546)
Any	27.7% (137/495)	25.8% (128/497)	28.5% (142/498)	22.0% (103/469)	23.5% (110/469)	27.7% (129/466)	17.3% (103/597)	17.0% (101/595)	17.5% (104/594)
Sexually transmitted and reproductive tract infections (STI/RTIs)									
Any STI or RTI‡	18.1% (89/492)	17.3% (85/492)	15.6% (77/495)	17.6% (79/450)	15.6% (70/449)	17.3% (77/445)	20.3% (103/508)	16.7% (86/515)	21.6% (110/509)
Any STI	11.8% (58/492)	12.6% (62/491)	10.7% (53/494)	12.1% (54/446)	11.7% (52/445)	11.8% (52/442)	12.0% (71/593)	7.5% (44/590)	10.7% (63/591)
Chlamydia trachomatis	21.3% (34/160)	20.5% (32/156)	20.9% (33/158)	6.1% (6/99)	8.2% (9/110)	14.7% (15/102)	11.3% (22/194)	9.2% (18/196)	12.2% (24/196)
Neisseria gonorrhoea	2.5% (4/160)	7.1% (11/156)	0.6% (1/158)	4.0% (4/99)	2.7% (3/110)	2.9% (3/102)	1.0% (2/194)	1.0% (2/196)	3.6% (7/196)
Treponema pallidum	2.0% (10/492)	2.0% (10/491)	1.6% (8/493)	5.9% (26/440)	7.7% (34/443)	5.2% (23/441)	2.8% (13/457)	1.1% (5/466)	2.6% (12/461)
Trichomonas vaginalis	10.0% (16/160)	14.1% (22/156)	9.5% (15/158)	20.2% (20/99)	14.5% (16/110)	18.6% (19/102)	19.1% (37/194)	12.8% (25/196)	15.3% (30/196)
Bacterial vaginosis	25.3% (41/162)	28.6% (46/161)	21.9% (35/160)	30.5% (32/105)	25.5% (28/110)	36.7% (40/109)	26.4% (51/193)	30.6% (60/196)	30.2% (58/192)

Values are mean (SD) or percentages (n/N) unless otherwise indicated. DP=dihydroartemisinin-piperaquine. AZ=azithromycin. SP=sulfadoxine-pyrimethamine. SES=socio-economic status. US=ultrasound scan. MUAC=mid-upper arm circumference. mRDT=malaria rapid diagnostic test. PCR=polymerase chain reaction. Any=malaria infection detected by either mRDT,

TABLE S2: BASELINE CHARACTERISTICS BY STUDY ARM AND COUNTRY

	Kenya			Malawi			Tanzania		
	**SP (n=495)**	**DP (n=497)**	**DP+AZ (n=498)**	**SP (n=469)**	**DP (n=469)**	**DP+AZ (n=466)**	**SP (n=597)**	**DP (n=595)**	**DP+AZ (n=594)**

microscopy or PCR. STI=sexually transmitted disease. RTIs=reproductive tract infections. *Single includes single unmarried participants not cohabiting, divorced, separated or widowed participants.†A random sample of one-third of participants was selected for fetal weight estimation at enrolment. Among these, 624 (209 in SP arm, 212 in DP arm, 203 in DPAZ arm) participants with a gestational age in weeks of >=22+0 had fetal weight estimates converted to Z-scores using the INTERGROWTH-21 reference (appendix p 4). ‡ Any STI or RTI includes infections with either *Chlamydia trachomatis*, *Neisseria gonorrhoea*, *Treponema pallidum*, or *Trichomonas vaginalis* and bacetrial vaginosis. Any STI includes infections with either *Chlamydia trachomatis*, *Neisseria gonorrhoea*, *Treponema pallidum*, or *Trichomonas vaginalis*.

TABLE S3: FOLLOW-UP VISITS SCHEDULE (MODIFIED ITT POPULATION)

	Overall (n=4680)	SP (n=1561)	DP (n=1561)	DPAZ (n=1558)
Duration of follow-up until delivery, pregnancy loss, or loss-to-follow-up (in months)				
Median (IQR) (range)	4.3 (3.5-5.0) (105-151)	4.3 (3.5-5.0) (106-152)	4.3 (3.5-5.0) (105-151)	4.2 (3.5-4.9) (105-150)
Possible no. of scheduled visits* adjusted for early delivery†, including enrolment, excluding delivery, no (%)				
1	0 (0.0%)	0 (0.0%)	0 (0.0%)	0 (0.0%)
2	0 (0.0%)	0 (0.0%)	0 (0.0%)	0 (0.0%)
3	682 (14.6%)	235 (15.1%)	214 (13.7%)	233 (15.0%)
4	1436 (30.7%)	486 (31.1%)	473 (30.3%)	477 (30.6%)
5	1947 (41.6%)	623 (39.9%)	682 (43.7%)	642 (41.2%)
6	615 (13.1%)	217 (13.9%)	192 (12.3%)	206 (13.2%)
Total planned scheduled visits	21,215	7,066	7,096	7,053
Total achieved scheduled visits	19,349	6,420	6,528	6,401
% achieved of planned visits	91.2%	90.9%	92.0%	90.8%
Number of IPTp received, no(%)‡				
0	9 (0.2%)	8 (0.5%)	0 (0.0%)	1 (0.1%)
1	208 (4.4%)	78 (5.0%)	54 (3.5%)	76 (4.9%)
2	217 (4.6%)	91 (5.8%)	61 (3.9%)	65 (4.2%)
3	545 (11.6%)	198 (12.7%)	186 (11.9%)	161 (10.3%)
4	1235 (26.4%)	403 (25.8%)	380 (24.3%)	452 (29.0%)
5	1448 (30.9%)	465 (29.8%)	518 (33.2%)	465 (29.8%)
6	941 (20.1%)	291 (18.6%)	336 (21.5%)	314 (20.2%)
7	76 (1.6%)	27 (1.7%)	26 (1.7%)	23 (1.5%)
8	1 (0.0%)	0 (0.0%)	0 (0.0%)	1 (0.1%)
Total number of IPTp courses	20,643	6,726	7,042	6,875
Number of IPTp courses, median (IQR, range)	5 (4-6, 0-8)	5 (4-6, 0-7)	5 (4-6, 1-7)	5 (4-6, 0-8)
% of women correctly self-administring AZ or placebo-AZ doses on day 2 at home as prescribed (random home visits followig enrolment visit) §	166/170 (97.6%)	NA	79/82 (96.3%)	87/88 (98.9%)
% of women correctly self-administring DP doses on days 2 and 3 at home as prescribed (random home visits) §	802/816 (98.3%)	NA	412/422 (97.6%)	390/394 (99.0%)

* The number of monthly scheduled visits was dependent on the gestational age at enrolment

† Adjusted for early delivery (i.e. excludes all planned antenatal visits that could not have occurred because the pregnancy ended before that scheduled date)

‡ The number of IPTp courses received can be more than the number of planned scheduled visits for women that delivered after 40 weeks of gestation

§ Adherence was assessed in a randomly selected subgroup of women visited at home for unannounced spot checks on days 2 and 3 after the start of treatment.

ITT=intention to treat. IPTp=intermittent preventive treatment in pregnancy. IQR=interquartile range. SP=sulfadoxine-pyrimethamine. DP=dihydroartemisinin-piperaquine. AZ=azithromycin. NA=not applicable (SP is a single dose, single-day treatment, so all tablets were administered under supervision in the clinic).

TABLE S4: PROPORTION OF WOMEN WITH MISSING DATA FOR THE PRIMARY ENDPOINT BY TREATMENT ARM (MODIFIED ITT POPULATION)

	no/No (%) of patients with missing/partial primary endpoint			Risk Ratio (95% CI), p-value		
	SP	DP	DPAZ	DP vs SP	DPAZ vs SP	DPAZ vs DP
Overall	126/1561 (8.1%)	119/1561 (7.6%)	124/1558 (8.0%)	0.98 (0.78, 1.23), p=0.86	1.03 (0.82, 1.29), p=0.81	1.05 (0.83, 1.32), p=0.69
Kenya	33/495 (6.7%)	24/497 (4.8%)	25/498 (5.0%)	0.73 (0.44, 1.21), p=0.22	0.75 (0.45, 1.24), p=0.26	1.03 (0.60, 1.78), p=0.91
Malawi	45/469 (9.6%)	39/469 (8.3%)	41/466 (8.8%)	0.87 (0.59, 1.29), p=0.48	0.95 (0.64, 1.40), p=0.78	1.09 (0.73, 1.63), p=0.68
Tanzania	48/597 (8.0%)	56/595 (9.4%)	58/594 (9.8%)	1.23 (0.87, 1.74), p=0.24	1.26 (0.90, 1.78), p=0.18	1.03 (0.74, 1.42), p=0.87

DP=dihydroartemisinin-piperaquine. AZ=azithromycin. DPAZ= DP=dihydroartemisinin-piperaquine plus azithromycin.

TABLE S5: SECONDARY EFFICACY ENDPOINTS (CONTINUOUS) BY COUNTRY (MODIFIED ITT POPULATION)

	Number of women, mean (SD)				Mean difference*(95% CI), p-value		
	SP	DP	DPAZ		DP vs SP	DPAZ vs SP	DPAZ vs DP
**Birthweight (grams)**							
Overall	1401	1407	1378	Unadjusted	-89 (-125,-53), p<0.0001	-78 (-114,-42), p<0.0001	11 (-25,47), p=0.55
	3121 (500)	3033 (507)	3042 (480)	Adjusted*	-90 (-126, -54), p<0.0001	-78 (-114, -42), p<0.0001	12 (-24, 48), p=0.52
Kenya	450	458	450	Unadjusted	-101 (-165,-38), p=0.0017	-94 (-158,-31), p=0.0037	7 (-56,71), p=0.82
	3236 (493)	3134 (484)	3139 (486)	Adjusted*	-100 (-163, -36), p=0.0020	-93 (-157, -30), p=0.0041	7 (-57, 70), p=0.84
Malawi	412	414	410	Unadjusted	-72 (-136,-8), p=0.029	-70 (-134,-5), p=0.033	2 (-62,66), p=0.95
	3038 (484)	2965 (494)	2966 (457)	Adjusted*	-74 (-137, -10), p=0.023	-67 (-131, -3), p=0.039	7 (-57, 70), p=0.84
Tanzania	539	535	518	Unadjusted	-92 (-152,-33), p=0.0024	-71 (-131,-11), p=0.020	21 (-39,81), p=0.49
	3090 (502)	2999 (524)	3018 (478)	Adjusted*	-94 (-154, -35), p=0.0019	-73 (-133, -13), p=0.017	21 (-39, 81), p=0.49
**Birthweight for gestational age (Z-score)**							
Overall	1371	1388	1352	Unadjusted	-0.18 (-0.26,-0.11), p<0.0001	-0.18 (-0.25,-0.10), p<0.0001	0.00 (-0.07,0.08), p=0.95
	-0.32 (1.02)	-0.50 (1.05)	-0.50 (1.02)	Adjusted*	-0.18 (-0.26, -0.11), p<0.0001	-0.18 (-0.25, -0.10), p<0.0001	0.01 (-0.07, 0.08), p=0.88
Kenya	438	452	441	Unadjusted	-0.19 (-0.32,-0.07), p=0.0023	-0.22 (-0.34,-0.09), p=0.0006	-0.03 (-0.15,0.10), p=0.69
	-0.12 (0.94)	-0.31 (0.94)	-0.34 (0.97)	Adjusted*	-0.19 (-0.31, -0.07), p=0.0027	-0.22 (-0.34, -0.09), p=0.0007	-0.03 (-0.15, 0.10), p=0.67
Malawi	407	407	403	Unadjusted	-0.16 (-0.30,-0.02), p=0.028	-0.14 (-0.28,0.00), p=0.053	0.02 (-0.12,0.16), p=0.80
	-0.35 (1.06)	-0.52 (1.07)	-0.50 (1.04)	Adjusted*	-0.16 (-0.30, -0.02), p=0.028	-0.13 (-0.27, 0.01), p=0.064	0.02 (-0.12, 0.16), p=0.74
Tanzania	526	529	508	Unadjusted	-0.19 (-0.31,-0.06), p=0.0034	-0.17 (-0.30,-0.05), p=0.0073	0.01 (-0.11,0.14), p=0.83
	-0.47 (1.02)	-0.65 (1.10)	-0.64 (1.03)	Adjusted*	-0.19 (-0.31, -0.06), p=0.0030	-0.17 (-0.30, -0.04), p=0.0083	0.02 (-0.11, 0.15), p=0.77
**Gestational age at birth (weeks)**							
Overall	1395	1417	1395	Unadjusted	-0.1 (-0.3,0.0), p=0.18	-0.1 (-0.2,0.1), p=0.49	0.1 (-0.1,0.2), p=0.51
	39.5 (2.0)	39.4 (2.1)	39.5 (2.0)	Adjusted*	-0.1 (-0.3, 0.0), p=0.17	-0.1 (-0.2, 0.1), p=0.48	0.1 (-0.1, 0.2), p=0.51
Kenya	445	464	457	Unadjusted	-0.2 (-0.4,0.1), p=0.22	-0.0 (-0.3,0.2), p=0.89	0.1 (-0.1,0.4), p=0.28
	39.7 (1.8)	39.5 (2.0)	39.7 (2.0)	Adjusted*	-0.1 (-0.4, 0.1), p=0.28	0.0 (-0.3, 0.3), p=0.99	0.1 (-0.1, 0.4), p=0.27
Malawi	416	421	416	Unadjusted	-0.1 (-0.4,0.2), p=0.52	-0.1 (-0.4,0.2), p=0.56	0.0 (-0.3,0.3), p=0.96
	39.1 (2.1)	39.0 (2.2)	39.0 (2.2)	Adjusted*	-0.1 (-0.4, 0.2), p=0.45	-0.1 (-0.4, 0.2), p=0.59	0.0 (-0.3, 0.3), p=0.83
Tanzania	534	532	522	Unadjusted	-0.1 (-0.3,0.2), p=0.62	-0.1 (-0.3,0.2), p=0.64	0.0 (-0.2,0.2), p=0.98
	39.7 (2.0)	39.6 (2.0)	39.6 (1.9)	Adjusted*	-0.1 (-0.3, 0.2), p=0.59	-0.1 (-0.3, 0.2), p=0.55	-0.0 (-0.2, 0.2), p=0.94

	Number of women, mean (SD)				Mean difference*(95% CI), p-value		
	SP	DP	DPAZ		DP vs SP	DPAZ vs SP	DPAZ vs DP
**Neonatal length (cm)**							
Overall	1327	1309	1270	Unadjusted	-0.2 (-0.4,0.0), p=0.11	-0.2 (-0.4,0.0), p=0.062	-0.0 (-0.3,0.2), p=0.76
	48.6 (3.2)	48.4 (3.1)	48.4 (3.2)	Adjusted*	-0.2 (-0.4, 0.0), p=0.094	-0.2 (-0.4, 0.0), p=0.054	-0.0 (-0.2, 0.2), p=0.79
Kenya	423	422	408	Unadjusted	-0.2 (-0.5,0.1), p=0.12	-0.2 (-0.5,0.1), p=0.12	-0.0 (-0.3,0.3), p=0.98
	49.2 (2.4)	49.0 (2.0)	49.0 (2.3)	Adjusted*	-0.2 (-0.5, 0.1), p=0.13	-0.2 (-0.5, 0.1), p=0.12	-0.0 (-0.3, 0.3), p=0.97
Malawi	406	410	400	Unadjusted	0.1 (-0.4,0.6), p=0.71	-0.1 (-0.6,0.4), p=0.59	-0.2 (-0.7,0.3), p=0.36
	47.4 (3.7)	47.5 (3.7)	47.3 (3.6)	Adjusted*	0.1 (-0.4, 0.5), p=0.81	-0.1 (-0.6, 0.3), p=0.58	-0.2 (-0.7, 0.3), p=0.43
Tanzania	498	477	462	Unadjusted	-0.3 (-0.7,-0.0), p=0.047	-0.2 (-0.6,0.1), p=0.16	0.1 (-0.3,0.5), p=0.58
	49.1 (3.0)	48.7 (3.3)	48.8 (3.2)	Adjusted*	-0.4 (-0.7, -0.0), p=0.044	-0.2 (-0.6, 0.1), p=0.17	0.1 (-0.2, 0.5), p=0.53
**Maternal MUAC (cm)**							
Overall	1345	1339	1299	Unadjusted	-0.3 (-0.6,-0.1), p=0.0042	-0.3 (-0.5,-0.1), p=0.016	0.0 (-0.2,0.3), p=0.68
	26.6 (3.3)	26.3 (3.1)	26.3 (3.2)	Adjusted*	-0.4 (-0.6, -0.1), p=0.0020	-0.3 (-0.5, -0.0), p=0.018	0.1 (-0.1, 0.3), p=0.48
Kenya	427	437	424	Unadjusted	-0.6 (-1.0,-0.2), p=0.0070	-0.3 (-0.7,0.1), p=0.18	0.3 (-0.1,0.7), p=0.18
	27.0 (3.1)	26.5 (3.0)	26.7 (3.5)	Adjusted*	-0.6 (-1.0, -0.1), p=0.0076	-0.3 (-0.7, 0.1), p=0.17	0.3 (-0.1, 0.7), p=0.20
Malawi	413	417	408	Unadjusted	0.0 (-0.3,0.4), p=0.95	0.1 (-0.3,0.4), p=0.66	0.1 (-0.3,0.4), p=0.70
	25.7 (2.8)	25.7 (2.7)	25.8 (2.7)	Adjusted*	-0.0 (-0.4, 0.3), p=0.94	0.1 (-0.2, 0.5), p=0.52	0.1 (-0.2, 0.5), p=0.47
Tanzania	505	485	467	Unadjusted	-0.4 (-0.8,-0.0), p=0.047	-0.6 (-1.0,-0.2), p=0.0053	-0.2 (-0.6,0.3), p=0.42
	27.0 (3.8)	26.7 (3.4)	26.5 (3.3)	Adjusted*	-0.5 (-0.9, -0.1), p=0.023	-0.6 (-1.0, -0.2), p=0.0038	-0.1 (-0.6, 0.3), p=0.53
**Estimated fetal weight by US 32-35 weeks (Z-score) ‡**							
Overall	369, 479	407, 482	407, 479	Unadjusted	-0.16 (-0.27, -0.05), p=0.0037	-0.18 (-0.29, -0.07), p=0.0012	-0.02 (-0.13, 0.09), p=0.74
	0.19 (0.84)	-0.01 (0.92)	0.05 (0.95)	Adjusted*	-0.16 (-0.26, -0.05), p=0.0055	-0.17 (-0.28, -0.06), p=0.0022	-0.02 (-0.13, 0.09), p=0.77
Kenya	147, 156	153, 160	153, 162	Unadjusted	0.02 (-0.19, 0.22), p=0.88	-0.01 (-0.21, 0.19), p=0.91	-0.03 (-0.23, 0.17), p=0.79
	0.21 (0.91)	0.17 (0.94)	0.21 (0.97)	Adjusted*	0.01 (-0.19, 0.22), p=0.90	-0.01 (-0.21, 0.19), p=0.92	-0.02 (-0.22, 0.18), p=0.82
Malawi	111, 130	118, 135	123, 133	Unadjusted	-0.27 (-0.50, -0.04), p=0.021	-0.36 (-0.59, -0.14), p=0.0018	-0.09 (-0.32, 0.13), p=0.41
	-0.02 (0.92)	-0.37 (1.02)	-0.35 (1.03)	Adjusted*	-0.23 (-0.46, -0.01), p=0.045	-0.34 (-0.56, -0.11), p=0.0038	-0.10 (-0.33, 0.12), p=0.37
Tanzania	186, 193	181, 187	175, 184	Unadjusted	-0.23 (-0.39, -0.07; p=0.0059	-0.24 (-0.40, 0.08; p=0.0039	-0.01 (-0.18, 0.15; p=0.88
	0.26 (0.80)	0.04 (0.75)	0.17 (0.92)	Adjusted*	-0.22 (-0.39, -0.06), p=0.0065	-0.23 (-0.39, -0.07), p=0.0051	-0.01 (-0.17, 0.16), p=0.92
**Gestational weight gain per week (kg) †**							
Overall	1443	1454	1434	Unadjusted	-0.04 (-0.06,-0.03), p<0.0001	-0.04 (-0.05,-0.02), p<0.0001	0.00 (-0.01,0.02), p=0.67

	Number of women, mean (SD)				Mean difference*(95% CI), p-value		
	SP	DP	DPAZ		DP vs SP	DPAZ vs SP	DPAZ vs DP
	0.31 (0.20)	0.27 (0.21)	0.27 (0.20)	Adjusted*	-0.04 (-0.06, -0.03), p<0.0001	-0.04 (-0.05, -0.02), p<0.0001	0.00 (-0.01, 0.02), p=0.52
Kenya	461	466	465	Unadjusted	-0.04 (-0.06,-0.01), p=0.0021	-0.03 (-0.05,-0.00), p=0.037	0.01 (-0.01,0.04), p=0.32
	0.32 (0.20)	0.28 (0.20)	0.30 (0.18)	Adjusted*	-0.04 (-0.07, -0.02), p=0.0008	-0.03 (-0.05, -0.00), p=0.026	0.01 (-0.01, 0.04), p=0.26
Malawi	429	433	428	Unadjusted	-0.03 (-0.06,-0.00), p=0.035	-0.03 (-0.06,-0.00), p=0.040	0.00 (-0.03,0.03), p=0.96
	0.30 (0.22)	0.26 (0.23)	0.26 (0.23)	Adjusted*	-0.03 (-0.06, -0.00), p=0.038	-0.03 (-0.06, 0.00), p=0.056	0.00 (-0.03, 0.03), p=0.88
Tanzania	553	555	541	Unadjusted	-0.05 (-0.07,-0.03), p<0.0001	-0.05 (-0.08,-0.03), p<0.0001	-0.00 (-0.02,0.02), p=0.82
	0.31 (0.19)	0.26 (0.20)	0.25 (0.18)	Adjusted*	-0.05 (-0.07, -0.03), p<0.0001	-0.05 (-0.07, -0.03), p<0.0001	-0.00 (-0.02, 0.02), p=0.98
**Maternal Hb third trimester (g/dL)**							
Overall	1375	1390	1350	Unadjusted	-0.0 (-0.1,0.1), p=0.87	-0.0 (-0.1,0.1), p=0.62	-0.0 (-0.1,0.1), p=0.74
	10.9 (1.4)	10.9 (1.3)	10.9 (1.3)	Adjusted*	-0.0 (-0.1, 0.1), p=0.88	-0.0 (-0.1, 0.1), p=0.67	-0.0 (-0.1, 0.1), p=0.78
Kenya	433	446	440	Unadjusted	0.0 (-0.2,0.2), p=0.94	0.0 (-0.2,0.2), p=0.71	0.0 (-0.2,0.2), p=0.77
	10.7 (1.7)	10.7 (1.5)	10.7 (1.5)	Adjusted*	0.0 (-0.2, 0.2), p=0.91	0.0 (-0.2, 0.2), p=0.76	0.0 (-0.2, 0.2), p=0.84
Malawi	402	404	392	Unadjusted	0.0 (-0.1,0.2), p=0.88	-0.1 (-0.2,0.1), p=0.38	-0.1 (-0.2,0.1), p=0.31
	11.3 (1.1)	11.3 (1.1)	11.2 (1.1)	Adjusted*	0.0 (-0.1, 0.2), p=0.88	-0.1 (-0.2, 0.1), p=0.39	-0.1 (-0.2, 0.1), p=0.31
Tanzania	540	540	518	Unadjusted	-0.0 (-0.2,0.1), p=0.64	-0.0 (-0.2,0.1), p=0.54	-0.0 (-0.2,0.1), p=0.88
	10.9 (1.3)	10.9 (1.2)	10.8 (1.2)	Adjusted*	-0.0 (-0.2, 0.1), p=0.70	-0.0 (-0.2, 0.1), p=0.63	-0.0 (-0.2, 0.1), p=0.92
**Maternal Hb delivery (g/dL)**							
Overall	431	415	410	Unadjusted	0.1 (-0.2,0.3), p=0.55	0.1 (-0.1,0.4), p=0.30	0.1 (-0.2,0.3), p=0.66
	11.2 (1.8)	11.3 (1.6)	11.3 (1.8)	Adjusted*	0.1 (-0.2, 0.3), p=0.60	0.1 (-0.1, 0.4), p=0.30	0.1 (-0.2, 0.3), p=0.62
Kenya	289	306	293	Unadjusted	0.2 (-0.1,0.5), p=0.23	0.3 (-0.0,0.6), p=0.088	0.1 (-0.2,0.4), p=0.61
	11.1 (1.9)	11.3 (1.7)	11.4 (2.0)	Adjusted*	0.2 (-0.1, 0.5), p=0.24	0.3 (-0.0, 0.5), p=0.094	0.1 (-0.2, 0.4), p=0.61
Malawi	20	18	15	Unadjusted	-0.2 (-1.0,0.7), p=0.66	-0.1 (-1.1,0.8), p=0.77	0.1 (-0.9,1.0), p=0.91
	12.2 (1.5)	11.9 (1.2)	11.8 (1.1)	Adjusted*	-0.1 (-1.1, 0.8), p=0.81	-0.2 (-1.2, 0.8), p=0.69	-0.1 (-1.1, 0.9), p=0.86
Tanzania	122	91	102	Unadjusted	-0.2 (-0.6,0.2), p=0.39	-0.2 (-0.6,0.2), p=0.38	0.0 (-0.4,0.4), p=0.99
	11.3 (1.7)	11.1 (1.3)	11.1 (1.5)	Adjusted*	-0.2 (-0.6, 0.2), p=0.39	-0.2 (-0.6, 0.2), p=0.39	0.0 (-0.4, 0.4), p=0.98
**Cord blood Hb (g/dL)**							
Overall	1182	1185	1130	Unadjusted	-0.1 (-0.2,0.1), p=0.41	0.1 (-0.0,0.3), p=0.12	0.2 (0.0,0.4), p=0.017
	14.4 (2.0)	14.3 (2.1)	14.5 (2.0)	Adjusted*	-0.1 (-0.2, 0.1), p=0.36	0.1 (-0.0, 0.3), p=0.10	0.2 (0.0, 0.4), p=0.012
Kenya	398	406	380	Unadjusted	-0.2 (-0.5,0.1), p=0.18	-0.0 (-0.3,0.3), p=0.81	0.2 (-0.1,0.5), p=0.28

	Number of women, mean (SD)				Mean difference*(95% CI), p-value		
	**SP**	**DP**	**DPAZ**		**DP vs SP**	**DPAZ vs SP**	**DPAZ vs DP**
	14.4 (2.0)	14.2 (2.2)	14.3 (2.2)	Adjusted*	-0.2 (-0.5, 0.1), p=0.17	-0.0 (-0.3, 0.3), p=0.81	0.2 (-0.1, 0.5), p=0.27
Malawi	339	364	337	Unadjusted	0.1 (-0.2,0.4), p=0.53	0.3 (0.0,0.6), p=0.048	0.2 (-0.1,0.5), p=0.17
	14.3 (2.1)	14.3 (2.4)	14.6 (2.2)	Adjusted*	0.1 (-0.2, 0.4), p=0.60	0.3 (0.0, 0.6), p=0.042	0.2 (-0.1, 0.6), p=0.12
Tanzania	445	415	413	Unadjusted	-0.1 (-0.3,0.2), p=0.52	0.1 (-0.1,0.4), p=0.27	0.2 (-0.0,0.5), p=0.086
	14.5 (1.8)	14.5 (1.8)	14.7 (1.8)	Adjusted*	-0.1 (-0.3, 0.2), p=0.50	0.1 (-0.1, 0.4), p=0.24	0.2 (-0.0, 0.5), p=0.069

The crude models include the stratification factors site and gravidity (paucigravidae and multigravidae) as covariates. *The adjusted models include six additional prespecified covariates: patent malaria status at enrolment (positive/negative), gestational age in days at enrolment (continuous), socioeconomic status (socioeconomic index calculated using principal component analysis) (continuous), season (based on average ranked rainfall during the last 6 months of pregnancy) (continuous), malaria transmission intensity by site (based on the prevalence of malaria at enrolment) (continuous)and the degree of SP resistance by site (very high vs high based on the prevalence of the dhps-A581G mutation >40% vs <= 40%). † Post-hoc. P-values and the widths of the confidence intervals for the secondary endpoints have not been adjusted for multiplicity, so the values should not be used to infer definitive treatment effects. ITT=Intention to treat. SD=standard deviation. DP=dihydroartemisinin-piperaquine. AZ=azithromycin. SP=sulfadoxine-pyrimethamine. Cm=centimetre. US=ultrasound scan. Kg=kilogram. Hb=haemoglobin. g/dL=grams per decilitre. ‡ Estimated fetal weight obtained by ultrasound scan. The mean Z-scores for estimated fetal weight represent the scores measured around 32-35 weeks gestation. The mean difference represents the overall difference obtained by linear mixed models for repeated measures that also include the measures taken around 25-28 weeks gestation. The first N represent the number of participants contributing to the mean (SD) at 32-25 weeks. The second N represents the number of participants contributing to the mean difference estimates and includes participants contributing to either the 25-28 weeks measure or the 32-25 weeks measure or both. If no ultrasound scan was available during each time window, the closest ultrasound scan taken was used. The mean differences for estimated fetal weight Z-scores were obtained by linear mixed models for repeated measures in a random sample of one-third of the participants. All other effect estimates were obtained by standard linear regression. The unadjusted linear mixed models contained visit, study arm, the interaction between visit and study arm, and the stratification factors site and gravidity (paucigravidae and multigravidae) as fixed effect and participant as random effects to account for the clustering within subject. The adjusted linear mixed models also included the six additional prespecified covariates listed above (under *) as fixed effect, and participant as random effects.

Table S6: Drug tolerability in a sub-group of 10% women visited at home within the first three days after the start of an IPTp course

	Number of women, n/N(%)			Crude RR (95% CI), p-value		
	**SP**	**DP**	**DP+AZ**	**SP v DP**	**SP v DP+AZ**	**DP v DP+AZ**
**Vomiting**						
Any	1/384 (0.3%)	13/410 (3.2%)	13/371 (3.5%)	12.49 (1.66, 93.99), 0.014	12.39 (1.65, 93.19), 0.014	0.99 (0.48, 2.04), 0.98
Month 1	0/384 (0.0%)	3/410 (0.7%)	8/371 (2.2%)	0.25	0.0033	0.13
Month 2	0/374 (0.0%)	5/403 (1.2%)	4/364 (1.1%)	0.062	0.058	1
Month 3	0/372 (0.0%)	3/389 (0.7%)	1/347 (0.3%)	0.25	0.49	0.63
Month 4	1/345 (0.3%)	3/368 (0.7%)	3/336 (0.8%)	0.62	0.37	1
Month 5	0/255 (0.0%)	1/292 (0.2%)	0/243 (0.0%)	1	NA	1
Month 6	0/127 (0.0%)	1/142 (0.2%)	1/110 (0.3%)	1	0.49	1
Month 7	0/12 (0.0%)	0/11 (0.0%)	0/10 (0.0%)	NA	NA	NA
**Nausea**						
Any	1/384 (0.3%)	14/410 (3.4%)	8/371 (2.2%)	13.43 (1.81, 99.46), 0.011	7.33 (0.94, 57.06), 0.057	0.55 (0.25, 1.21), 0.14
Month 1	1/384 (0.8%)	3/410 (2.1%)	3/371 (2.7%)	0.62	0.37	1
Month 2	0/374 (0.0%)	6/403 (4.2%)	1/364 (0.9%)	0.031	0.49	0.13
Month 3	0/372 (0.0%)	3/389 (2.1%)	2/347 (1.8%)	0.25	0.24	1
Month 4	0/345 (0.0%)	3/368 (2.1%)	1/336 (0.9%)	0.25	0.49	0.63
Month 5	0/255 (0.0%)	0/292 (0.0%)	1/243 (0.9%)	NA	0.49	0.48
Month 6	0/127 (0.0%)	0/142 (0.0%)	1/110 (0.9%)	NA	0.49	0.48
Month 7	0/12 (0.0%)	0/11 (0.0%)	0/10 (0.0%)	NA	NA	NA
**Abdominal pain**						
Any	0/384 (0.0%)	3/410 (0.7%)	5/371 (1.3%)	0.98	0.98	1.71 (0.42, 6.88), 0.45
Month 1	0/384 (0.0%)	0/410 (0.0%)	1/371 (0.4%)	NA	0.49	0.48
Month 2	0/374 (0.0%)	2/403 (0.7%)	3/364 (1.2%)	0.5	0.12	0.67
Month 3	0/372 (0.0%)	1/389 (0.3%)	1/347 (0.4%)	1	0.49	1
Month 4	0/345 (0.0%)	0/368 (0.0%)	0/336 (0.0%)	NA	NA	NA
Month 5	0/255 (0.0%)	0/292 (0.0%)	0/243 (0.0%)	NA	NA	NA
Month 6	0/127 (0.0%)	0/142 (0.0%)	0/110 (0.0%)	NA	NA	NA
Month 7	0/12 (0.0%)	0/11 (0.0%)	0/10 (0.0%)	NA	NA	NA
**Diarrhoea**						
Any	1/384 (0.3%)	1/410 (0.2%)	4/371 (1.1%)	0.88 (0.06, 14.05), 0.93	4.08 (0.46, 35.86), 0.21	4.62 (0.52, 41.00), 0.17
Month 1	1/384 (0.3%)	1/410 (0.3%)	4/371 (1.2%)	1	0.21	0.2
Month 2	0/374 (0.0%)	0/403 (0.0%)	0/364 (0.0%)	NA	NA	NA
Month 3	0/372 (0.0%)	0/389 (0.0%)	0/347 (0.0%)	NA	NA	NA
Month 4	0/345 (0.0%)	0/368 (0.0%)	0/336 (0.0%)	NA	NA	NA
Month 5	0/255 (0.0%)	0/292 (0.0%)	0/243 (0.0%)	NA	NA	NA
Month 6	0/127 (0.0%)	0/142 (0.0%)	0/110 (0.0%)	NA	NA	NA
Month 7	0/12 (0.0%)	0/11 (0.0%)	0/10 (0.0%)	NA	NA	NA
**Other gastrointestinal complaints**						
Any	1/384 (0.3%)	4/410 (1.0%)	9/371 (2.4%)	3.59 (0.41, 31.70), 0.25	8.66 (1.12, 67.16), 0.039	2.41 (0.76, 7.61), 0.13
Month 1	1/384 (0.3%)	1/410 (0.3%)	5/371 (1.4%)	1	0.12	0.11
Month 2	0/374 (0.0%)	2/403 (0.5%)	3/364 (0.9%)	0.5	0.12	0.67
Month 3	0/372 (0.0%)	1/389 (0.3%)	1/347 (0.3%)	1	0.49	1

Month 4	0/345 (0.0%)	0/368 (0.0%)	0/336 (0.0%)	NA	NA	NA
Month 5	0/255 (0.0%)	0/292 (0.0%)	0/243 (0.0%)	NA	NA	NA
Month 6	0/127 (0.0%)	0/142 (0.0%)	0/110 (0.0%)	NA	NA	NA
Month 7	0/12 (0.0%)	0/11 (0.0%)	0/10 (0.0%)	NA	NA	NA
**Dizziness**						
Any	0/384 (0.0%)	15/410 (3.7%)	7/371 (1.9%)	0.98	0.98	0.46 (0.20, 1.05), 0.064
Month 1	0/384 (0.0%)	2/410 (0.5%)	2/371 (0.5%)	0.5	0.24	1
Month 2	0/374 (0.0%)	5/403 (1.2%)	2/364 (0.5%)	0.062	0.24	0.46
Month 3	0/372 (0.0%)	4/389 (1.0%)	0/347 (0.0%)	0.12	NA	0.13
Month 4	0/345 (0.0%)	4/368 (1.0%)	1/336 (0.3%)	0.12	0.49	0.38
Month 5	0/255 (0.0%)	1/292 (0.2%)	2/243 (0.5%)	1	0.24	0.61
Month 6	0/127 (0.0%)	1/142 (0.2%)	0/110 (0.0%)	1	NA	1
Month 7	0/12 (0.0%)	0/11 (0.0%)	0/10 (0.0%)	NA	NA	NA

RR=Risk ratio. CI=confidence interval. NA=Not available because of zero values,
Tolerability was assessed by assessing adverse events during random home visits in a pre-selected random sample 10% of participants.
DP=dihydroartemisinin-piperaquine. AZ=azithromycin. SP=sulfadoxine-pyrimethamine.

TABLE S7: ECG MONITORING QTC

Dose	Day and Time	N		Measure	DP (n=98)	DPAZ (n=91)	MD (95% CI) or RR (95% CI)	p-value
First	Day-0 (baseline)	189	QTc	Mean (SD)	418 (17.9)	416 (17.6)	-1.2 (-6.0, 3.6)	0.62
				Range	371-465	363-463		
				>480ms, n (%)	0/98 (0%)	0/91 (0%)	-	-
				>500ms, n (%)	0/98 (0%)	0/91 (0%)	-	-
	Day-2 + 4hr	181		Mean (SD)	445 (27.0)	452 (25.0)	7.3 (-0.4, 14.9)	0.062
				Range	379-567	400-532		
				>480ms, n (%)	10/97 (10%)	9/84 (11%)	0.97 (0.42, 2.26)	0.94
				>500ms, n (%)	2/97 (2%)	3/84 (4%)	1.69 (0.29, 9.76)	0.56
	from Day-0 first course		dQTc	Mean (SD)	27 (21.0)	36 (25.9)	8.8 (2.0, 15.7)	0.011
				>60ms, n (%)	5/97 (5%)	10/84 (12%)	2.21 (0.80, 6.16)	0.13
Middle	Day-0	159	QTc	Mean (SD)	415 (22.1)	411 (19.3)	-3.1 (-8.5, 2.4)	0.27
				Range	362-459	358-461		
				>480ms, n (%)	0/82 (0%)	0/77 (0%)	-	-
				>500ms, n (%)	0/82 (0%)	0/77 (0%)	-	-
	Day-2 + 4hr	155		Mean (SD)	435 (23.2)	439 (22.7)	3.8 (-2.9, 10.5)	0.27
				Range	381-490	383-494		
				>480ms, n (%)	4/80 (5%)	5/75 (7%)	1.38 (0.40, 4.72)	0.60
				>500ms, n (%)	0/80 (0%)	0/75 (0%)	-	-
	from Day-0 middle course		dQTc	Mean (SD)	20 (18.0)	28 (16.8)	6.1 (0.8, 11.4)	0.024
				>60ms, n (%)	1/80 (1%)	2/75 (3%)	2.00 (0.20, 19.78)	0.55
	from Day-0 first course		dQTc	Mean (SD)	16 (19.2)	22 (20.4)	4.7 (-1.4, 10.8)	0.13
				>60ms, n (%)	1/80 (1%)	4/75 (5%)	3.84 (0.44, 33.16)	0.22
Last	Day-0	147	QTc	Mean (SD)	415 (22.7)	416 (24.1)	1.0 (-5.9, 7.9)	0.77
				Range	303-462	361-511		
				>480ms, n (%)	0/81 (0%)	1/66 (2%)	-	-
				>500ms, n (%)	0/81 (0%)	1/66 (2%)	-	-
	Day-2 + 4hr	139		Mean (SD)	428 (21.7)	424 (55.6)	-6.0 (-19.4, 7.4)	0.38
				Range	379-505	21-473		
				>480ms, n (%)	1/77 (1%)	0/63 (0%)	-	-
				>500ms, n (%)	1/77 (1%)	0/63 (0%)	-	-
	from Day-0 last course		dQTc	Mean (SD)	13 (24.0)	6 (55.9)	-8.3 (-22.1, 5.6)	0.24
				>60ms, n (%)	1/77 (1%)	0/61 (0%)	-	-
	from Day-0 first course		dQTc	Mean (SD)	9 (20.1)	6 (54.4)	-4.6 (-17.7, 8.5)	0.49
				>60ms, n (%)	1/77 (1%)	1/63 (2%)	1.31 (0.09, 19.53)	0.84
All visits	Day-0	495	QTc	Mean (SD)	416 (20.8)	415 (20.2)	-1.1 (-4.3, 2.2)	0.52
				Range	303-465	358-511		
				>480ms, n (%)	0/99 (0%)	1/93 (1%)	-	-
				>500ms, n (%)	0/99 (0%)	1/93 (1%)	-	-
	Day-2 + 4hr	475		Mean (SD)	437 (25.2)	440 (37.5)	2.6 (-3.0, 8.2)	0.37
				Range	379-567	21-532		
				>480ms, n (%)	11/98 (11%)	13/89 (15%)	1.23 (0.59, 2.57)	0.59
				>500ms, n (%)	2/98 (2%)	3/89 (3%)	1.65 (0.29, 9.54)	0.57
	from Day-0 current course		dQTc	Mean (SD)	21 (21.7)	25 (36.8)	3.5 (-1.8, 8.8)	0.20
				>60ms, n (%)	7/98 (7%)	12/89 (13%)	1.83 (0.77, 4.31)	0.17
	from Day-0 first course		dQTc	Mean (SD)	18 (21.4)	23 (37.0)	3.9 (-1.4, 9.2)	0.15
Difference in dQTc between courses	Middle v first course paired t-test	149	Diff dQTc	Mean (SD)	-7 (26.4) p=0.031	-3 (25.7) p=0.32		
	Last v first course paired t-test	132	Diff dQTc	Mean (SD)	-12 (27.5) p=0.0002	-25 (63.1) p=0.0042		

ECG=electrocardiogram. QTc: QT interval corrected for heart rate using study-derived correction factor (see appendix p-3). DP=dihydroartemisinin-piperaquine. AZ=azithromycin. MD=mean difference. RR=Risk ratio. SD=standard deviation. Ms=milliseconds. MD=mean difference. CI=confidence interval.

Table S8: Sensitivity analysis of the treatment effect on estimated foetal weight (EFW) using multiple imputations for missing baseline EFW values (post-hoc)

**Model description**	**Treatment effect (mean diff (95% CI))**		
	**DP v SP**	**DP+AZ v SP**	**DP+AZ v DP**
**Linear mixed model (no enrolment EFW as covariate)**	-0.16 (-0.27, -0.05)	-0.17 (-0.28, -0.06)	-0.02 (-0.13, 0.09)
**Linear mixed model with imputed values for enrolment EFW**	-0.16 (-0.27, -0.05)	-0.17 (-0.28, -0.06)	-0.01 (-0.12, 0.10)

Models showing the results of linear mixed models of the treatment effect on estimated fetal weight Z-score among 1,560 participants randomised to be included in the nested study involving serial ultrasound scan to assess fetal growth. The first row represents models excluding baseline Z-scores for EFW at enrolment as a covariate. The second row presents models that include enrolment Z-scores for EFW following multiple imputations of missing baseline EFW observation in 60% of the 1,560 participants because they were recruited before 22 weeks gestation. The international estimated fetal weight standards of the INTERGROWTH-21st Project only start at 22 weeks (Stirnemann J, Villar J, Salomon LJ, et al. International estimated fetal weight standards of the INTERGROWTH-21(st) Project. Ultrasound Obstet Gynecol 2017; 49(4): 478-86).

Supplemental figures

Figure S1: The efficacy of IPTp-DP and IPTp-DPAZ versus IPTp-SP on the composite primary endpoint and its components (per-protocol population)

Subgroup	Arm 1, n/N(%)	Arm 2, n/N(%)	Crude RR (95% CI)	P-value	Adjusted RR (95% CI)	P-value
DP v SP						
Adverse pregnancy outcome (primary outcome)	364/1303 (27.9%)	291/1271 (22.9%)	**1.21 (1.06, 1.38)**	**0.0045**	1.21 (1.06, 1.38)	0.0043
Fetal loss	27/1303 (2.1%)	22/1271 (1.7%)	**1.19 (0.68, 2.07)**	**0.55**	1.18 (0.68, 2.07)	0.55
LBW	138/1279 (10.8%)	89/1250 (7.1%)	**1.52 (1.18, 1.96)**	**0.0012**	1.52 (1.18, 1.95)	0.0013
Pre-term	83/1303 (6.4%)	82/1271 (6.5%)	**1.00 (0.74, 1.34)**	**0.98**	0.99 (0.74, 1.33)	0.97
SGA	281/1279 (22.0%)	204/1250 (16.3%)	**1.34 (1.14, 1.57)**	**0.0004**	1.34 (1.14, 1.57)	0.0004
Neonatal death	12/1156 (1.0%)	15/1118 (1.3%)	**0.78 (0.37, 1.65)**	**0.51**	0.77 (0.36, 1.62)	0.49
DP+AZ v SP						
Adverse pregnancy outcome (primary outcome)	356/1272 (28.0%)	291/1271 (22.9%)	**1.19 (1.05, 1.36)**	**0.0088**	1.20 (1.05, 1.37)	0.0073
Fetal loss	31/1272 (2.4%)	22/1271 (1.7%)	**1.38 (0.81, 2.38)**	**0.24**	1.38 (0.81, 2.37)	0.24
LBW	120/1230 (9.8%)	89/1250 (7.1%)	**1.37 (1.05, 1.77)**	**0.019**	1.37 (1.05, 1.77)	0.019
Pre-term	80/1272 (6.3%)	82/1271 (6.5%)	**0.97 (0.72, 1.31)**	**0.86**	0.97 (0.72, 1.31)	0.86
SGA	269/1230 (21.9%)	204/1250 (16.3%)	**1.31 (1.12, 1.54)**	**0.0010**	1.32 (1.12, 1.55)	0.0009
Neonatal death	14/1123 (1.2%)	15/1118 (1.3%)	**0.95 (0.46, 1.96)**	**0.89**	0.97 (0.47, 1.99)	0.92
DP+AZ v DP						
Adverse pregnancy outcome (primary outcome)	356/1272 (28.0%)	364/1303 (27.9%)	**0.99 (0.87, 1.11)**	**0.82**	0.99 (0.88, 1.12)	0.87
Fetal loss	31/1272 (2.4%)	27/1303 (2.1%)	**1.17 (0.70, 1.94)**	**0.55**	1.17 (0.70, 1.94)	0.55
LBW	120/1230 (9.8%)	138/1279 (10.8%)	**0.90 (0.71, 1.13)**	**0.36**	0.90 (0.72, 1.13)	0.38
Pre-term	80/1272 (6.3%)	83/1303 (6.4%)	**0.98 (0.73, 1.32)**	**0.89**	0.98 (0.73, 1.32)	0.89
SGA	269/1230 (21.9%)	281/1279 (22.0%)	**0.98 (0.85, 1.14)**	**0.81**	0.99 (0.85, 1.14)	0.84
Neonatal death	14/1123 (1.2%)	12/1156 (1.0%)	**1.22 (0.57, 2.63)**	**0.61**	1.26 (0.59, 2.71)	0.55

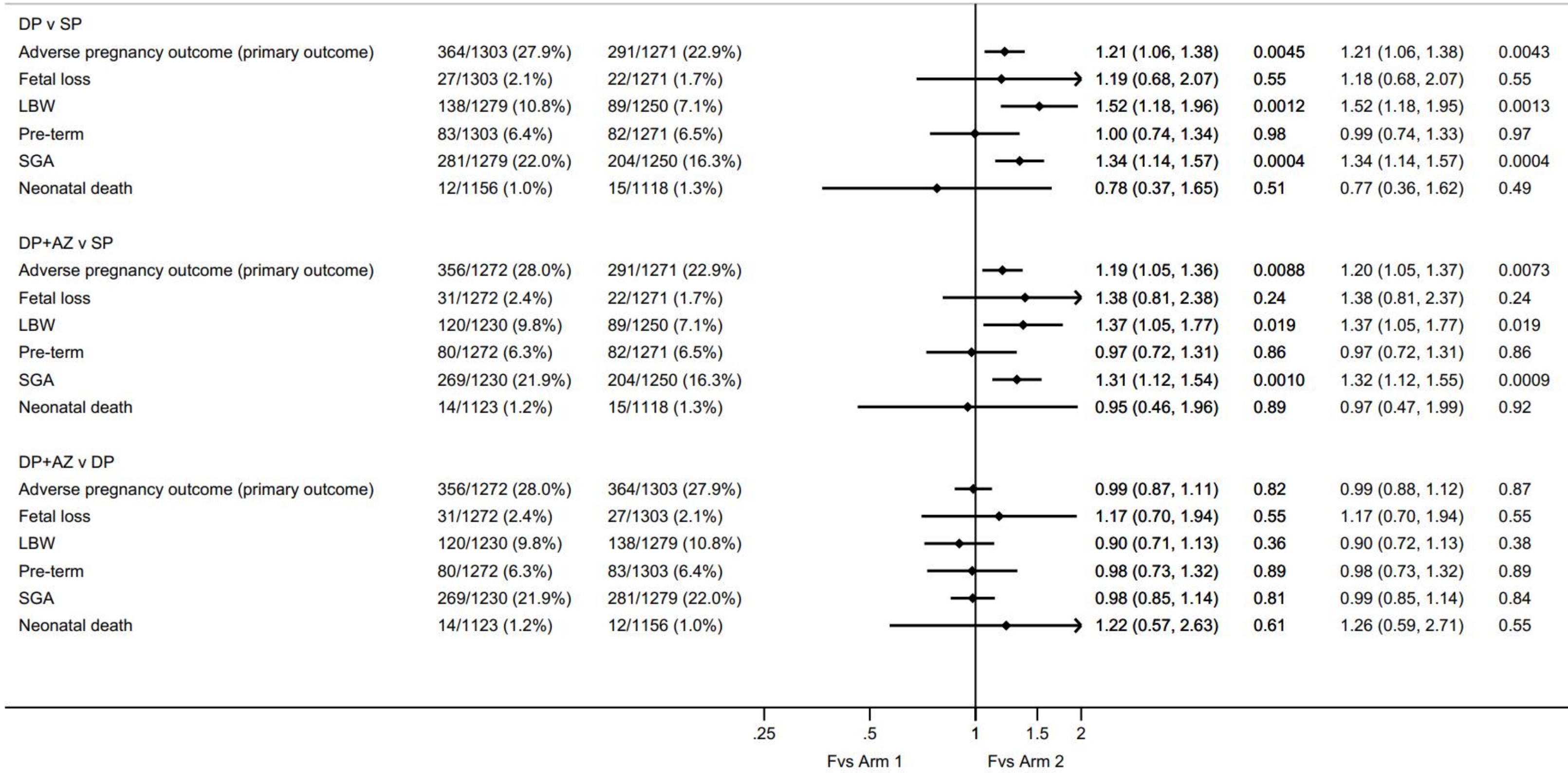

The crude RR values were obtained from log-binomial regressions with the stratification factors site and gravidity (paucigravidae and multigravidae) included as covariates. The adjusted RR values were adjusted for site, gravidity, and six other prespecified covariates. IPTp=intermittent preventive treatment in pregnancy. ITT=intention to treat. RR=risk ratio. CI=confidence interval, DP=dihydroartemisinin-piperaquine. AZ=azithromycin. SP=sulfadoxine-pyrimethamine. LBW=low birthweight. SGA=small for gestational age. Fvs=favours.

Figure S2: Sensitivity analysis of the primary endpoint with missing endpoints imputed (ITT population)

Analysis	Arm 1 (N)	Arm 2 (N)	Crude RR (95% CI)	P-value	Adjusted RR (95% CI)	P-value
DP v SP						
Adverse pregnancy outcome (primary analysis)	1442	1435	1.20 (1.06, 1.36)	0.0040	1.20 (1.06, 1.36)	0.0035
Adverse pregnancy outcome (imputed missing values)	1558	1560	1.19 (1.05, 1.35)	0.0063	1.19 (1.05, 1.35)	0.0057
DP+AZ v SP						
Adverse pregnancy outcome (primary analysis)	1433	1435	1.16 (1.03, 1.32)	0.017	1.17 (1.03, 1.32)	0.015
Adverse pregnancy outcome (imputed missing values)	1557	1560	1.17 (1.03, 1.32)	0.015	1.17 (1.03, 1.32)	0.014
DP+AZ v DP						
Adverse pregnancy outcome (primary analysis)	1433	1442	0.97 (0.86, 1.09)	0.62	0.97 (0.86, 1.09)	0.61
Adverse pregnancy outcome (imputed missing values)	1557	1558	0.98 (0.87, 1.10)	0.73	0.98 (0.87, 1.10)	0.72

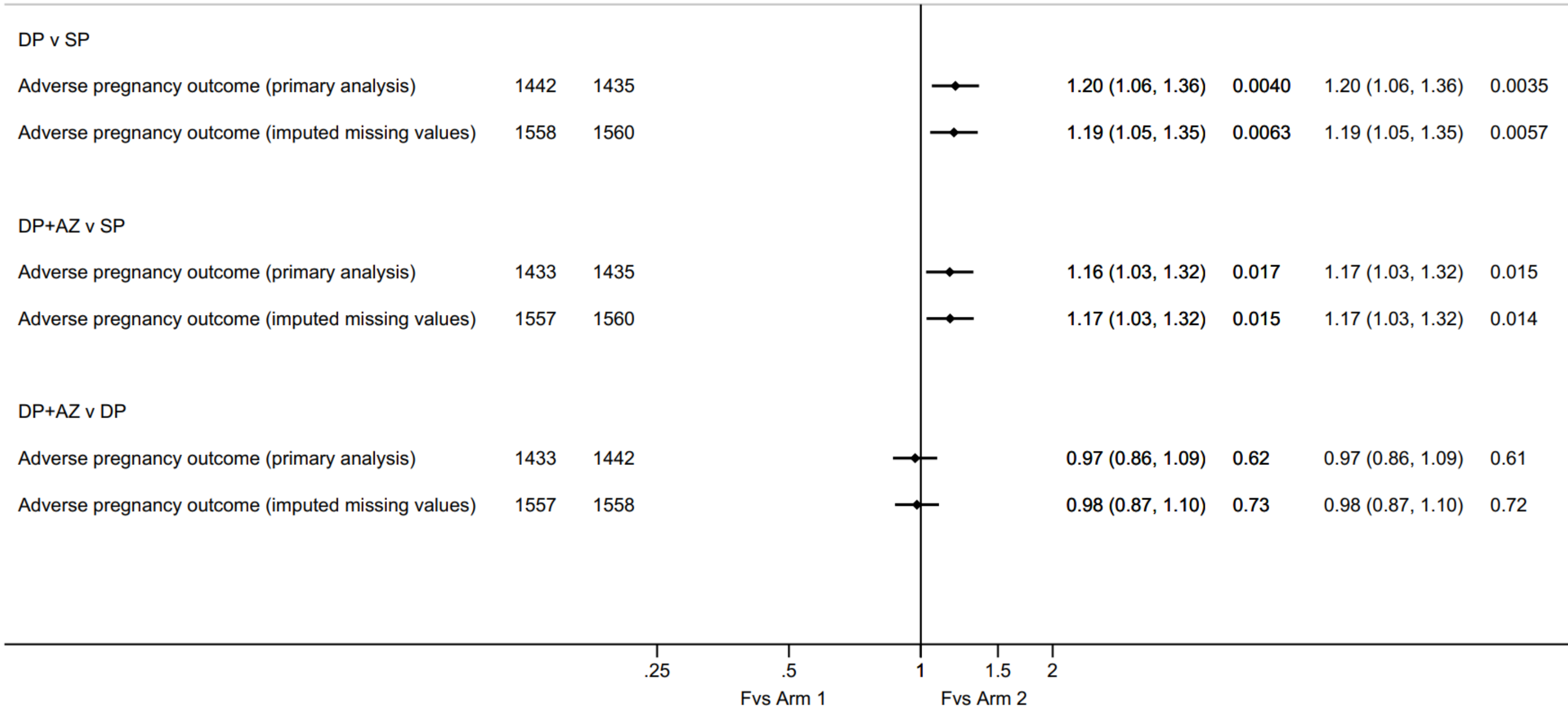

Adverse pregnancy outcome is defined as either fetal loss, LBW/Preterm/SGA or neonatal death. Missing primary endpoints for non-responder were imputed using multiple imputations. The crude RR values were obtained from log-binomial regressions with the stratification factors site and gravidity (paucigravidae and multigravidae) included as covariates. The adjusted RR values were adjusted for site, gravidity, and six other prespecified covariates. IPTp=intermittent preventive treatment in pregnancy. ITT=intention to treat. RR=risk ratio. CI=confidence interval, DP=dihydroartemisinin-piperaquine. AZ=azithromycin. SP=sulfadoxine-pyrimethamine. LBW=low birthweight. SGA=small for gestational age. Fvs=favours

FIGURE S3: SUBGROUP ANALYSIS OF THE PRIMARY ENDPOINT (MODIFIED ITT POPULATION) (DP VS SP)

DP vs SP Subgroup	DP n/N(%)	SP n/N(%)	Crude RR (95% CI)	P-value	P-value for interaction
**Country**					
Kenya	85/473 (18.0%)	74/462 (16.0%)	1.13 (0.85, 1.50)	0.40	0.79
Malawi	129/430 (30.0%)	107/424 (25.2%)	1.17 (0.94, 1.46)	0.15	
Tanzania	189/539 (35.1%)	154/549 (28.1%)	1.25 (1.05, 1.49)	0.012	
**Study site**					
Kenya - Ahero	34/191 (17.8%)	31/184 (16.8%)	1.06 (0.68, 1.64)	0.81	0.0023
Kenya - Akala	3/30 (10.0%)	6/31 (19.4%)	0.52 (0.14, 1.89)	0.32	
Kenya - Homa Bay	11/89 (12.4%)	12/85 (14.1%)	0.88 (0.41, 1.88)	0.74	
Kenya - Rabour	37/163 (22.7%)	25/162 (15.4%)	1.47 (0.93, 2.32)	0.10	
Malawi - Chikwawa	43/135 (31.9%)	29/133 (21.8%)	1.46 (0.97, 2.19)	0.067	
Malawi - Madziabango	9/50 (18.0%)	23/49 (46.9%)	0.38 (0.20, 0.73)	0.0039	
Malawi - Mangochi	25/90 (27.8%)	17/88 (19.3%)	1.46 (0.85, 2.50)	0.17	
Malawi - Mpemba	35/84 (41.7%)	20/86 (23.3%)	1.79 (1.13, 2.83)	0.013	
Malawi - Zomba	17/71 (23.9%)	18/68 (26.5%)	0.91 (0.52, 1.60)	0.74	
Tanzania - Handeni	49/133 (36.8%)	39/131 (29.8%)	1.28 (0.91, 1.80)	0.15	
Tanzania - Korogwe	125/349 (35.8%)	90/349 (25.8%)	1.39 (1.11, 1.74)	0.0041	
Tanzania - Mkata	15/57 (26.3%)	25/69 (36.2%)	0.69 (0.41, 1.17)	0.17	
**SP parasite resistance**					
High	214/903 (23.7%)	181/886 (20.4%)	1.16 (0.97, 1.38)	0.10	0.52
Very high	189/539 (35.1%)	154/549 (28.1%)	1.25 (1.05, 1.49)	0.012	
**Malaria transmission intensity**					
Moderate	237/739 (32.1%)	187/735 (25.4%)	1.29 (1.10, 1.51)	0.0018	0.20
High	166/703 (23.6%)	148/700 (21.1%)	1.10 (0.91, 1.34)	0.32	
**Malaria status by microscopy**					
Negative	368/1308 (28.1%)	304/1295 (23.5%)	1.21 (1.06, 1.37)	0.0048	0.87
Positive	35/134 (26.1%)	31/140 (22.1%)	1.17 (0.77, 1.77)	0.46	
**Gravidity**					
Paucigravidae	244/791 (30.8%)	208/784 (26.5%)	1.16 (1.00, 1.36)	0.055	0.61
Multigravidae	159/651 (24.4%)	127/651 (19.5%)	1.27 (1.04, 1.56)	0.022	
**Season**					
Dry	136/468 (29.1%)	115/505 (22.8%)	1.27 (1.02, 1.56)	0.029	0.32
Intermediate	128/486 (26.3%)	116/468 (24.8%)	1.05 (0.85, 1.31)	0.63	
Wet	139/488 (28.5%)	104/462 (22.5%)	1.28 (1.03, 1.59)	0.027	
**Socio-Economic Status (SES)**					
Low	147/468 (31.4%)	119/480 (24.8%)	1.28 (1.04, 1.57)	0.020	0.74
Medium	136/493 (27.6%)	117/483 (24.2%)	1.14 (0.93, 1.41)	0.21	
High	120/481 (24.9%)	99/472 (21.0%)	1.21 (0.96, 1.52)	0.11	
**Number of IPTp courses received**					
1-3	81/215 (37.7%)	97/277 (35.0%)	1.03 (0.82, 1.31)	0.78	<0.0001
4	108/360 (30.0%)	86/388 (22.2%)	1.31 (1.03, 1.66)	0.027	
5	132/509 (25.9%)	98/458 (21.4%)	1.21 (0.96, 1.51)	0.10	
6+	82/358 (22.9%)	54/312 (17.3%)	1.38 (1.02, 1.88)	0.037	

.25 .5 1 1.5 2

Fvs DP Fvs SP

Adverse pregnancy outcome is defined as either fetal loss, LBW/Preterm/SGA or neonatal death. The crude RR values were obtained from log-binomial regressions with the stratification factors site and gravidity (paucigravidae and multigravidae) included as covariates. The adjusted RR values were adjusted for site, gravidity, and six other prespecified covariates. IPTp=intermittent preventive treatment in pregnancy. ITT=intention to treat. RR=risk ratio. CI=confidence interval, DP=dihydroartemisinin-piperaquine. AZ=azithromycin. SP=sulfadoxine-pyrimethamine. LBW=low birthweight. SGA=small for gestational age. Fvs=favours.

Figure S4: Subgroup analysis of the primary endpoint (modified ITT population) (DPAZ vs SP)

DP+AZ vs SP	Subgroup	DP+AZ n/N(%)	SP n/N(%)	Crude RR (95% CI)	P-value	P-value for interaction
Country						
	Kenya	117/473 (24.7%)	74/462 (16.0%)	1.54 (1.19, 1.99)	0.0012	0.037
	Malawi	125/425 (29.4%)	107/424 (25.2%)	1.15 (0.93, 1.44)	0.20	
	Tanzania	154/535 (28.8%)	154/549 (28.1%)	1.01 (0.84, 1.22)	0.93	
Study site						
	Kenya - Ahero	45/187 (24.1%)	31/184 (16.8%)	1.42 (0.94, 2.13)	0.095	0.23
	Kenya - Akala	11/33 (33.3%)	6/31 (19.4%)	1.72 (0.73, 4.06)	0.22	
	Kenya - Homa Bay	15/86 (17.4%)	12/85 (14.1%)	1.24 (0.62, 2.49)	0.54	
	Kenya - Rabour	46/167 (27.5%)	25/162 (15.4%)	1.77 (1.15, 2.74)	0.010	
	Malawi - Chikwawa	39/135 (28.9%)	29/133 (21.8%)	1.32 (0.87, 2.01)	0.19	
	Malawi - Madziabango	19/53 (35.8%)	23/49 (46.9%)	0.75 (0.47, 1.18)	0.21	
	Malawi - Mangochi	22/85 (25.9%)	17/88 (19.3%)	1.33 (0.76, 2.32)	0.32	
	Malawi - Mpemba	20/82 (24.4%)	20/86 (23.3%)	1.06 (0.62, 1.83)	0.82	
	Malawi - Zomba	25/70 (35.7%)	18/68 (26.5%)	1.36 (0.83, 2.23)	0.22	
	Tanzania - Handeni	31/123 (25.2%)	39/131 (29.8%)	0.86 (0.58, 1.29)	0.47	
	Tanzania - Korogwe	104/353 (29.5%)	90/349 (25.8%)	1.12 (0.88, 1.42)	0.36	
	Tanzania - Mkata	19/59 (32.2%)	25/69 (36.2%)	0.83 (0.52, 1.34)	0.45	
SP parasite resistance						
	High	242/898 (26.9%)	181/886 (20.4%)	1.30 (1.10, 1.54)	0.0020	0.064
	Very high	154/535 (28.8%)	154/549 (28.1%)	1.01 (0.84, 1.22)	0.93	
Malaria transmission intensity						
	Moderate	213/736 (28.9%)	187/735 (25.4%)	1.13 (0.95, 1.33)	0.16	0.54
	High	183/697 (26.3%)	148/700 (21.1%)	1.22 (1.01, 1.47)	0.036	
Malaria status by microscopy						
	Negative	345/1264 (27.3%)	304/1295 (23.5%)	1.14 (1.00, 1.31)	0.045	0.32
	Positive	51/169 (30.2%)	31/140 (22.1%)	1.37 (0.94, 2.00)	0.100	
Gravidity						
	Paucigravidae	224/793 (28.2%)	208/784 (26.5%)	1.05 (0.89, 1.23)	0.58	0.036
	Multigravidae	172/640 (26.9%)	127/651 (19.5%)	1.38 (1.13, 1.68)	0.0017	
Season						
	Dry	125/453 (27.6%)	115/505 (22.8%)	1.18 (0.95, 1.47)	0.12	0.92
	Intermediate	135/474 (28.5%)	116/468 (24.8%)	1.12 (0.91, 1.38)	0.29	
	Wet	136/506 (26.9%)	104/462 (22.5%)	1.16 (0.93, 1.45)	0.18	
Socio-Economic Status (SES)						
	Low	157/486 (32.3%)	119/480 (24.8%)	1.29 (1.05, 1.57)	0.014	0.51
	Medium	119/467 (25.5%)	117/483 (24.2%)	1.04 (0.83, 1.29)	0.74	
	High	120/480 (25.0%)	99/472 (21.0%)	1.16 (0.92, 1.46)	0.22	
Number of IPTp courses received						
	1-3	82/213 (38.5%)	97/277 (35.0%)	1.05 (0.83, 1.32)	0.69	0.29
	4	112/436 (25.7%)	86/388 (22.2%)	1.07 (0.84, 1.36)	0.61	
	5	118/453 (26.0%)	98/458 (21.4%)	1.21 (0.96, 1.53)	0.10	
	6+	84/331 (25.4%)	54/312 (17.3%)	1.50 (1.11, 2.04)	0.0080	

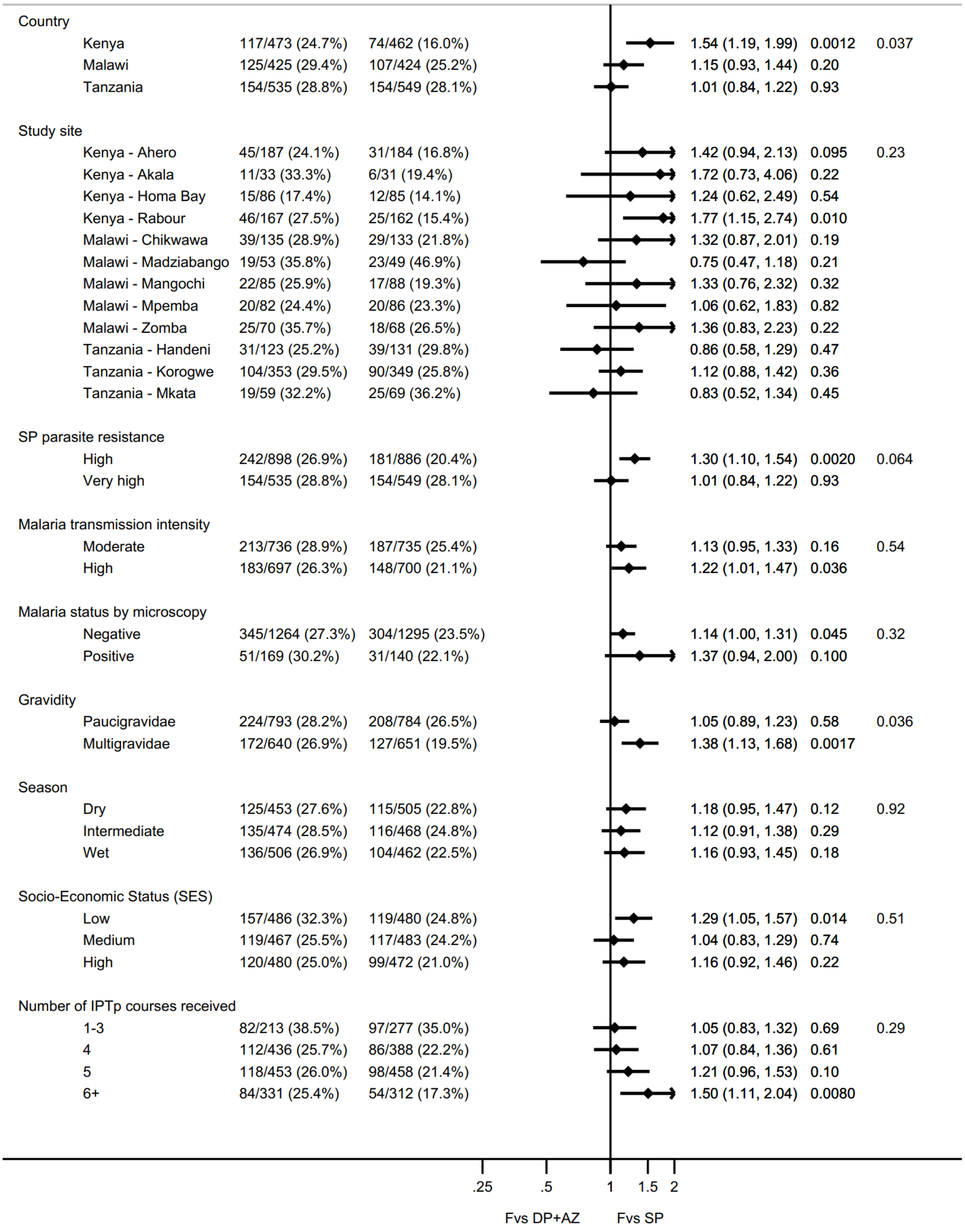

Adverse pregnancy outcome is defined as either fetal loss, LBW/Preterm/SGA or neonatal death. The crude RR values were obtained from log-binomial regressions with the stratification factors site and gravidity (paucigravidae and multigravidae) included as covariates. The adjusted RR values were adjusted for site, gravidity, and six other prespecified covariates. IPTp=intermittent preventive treatment in pregnancy. ITT=intention to treat. RR=risk ratio. CI=confidence interval, DP=dihydroartemisinin-piperaquine. AZ=azithromycin. SP=sulfadoxine-pyrimethamine. LBW=low birthweight. SGA=small for gestational age. Fvs=favours.

FIGURE S5: SUBGROUP ANALYSIS OF THE PRIMARY ENDPOINT (MODIFIED ITT POPULATION) (DPAZ VS DP)

DP+AZ vs DP	Subgroup	DP+AZ n/N(%)	DP n/N(%)	Crude RR (95% CI)	P-value	P-value for interaction
Country						
	Kenya	117/473 (24.7%)	85/473 (18.0%)	1.36 (1.06, 1.75)	0.014	0.0032
	Malawi	125/425 (29.4%)	129/430 (30.0%)	0.98 (0.80, 1.21)	0.87	
	Tanzania	154/535 (28.8%)	189/539 (35.1%)	0.81 (0.68, 0.96)	0.015	
Study site						
	Kenya - Ahero	45/187 (24.1%)	34/191 (17.8%)	1.34 (0.90, 1.99)	0.15	0.0015
	Kenya - Akala	11/33 (33.3%)	3/30 (10.0%)	3.30 (1.02, 10.69)	0.046	
	Kenya - Homa Bay	15/86 (17.4%)	11/89 (12.4%)	1.41 (0.69, 2.90)	0.34	
	Kenya - Rabour	46/167 (27.5%)	37/163 (22.7%)	1.21 (0.83, 1.76)	0.33	
	Malawi - Chikwawa	39/135 (28.9%)	43/135 (31.9%)	0.91 (0.63, 1.30)	0.60	
	Malawi - Madziabango	19/53 (35.8%)	9/50 (18.0%)	1.97 (0.99, 3.92)	0.055	
	Malawi - Mangochi	22/85 (25.9%)	25/90 (27.8%)	0.91 (0.56, 1.49)	0.71	
	Malawi - Mpemba	20/82 (24.4%)	35/84 (41.7%)	0.60 (0.38, 0.94)	0.026	
	Malawi - Zomba	25/70 (35.7%)	17/71 (23.9%)	1.50 (0.90, 2.49)	0.12	
	Tanzania - Handeni	31/123 (25.2%)	49/133 (36.8%)	0.67 (0.46, 0.98)	0.037	
	Tanzania - Korogwe	104/353 (29.5%)	125/349 (35.8%)	0.80 (0.65, 1.00)	0.045	
	Tanzania - Mkata	19/59 (32.2%)	15/57 (26.3%)	1.21 (0.69, 2.12)	0.51	
SP parasite resistance						
	High	242/898 (26.9%)	214/903 (23.7%)	1.13 (0.96, 1.32)	0.14	0.0069
	Very high	154/535 (28.8%)	189/539 (35.1%)	0.81 (0.68, 0.96)	0.015	
Malaria transmission intensity						
	Moderate	213/736 (28.9%)	237/739 (32.1%)	0.87 (0.75, 1.02)	0.079	0.060
	High	183/697 (26.3%)	166/703 (23.6%)	1.11 (0.92, 1.32)	0.27	
Malaria status by microscopy						
	Negative	345/1264 (27.3%)	368/1308 (28.1%)	0.95 (0.84, 1.07)	0.41	0.34
	Positive	51/169 (30.2%)	35/134 (26.1%)	1.17 (0.83, 1.67)	0.37	
Gravidity						
	Paucigravidae	224/793 (28.2%)	244/791 (30.8%)	0.90 (0.77, 1.04)	0.16	0.10
	Multigravidae	172/640 (26.9%)	159/651 (24.4%)	1.09 (0.90, 1.30)	0.39	
Season						
	Dry	125/453 (27.6%)	136/468 (29.1%)	0.94 (0.76, 1.15)	0.52	0.55
	Intermediate	135/474 (28.5%)	128/486 (26.3%)	1.06 (0.87, 1.30)	0.56	
	Wet	136/506 (26.9%)	139/488 (28.5%)	0.91 (0.75, 1.11)	0.36	
Socio-Economic Status (SES)						
	Low	157/486 (32.3%)	147/468 (31.4%)	1.01 (0.84, 1.21)	0.92	0.77
	Medium	119/467 (25.5%)	136/493 (27.6%)	0.91 (0.74, 1.12)	0.36	
	High	120/480 (25.0%)	120/481 (24.9%)	0.96 (0.77, 1.19)	0.71	
Number of IPTp courses received						
	1-3	82/213 (38.5%)	81/215 (37.7%)	1.01 (0.80, 1.29)	0.91	0.42
	4	112/436 (25.7%)	108/360 (30.0%)	0.81 (0.65, 1.01)	0.064	
	5	118/453 (26.0%)	132/509 (25.9%)	1.01 (0.81, 1.24)	0.96	
	6+	84/331 (25.4%)	82/358 (22.9%)	1.09 (0.84, 1.41)	0.53	

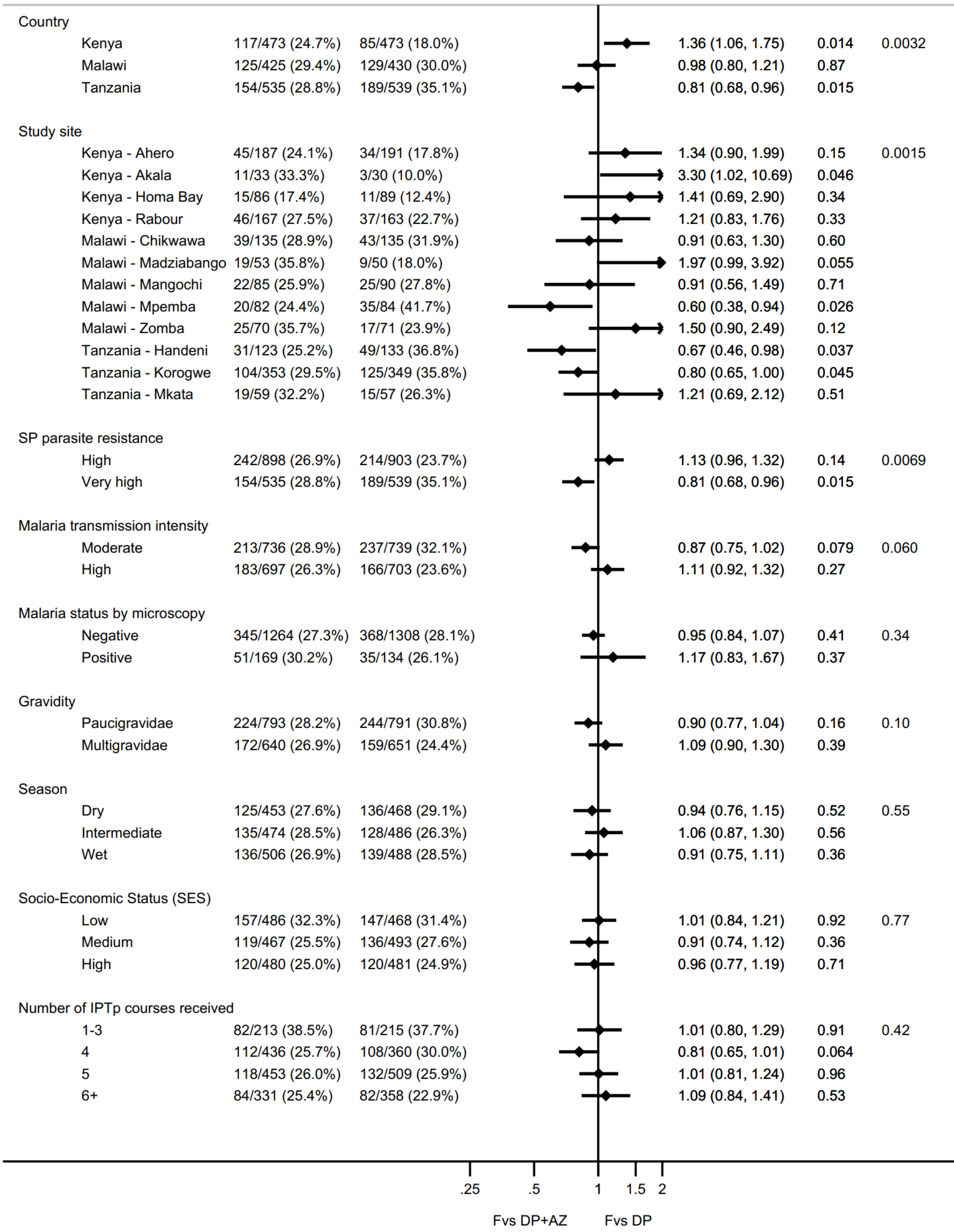

Adverse pregnancy outcome is defined as either fetal loss, LBW/Preterm/SGA or neonatal death. The crude RR values were obtained from log-binomial regressions with the stratification factors site and gravidity (paucigravidae and multigravidae) included as covariates. The adjusted RR values were adjusted for site, gravidity, and six other prespecified covariates. IPTp=intermittent preventive treatment in pregnancy. ITT=intention to treat. RR=risk ratio. CI=confidence interval, DP=dihydroartemisinin-piperaquine. AZ=azithromycin. SP=sulfadoxine-pyrimethamine. LBW=low birthweight. SGA=small for gestational age. Fvs=favours.

FIGURE S6: EFFICACY OF IPTP-DP AND IPTP-DPAZ VERSUS IPTP-SP ON OTHER SECONDARY EFFICACY ENDPOINTS NEWBORN (MODIFIED ITT POPULATION)

Binary secondary endpoints - infant	Arm 1, n/N(%)	Arm 2, n/N(%)	Crude RR (95% CI)	P-value	Adjusted RR (95% CI)	P-value
**DP v SP**						
Miscarriage	6/1441 (0.4%)	6/1431 (0.4%)	0.99 (0.32, 3.06)	0.99	0.94 (0.31, 2.91)	0.92
Stillbirth	23/1435 (1.6%)	17/1425 (1.2%)	1.35 (0.72, 2.51)	0.35	1.37 (0.73, 2.54)	0.33
Fetal or neonatal death*	44/1442 (3.1%)	43/1435 (3.0%)	1.02 (0.67, 1.54)	0.93	1.02 (0.68, 1.54)	0.92
Early neonatal death*	11/1283 (0.9%)	14/1268 (1.1%)	0.78 (0.36, 1.70)	0.53	0.78 (0.36, 1.71)	0.54
Perinatal death*	34/1435 (2.4%)	31/1425 (2.2%)	1.09 (0.67, 1.76)	0.73	1.10 (0.68, 1.78)	0.69
SGA/LBW/PT	390/1442 (27.0%)	318/1435 (22.2%)	1.22 (1.08, 1.39)	0.0020	1.22 (1.08, 1.39)	0.0018
Fetal anaemia	194/1185 (16.4%)	161/1182 (13.6%)	1.16 (0.97, 1.40)	0.10	1.18 (0.98, 1.41)	0.079
Neonatal stunting	175/1296 (13.5%)	174/1299 (13.4%)	0.97 (0.81, 1.17)	0.78	0.99 (0.83, 1.19)	0.93
Neonatal wasting*	108/1388 (7.8%)	77/1371 (5.6%)	1.41 (1.06, 1.86)	0.017	1.41 (1.07, 1.87)	0.016
Neonatal stunting or wasting*	249/1309 (19.0%)	221/1306 (16.9%)	1.09 (0.93, 1.27)	0.28	1.10 (0.95, 1.29)	0.21
**DP+AZ v SP**						
Miscarriage	10/1431 (0.7%)	6/1431 (0.4%)	1.65 (0.60, 4.51)	0.33	1.59 (0.58, 4.36)	0.36
Stillbirth	24/1421 (1.7%)	17/1425 (1.2%)	1.42 (0.77, 2.62)	0.27	1.43 (0.77, 2.66)	0.25
Fetal or neonatal death*	51/1433 (3.6%)	43/1435 (3.0%)	1.19 (0.80, 1.77)	0.40	1.20 (0.80, 1.79)	0.37
Early neonatal death*	15/1251 (1.2%)	14/1268 (1.1%)	1.09 (0.53, 2.24)	0.82	1.12 (0.55, 2.31)	0.75
Perinatal death*	39/1421 (2.7%)	31/1425 (2.2%)	1.26 (0.79, 2.01)	0.32	1.28 (0.81, 2.05)	0.29
SGA/LBW/PT	374/1433 (26.1%)	318/1435 (22.2%)	1.16 (1.02, 1.32)	0.023	1.16 (1.02, 1.32)	0.022
Fetal anaemia	160/1130 (14.2%)	161/1182 (13.6%)	1.05 (0.87, 1.27)	0.62	1.07 (0.88, 1.30)	0.50
Neonatal stunting	177/1252 (14.1%)	174/1299 (13.4%)	1.03 (0.86, 1.23)	0.77	1.04 (0.87, 1.25)	0.64
Neonatal wasting*	107/1352 (7.9%)	77/1371 (5.6%)	1.41 (1.07, 1.87)	0.016	1.42 (1.07, 1.88)	0.014
Neonatal stunting or wasting*	249/1256 (19.8%)	221/1306 (16.9%)	1.14 (0.98, 1.33)	0.096	1.15 (0.99, 1.35)	0.068
**DP+AZ v DP**						
Miscarriage	10/1431 (0.7%)	6/1441 (0.4%)	1.66 (0.61, 4.54)	0.32	1.69 (0.62, 4.64)	0.31
Stillbirth	24/1421 (1.7%)	23/1435 (1.6%)	1.05 (0.60, 1.85)	0.86	1.05 (0.60, 1.85)	0.87
Fetal or neonatal death*	51/1433 (3.6%)	44/1442 (3.1%)	1.17 (0.78, 1.73)	0.45	1.17 (0.79, 1.75)	0.43
Early neonatal death*	15/1251 (1.2%)	11/1283 (0.9%)	1.40 (0.65, 3.03)	0.39	1.44 (0.66, 3.11)	0.36
Perinatal death*	39/1421 (2.7%)	34/1435 (2.4%)	1.16 (0.74, 1.83)	0.52	1.17 (0.74, 1.83)	0.51
SGA/LBW/PT	374/1433 (26.1%)	390/1442 (27.0%)	0.95 (0.84, 1.07)	0.40	0.95 (0.84, 1.07)	0.39
Fetal anaemia	160/1130 (14.2%)	194/1185 (16.4%)	0.90 (0.75, 1.08)	0.27	0.91 (0.76, 1.09)	0.30
Neonatal stunting	177/1252 (14.1%)	175/1296 (13.5%)	1.05 (0.88, 1.26)	0.56	1.05 (0.88, 1.26)	0.57
Neonatal wasting*	107/1352 (7.9%)	108/1388 (7.8%)	1.00 (0.78, 1.30)	0.97	1.01 (0.78, 1.30)	0.96
Neonatal stunting or wasting*	249/1256 (19.8%)	249/1309 (19.0%)	1.05 (0.90, 1.21)	0.53	1.05 (0.90, 1.21)	0.55

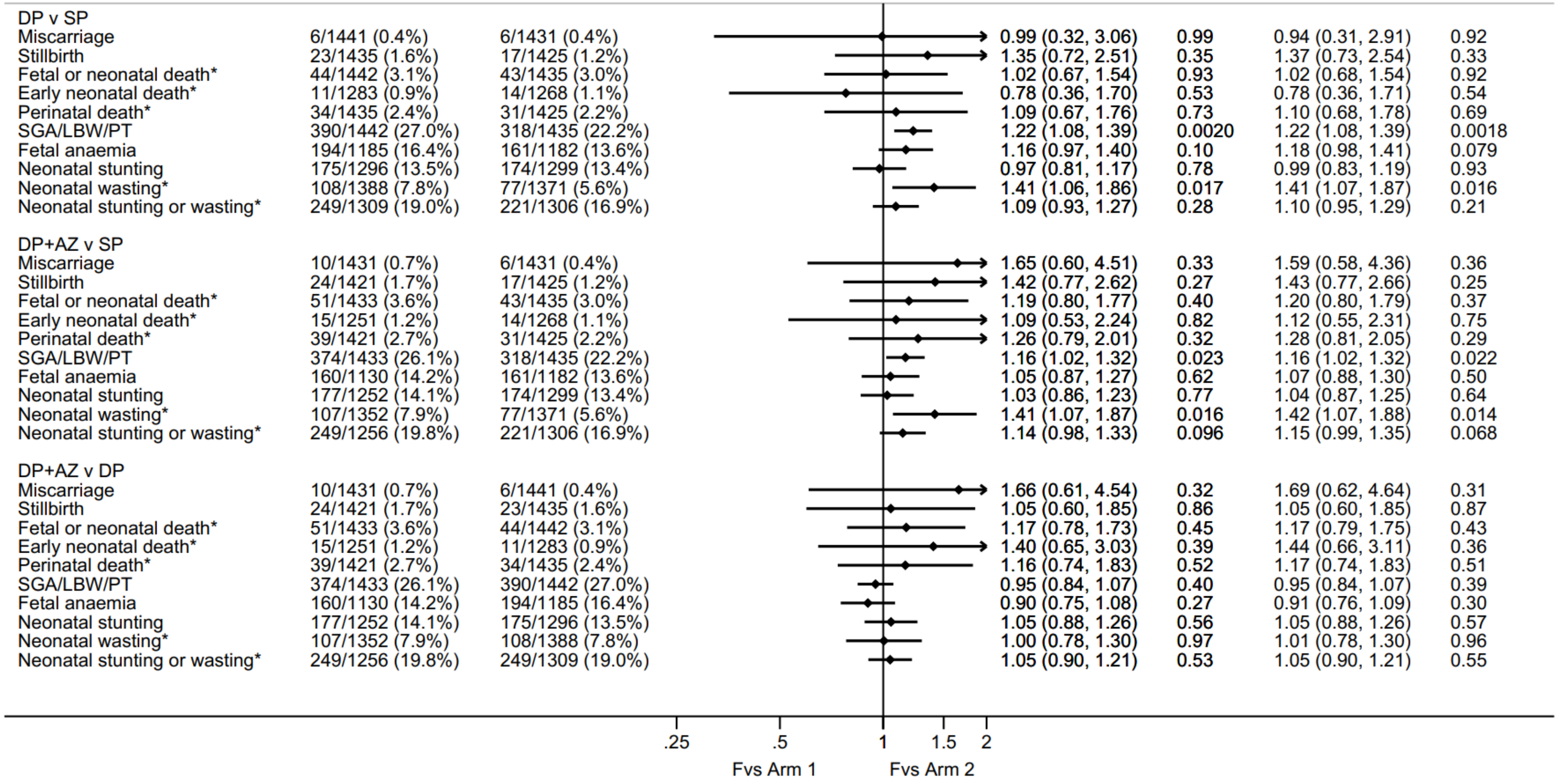

* Post-hoc. The crude RR values were obtained from log-binomial regressions with the stratification factors site and gravidity (paucigravidae and multigravidae) included as covariates. P-values and the widths of the confidence intervals for the secondary endpoints have not been adjusted for multiplicity, so the values should not be used to infer definitive treatment effects. ITT=intention to treat. IPTp=intermittent preventive treatment in pregnancy. RR=risk ratio. CI=confidence interval, DP=dihydroartemisinin-piperaquine. AZ=azithromycin. SP=sulfadoxine-pyrimethamine. LBW=low birthweight. PT=preterm. SGA=small for gestational age. Fvs=favours

Figure S7: Efficacy of IPTp-DP and IPTp-DPAZ versus IPTp-SP on malaria infection at delivery (modified ITT population)

Endpoints	Arm 1, n/N(%)	Arm 2, n/N(%)	Crude RR (95% CI)	P-value	Adjusted RR (95% CI)	P-value
DP v SP						
Maternal malaria (microscopy)	22/1328 (1.7%)	48/1332 (3.6%)	**0.46 (0.28, 0.75)**	**0.0021**	0.46 (0.28, 0.75)	0.0021
Maternal malaria (PCR)	39/1193 (3.3%)	82/1224 (6.7%)	**0.51 (0.35, 0.74)**	**0.0003**	0.51 (0.35, 0.73)	0.0003
Placental malaria (any)	62/1309 (4.7%)	89/1305 (6.8%)	**0.70 (0.51, 0.96)**	**0.025**	0.70 (0.51, 0.96)	0.025
Placental malaria (microscopy)	10/1239 (0.8%)	9/1238 (0.7%)	**1.08 (0.44, 2.65)**	**0.86**	1.07 (0.44, 2.62)	0.88
Placental malaria (PCR placental blood)	49/1105 (4.4%)	69/1100 (6.3%)	**0.72 (0.51, 1.03)**	**0.072**	0.72 (0.51, 1.03)	0.071
Congenital malaria infection	8/1233 (0.6%)	1/1251 (0.1%)	**8.08 (1.01, 64.44)**	**0.048**	8.13 (1.02, 64.79)	0.048
DP+AZ v SP						
Maternal malaria (microscopy)	32/1286 (2.5%)	48/1332 (3.6%)	**0.69 (0.45, 1.07)**	**0.10**	0.70 (0.45, 1.09)	0.12
Maternal malaria (PCR)	55/1172 (4.7%)	82/1224 (6.7%)	**0.72 (0.52, 0.99)**	**0.045**	0.71 (0.51, 0.98)	0.037
Placental malaria (any)	59/1264 (4.7%)	89/1305 (6.8%)	**0.69 (0.50, 0.94)**	**0.020**	0.67 (0.49, 0.92)	0.014
Placental malaria (microscopy)	10/1198 (0.8%)	9/1238 (0.7%)	**1.14 (0.47, 2.80)**	**0.77**	1.12 (0.46, 2.75)	0.80
Placental malaria (PCR placental blood)	41/1055 (3.9%)	69/1100 (6.3%)	**0.63 (0.43, 0.91)**	**0.014**	0.61 (0.42, 0.88)	0.0089
Congenital malaria infection	3/1209 (0.2%)	1/1251 (0.1%)	**3.01 (0.31, 28.88)**	**0.34**	3.10 (0.32, 29.66)	0.33
DP+AZ v DP						
Maternal malaria (microscopy)	32/1286 (2.5%)	22/1328 (1.7%)	**1.51 (0.88, 2.58)**	**0.13**	1.53 (0.90, 2.62)	0.12
Maternal malaria (PCR)	55/1172 (4.7%)	39/1193 (3.3%)	**1.41 (0.95, 2.09)**	**0.092**	1.40 (0.94, 2.08)	0.10
Placental malaria (any)	59/1264 (4.7%)	62/1309 (4.7%)	**0.98 (0.69, 1.39)**	**0.92**	0.96 (0.68, 1.36)	0.82
Placental malaria (microscopy)	10/1198 (0.8%)	10/1239 (0.8%)	**1.06 (0.44, 2.52)**	**0.90**	1.05 (0.44, 2.50)	0.92
Placental malaria (PCR placental blood)	41/1055 (3.9%)	49/1105 (4.4%)	**0.87 (0.58, 1.29)**	**0.48**	0.84 (0.56, 1.26)	0.40
Congenital malaria infection	3/1209 (0.2%)	8/1233 (0.6%)	**0.37 (0.10, 1.40)**	**0.14**	0.38 (0.10, 1.43)	0.15

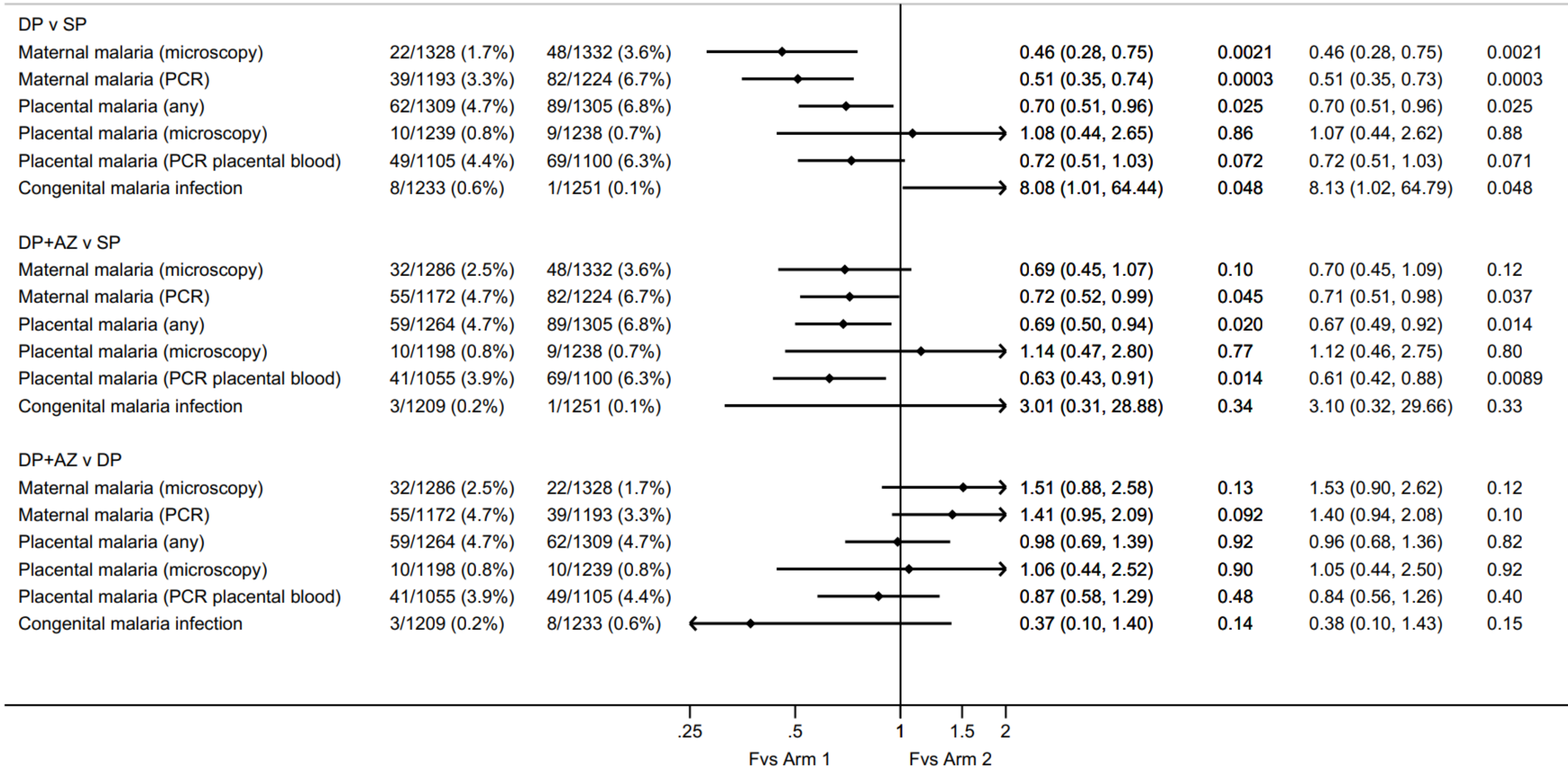

The crude RR values were obtained from log-binomial regressions with the stratification factors site and gravidity (paucigravidae and multigravidae) included as covariates. The adjusted RR values were adjusted for site, gravidity, and six other prespecified covariates. IPTp= intermittent preventive treatment in pregnancy. ITT=intention to treat. IRR=incidence rate ratio. RR=risk ratio. CI=confidence interval, DP=dihydroartemisinin-piperaquine. AZ=azithromycin. SP=sulfadoxine-pyrimethamine. LBW=low birthweight. SGA=small for gestational age. Fvs=favours.

FIGURE S8: EFFICACY OF IPTP-DP AND IPTP-DPAZ VERSUS IPTP-SP ON PLACENTAL MALARIA (HISTOLOGY) (MODIFIED ITT POPULATION)

Exploratory outcomes	Arm 1, n/N(%)	Arm 2, n/N(%)	Crude RR (95% CI)	P-value	Adjusted RR (95% CI)	P-value
DP v SP						
Placental histology (any)	137/1204 (11.4%)	213/1208 (17.6%)	0.66 (0.55, 0.79)	<0.0001	0.66 (0.55, 0.78)	<0.0001
Placental histology (active)	8/1204 (0.7%)	20/1208 (1.7%)	0.40 (0.18, 0.90)	0.027	0.40 (0.18, 0.91)	0.029
Placental histology (active-acute)	3/1203 (0.2%)	10/1205 (0.8%)	0.30 (0.08, 1.09)	0.067	0.30 (0.08, 1.09)	0.068
Placental histology (acive-chronic)	5/1203 (0.4%)	10/1205 (0.8%)	0.50 (0.17, 1.45)	0.20	0.51 (0.17, 1.48)	0.21
Placental histology (past)	129/1203 (10.7%)	193/1205 (16.0%)	0.69 (0.57, 0.83)	0.0001	0.69 (0.57, 0.82)	<0.0001
Placental inflammation or chorioamnionitis*	358/1204 (29.7%)	349/1208 (28.9%)	1.03 (0.92, 1.16)	0.61	1.02 (0.91, 1.15)	0.72
DP+AZ v SP						
Placental histology (any)	180/1171 (15.4%)	213/1208 (17.6%)	0.85 (0.73, 1.00)	0.053	0.83 (0.71, 0.97)	0.023
Placental histology (active)	14/1171 (1.2%)	20/1208 (1.7%)	0.71 (0.36, 1.40)	0.33	0.72 (0.37, 1.41)	0.34
Placental histology (active-acute)	7/1168 (0.6%)	10/1205 (0.8%)	0.73 (0.28, 1.90)	0.51	0.74 (0.28, 1.93)	0.53
Placental histology (acive-chronic)	7/1168 (0.6%)	10/1205 (0.8%)	0.71 (0.27, 1.85)	0.48	0.70 (0.27, 1.83)	0.47
Placental histology (past)	166/1170 (14.2%)	193/1205 (16.0%)	0.88 (0.74, 1.04)	0.15	0.84 (0.71, 1.00)	0.046
Placental inflammation or chorioamnionitis*	338/1171 (28.9%)	349/1208 (28.9%)	1.00 (0.88, 1.13)	0.96	0.98 (0.87, 1.10)	0.71
DP+AZ v DP						
Placental histology (any)	180/1171 (15.4%)	137/1204 (11.4%)	1.30 (1.08, 1.58)	0.0064	1.26 (1.05, 1.51)	0.013
Placental histology (active)	14/1171 (1.2%)	8/1204 (0.7%)	1.79 (0.75, 4.24)	0.19	1.78 (0.75, 4.21)	0.19
Placental histology (active-acute)	7/1168 (0.6%)	3/1203 (0.2%)	2.41 (0.63, 9.29)	0.20	2.45 (0.63, 9.43)	0.19
Placental histology (acive-chronic)	7/1168 (0.6%)	5/1203 (0.4%)	1.42 (0.45, 4.46)	0.54	1.38 (0.44, 4.34)	0.58
Placental histology (past)	166/1170 (14.2%)	129/1203 (10.7%)	1.28 (1.05, 1.57)	0.013	1.23 (1.02, 1.49)	0.033
Placental inflammation or chorioamnionitis*	338/1171 (28.9%)	358/1204 (29.7%)	0.97 (0.86, 1.09)	0.57	0.96 (0.85, 1.08)	0.46

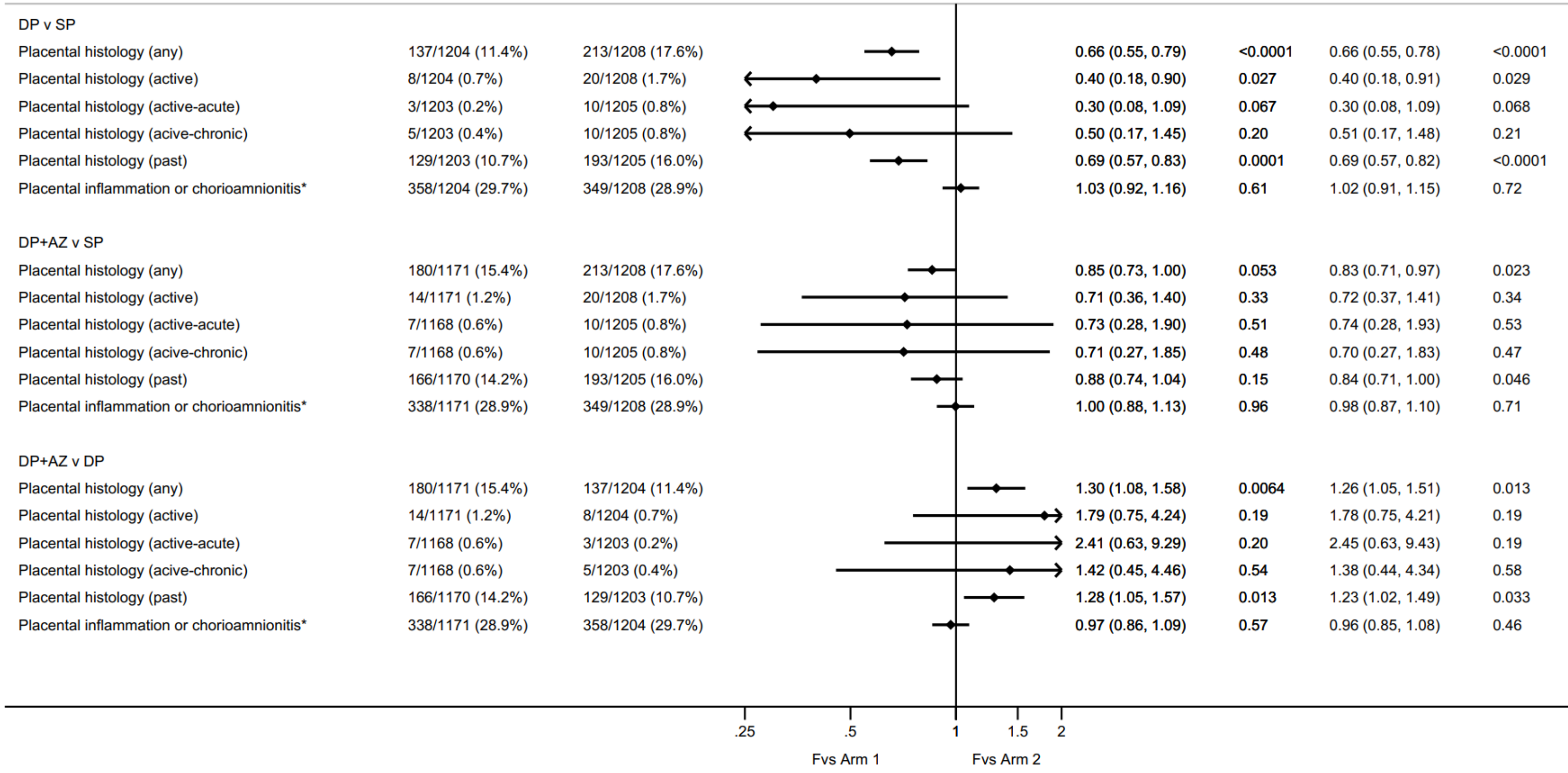

* Post-hoc. The crude RR values were obtained from log-binomial regressions with the stratification factors site and gravidity (paucigravidae and multigravidae) included as covariates. The adjusted RR values were adjusted for site, gravidity, and six other prespecified covariates. P-values and the widths of the confidence intervals for the secondary endpoints have not been adjusted for multiplicity, so the values should not be used to infer definitive treatment effects. IPTp= intermittent preventive treatment in pregnancy. ITT=intention to treat. RR=risk ratio. CI=confidence interval, DP=dihydroartemisinin-piperaquine. AZ=azithromycin. SP=sulfadoxine-pyrimethamine. Fvs=favours

Figure S9: Efficacy of IPTp-DP and IPTp-DPAZ versus IPTp-SP on maternal anaemia (modified ITT population)

Endpoints	Arm 1, n/N(%)	Arm 2, n/N(%)	Crude RR (95% CI)	P-value	Adjusted RR (95% CI)	P-value
**DP v SP**						
Maternal anaemia (third trimester) - any	783/1482 (52.8%)	769/1479 (52.0%)	**1.02 (0.96, 1.09)**	0.51	1.02 (0.96, 1.09)	0.56
Maternal anaemia (third trimester) - mod severe	129/1482 (8.7%)	153/1479 (10.3%)	**0.85 (0.68, 1.06)**	0.14	0.85 (0.69, 1.06)	0.16
Maternal anaemia (third trimester) - severe	11/1482 (0.7%)	16/1479 (1.1%)	**0.69 (0.32, 1.48)**	0.34	0.72 (0.33, 1.53)	0.39
Maternal anaemia (delivery) - any	156/415 (37.6%)	183/431 (42.5%)	**0.88 (0.75, 1.04)**	0.14	0.88 (0.75, 1.04)	0.13
Maternal anaemia (delivery) - mod severe	35/415 (8.4%)	46/431 (10.7%)	**0.79 (0.52, 1.19)**	0.26	0.80 (0.53, 1.20)	0.28
Maternal anaemia (delivery) - severe	3/415 (0.7%)	6/431 (1.4%)	**0.50 (0.13, 1.96)**	0.32	0.59 (0.15, 2.36)	0.46
Maternal anaemia (third trimester or delivery) - any	809/1485 (54.5%)	799/1480 (54.0%)	**1.01 (0.95, 1.08)**	0.68	1.01 (0.95, 1.08)	0.68
Maternal anaemia (third trimester or delivery) - mod severe	145/1485 (9.8%)	168/1480 (11.4%)	**0.86 (0.70, 1.06)**	0.17	0.87 (0.71, 1.07)	0.19
Maternal anaemia (third trimester or delivery) - severe	13/1485 (0.9%)	20/1480 (1.4%)	**0.65 (0.33, 1.30)**	0.23	0.68 (0.34, 1.35)	0.27
**DP+AZ v SP**						
Maternal anaemia (third trimester) - any	753/1471 (51.2%)	769/1479 (52.0%)	**0.99 (0.92, 1.05)**	0.68	0.98 (0.92, 1.05)	0.65
Maternal anaemia (third trimester) - mod severe	124/1471 (8.4%)	153/1479 (10.3%)	**0.81 (0.65, 1.01)**	0.066	0.81 (0.65, 1.01)	0.064
Maternal anaemia (third trimester) - severe	14/1471 (1.0%)	16/1479 (1.1%)	**0.88 (0.43, 1.78)**	0.71	0.88 (0.43, 1.79)	0.73
Maternal anaemia (delivery) - any	168/410 (41.0%)	183/431 (42.5%)	**0.95 (0.81, 1.11)**	0.53	0.95 (0.81, 1.11)	0.51
Maternal anaemia (delivery) - mod severe	30/410 (7.3%)	46/431 (10.7%)	**0.66 (0.43, 1.02)**	0.060	0.66 (0.43, 1.02)	0.060
Maternal anaemia (delivery) - severe	4/410 (1.0%)	6/431 (1.4%)	**0.69 (0.19, 2.41)**	0.56	0.79 (0.22, 2.79)	0.71
Maternal anaemia (third trimester or delivery) - any	788/1475 (53.4%)	799/1480 (54.0%)	**0.99 (0.93, 1.05)**	0.73	0.99 (0.93, 1.05)	0.77
Maternal anaemia (third trimester or delivery) - mod severe	138/1475 (9.4%)	168/1480 (11.4%)	**0.82 (0.66, 1.01)**	0.058	0.82 (0.66, 1.01)	0.058
Maternal anaemia (third trimester or delivery) - severe	17/1475 (1.2%)	20/1480 (1.4%)	**0.85 (0.45, 1.60)**	0.61	0.86 (0.45, 1.62)	0.63
**DP+AZ v DP**						
Maternal anaemia (third trimester) - any	753/1471 (51.2%)	783/1482 (52.8%)	**0.97 (0.90, 1.03)**	0.29	0.97 (0.91, 1.03)	0.29
Maternal anaemia (third trimester) - mod severe	124/1471 (8.4%)	129/1482 (8.7%)	**0.96 (0.76, 1.21)**	0.72	0.95 (0.75, 1.20)	0.66
Maternal anaemia (third trimester) - severe	14/1471 (1.0%)	11/1482 (0.7%)	**1.27 (0.58, 2.78)**	0.55	1.23 (0.56, 2.70)	0.60
Maternal anaemia (delivery) - any	168/410 (41.0%)	156/415 (37.6%)	**1.08 (0.91, 1.28)**	0.39	1.08 (0.91, 1.27)	0.39
Maternal anaemia (delivery) - mod severe	30/410 (7.3%)	35/415 (8.4%)	**0.84 (0.53, 1.33)**	0.45	0.83 (0.52, 1.32)	0.42
Maternal anaemia (delivery) - severe	4/410 (1.0%)	3/415 (0.7%)	**1.38 (0.31, 6.13)**	0.67	1.33 (0.30, 5.88)	0.71
Maternal anaemia (third trimester or delivery) - any	788/1475 (53.4%)	809/1485 (54.5%)	**0.98 (0.92, 1.04)**	0.45	0.98 (0.92, 1.04)	0.48
Maternal anaemia (third trimester or delivery) - mod severe	138/1475 (9.4%)	145/1485 (9.8%)	**0.94 (0.76, 1.17)**	0.60	0.94 (0.75, 1.17)	0.57
Maternal anaemia (third trimester or delivery) - severe	17/1475 (1.2%)	13/1485 (0.9%)	**1.30 (0.63, 2.65)**	0.48	1.27 (0.62, 2.59)	0.52

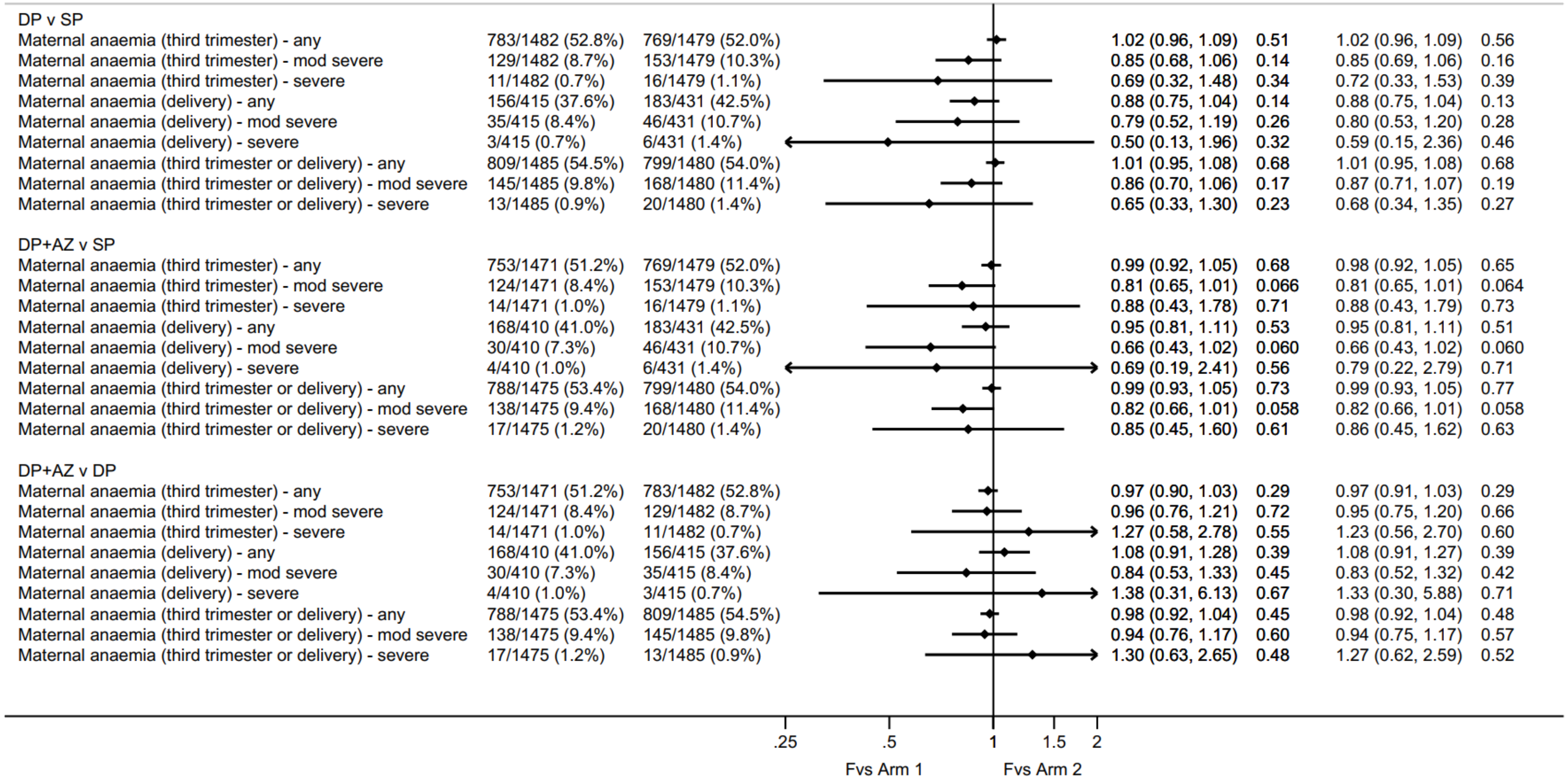

The crude RR values were obtained from log-binomial regressions with the stratification factors site and gravidity (paucigravidae and multigravidae) included as covariates. The adjusted RR values were adjusted for site, gravidity, and six other prespecified covariates. IPTp= intermittent preventive treatment in pregnancy. ITT=intention to treat. RR=risk ratio. CI=confidence interval, DP=dihydroartemisinin-piperaquine. AZ=azithromycin. SP=sulfadoxine-pyrimethamine. Any=Hb<11 g/dL. mod severe=Hb<9 g/dL. severe=Hb<7 g/dL. Fvs=favours.

Figure S10: Efficacy of IPTp-DP and IPTp-DPAZ versus IPTp-SP on Sexually transmitted infections and reproductive tract infections (modified ITT population)

Endpoints	Arm 1, n/N(%)	Arm 2, n/N(%)	Crude RR (95% CI)	P-value	Adjusted RR (95% CI)	P-value
DP v SP						
Any STI/RTI*	172/606 (28.4%)	151/596 (25.3%)	1.12 (0.93, 1.34)	0.23	1.10 (0.92, 1.32)	0.30
Chlamydia trachomatis	40/416 (9.6%)	10/410 (2.4%)	4.11 (2.09, 8.08)	<0.0001	4.17 (2.12, 8.19)	<0.0001
Neisseria gonorrhoea	8/416 (1.9%)	6/410 (1.5%)	1.35 (0.48, 3.84)	0.57	1.27 (0.45, 3.59)	0.66
Treponema pallidum	0/249 (0.0%)	1/262 (0.4%)	NA		NA	
Trichomonas vaginalis	66/416 (15.9%)	77/410 (18.8%)	0.84 (0.62, 1.13)	0.26	0.84 (0.62, 1.12)	0.23
Bacterial vaginosis	90/414 (21.7%)	75/413 (18.2%)	1.19 (0.91, 1.56)	0.21	1.20 (0.91, 1.57)	0.20
DP+AZ v SP						
Any STI/RTI*	155/602 (25.7%)	151/596 (25.3%)	1.01 (0.83, 1.21)	0.95	0.99 (0.82, 1.20)	0.94
Chlamydia trachomatis	30/421 (7.1%)	10/410 (2.4%)	2.90 (1.44, 5.85)	0.0028	2.86 (1.42, 5.76)	0.0033
Neisseria gonorrhoea	9/421 (2.1%)	6/410 (1.5%)	1.49 (0.54, 4.11)	0.45	1.34 (0.48, 3.70)	0.57
Treponema pallidum	3/255 (1.2%)	1/262 (0.4%)	2.66 (0.28, 25.27)	0.39	2.34 (0.34, 15.98)	0.39
Trichomonas vaginalis	63/421 (15.0%)	77/410 (18.8%)	0.79 (0.59, 1.07)	0.14	0.78 (0.58, 1.06)	0.11
Bacterial vaginosis	75/424 (17.7%)	75/413 (18.2%)	0.97 (0.73, 1.30)	0.85	0.97 (0.73, 1.30)	0.86
DP+AZ v DP						
Any STI/RTI*	155/602 (25.7%)	172/606 (28.4%)	0.90 (0.75, 1.08)	0.25	0.90 (0.76, 1.08)	0.26
Chlamydia trachomatis	30/421 (7.1%)	40/416 (9.6%)	0.71 (0.45, 1.11)	0.13	0.69 (0.44, 1.08)	0.10
Neisseria gonorrhoea	9/421 (2.1%)	8/416 (1.9%)	1.10 (0.43, 2.80)	0.84	1.06 (0.41, 2.69)	0.91
Treponema pallidum	3/255 (1.2%)	0/249 (0.0%)	NA		NA	
Trichomonas vaginalis	63/421 (15.0%)	66/416 (15.9%)	0.94 (0.69, 1.30)	0.72	0.94 (0.68, 1.28)	0.68
Bacterial vaginosis	75/424 (17.7%)	90/414 (21.7%)	0.82 (0.62, 1.07)	0.15	0.81 (0.62, 1.07)	0.14

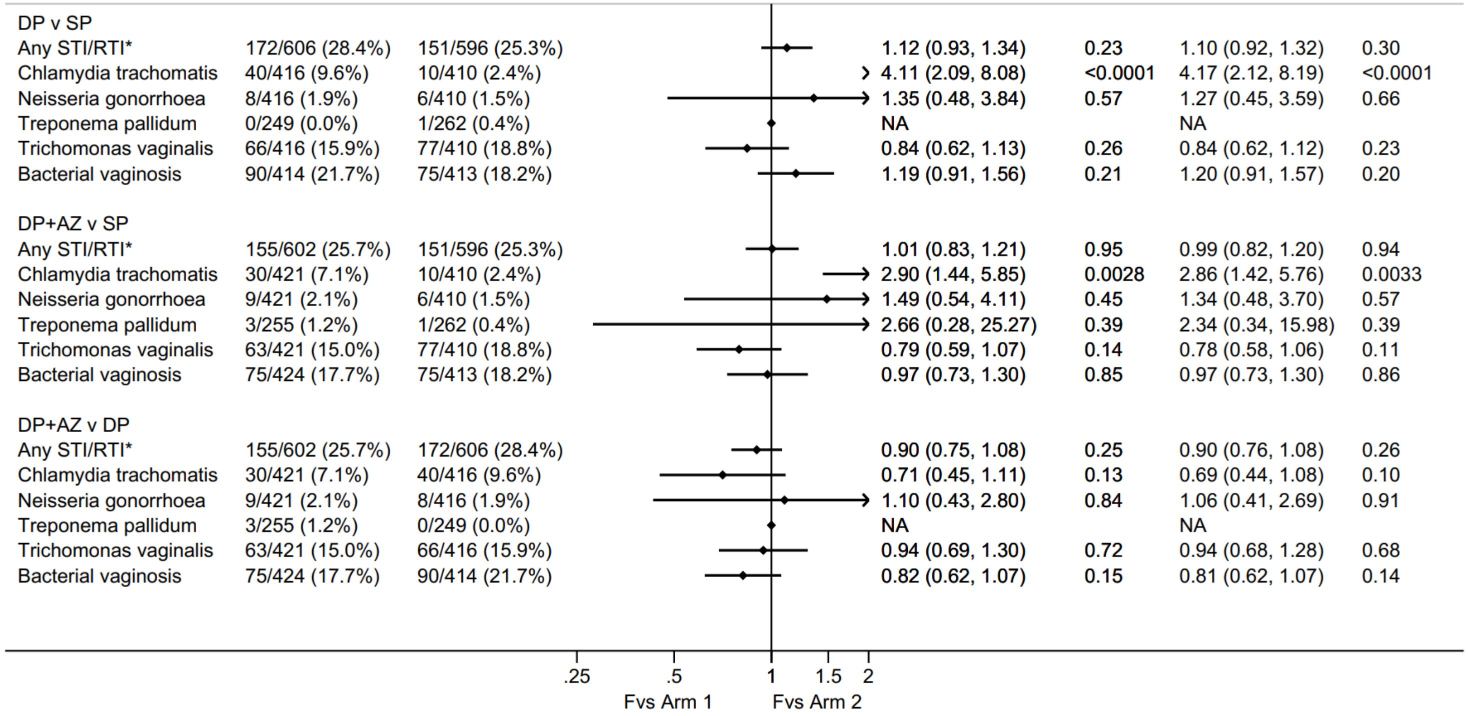

Estimates are based on samples collected after enrolment (mostly between weeks 32-35 gestation) and confirmed by laboratory diagnosis. The crude RR values were obtained from log-binomial regressions with the stratification factors site and gravidity (paucigravidae and multigravidae) included as covariates. The adjusted RR values were adjusted for site, gravidity, and six other prespecified covariates. IPTp= intermittent preventive treatment in pregnancy. ITT=intention to treat. RR=risk ratio. CI=confidence interval, DP=dihydroartemisinin-piperaquine. AZ=azithromycin. SP=sulfadoxine-pyrimethamine. STI=sexually transmitted infection. RTI=reproductive tract infection. Fvs=favours. *Any STI/RTI reflects any of the four sexually transmitted infections listed or the presence of bacterial vaginosis

IPTp with dihydroartemisinin-piperaquine and azithromycin for malaria, sexually transmitted and reproductive tract infections in pregnancy in high sulphadoxine-pyrimethamine resistance areas in Kenya, Malawi and Tanzania: an international multi-centre 3-arm placebo-controlled trial

Short Title: Improving PRegnancy Outcomes with intermittent PReVEntive treatment in Africa (TRIA-2015-1076-IMPROVE)

Trial Identifiers:

Kenya SERU	Malawi COMREC	Tanzania NatHREC	UK LSTM REC
75-3421	P.02/17/2110	NIMR/HQ/R.8a/Vol.IX/2533	16.049
**KPPB**	**PMPB**	**TFDA**	**Clinicaltrials.gov**
ECCT/17/08/02	[#####]	0017/CTR/0003/01	NCT03208179

Chief Investigator: *Prof Feiko ter Kuile*, MD, PhD, LSTM, Address: Liverpool School of Tropical Medicine, Pembroke Place, Liverpool L3 5QA, UK, Mobile: +44 (0)7846 377 369 (UK), +254 (0)708 739 228 (Kenya); Email: feiko.terkuile@lstmed.ac.uk

1.1.1.1 Co-Principal Investigators:

Malawi: *Dr Mwayiwawo Madanitsa*, MD, PhD, College of Medicine, University of Malawi, Private Bag 360, Chichiri, Blantyre 3, Mobile: +265(0) 999 616 537; E-mail: mmadanitsa@medcol.mw , mwayi.madanitsa@gmail.com

Kenya: *Dr Simon Kariuki*, PhD, KEMRI Centre for Global Health Research (CGHR) and CDC Collaboration, P.O. Box 1578, Kisumu 40100. Mobile: + 254 725 389 246; E-mail: SKariuki@kemricdc.org

Tanzania: a) *Prof John Lusingu*, MD, PhD, National Institute for Medical Research (NIMR), Tanga Medical Research Centre, P.O.Box 5004, Mobile: +255 787 679 515; E-mail: jpalusingu@gmail.com; b) *Prof Frank Mosha*, PhD, KCMC, Address: Kilimanjaro Christian Medical University College, P.O.Box 2240, Mobile:+255 745 102 523 & 255 784 317 316; E-mail: fwmosha@gmail.com

Co-investigators: See page 7

Funder: Joint Global Health Trials Scheme, Medical Research Council/Wellcome Trust/DFID, UK; and the European and Developing Countries Clinical Trials Partnership (EDCTP)

Sponsor: Liverpool School of Tropical Medicine (LSTM); Pembroke Place, Liverpool L3 5QA, UK, Phone: +44 0151 7053794; Email: lstmgov@lstmed.ac.uk

Revision chronology:

Version and date of protocol	Details of Changes	Authors (see also page 7)	Signature Chief Investigator
v1.1-01May17	Original	MM, FtK	Removed in online version
v2.1-31Aug17	1st amendment (see page 1)	MM, FtK	Removed in online version
V2.2-28Nov17	1st amendment (see page 2)	MM, FtK	Removed in online version

Confidentiality Statement: This document contains confidential information that must not be disclosed to anyone other than the sponsor, the investigator team, host institution, relevant ethics committee and regulatory authorities

DESCRIPTION OF AMENDMENTS

DETAILS OF KEY CHANGES TO PROTOCOL VERSION

V2.1-31AUG17

- Protocol v2.1-31Aug17 supersedes the original protocol v1.1-01May17
- Administrative updates: Addition of Tanzania NatHREC, KPPB and TFDA, and clinicaltrials.gov numbers (pg1 & pg7); Collaborators: Dr Eva Maria Hodel replaces Dr Christine Bachman (2.2.4 Collaborators, pg7)
- Removal of economic components from summaries (2.3.3 Short protocol summary, pg12; 2.3.4 Long protocol summary, pg13)
- Replacement of neonatal plethysmography with near-infrared spectroscopy and deuterium analysis (Table 1 Summary Table of Study Design and Schedule of Assessment, pg15; 7.5.4.4 Examination of the baby, pg36)
- Additional description of whole blood heel prick sampling for neonatal malaria diagnosis and immunity component (Table 1 Summary Table of Study Design and Schedule of Assessment, pg15; 7.5.5.1 Seven-day postnatal visit, pg36; 7.5.5.2 Six to eight -week postnatal visit, pg36)
- Addition of measurement of drug levels in cord and placental blood for safety (Table 1 Summary Table of Study Design and Schedule of Assessment, pg15; 7.5.4.3 Umbilical cord sampling pg36; 9.1.2 Plasma piperaquine levels, full blood count, renal function and liver function, pg41)
- Removal of collection of urine samples on day 28 post-partum (16.8.3 Antimicrobial activity and azithromycin resistance, pg86)
- Dose of azithromycin (and matching placebo): Changed from monthly courses to single course at enrolment; changed from 1.5g as 500mg for 3 days to 2g as 2x500 mg per day for 2 days. (2.3.4 Long protocol summary, pg13; 4.3 Hypotheses, pg22; 5 Aim & Objectives, pg22; 5.1 Primary objective, pg22; 5.2 Secondary objectives, pg22; 7.3.1.1 Name and description of study arms, pg27; Table 2 Dosing regimen per course, pg27)
- Change of name of supplier of DP from Sigma Tau to Alfasigma following the merger of companies (7.3.1.3.1 Dihydroartemisinin-piperaquine and matching placebo, pg28).
- Change that samples for STI/RTI and microbiome will be collected from all sites, not just Mangochi and Handeni (16.8.3 Antimicrobial activity and azithromycin resistance, pg86)
- Revision of sample size for antimicrobial components and serial ultrasound scans from 1,750 to 1,464 (Table 1 Summary Table of Study Design and Schedule of Assessment, pg15; 7.5.1.2 Baseline assessment, pg33; 16.8.3 Antimicrobial activity and azithromycin resistance, pg86; 7.5.2.1 Serial ultrasound scans, pg34))
- Revision if sample size macrolide resistance component from 819 to 1,032 (Table 1 Summary Table of Study Design and Schedule of Assessment, pg15; 7.5.1.2 Baseline assessment, pg33)
- Addition of text about blinded interim sample re-estimation (10.1.3.1, p45)
- Revision of time point serial ultrasound scans from 26-28 to 25-28 weeks (Table 1 Summary Table of Study Design and Schedule of Assessment, pg15; 7.5.2.1 Serial ultrasound scans, pg34)
- Addition of Tanzanian member Dr Ngasala, to DSMB: (16.2.3, pg73)
- Clarification that assays for protective maternal antibodies include antibodies against placental malaria (16.8.4 Maternal antibody, trans-placental antibody transfer and multi-pathogen neonatal cell mediated immune responses, pg88)
- Clarification that stool sample will also be used for evaluation of environmental enteric dysfunction and gut inflammation, and that blood sample will also be used for assessment of biomarkers of systemic inflammation (e.g. C-reactive protein) (16.8.3 Antimicrobial activity and azithromycin resistance, pg86; 16.8.6 Biomarkers of placental function and adverse birth outcomes, pg89)

V2.2-28NOV17

Main protocol

- Main protocol text in v2.2 is identical to v2.1. The only change is the version number. This was done to keep the version number of the main text document the same as the version number of the patient information sheet (PIS) and consent statements (CS) document.

Patient information sheet (pis) and consent statements (cs) (see separate document for pis/cs).

- PIS: Addition of the names of countries where some of the laboratory assays will be performed. 2.1.1. Participant Information Sheet for main trial (all women) (English), p10, PIS/CS document
- CS: Further clarification was added to the CS what the blood samples will be used for (2.12, Consent statement for screening and participation in the trial (all women) (English), p12, PIS/CS).
- CS: A sentence was added to the consent statement that the mother understands that their child will be measured and examined after birth (2.12, Consent statement for screening and participation in the trial (all women) (English), p12, PIS/CS document).

Table of content

1 Abbreviations 5
2 Administrative information 7
2.1 Title 7
2.2 Investigators and collaborators 7
2.3 Trial registration and protocol summaries 8
3 Introduction 18
3.1 The burden and control of malaria in pregnancy 18
3.2 SP resistance and waning effectiveness of IPTP-SP 18
3.3 Alternative drugs or strategies 18
3.4 Recent exploratory trials of IPTp with dihydroartemisinin-piperaquine 18
3.5 Azithromycin 19
4 Justification for the study 20
4.1 Why is this study needed now? 20
4.2 Other relevant research ongoing elsewhere 21
4.3 Hypotheses 22
5 Aim & Objectives 22
5.1 Primary objective 22
5.2 Secondary objectives 22
6 Trial Design & design considerations 23
6.1 Overview of design 23
6.2 Design considerations 23
7 Methods: Participants, interventions, and outcomes 25
7.1 Study setting 25
7.2 Eligibility criteria 26
7.3 Interventions 27
7.4 Outcomes 32
7.5 Participants timeline 33
7.6 Sample size 37
7.7 Recruitment and retention 39
8 Methods: Assignment of interventions 39
8.1 Allocation 39
8.2 Blinding and unblinding 39
9 Methods Data collection, management and analysis 40
9.1 Procedures for assessing efficacy and safety parameters 40
9.2 Data collection methods & storage 42
9.3 Statistical methods 43
10 Methods: Monitoring 44
10.1 Data monitoring and trial oversight committees 44
10.2 Safety Monitoring and reporting 46
10.3 Trial monitoring and auditing 50
10.4 Other quality control measures 50
11 Ethics and dissemination 51
11.1 Declaration of Helsinki 51
11.2 Research ethics and regulatory approval 51
11.3 Informed consent procedures 52

11.4 Protection of privacy and confidentiality ... 52
11.5 Declaration of interest ... 53
11.6 Access to source data/documents ... 53
11.7 Risks and benefits ... 53
11.8 Ancillary and post-trial care ... 55
11.9 Expenses reimbursement and incentives ... 56
11.10 Dissemination and application of the results ... 56

12 Timeframe and duration of the study ... 59
12.1 Timeline ... 59

13 References ... 61

14 Financial aspects and conflict of interest ... 68
14.1 Funding of the trial ... 68
14.2 Provision of study drugs ... 68

15 Budget & budget Justification ... 68

16 Appendices ... 69
16.1 Appendix I. Role of Investigators and collaborators ... 69
16.2 Appendix II. Terms of reference oversight committees ... 71
16.3 Appendix III. Declaration of Helsinki ... 74
16.4 Appendix IV. Budget and budget justification ... 77
16.5 Appendix V. SPIRIT 2013 checklist clinical trial protocol ... 78
16.6 Appendix VI. Participant information sheets and informed consent statements ... 83
16.7 Appendix VII. Description of other clinical and laboratory methods ... 84
16.8 Participants timeline ... **Error! Bookmark not defined.**
16.9 Appendix VIII. Product characteristics ... 92
16.10 Appendix IX. Questionnaires ... 110

1 ABBREVIATIONS

95% CI	95 percent confidence interval
ACT	artemisinin-based combination therapy
AE	adverse event
AIDS	acquired immunodeficiency syndrome
AL	artemether-lumefantrine
ANC	antenatal care
AUC	area under the curve
AZ	azithromycin
CDC	Centers for Disease Control and Prevention
CHW	community health worker
CHEW	community health extension worker
Cmax	maximum drug concentration
COM	College of Medicine, University of Malawi
CRF	case record form
CRO	contract research organization
DHA	dihydroartemisinin
DBS	dried blood spot (on filter paper)
DP	dihydroartemisinin-piperaquine
DMEC	data monitoring and ethics committee
DSMB	data safety and monitoring board
ECG	electrocardiogram
EDCTP	European and Developing Countries Clinical Trials
ERG	evidence review group from WHO
FDA	Food and Drug Administration
GCP	Good Clinical Practice
GLM	generalised linear model
GMP	Good Manufacturing Practice
Hb	haemoglobin
HIV	human immunodeficiency virus
IRB	institutional review board
IRS	indoor residual spraying
IPTp	intermittent preventive treatment in pregnancy
ITNs	insecticide treated nets
ITT	intention to treat
KCMC	Kilimanjaro Christian Medical Centre
KEMRI	Kenya Medical Research Institute
LBW	low birthweight
LLINS	long-lasting insecticide treated nets
LSHTM	London School of Hygiene and Tropical Medicine
LSTM	Liverpool School of Tropical Medicine
MDA	mass drug administration
MoH	Ministry of Health
MPAC	Malaria Policy Advisory Committee
MRC	Medical Research Council, UK
NathREC	National Health Research Ethics Committee, Tanzania
NIMR	National Institute for Medical Research, Tanzania

NMCP	National Malaria Control Programme
PCR	polymerase chain reaction
PT	preterm
RCT	randomized controlled trial
RDT	rapid diagnostic test
REC	research ethics committee
RTI	reproductive tract infection
SAE	serious adverse event
SGA	small for gestational age
SOP	standard operating procedure
SP	sulphadoxine-Pyrimethamine
STI	sexually transmitted infection
T1/2	plasma half-life
QTc	rate corrected time QT interval on electrocardiogram (ECG)
QTcB	rate corrected time QT interval on ECG using Bazett's correction
QTcF	rate corrected time QT interval on ECG using Fridericia's correction
TFDA	Tanzania Food and Drugs Authority
Tmax	time to maximum plasma concentration
TSC	trial steering committee
VHV	village health volunteer
VHW	village health worker
VHEV	village health extension volunteer
VHEW	village health extension worker
WHO	World Health Organization

2 ADMINISTRATIVE INFORMATION

2.1 TITLE

IPTp with dihydroartemisinin-piperaquine and azithromycin for malaria, sexually transmitted and reproductive tract infections in pregnancy in high sulphadoxine-pyrimethamine resistance areas in Kenya, Malawi and Tanzania: an international multi-centre 3-arm placebo-controlled trial
SHORT: **TRIA-2015-1076-IMPROVE**

2.2 INVESTIGATORS AND COLLABORATORS

2.2.1 Chief Investigator

Prof Feiko ter Kuile, MD, PhD[1,2]

2.2.2 Co-Principal investigators

Dr Mwayiwawo Madanitsa, MD, PhD[3] (Malawi)
Dr Simon Kariuki, PhD[2] (Kenya)
Prof John Lusingu, MD, PhD[5] (Tanzania)
Prof Franklin Mosha, PhD[6] (Tanzania)

2.2.3 Co-investigators

Prof Duolao Wang, PhD[1]
Prof Victor Mwapasa, MD, MPH, PhD[3]
Prof Kamija Phiri, MD, PhD[3]
Prof Ken Maleta, MD, PhD[3]
Dr Titus Kwambai, MD[2]
Dr Dianna Terlouw, MD, PhD[1,3,4]
Dr Daniel Minja, MSc, PhD[5]
Dr Jacklin Mosha, MD, MSc[6]
Dr Christentze Schmiegelow, MD, PhD[7]

2.2.4 Collaborators

Dr Aaron Samuels, MD[8]
Dr Meghna Desai, PhD[8]
Dr Julie Gutman, MD, MSc[8]
Dr Jenny Hill, PhD[1]
Dr Emily Adams, PhD[1]
Dr Jayne Webster, PhD[9]
Assist Prof Matthew Chico, MPH[9]
Prof Nigel Klein, MD, PhD[10]
Prof Thor Theander, MD, DSc[7]
Ass. Prof Dr Michael Alifrangis, PhD[7]
Ass. Prof Pascal Magnussen, MD[7]
Dr Ulla Ashorn, MD, PhD[11]
Prof Bjarne Robberstad, PhD[12]
Dr Ann Moormann, PhD[13]
Prof Kevin Kain, MD, PhD[14]
Prof Stephen Rogerson, MBBS, PhD[15]
Dr Holger W Unger, MD, PhD[15]
Dr Gonzalo Domingo, MD, PhD[16]
Dr David Bell, PhD[17]
Dr Eva Maria Hodel, PhD[1,4]
Dr Pascal Voiriot, MD[18]
Dr Iveth Gonzalez[19]

2.2.5 Institutions

1 Liverpool School of Tropical Medicine, UK
2 Kenya Medical Research Institute, Kenya
3 College of Medicine, University of Malawi, Malawi
4 MLW Clinical Research Programme, Malawi
5 National Institute for Medical Research, Tanzania
6 Kilimanjaro Christian Medical Centre, Tanzania
7 University of Copenhagen, Denmark
8 CDC, Atlanta, Georgia, USA
9 London School of Hygiene and Trop. Medicine, UK
10 University College London, UK
11 University of Tampere, Finland
12 University of Bergen, Norway
13 University of Massachusetts, Worcester, USA
14 University of Toronto, Canada
15 University of Melbourne, Melbourne, Australia
16 PATH, Seattle, Washington, USA
17 Intellectual Ventures Lab., Washington, USA
18 Cardiabase, Banook Group, Nancy, France
19 FIND, Geneva, Switzerland

2.3 TRIAL REGISTRATION AND PROTOCOL SUMMARIES

2.3.1 Trial Registration data

Data Category	Information
Primary registry and trial identifying number	Clinicaltrials.gov: NCT03208179
Date of registration in primary registry	30 June 2017
Secondary identifying numbers	Kenya SERU 75-3421<br>Malawi COMREC P.02/17/2110<br>Tanzania NatHREC NIMR/HQ/R.8a/Vol.IX/2533<br>UK LSTM REC 16.049<br>KPPB ECCT/17/08/02<br>PMPB [#####]<br>TFDA 0017/CTR/0003/01
Source(s) of monetary or material support	Joint Global Health Trials Scheme, Medical Research Council/Wellcome Trust/DFID, UK; and the European and Developing Countries Clinical Trials Partnership (EDCTP)
Primary sponsor	Liverpool School of Tropical Medicine (LSTM); Pembroke Place, Liverpool L3 5QA, UK, Phone: +44 0151 7053794; Email: lstmgov@lstmed.ac.uk
Secondary sponsor(s)	NA
Contact for public queries	Prof Feiko ter Kuile, LSTM, Pembroke Place, Liverpool, L3 5QA, UK, E-mail: feiko.terkuile@lstmed.ac.uk
Contact for scientific queries	Prof Feiko ter Kuile, LSTM, Pembroke Place, Liverpool, L3 5QA, UK, E-mail: feiko.terkuile@lstmed.ac.uk
Public title	Improving PRegnancy Outcomes with intermittent PReVEntive treatment in Africa (TRIA-2015-1076-IMPROVE)
Scientific title	IPTp with dihydroartemisinin-piperaquine and azithromycin for malaria, sexually transmitted and reproductive tract infections in pregnancy in high sulphadoxine-pyrimethamine resistance areas in Kenya, Malawi and Tanzania: an international multi-centre 3-arm placebo-controlled trial
Countries of recruitment	Malawi; Kenya; Tanzania
Health condition(s) or problem(s) studied	Pregnancy; Malaria; Sexual and Reproductive Tract Infections
Intervention(s)	1. IPTp-DPAZ: Dihydroartemisinin-piperaquine [3 to 5 tablets of DP (tablets of 40 mg of dihydroartemisinin and 320 mg of piperaquine, based on bodyweight) daily for 3 days] + AZ tablet [2g over 2 days as 1g (2 tablets of 500 mg) per day]<br>2. IPTp-DP: Dihydroartemisinin-piperaquine [3 to 5 tablets of DP (tablets of 40 mg of dihydroartemisinin and 320 mg of piperaquine, based on bodyweight) daily for 3 days] + placebo AZ<br>3. IPTp-SP: stat course of 3 tablets of quality-assured SP (tablets of 500 mg of sulphadoxine and 25 mg of pyrimethamine)
Study type	Interventional<br>Allocation: randomised; intervention model: parallel assignment; arms: 3; allocation ratio: 1:1:1; stratified by site. Masking: Partially placebo controlled<br>Primary purpose: Treatment and prevention<br>Phase-III
Date first enrolment	[dd mmm yyyy]
Target sample size	4,680 women (1,560 per arm)
Recruitment status	Not yet recruiting

Data Category	Information
Primary Objective	To determine if monthly IPTp with DP, either alone or combined with a single course of AZ at enrolment, for the control of malaria and STIs/RTIs in pregnancy is safe and superior to monthly IPTp with SP for reducing adverse pregnancy outcomes
Key inclusion criteria	• Pregnant women between 16-28 weeks' gestation<br>• Viable singleton pregnancy<br>• Resident of the study area<br>• Willing to adhere to scheduled and unscheduled study visit procedures<br>• Willing to deliver in a study clinic or hospital<br>• Provide written informed consent
Exclusion criteria	• Multiple pregnancies (i.e. twin/triplets)<br>• HIV-positive<br>• Known heart ailment<br>• Severe malformations or non-viable pregnancy if observed by ultrasound<br>• History of receiving IPTp-SP during this current pregnancy<br>• Unable to give consent<br>• Known allergy or contraindication to any of the study drugs
Primary outcome(s)	'Adverse pregnancy outcome' defined as the composite of foetal loss (spontaneous abortion or stillbirth), or singleton live births born small-for-gestational age (SGA), or with low birthweight (LBW), or preterm (PT) (SGA-LBW-PT), or subsequent neonatal death by day 28.
Key secondary outcomes	
Efficacy outcomes	• Composite of foetal loss and neonatal mortality<br>• SGA-LBW-PT composite<br>• The individual components of the above composite newborn outcome both as categorical and continuous variables<br>• Neonatal length and stunting<br>• Clinical malaria during pregnancy<br>• Malaria infection during pregnancy detected by microscopy and PCR (not for point of care)<br>• Placental malaria detected by microscopy, by molecular methods or by histology (past and active infection)<br>• Individual components of the placental malaria composite<br>• Maternal nutritional status<br>• Maternal anaemia during pregnancy and delivery<br>• Congenital anaemia<br>• Congenital malaria infection
Safety outcomes	• QTc-prolongation<br>• Congenital malformations<br>• Maternal mortality<br>• Other SAEs and AEs
Tolerance	• History of vomiting study drug<br>• Dizziness<br>• Gastrointestinal complaints
Antimicrobial activity and resistance	• Molecular markers of drug resistance in *Plasmodium falciparum* infections during pregnancy and delivery<br>• Presence of STIs/RTIs prior to delivery (syphilis, gonorrhoea, *Chlamydia trachomatis*, *Trichomonas vaginalis,* and bacterial vaginosis)

Data Category	Information
	• Changes in macrolide resistance in Pneumococcus detected in maternal nasopharyngeal samples<br>• Changes in the colony composition of maternal vaginal microbiota, and intestinal microbiota of mother and infant.

2.3.2 Lay summary

Context of the research: Each year over 30 million pregnancies occur in malaria endemic areas of sub-Saharan Africa. Malaria in pregnancy (MiP) has devastating consequences for the mother and unborn child. The control of malaria in pregnancy in parts of East and southern Africa is under threat. Pregnant women are often infected with malaria without showing any outward signs or symptoms which, if left undetected and untreated, can cause anaemia and interfere with the development of the foetus leading to loss of the pregnancy, or premature birth and low birth weight, which in turn increases the risk of early infant death. The World Health Organisation (WHO) therefore recommends a preventive strategy called 'intermittent preventive treatment in pregnancy' (IPTp) in which mothers receive a single dose of 3 tablets of medication called sulphadoxine-pyrimethamine (SP) at each scheduled antenatal visit starting in the 2nd and 3rd trimester. However, the effectiveness of this strategy is being compromised due to high levels of resistance to SP in the malaria parasite population.

The recent search for safe, effective and well-tolerated alternatives drugs has proven elusive because most of the new candidates tested were not tolerated well enough to be used for preventive purposes. Other trials evaluating test and treat strategies have also proven disappointing. All hopes are now pinned on an antimalarial called dihydroartemisinin-piperaquine (DP), which is known to be safe in the 2nd and 3rd trimester of pregnancy and highly effective for treatment of clinical malaria. Two exploratory trials showed that DP, when taken as IPT by pregnant women, was well tolerated and much more effective than SP in preventing malaria. However, these two trials were not big enough to to evaluate the impact on the pregnancy outcome and the health of the newborn. WHO reviewed the evidence in July 2015 and concluded that DP is indeed a promising alternative to SP and recommended that a larger, confirmatory, trial is needed, before it can consider whether to recommend this drug as an alternative to SP in areas of high resistance.

Sexually transmitted and reproductive tract infections (STIs/RTIs) also cause poor birth outcomes and are highly prevalent in East and Southern Africa, and similar to malaria, remain mostly asymptomatic, and therefore undetected and untreated. We will therefore determine whether combining DP with azithromycin, a broad spectrum antibiotic active against STIs/RTIs, can further improve birth outcomes, potentially paving the way for integrated control strategies for malaria and curable sexually transmitted and reproductive tract co-infections.

Study aims and objectives: This multi-centre trial will enrol about 4,680 pregnant women in 10 hospitals in Kenya, Tanzania and Malawi and compare the safety, tolerance and beneficial effects of IPTp with DP alone, or combined with azithromycin, to the current strategy with sulphadoxine-pyrimethamine in reducing pregnancy loss, low birthweight, preterm birth and small-for-gestational-age babies, and early infant deaths.

Potential applications and benefits: After a decade of trials to find new prevention strategies for malaria in pregnancy, DP has been shortlisted as the only potential alternative to SP for IPTp, but evidence of its benefits on infant outcomes is needed. Combining antimalarial and antibiotic interventions will potentially pave the way for integrated control strategies for malaria and curable sexually transmitted and reproductive tract co-infections. A positive result may lead to a direct policy change by the WHO in countries experiencing high level of parasite resistance, including most countries in East and southern Africa, benefiting women at risk of malaria in these regions resulting in healthier pregnancies and healthier newborns.

2.3.3 Short protocol summary

Malaria in pregnancy has devastating consequences for mother and foetus. WHO recommends intermittent preventive treatment in pregnancy (IPTp) with sulphadoxine-pyrimethamine (SP) for asymptomatic women, but high-level SP resistance threatens its efficacy. Over the last decade, several IPTp trials showed that neither amodiaquine, mefloquine, nor chloroquine-azithromycin are suitable replacements for SP because of their poor tolerability as IPTp. Furthermore, intermittent screening for malaria and treatment with artemisinin-based combination therapies has recently shown to be non-superior to IPTp-SP even in areas with very high SP resistance. Thus, there is an ever urgent need to find alternative drugs for IPTp.

Dihydroartemisinin-piperaquine (DP) has the potential to replace SP for IPTp. Exploratory trials from Kenya and Uganda showed that IPTp-DP was more effective than SP in reducing malaria infection (Incidence Rate Ratio [IRR]=0.32) and clinical malaria (IRR=0.16), but these preliminary trials were not powered to assess birth outcomes. WHO, in July 2015, recommended that definitive multi-centre trials are needed before IPTp-DP can be considered for policy.

Sexually transmitted and reproductive tract infections (STIs/RTIs) also cause poor birth outcomes and are highly prevalent in East and Southern Africa, and similar to malaria, remain mostly asymptomatic, and therefore undetected and untreated. We will therefore determine whether combining DP with azithromycin, a broad spectrum antibiotic active against STIs/RTIs, can further improve birth outcomes, potentially paving the way for integrated control strategies for malaria and curable sexually transmitted and reproductive tract co-infections.

This is a multi-national, individually-randomized, 3-arm, partially-placebo controlled superiority trial comparing the efficacy, safety and tolerance of monthly IPTp-SP (control) versus monthly IPTp-DP, alone or combined with a single course of azithromycin at enrolment (1gr/daily for 2 days) to reduce the adverse effects of malaria and curable STIs/RTIs in 4,680 women in 10 sites in high SP resistance areas in Kenya, Malawi, and Tanzania. The study is powered (80%, alpha=0.05) to detect a 20% reduction (RR=0.80) in 'adverse pregnancy outcomes' (composite of foetal loss, small-for-gestational age, low birthweight, preterm, or neonatal death). The project includes cardiac monitoring for safety, assessment of antimalarial drug and macrolide resistance, nutritional outcomes and the impact of SP and AZ on vaginal and intestinal microbiota.

2.3.4 Long protocol summary

Title: IPTp with dihydroartemisinin-piperaquine and azithromycin for malaria, sexually transmitted and reproductive tract infections in pregnancy in high sulphadoxine-pyrimethamine resistance areas in Kenya, Malawi and Tanzania: an international multi-centre 3-arm placebo-controlled trial

Short Title: Improving PRegnancy Outcomes with intermittent PReVEntive treatment in Africa (TRIA-2015-1076-IMPROVE)

Background and rationale: Malaria in pregnancy has devastating consequences for the mother and foetus. The WHO recommends the provision of intermittent preventive treatment in pregnancy (IPTp) using sulphadoxine-pyrimethamine (SP) for asymptomatic pregnant women, but high-level SP resistance threatens the efficacy of the intervention. Over the last decade, several IPTp trials have shown that neither amodiaquine, mefloquine, nor chloroquine-azithromycin are suitable replacements for SP because of their poor tolerability as IPTp. Thus, there is an even more urgent need to find alternative drugs for IPTp. Dihydroartemisinin-piperaquine (DP) has the potential to replace SP for IPTp. Exploratory trials from Kenya and Uganda showed that IPTp-DP was more effective than SP in reducing malaria infection (Incidence Rate Ratio [IRR]=0.32) and clinical malaria (IRR=0.16), but both these trials were small and not powered to assess birth outcomes. In September 2015, WHO recommended that definitive multi-centre trials are needed before IPTp-DP can be considered for policy. Sexually transmitted and reproductive tract infections (STIs/RTIs) also cause poor birth outcomes and are highly prevalent in East and Southern Africa and, similar to malaria infections, remain mostly asymptomatic, and therefore undetected and untreated. We will therefore determine whether combining DP with azithromycin, a broad spectrum antibiotic active against STIs/RTIs, can further improve birth outcomes, potentially paving the way for integrated control strategies for malaria and curable sexually transmitted and reproductive tract co-infections.

Overall aim: To provide the WHO with definitive evidence to determine whether monthly IPTp with DP, alone or combined with a single course of AZ at enrolment, is a viable alternative to the current strategy of monthly IPTp with SP in order to improve the outcome of pregnancies in areas with high levels of parasite resistance to SP and moderate to high malaria transmission and prevalence of STIs/RTIs in East and Southern Africa.

Primary objective: To determine if monthly IPTp with DP, either alone or combined with a single course of AZ at enrolment, for the control of malaria and STIs/RTIs in pregnancy is safe and superior to monthly IPTp with SP for reducing adverse pregnancy outcomes.

Hypotheses:

- IPTp with DP is superior to IPTp with SP in preventing adverse pregnancy outcomes.
- The combination of DP with single 2 day course of AZ at first antenatal clinic visit further reduces adverse pregnancy outcomes compared to IPTp with DP alone.

Overview Study Design: An international, multi-centre, 3-arm, parallel, partially placebo-controlled, individually randomised, phase-3, superiority trial involving 4,680 (1,560 per arm) pregnant women in approximately 10 sites in areas of high malaria transmission and high SP resistance in western Kenya, northern-eastern Tanzania and southern Malawi. HIV-negative pregnant women (all gravidae) attending for antenatal care (ANC) between 16 and 28 weeks' gestation inclusive, assessed by ultrasound dating, will be eligible. Women will be seen monthly until delivery. Mothers and infants will be followed for 6 to 8 weeks post-partum. Permuted block randomisation, stratified by site and gravidity group, will be used. Allocation concealment will be ensured by using sequentially numbered, sealed, opaque envelopes.

The study will include nested cardiac safety monitoring in a sub group of women. Other components of evaluation of primary and secondary trial outcomes include molecular marker studies of antimalarial resistance, and antimicrobial activity of and resistance to azithromycin studies. We will also look at the impact on **TRANSPLACENTAL ANTIBODY TRANSFER AND MULTI-PATHOGEN NEONATAL CELL MEDIATED IMMUNE RESPONSES**, on maternal and neonatal nutritional outcomes, and at correlations between biomarkers of placental function and adverse birth outcomes.

Sites: The study will be conducted in approximately 10 sites in 3 countries, selected because they are situated in areas with high malaria transmission and high SP resistance.

Study Population: HIV-negative pregnant women (all gravidae) between 16 and 28 weeks' gestation, as assessed by ultrasound dating who have not yet started IPTp during the current pregnancy.

Study Interventions: 1. Standard 3-day course of DP (40/320mg) per day based on body weight (Eurartesim) given monthly, plus AZ, 2g as 2x500mg daily for 2 days at enrolment, followed by standard 3-day course of DP only, given monthly; 2. Standard 3-day course of DP (40/320mg) per day based on body weight (Eurartesim) given monthly, plus placebo AZ at enrolment; 3. Standard single-day stat course of quality-assured SP given monthly.

Outcome Measures: Primary efficacy outcome: 'Adverse pregnancy outcome' defined as the composite of foetal loss (spontaneous abortion or stillbirth), or singleton live births born small-for-gestational age (SGA), or with low birthweight (LBW), or preterm (PT) (SGA-LBW-PT), or subsequent neonatal death by day 28.

Follow-up procedures: Enrolment at first antenatal visit and follow-up at routine scheduled antenatal care visits monthly with administration of intervention at all visits.

Sample size: 4,680 women (1,560 per arm) , allowing for 1 interim analysis

Data Analysis: Log-binomial regression will be used to analyse the primary endpoint, controlled for site and gravidity [paucigravidae (primi- and secundigravidae combined) and multigravidae].

Partner institutions: KEMRI and CDC Collaboration, Kisumu, western Kenya; College of Medicine, University of Malawi, Malawi; MLW Clinical Research Programme, Malawi; National Institute for Medical Research, Tanzania; Kilimanjaro Christian Medical Centre, Tanzania; Liverpool School of Tropical Medicine (LSTM); London School of Hygiene and Tropical Medicine (LSHTM); US Centers for Disease Control and Prevention (CDC); University of Copenhagen; Denmark University College London; University of Tampere, Finland; University of Bergen, Norway; University of Massachusetts, Worcester, USA; University of Melbourne, Melbourne, Australia; University of Toronto, Canada.

Funding: Joint Global Health Trials Scheme, Medical Research Council/Wellcome Trust/DFID, UK; and the European and Developing Countries Clinical Trials Partnership (EDCTP).

SPONSOR: Liverpool School of Tropical Medicine (LSTM); Pembroke Place, Liverpool L3 5QA, UK, Phone: +44 0151 7053794; Email: lstmgov@lstmed.ac.uk.

Table 1: Summary Table of Study Design and Schedule of Assessment

Phase	Recruitment Phase	Enrolment OPD	Initial course of IPTp	Follow-up & subsequent treatment monthly IPTp courses			Delivery	1 week Postnatal	6-8 weeks Postnatal
Visit number<br>Visit description	#1<br>Screening[b]	#1<br>Enrolment & baseline	#1<br>IPTp, maternal & fetal outcomes	#2[a]	#3[a]	#4[a]	#5<br>Maternal and birth outcome assessment	#6<br>Maternal and neonatal follow-up	#7
**Recruitment**									
Pre-screening eligibility	X								
Prior consent discussion	X								
**Enrolment**									
Eligibility screen		X							
Informed consent		X							
Study code issued		X							
Randomisation		X							
Allocation		X							
**Interventions** [d]									
IPTp-DP+AZ			Day 0,1,2 DP+AZ	Day 0,1,2 DP	Day 0,1,2 DP	Day 0,1,2 DP			
IPTp-DP			Day 0,1,2 DP+AZPlac	Day 0,1,2 DP	Day 0,1,2 DP	Day 0,1,2 DP			
IPTp-SP			Day 0 SP	Day 0 SP	Day 0 SP	Day 0 SP			
**Assessments**<br>Clinical measures									
Copy Clinic/Lab data from ANC card and ANC and other clinic registers		X		X	X	X	X		
Study CRF		X		X	X	X	X	X	X
Physical Exam.		X		X	X	X	X	X	X
Maternal antropometrics[m]		X		X	X	X	X	X	X
Ultra Sound Scan[e]		X		X[e]	X[e]	X[e]			
Drug vomiting questionnaire AE[f]			X[f]	X[f]	X[f]	X[f]	X	X	X
Newborn anthropometrics[m]							X	X	X
Unscheduled sick-patient clinic visits[f]		Continuous passive surveillance (clinical malaria + other acute illnesses) (RDT/smear, Hb, DBS for parasite genetics) [f]							
Cardiac monitoring ECG[c]			X	X	X	X			
**Samples**									
Blood sample		VP [g]		VP [h]	VP [h]	VP [h]	VP[g] ; CB[i]	HP[i]	HP[i]
Blood sample ECG study[c,j]			VP [j]	VP [j]	VP [j]	VP [j]			

Vaginal swab[o]	X	X		
Stool[K]	X	X		X[k]
Placental samples[l]			X[l]	
Nasopharyngeal swabs[n]	X		X	X

Visit 1: Pre-screening interview, Screening, Consent, Enrolment, Randomisation, and Allocation. First IPTp treatment dose given under direct observation.
Visits 2 to 4: Monthly IPTp treatment and ANC follow-up visits.
Visit 5: Delivery visit. To be conducted as soon as possible after birth without interrupting the initiation of breastfeeding or any necessary care required to the mother or newborn. A surface examination shall be conducted to assess for external congenital abnormalities.
Visits 6 and 7: Routine postnatal care, physical examination and follow-up of the mother-newborn pair.

a. Visits spaced approximately one month apart. The max. number of scheduled visits depends on the gestational age at enrolment (estimated median of 3 to 5).
b. Pre-study screening can take place any time women start visiting the ANC; i.e. even prior to 16 weeks gestation, but the intervention can only start at 16 weeks.
c. Cardiac safety monitoring; One ECG prior to 1st dose of each course and one 4-6 hours after the 3rd dose of each course (subgroup 160 women, 80 in each DP arm).
d. The first dose (day-0) always given under direct observation and subsequent 2 doses are to be taken at home (day-1 and day-2).
e. Baseline ultra sound scans in all women to assess the gestational age. Additional ultrasound scans to determine foetal growth at scheduled visits occurring at the approximate gestational ages of 25-28 weeks and 32-35 weeks (subgroup only), or otherwise at the nearest scheduled monthly visit.
f. Vomiting and other adverse events (AEs) occurring within 30 minutes of the first dose will be assessed. Subsequent AEs will be captured though passive surveillance. Participants will be encouraged to report to the study clinic for any subsequent AEs that may arise after each study visit at any time before the next study visit.
g. Enrolment & delivery blood sample by venepuncture (VP) (up to 10 mL) or finger prick (~250µL): All women all sites: haemoglobin level, HIV test (unless known to be HIV+), syphilis, malaria smear, dried blood spots (DBS) for molecular malaria diagnostics (not for point of care, DBS for multi-pathogen antibody assays and drug resistance. Sub-group (venepuncture): Nutritional biomarker including leptin, adiponectin, aflatoxin-albumin adduct (AF-alb) levels; antibody and cell mediated immune responses to malaria and other pathogens; biomarkers of placental function, inflammation and gestational age; malaria diagnostics, and drug levels. Remaining red cell pellets will be stored.
h. Interim study visits blood sample by venepuncture (up to 10 mL) or finger prick (~250µL): Same assays as for baseline, with the exception of syphilis. HIV testing and nutritional biomarkers will be done once in third trimester only during one of the last scheduled visits prior to delivery.
i. Umbilical cord blood (CB) sample (7 mL in subgroup and 5 mL in all others): Haemoglobin level, malaria molecular diagnostics, antibody and cell mediated immune responses to malaria and other pathogens, and nutritional biomarkers including leptin, adiponectin and drug levels. Heel prick (HP) sample (~ 500 microL) in all babies with fever or other symptoms for malaria RDT, microscopy and dried blood spot, and in subgroup of asymptomatic infants for immunological assays.
j. Venepuncture 2 ml blood sample taken 4-6 hours after last dose of each course (cMax) for biochemistry and drug levels (subgroup of 160 women in ECG component)
k. Stool sample at enrolment and last scheduled antenatal ANC visit prior to delivery in subgroup (n~1,464). Postnatally, only new-born stool sample shall be collected.
l. Placental samples at delivery/termination of pregnancy: placental section for histopathology, amnion roll, placental impression smear, maternal placental blood for malaria diagnostics (not for point of care), drug levels , and cord blood (i.e. newborn's side) (see cord blood sample).
m. Maternal anthropometric measurements: Enrolment: height; All visits: Weight, and MUAC. Sub-group only: delivery, 1 and 6-8 postpartum: skinfold thicknesses (SFTs), hip and waist circumference, bioelectrical impedance analysis and deuterium analysis (FTIR).

Infant anthropometric measurements at birth, 1 and 6 weeks post-partum: weight and length, head, abdominal, mid-upper arm circumference. Subgroup only: skinfold thicknesses (e.g. triceps, biceps, subscapular, suprailiac, quadriceps), near-infrared spectroscopy and deuterium analysis (FTIR) .

n. Nasopharyngeal swabs for macrolide resistant Streptococcus: enrolment, around delivery and/or <=6-8 weeks) and at ~6 months or later (sub-group n~1,032 women)
o. Self-administered vaginal swab at enrolment & ~last scheduled antenatal ANC visit prior to delivery in subgroup (n~1,464) women for vaginal microbiome and STI/RTI

3 INTRODUCTION

3.1 THE BURDEN AND CONTROL OF MALARIA IN PREGNANCY

Pregnancy increases women's susceptibility to malaria infection, with primi- and secundigravidae at particularly high risk. In countries with stable transmission in sub-Saharan Africa, most malaria infections in pregnant women remain asymptomatic, yet are associated with foetal loss, maternal anaemia, and intrauterine growth retardation and preterm birth, which are risk factors for neonatal and infant death.[1]The World Health Organization (WHO) currently recommends a three-pronged approach for the control of malaria in pregnancy; case-management of symptomatic malaria; providing long lasting insecticide-treated bed nets (LLITNs) to reduce exposure to infective mosquito bites; and intermittent preventive treatment of malaria in pregnancy (IPTp) for prevention of malaria in HIV-negative pregnant women. IPTp consists of the administration of treatment doses of an efficacious antimalarial drug during the second and third trimesters of pregnancy at predefined intervals at least a month apart.

3.2 SP RESISTANCE AND WANING EFFECTIVENESS OF IPTP-SP

Sulphadoxine-pyrimethamine (SP) is the only antimalarial currently recommended by the WHO for IPTp. However, high-level parasite resistance to SP threatens the efficacy of the strategy. In East and Southern Africa, the effectiveness of IPTp-SP to clear peripheral parasitaemia and prevent low birthweight (LBW) decreases with increasing population prevalence of the *Plasmodium falciparum dihydropteroate synthase* (*Pfdhps*) *Pfdhps*-K540E mutation, which is a proxy of the quintuple *Pfdhfr/dhps* mutant.[2] Nevertheless, some beneficial effect on birthweight remains even in areas with high resistance (defined as >90% prevalence of *Pfdhps*-K540E).[2,3] However, IPTp-SP fails to inhibit parasite growth among women infected with 'sextuple' mutant parasites (*Pfdhps*-A581G in combination with the quintuple mutant),[3-6] defined as super-resistant parasites.[7] Evidence is emerging that the IPTp-SP strategy is compromised if this additional mutation is prevalent at >10%.[4,5,8,9] The continued use of SP in these highly resistant areas has been a topic of recent debate, reflecting the urgent need for an effective, safe and affordable alternative to SP.[10-14]

3.3 ALTERNATIVE DRUGS OR STRATEGIES

No suitable alternatives to SP for IPTp have been found despite a decade of intensive multicentre trials. These have shown that neither amodiaquine, alone or combined with SP,[15] mefloquine (15 mg/kg),[16-19] or the fixed-dose combination of chloroquine-azithromycin[20,21] are tolerated well enough to replace SP for IPTp in Africa. More recently, trials in Malawi[22] and Kenya[23], assessed intermittent screening and treatment in pregnancy (ISTp) as an alternative strategy to IPTp, consisting of intermittent rapid diagnostic testing (RDT) for malaria and treatment of RDT-positive cases with dihydroartemisinin-piperaquine (DP). The results from both trials were disappointing, showing no evidence that this screen-and-treat strategy is a suitable alternative to IPTp-SP in areas with high SP resistance and malaria transmission.[22,23]

3.4 RECENT EXPLORATORY TRIALS OF IPTP WITH DIHYDROARTEMISININ-PIPERAQUINE

Currently, the only promising candidate for IPTp as a replacement for SP is dihydroartemisinin-piperaquine (DP). As an artemisinin-based combination therapy (ACT), DP is currently recommended by WHO for the case-management of malaria in the 2nd and 3rd trimesters. It has been first-line

treatment for this indication in Indonesia for >8 years. A recent comparison of four different fixed-dose ACTs for the treatment of malaria in pregnancy showed that DP had the best efficacy and an acceptable safety profile, with an additional benefit of a longer post-treatment prophylactic effect, which supports its suitability as a candidate for IPTp in high transmission areas.[24,25]

Until recently, DP had not been evaluated for use as IPTp (i.e. repeated dosing). Our trial in Kenya (STOPMIP1), recently published in *The Lancet,*[23] was the first to compare IPTp-SP versus IPTp-DP (n~515/arm).[26] Results of a second, smaller IPTp-DP trial (n~100/arm) in Uganda were also recently published in the *New England Journal of Medicine*.[27] The results across these two trials consistently showed that, compared to IPTp-SP, 3 or 4 courses of IPTp-DP were well tolerated and associated with a 68% and 59% reduction in malaria infection at delivery in Kenya and Uganda, respectively, an 84% and 67% reduction in the incidence of clinical malaria during pregnancy, and a 22% and 13% lower risk of anaemia at delivery. The risk of foetal loss and early neonatal death were 61% to 47% lower in the IPTp-DP arm, but the impact on foetal growth and preterm birth were either modest (Uganda[27]) or absent (Kenya[23]).

3.5 AZITHROMYCIN

3.5.1 Azithromycin in pregnancy

Azithromycin (AZ) is a broad-spectrum macrolide antibiotic that can be administered safely in all trimesters of pregnancy and may protect against adverse birth outcomes, independently of the effect of IPTp-DP. A recent trial from Papua New Guinea showed that IPTp with SP plus AZ reduced both the incidence of LBW and preterm birth,[28] adding to evidence from two previous trials in Malawi, one of which also showed that the addition of AZ further improved birthweight,[29] whereas the other did not.[30] When administered as monotherapy, AZ is efficacious against *P. vivax* at relatively low doses of 1 to 2g,[31,32] but needs an efficacious and fast acting antimalarial partner drug against *P. falciparum*, and requires doses of 3g or more over 3 days to achieve its 'delayed death' effect.[33] Such quantities of AZ are not well tolerated nor suitable for IPTp as was shown with chloroquine-azithromycin.[20]

AZ, at doses between 1 and 2g, has excellent activity against curable sexually transmitted diseases and reproductive tract infections (STIs/RTIs), including syphilis, gonorrhoea, and chlamydia and, potentially, trichomoniasis, that are associated with pregnancy loss, intrauterine growth retardation, preterm birth and LBW.[34]A recent meta-analysis suggests that these curable STIs/RTIs are highly prevalent among women attending ANC in sub-Saharan Africa,[35] many of which remain asymptomatic similar to malaria, and thus undetected and untreated.[36] The addition of AZ to IPTp could be expected to mitigate a considerable proportion of this unattended burden of curable STIs/RTIs, potentially paving the way for adoption of control strategies for integrated malaria and curable infections in pregnancy that employ combination treatment to improve birth outcomes.[34] This may enhance the acceptability of IPTp, as suggested by a recent discrete choice experiment among pregnant women and health care providers in Tanzania, showing a preference for therapy that was protective against malaria and STIs/RTIs versus malaria alone (Chico, unpublished observations).

3.5.2 Macrolide resistance

A single dose of AZ (1g) clears ocular strains of chlamydia that cause trachoma. During the past two decades, over 500 million doses of AZ have been distributed in trachoma-endemic areas[37] as part of the WHO-led trachoma elimination programme.[38] Mass distribution has been shown also to reduce the incidence of respiratory infections,[39] diarrhoea,[40] and malaria.[41] These secondary benefits likely

underlie the statistically significant reductions in all-cause childhood mortality reported in Ethiopia following trachoma treatment campaigns where odds were cut by one-half (OR=0.51; 95% CI: 0.29-0.90) in a clinical trial[42] and 35% (OR=0.35; 95% CI: 0.17–0.74) in a cohort study.[43] However, there are concerns regarding clonal expansion of pneumococcal multi-locus sequence types following mass treatment in the same setting where four mass treatments of AZ were conducted.[44] Specifically, significant associations were reported between AZ dosing and *ermB* and *mefA/E* genetic resistant determinants responsible for the vast majority of pneumococcal macrolide resistance.

Whether this is of clinical consequence is unknown. Studies of mass treatment with follow-up periods of $\geq$6 months post AZ exposure have found that the prevalence of resistant isolates returns to pre-exposure levels.[45,46] Moreover, there is no evidence of noticeable increases in pneumococcal resistance over time in countries that have participated in mass campaigns.

4 JUSTIFICATION FOR THE STUDY

4.1 WHY IS THIS STUDY NEEDED NOW?

The results of the prior ISTp trials and exploratory trials of IPTp with DP were presented to WHO's Evidence Review Group (ERG) in July 2015 as part of WHO's policy making process to formulate recommendations to the Malaria Policy Advisory Committee (MPAC).WHO concluded that DP is a promising alternative to SP for IPTp and recommended that a larger confirmatory trial be conducted to provide definitive evidence of its efficacy, safety, operational feasibility and cost-effectiveness so that it can be considered by WHO for policy.[47] This trial aims to address the gaps regarding efficacy, safety and cost-effectiveness as outlined in the following paragraphs.

4.1.1 Confirmation of potential efficacy and safety

4.1.1.1 Efficacy

The previous trials in Kenya[23]and Uganda[27]are considered exploratory, as the total combined sample size was 541 completers in the IPTp-DP arms. Neither was powered to look at adverse birth outcomes for which this larger confirmatory trial is needed.

4.1.1.2 Safety

Treatment with DP has been shown to be safe in the 2nd and 3rd trimester of pregnancy.[23,24,27,48-53] A single course of DP is safe for use in the 2nd and 3rd trimesters, and is recommended by WHO for treatment of malaria in the 2nd and 3rd trimester and has been the first-line treatment for this indication in Papua, Indonesia, for over 10 years. The main safety concern is a dose-dependent QTc prolongation associated with piperaquine, similar to that observed with other antimalarials like chloroquine, but less severe than with halofantrine and quinine. Clinical data do not suggest any association with arrhythmias when used as a single three-day course for treatment.[54,55]The experience, however, with monthly dosing is more limited; approximately 1,000 women have received either 3-course or monthly IPTp-DP in Kenya,[56] Uganda,[57] and an ongoing IPTp-DP trial in Indonesia (STOPMIP-Indonesia[58]).

There has been no indication that IPTp-DP is associated with an increased risk of congenital malformation or pregnancy loss (the existing evidence in Africa suggest a 61% [95% CI 12-83] reduction in the risk of foetal loss compared to SP [fixed effect meta-analysis conducted for this application[56,57]). Because of its slow elimination, monthly doses of DP could result in dose accumulation of piperaquine. Nevertheless, there is no evidence to date that the risk of QTc

prolongation increases with each subsequent monthly course, likely because piperaquine-associated QTc prolongation is transient and lasts for a few days only. The magnitude of transient QTc prolongation remained constant and was similar after ≥12 monthly courses compared to a single course in a study of Ugandan children provided with 18 monthly courses of DP from 6 to 24 months of age.[59]

One further study in Africa assessed the cardiac safety of single course (rather than repeat course) of IPTp with DP in pregnancy (n=100) in north-eastern Tanzania (Matthew Chico et al, LSHTM; Clinicaltrials.gov NCT02909712). Cardiac monitoring and pharmacodynamic studies of the correlation with piperaquine drug levels and QTc prolongation with repeat doses are currently being assessed in 3 open label studies conducted by our study team in Indonesia[58] and in Malawi (about 35 women with monthly DP, PMI-funded, Julie Gutman, CDC, personal communications, Clinicaltrials.gov NCT03009526) and in Uganda (PROMOTE-II, 391 women with monthly DP, Clinicaltrials.gov NCT02793622,Prof Grant Dorsey, UCSF, personal communications).

4.1.2 Resilient protective effect of SP on birthweight: antimalarial or antimicrobial effect?

Several observational studies have shown that SP has a surprisingly resilient beneficial effect on birthweight,[2,23] even in areas where approximately 50% of paucigravidae had recurrent infections within 42 days of their first course of IPTp-SP.[2] It is possible that radical clearance of parasites may not be needed to reduce placental inflammation; suppression of parasite densities maybe sufficient. It could also reflect its broad antimicrobial activity conferring some protection against undetected bacterial infections, particularly gram-positive bacteria,[60]or due to an effect on the intestinal or vaginal microbiota. Antibiotics, in general, have growth promoting effect in children, possibly by alleviating chronic enteropathy[61] or altering metabolism.[62] In addition, changes in the vaginal microbiome could decrease the risk of ascending infection which would lead to preterm birth.[63]

4.2 OTHER RELEVANT RESEARCH ONGOING ELSEWHERE

A search of WHO's trial registry indicated no other IPTp-DP trials are ongoing in Africa. We are aware of two smaller trials comparing monthly IPTp-DP and IPTp-SP in a high SP resistance area in eastern Uganda, scheduled to start in 2016 (PROMOTE-II, 782 women [391/arm], Clinicaltrials.gov NCT02793622, Prof Grant Dorsey, UCSF, personal communications) and Malawi (PMI-funded, Julie Gutman, CDC, personal communications, Clinicaltrials.gov NCT03009526). These two trials will include a combined total of approximately 600 women with IPTp-DP, which will double the experience with IPTp-DP in Africa, but will be insufficient to address the impact on adverse birth outcomes required by WHO, and will not address the cost-effectiveness and feasibility of IPTp-DP. Our proposed study has been designed in discussion with these other teams to allow a prospective meta-analysis of individual participant data.

We are also aware of one other study in Africa looking at the cardiac safety of single course (rather than repeat course) of IPTp with DP in pregnancy (n=100) in north-eastern Tanzania (Matthew Chico *et al*, LSHTM; Clinicaltrials.gov NCT02909712). Lastly, our STOPMIP-Indonesia trial,[58]comparing IPTp-DP with screening strategies will add safety data from a further 700 women on monthly IPTp-DP, but the efficacy results cannot be extrapolated to Africa because the study is conducted in low transmission areas where *P. falciparum* and *P. vivax* co-exist, and the control arm does not involve IPTp-SP.

4.3 Hypotheses

We propose to conduct a 3-arm trial, comparing IPTp with SP, versus IPTp with DP alone, and IPTp with DP+AZ with the following hypotheses:

- IPTp with DP is superior to IPTp with SP in preventing adverse pregnancy outcomes.
- The combination of DP with single 2 day course of AZ at first antenatal clinic visit further reduces adverse pregnancy outcomes compared to IPTp with DP alone.

5 Aim & Objectives

The overall aim of the study is to provide the WHO with definitive evidence to determine whether monthly IPTp with DP, alone or combined with a single course of AZ at enrolment, is a viable alternative to the current strategy of monthly IPTp with SP in order to improve the outcome of pregnancies in areas with high levels of parasite resistance to SP and moderate to high malaria transmission and prevalence of STIs/RTIs in East and Southern Africa.

5.1 Primary objective

To determine if monthly IPTp with DP, either alone or combined with a single course of AZ at enrolment, for the control of malaria and STIs/RTIs in pregnancy is safe and superior to monthly IPTp with SP for reducing adverse pregnancy outcomes

5.2 Secondary objectives

1. To determine if monthly IPTp with DP plus a single course of AZ at enrolment, is superior to monthly IPTp with DP alone for reducing adverse pregnancy outcomes.
2. To determine the safety of monthly IPTp with DP by conducting nested cardiac monitoring study to specifically address whether previously documented transient QTc prolongation associated with DP increases in magnitude with subsequent courses as well as monitoring adverse drug reactions and adverse/severe adverse events.
3. To conduct, on completion of the trial, a prospective meta-analysis combining the evidence from this and previous trials to provide (with≥80% power) definitive evidence on whether IPTp with DP is superior to the existing strategy of IPTp with SP for controlling malaria and reducing adverse pregnancy outcomes in areas with intense year-round malaria transmission and high SP resistance.
4. To determine if the level of SP drug resistance, assessed by molecular markers, affects the potential impact of IPTp-DP or IPTp with DP+AZ relative to IPTp-SP.
5. To determine the efficacy of IPTp with DP+AZ on curable sexually-transmitted and reproductive -tract infections (STIs/RTIs) relative to IPTp with SP and DP alone.
6. To determine the effect of SP and AZ on the intestinal and vaginal microbiomes of mothers, and the intestinal microbiomes of neonates relative to DP alone.
7. To determine the effect of AZ on the prevalence of macrolide resistance.
8. To build trial research capacity in Kenya, Malawi and Tanzania.

6 TRIAL DESIGN & DESIGN CONSIDERATIONS

6.1 OVERVIEW OF DESIGN

An international, multi-centre, 3-arm, parallel, partially placebo-controlled, individually randomised, phase-3, superiority trial involving 4,680 (1,560 per arm) pregnant women in approximately 10 sites in areas of high malaria transmission and high SP resistance in western Kenya, northern-eastern Tanzania and southern Malawi. HIV-negative pregnant women (all gravidae) attending for antenatal care (ANC) between 16 and 28 weeks' gestation inclusive, assessed by ultrasound dating, will be eligible. Women will be seen monthly until delivery. Mothers and infants will be followed for 6 to 8 weeks post-partum.

The trial is designed to provide definitive evidence required by WHO to determine if IPTp-DP can be recommended in high SP resistance areas. It is powered at 80% to detect a 20% relative reduction (Risk Ratio [RR] = 0.80) in adverse pregnancy outcomes from 21.3% in the IPTp-SP arm to 17.0% in any of the two new intervention arms (alpha=0.05), and is also powered at 80% to detect a further 22.8% relative reduction (RR=0.772) to 13.1% in the IPTp-DP+AZ arm relative to IPTp-DP alone. The trial will take 3 years to complete, including 9 months for study preparation, 24 months for field work, and 3 months for laboratory and data analysis. The trial will also contain nested cardiac monitoring studies.

6.2 DESIGN CONSIDERATIONS

6.2.1 Design as a superiority trial

Sulphadoxine-pyrimethamine is cheap, well tolerated, safe and widely available. In contrast, IPTp-DP with or without AZ will be more expensive and confer a greater pill burden during implementation than IPTp. Therefore, the health benefits need to be sufficiently greater than observed with SP to outweigh the reduced practicality of a 3,- vs 1-day regimen and the additional cost associated with the drug and implementing a new policy.

6.2.2 Why pregnant women of any gravidity?

We anticipate most or all of the impact on birth outcomes and morbidity to be in women in their first and second pregnancies based on systematic review of previous trials with IPTp-SP and ITNs.[64,65] However, the impact on placental malaria infection is anticipated to occur across all pregnant women, including those in their third and subsequent pregnancies. Placental malaria and malaria at term are now recognised to be important endpoints in themselves as they have been found to be risk factors for malaria, anaemia and other infections in infants up to the age of two years, independent of the effect of malaria on gestational age low birth weight. The effect appears to be particularly evident in multigravidae. Furthermore, IPTp is currently recommended for and provided to women of all gravidities, and it is anticipated that any alternative strategies to IPTp may also be provided to all pregnant women, if any potential benefit is identified.

6.2.3 Why include women up to 28 weeks' gestation?

The study staff will enrol women as early as possible in the 2nd trimester and up to 28 weeks gestation for those that start attending ANC late in the second trimester. The benefit of IPTp in these late attenders is likely to be less than women who come earlier and who are thus eligible to receive multiple monthly courses of IPTp. There is increasing evidence that preventing malaria early in pregnancy, including the first trimester is likely to have major health benefits for the mother and

developing foetus. However, the average gestational age at antenatal booking is approximately 22 weeks from last menstrual period, with some women attending ANC only later in the third trimester. A study that would restrict enrolment to the early second trimester would thus not be representative of the antenatal study population.

6.2.4 Why exclude women with HIV co-infection?

To address a similar research question in HIV-infected women will require a standalone trial, because HIV-infected women receive anti-retroviral therapy, and do not receive IPTp with SP but daily cotrimoxazole, which has both antimicrobial and antimalarial properties. Since both cotrimoxazole and SP are sulpha-based drugs, their co-administration is contraindicated because of the enhanced risk of severe cutaneous reactions.[66]

6.2.5 Why use a composite primary outcome?

A composite outcome is used rather than a single outcome, such as LBW, because 1) it provides a single summary measure of all clinically relevant outcomes that can drive policy decisions; 2) malaria, STIs and RTIs affects each of these individual outcomes which interact with each other to affect the health and survival of the infant; finally, 3) it allows for a more time and cost efficient trial by requiring a smaller sample size. Two exploratory trials comparing IPTp-DP vs IPTp-SP in high SP-resistance areas in Kenya[23] and Uganda[27] showed that, relative to IPTp-SP, IPTp-DP was associated with a 15% (95% CI: -21 to 40) and 22% (95% CI: -25 to 52) reduction in this primary endpoint, respectively. The pooled effect size calculated using fixed effects meta-analyses, calculated for this protocol, is 17% (95% CI: -10 to 37).

6.2.6 Why not use a malaria-specific primary outcome?

Use of clinical malaria or malaria infection during pregnancy as the primary outcome would require a smaller study. However, it is already known that DP is superior to SP in preventing malaria in pregnancy,[23,27] but neither of the previous trials were powered to quantify the impact on more tangible outcomes required by WHO that drive public health decision making, such as adverse pregnancy outcomes.

6.2.7 Rationale for testing parasite genetics and resistance-associate genes

This study also includes genetic examination of the *P. falciparum* parasites infecting the enrolled patients. Parasite genetic material will be obtained from the same blood sample as those required for the clinical follow-up. Whole blood will be stored on filter paper blood spots for later processing. Genetic evaluation of the parasite can provide important insight into the degree of infection and importantly monitor for developing resistance due to ongoing drug pressure. First by examining the parasite genetic diversity, comparing baseline parasite composition to treatment and post-treatment complexity of infection (number of strains). The complexity of infection as well as the presence or absence of infection provides a gross measure for the efficacy of the IPTp regimen in reducing the parasite burden. Secondly, we will focus our genetic studies to monitor for signs of drug resistance. Artemisinin-based combination therapies have demonstrable parasite resistance in South East Asia and there is great concern that resistance will spread to or develop within Africa.[67] Importantly, we will monitor for signs that drug pressure is selecting for resistant parasite strains. Genes associated with parasite drug resistance will be sequenced including those implicated in artemisinin resistance (kelch K13), piperaquine (plasmepsin 2,3), and lumefantrine (pfmdr1). Compared to baseline parasites we will determine if any mutations within these known drug resistance candidates have an increased frequency within the treated individuals. For a description of the laboratory techniques see section Appendix VII. Description of other clinical and laboratory methods, page 84.

7 METHODS: PARTICIPANTS, INTERVENTIONS, AND OUTCOMES

7.1 STUDY SETTING

In order to have a high likelihood of showing a differential impact relative to IPTp-SP, the trial will be conducted in areas with moderate to intense malaria transmission and high to very high SP resistance in western Kenya, north-eastern Tanzania and southern Malawi. The National Malaria Control Programmes (NMCP) in these 3 countries regard SP resistance as a major threat to the existing IPTp-SP policy.

The prevalence of placental infection detected by PCR and histopathology among paucigravidae in the SP arms of our recent trials was 50% in Kenya[23] and 57% in Malawi.[68] The study sites in north-eastern Tanzania have seen a dramatic increase in malaria in the last 2 years. In the ongoing pregnancy cohort studies, at least 30% of women have evidence of patent malaria infections detected by malaria rapid diagnostic tests, and the overall risk will be similar to that in Kenya and Malawi once sub-patent infections are taken into account. These high rates in all 3 countries suggest both a failure of SP to clear or prevent infections and high transmission intensity. In the sites in Kenya and Malawi, the prevalence of the quintuple mutant is >98% and approximately 6-9% of women in 2013 were infected with parasites carrying the sextuple *Pfdhfr/dhps* mutation (*Pfdhps*-A581G in combination with the quintuple mutant), and in north-eastern Tanzania, the prevalence of the sextuple mutant parasite is as high as 40-50%. [5,69]

The study will be conducted in antenatal clinics and delivery units of Government or private (e.g. mission) hospitals and clinics with at least 30 new antenatal attendants per month in areas with year-round malaria transmission. The final choice of sites will depend on the available infrastructure and absence of planned malaria control interventions in the catchment area that might have a major impact on the malaria transmission intensity during the study period (e.g. planned mass campaigns to reduce malaria transmission such as indoor residual spraying [IRS]).

7.1.1 Kenya

The study is part of a multi-year collaboration between LSTM and the Kenya Medical Research Institute (KEMRI), Kisumu, western Kenya, and the US Centers for Diseases Control and Prevention (CDC)-Kenya, and will be conducted in approximately 3 to 5 Hospitals and satellite ANC clinics in the counties of the previous Western and Nyanza Provinces; including, but not limited to Busia, Siaya, Ahero, Homa Bay and Migori counties in high malaria transmission areas in western Kenya.

7.1.1.1 Suggested primary hospitals

Migori County Referral Hospital, Homa Bay County Referral Hospital, Rongo sub-county hospital, Ahero sub-county hospital, and Rabuor sub-county hospital and the corresponding satellite clinics that refer patients to these hospitals.

7.1.1.2 Backup and expansion hospitals and clinics

St Mary's Hospital, Mumias, Mukumu Mission Hospital**, KAKAMEGA,** Kisumu District Hospital, Busia District Hospital, Nyamira District Hospital, Bondo District Hospital, Siaya County Referral Hospital, Kisumu County Referral Hospital, Jaramogi Oginga Odinga Teaching and Referral Hospital, Rachounyo sub county hospital, Kendu Bay sub-county hospital and Kendu Adventist hospital. This list is not exclusive **AND** another hospital may be considered for inclusion in western Kenya if they fulfil the entry criteria for size and malaria transmission and control.

7.1.2 Malawi
The sites in southern Malawi will include 3 to 5 district hospitals and satellite ANC clinics where several pregnancy studies have been conducted. The Research Support Centre from the College of Medicine (CoM), University of Malawi, and supported by Malawi-Liverpool-Wellcome Trust Clinical Research-Programme linked with the CoM in Blantyre will be the coordination institutions providing administrative and laboratory support.

7.1.2.1 Suggested primary hospitals
Mangochi District Hospital, Zomba Central Hospital, Chikwawa District Hospital, Madziabango and Mpemba Health Centers and, the corresponding satellite clinics that refer patients to these facilities.

7.1.2.2 Backup and expansion hospitals and clinics
These included, but are not limited to Machinga District Hospital, Lungwena Health Center, Makanjira Health Center, Monkeybay Community Hospital, Domasi Rural Hospital, Mfera Health Center, Chapananga Health Center, Balaka District Hospital, Chiradzulu District Hospital, Mwanza District Hospital and Ntcheu District Hospital. This list is not exclusive as other hospitals may be considered for inclusion in southern Malawi if they fulfil the entry criteria for size and malaria transmission and control.

7.1.3 Tanzania
The sites in north-eastern Tanzania will include 3 to 5 district hospitals and satellite ANC clinics where several pregnancy studies have been conducted. The sites will be coordinated by the National Institute for Medical Research, in Korogwe, Tanga, and the other sites by the Kilimanjaro Christian Medical Centre (KCMC) in Moshi*.* The study will be conducted in Korogwe District Hospital (Magunga), Muheza Muheza Designated District Hospital (Teule) and Handeni District hospital. We will also include the following health centres in Muheza: Mkuzi and Ubwari; and dispensaries: Songa Batini, Kwafungo, Mbambara, Kwabada, Mtindiro, Mindu, Kilulu, Nkumba, Kicheba, Misozwe, Kwatango, Bwembwera, Mkanyageni, Kibaoni, Umba, Mhamba, Potwe, Zeneti. In Handeni district these include the health centres: Kabuku, Kideleko, Segera, Kwamatuku, and Bondo, and the dispensaries Segera, Masatu, Kwamatuku, and Komsala dispensaries; In Korogwe we will include all health centres and the following dispensaries: Majengo, Mtonga , Mgombezi , Msambiazi , Lewa , Makuyuni , Chekelei , Mbaghai , Hale , Kwakombo , Kwamsisi , Mnyuzi , Magazini , Lwengera , Kerenge , Kwamndolwa. If so required, we will also collaborate with the other health centres and dispensaries in neighbouring districts to ensure continuum of care and referrals if women move to these areas.

7.2 ELIGIBILITY CRITERIA

7.2.1 Inclusion criteria
Pregnant women between 16-28 (both inclusive) weeks gestation, who have a viable singleton pregnancy, resident of the study area, willing to adhere to scheduled and unscheduled study visit procedures, and willing to deliver in a study clinic or hospital.

7.2.2 Exclusion criteria
Multiple pregnancies (i.e. twin/triplets), HIV-positive, known heart ailment, severe malformations or nonviable pregnancy observed by ultrasound, history of receiving SP during this pregnancy, unable to give consent, known allergy or contraindication to any of the study drugs.

7.3 INTERVENTIONS

7.3.1 Trial Medications and interventions

7.3.1.1 Name and description of study arms

All women will be randomly allocated to receive one of three IPTp regimens at enrolment and at each subsequent monthly ANC visit during the 2nd and 3rd trimesters. The range will be 3 to 7 visits depending on the gestational age of the woman at enrolment and delivery. Because recruitment is restricted to 16-28 weeks gestation, a median of 4 to 5 courses is expected.

1. ***IPTP-SP:*** This is the control arm and consists of a standard single-day stat course of 3 tablets of quality-assured SP (tablets of 500 mg of sulphadoxine and 25 mg of pyrimethamine) provided at enrolment and at each subsequent monthly ANC visit in the 2nd and 3rd trimester.
2. ***IPTP-DP:*** 3 to 5 tablets of DP (tablets of 40 mg of dihydroartemisinin and 320 mg of piperaquine, based on bodyweight) daily for 3 days provided at enrolment and at each subsequent monthly ANC visit in the 2nd and 3rd trimester plus 1gr (as 2 x 500mg equivalent tablet of placebo AZ daily for 2 days at enrolment. The dose of DP is equivalent to the standard treatment (case-management) dose for malaria in adults recommended by WHO with a target dose (range) of 4 (2-10) mg/kg bodyweight per day dihydroartemisinin and 18 (16-27) mg/kg bodyweight per day piperaquine given once a day for 3 days for adults and children weighing>= 25 kg (36-<60 kg: 3 tablets, 60-<80 kg:4 tablets, >=80 kg 5 tablets).[70] One 3-day course will be provided at enrolment and again at each subsequent monthly ANC visit in the 2nd and 3rd trimester. The weight at enrolment will be used to define the weight class to guide treatment both at enrolment and subsequent scheduled visits.
3. ***IPTP-DPAZ:*** 3 to 5 tablets of DP (based on bodyweight) daily for 3 days provided at enrolment and at each subsequent monthly ANC visit in the 2nd and 3rd trimester, plus 2 tablets of AZ (500 mg) daily for 2 days (2 g total over 2 days) at enrolment. The dose of AZ is the standard dose for the treatment of STIs/RTIs.[34]

Table 2: Dosing regimen per course

Study arm	Drug	Number of tablets once daily per course		
		Day-0	Day-1	Day-2
IPTp-SP	SP	3	0	0
	Total	3	0	0
IPTp-DP	DP*	3 to 5	3 to 5	3 to 5
	Placebo-AZ	2	2	0
	Total	5 to 7	5 to 5	3 to 5
IPTp-DPAZ	DP*	3 to 5	3 to 5	3 to 5
	AZ	2	2	0
	Total	5 to 7	5 to 7	3 to 5

SP: stat course of 3 tablets of quality-assured SP (tablets of 500 mg of sulphadoxine and 25 mg of pyrimethamine); **DP**: 3 to 5 tablets of DP (tablets of 40 mg of dihydroartemisinin and 320 mg of piperaquine, based on bodyweight) daily for 3 days (36-<60 kg: 3 tablets, 60-<80 kg:4 tablets, >=80 kg 5 tablets). **AZ**: 2g over 2 days as 1g (2 tablets of 500 mg) per day.

7.3.1.2 Administration of the study drugs

7.3.1.2.1 Day-0
All doses on day-0 will be provided as directly observed therapy. Participants will be given some clean water for swallowing the tablets. Women will then be observed for thirty minutes. If vomiting occurs during this time the full dose will be repeated. If the participant vomits again after the repeat dose she will be provided with unblinded standard IPTp-SP and not be issued the subsequent doses for that course (if any).

7.3.1.2.2 Day-1 and Day-2 (both DP arms)
Participants will be given the remaining day-1 and day-2 doses to take at home, with instructions to take one set of the pre-packaged tablets on each of the two days. Approximately 10% of women will be visited by a fieldworker on the third day to check whether they have taken the tablets and whether they have had any problems. This visit shall also serve to determine the availability, use the previous night, and integrity of LLITNs on a random basis.

7.3.1.3 Manufacturing and supply of study drugs

7.3.1.3.1 Dihydroartemisinin-piperaquine and matching placebo
Dihydroartemisinin-piperaquine (Eurartesim®) will be provided for the purpose of this study by Alfasigma (formerly Sigma Tau), Rome, Italy.

7.3.1.3.2 Azithromycin
Azithromycin and matching placebo tablets will be provided either by Pfizer as Zithromax®, or as generic azithromycin (and placebo) by other GMP manufactures (e.g Idifarma, a contract manufacturer in Spain, or Universal, Nairobi).

7.3.1.3.3 Sulphadoxine-pyrimethamine
Quality assured SP will be procured from a GMP qualified manufacturer.

7.3.1.4 Procedures for drug handling & accountability

7.3.1.4.1 Preparation and packaging
Each manufacturer will be responsible for the labelling of the active drug and will arrange shipment for further packaging and labelling. Bodyweight specific blister packs with the study arm specific active drug and placebo combinations will be prepared by Idifarma or an equivalent organisation or the study pharmacist, such that one box or envelop contains all the study drugs for that participant. Each box or envelope will contain smaller envelopes each containing a blister pack for a specific course. The specific study drug blisters will be kept in the box/envelope until dispensing. Packaging used for all treatment arms will be identical, labelled and blinded to the contents. The study drugs will be prepared and shipped in at least two different batches, to avoid any shelf life limitations.

7.3.1.4.2 Product storage
All study drugs will be stored in a secure area with access limited to Investigator and authorised study site personnel, and under appropriate storage conditions.

7.3.1.4.3 Product accountability
The site-PI will be responsible for establishing a system for the correct handling of study drug to ensure that:

1. Deliveries of study drug from the sponsor are correctly received by a responsible person (e.g. pharmacist assistant)
2. Accurate records are maintained for the receipt of study drug, for the dispensing of study drug to subjects and for returned drug.
3. Certificates of delivery and return must be signed preferably by the investigator or authorised personnel and copies retained in the investigator file.
4. Study drug is to be handled and stored safely and properly and in agreement with the given storage instructions.
5. The study drug is to be prescribed only by the principal investigator, co-investigators or study site personnel authorised to do so by the principal investigator.
6. Study drug is dispensed only to study subjects in accordance with the protocol.
7. Subjects must return all unused medication and empty containers to the investigator.
8. At the end of the study, delivery records must be reconciled with records of usage and returned stock. Any discrepancies must be accounted for in writing.
9. Once accounted for, any returned and unused study treatment at the site will be returned to the sponsor for destruction or destroyed locally upon agreement with the sponsor. Drug destruction certificates will be issued that refers to the subject study numbers for subject specific medication that was destroyed.

7.3.1.4.4 Pharmacist, pharmacist assistant or dispenser

All efforts will be made for the preparation, packaging and labelling of the blinded study drug to be performed and documented in accordance with Good Manufacturing Practice (GMP).

7.3.2 Criteria for discontinuing or modifying allocated interventions for a given trial participant

7.3.2.1 Symptomatic malaria

7.3.2.1.1 Febrile women at enrolment visit

Women who are febrile during the enrolment visit will not be excluded. They will follow the standard enrolment and randomization procedures during that initial visit. In addition, a malaria RDT will be taken. Women who have confirmed malaria by RDT will be treated with a 3-day course of artemether-lumefantrine (AL) for clinical malaria as per national guidelines and will not yet be given the study intervention. The next study course will then be provided during the next scheduled monthly visit. This will be recorded on the CRF. Women with negative RDT will be given study drug as per randomization arm.

7.3.2.1.2 Febrile women during scheduled visits

Similarly, women in any group who attend for a scheduled study appointment with fever and malaria confirmed by RDT will be treated with a 3-day course of AL for clinical malaria and will not be given the study intervention at that visit. This will be recorded on the CRF. The next study course will then be provided during the next scheduled monthly visit. This will be recorded on the CRF. Women with negative RDT will be given study drug as per randomization arm.

7.3.2.1.3 Unscheduled visits

Women diagnosed with symptomatic uncomplicated malaria during the course of the study will be treated with AL and their next scheduled monthly ANC visit postponed until at least two weeks have passed. This will be recorded on the CRF.

7.3.2.2 Allergy to study medication or other safety reasons
Other reasons leading to withdrawal from study interventions include suspected or confirmed allergic reaction to the study drug or for safety reasons as judged by the investigator, study safety monitor or DMEC. See also paragraph 7.3.5 Strategies to improve adherence to study protocol monitoring .

7.3.3 Permitted and prohibited concomitant medication and care

7.3.3.1 Routine care and permitted concomitant medication
All participants will be offered routine antenatal care according to local policy and the principles of 'focused antenatal care'. This includes the provision of insecticide-treated bed nets (ITNs) free of charge. Routine antenatal care also includes blood screening at the first appointment for syphilis, anaemia and HIV, and the provision of appropriate treatment for these conditions. Because HIV is an exclusion criterion, women diagnosed prior to enrolment as HIV-positive will be excluded from the trial and referred to routine care for assessment, and initiation of cotrimoxazole prophylaxis and appropriate anti-retroviral treatment.

Participants will be counselled to avoid concomitant medications not prescribed by the study clinic, specifically antimalarial drugs or drugs that may be associated with QTc prolongation. All concomitant medications taken during the study will be recorded in the appropriate sections of the CRF with indication, dose information, and dates of administration.

All routine medication used for antenatal care is permitted, with the exception listed under the paragraph 7.3.3.2, Prohibited medication, page 30. Permitted medication includes the use of AL (first line) and quinine or amodiaquine-artesunate (second line) for treatment of uncomplicated malaria, or artesunate or quinine for severe malaria as per national guidelines. As part of their antenatal care participants will be given iron and folic acid supplements (folic acid dose 0.4 mg to 0.6 mg / day) and tetanus vaccination if applicable. Helminth infections will be treated presumptively with albendazole 400 mg / kg single dose for hookworm, trichuriasis and ascariasis.

7.3.3.2 Prohibited medication
Prohibited medication includes antimalarial drugs not prescribed within the trial protocol, and other drugs with antimalarial properties including daily cotrimoxazole prophylaxis. It also includes high dose folic acid supplementation (>1.5. mg/day such as the commonly used 5 mg tablets per day) and antiretroviral therapy. Randomised participants who take prohibited medications resulting in the premature cessation of the study intervention, will remain in the trial and will be included in the primary, intention-to-treat analysis, but excluded from the per-protocol analysis.

7.3.4 Strategies to improve adherence to study protocol monitoring adherence

7.3.4.1 Adherence to study protocol and medication
Where feasible, study participants will be reminded about any follow-up visits through mobile phone contact. All information will be recorded on the appropriate sections of the CRF. Subjects judged to be non-compliant may continue in the study but will be counselled on the importance of taking their study medication as prescribed.

7.3.4.2 Strategies for retention
During screening and consent procedures, women will be asked whether they live in the catchment area, are willing to adhere to the study protocol, and deliver in a study clinic or hospital. Specifically, potential participants will be asked whether they will be willing and able to comply with the

frequent follow-up schedule and whether they need to travel out of the study area for an extended period during the follow-up period. The 'study catchment area' will be defined for each study site before the start of the study. All participants will be reimbursed for transportation costs to and from the clinic.

GPS position and otherwise, detailed directions to participants' homes as well as contact information, including mobile phone information, will be recorded at enrolment. If women do not return for scheduled follow-up visits, the study team will call them and ask them to come to the clinic for evaluation, offering transport reimbursement, or if women do not have access to a mobile phone, study staff may visit their house to help arrange transport to the clinic if the women are willing to come to the clinic, or, alternately, a study staff member may go to their home for clinical evaluation and to assess if they still wish to participate in the study. The women's travel costs will be reimbursed. All information will be recorded on the appropriate sections of the CRF. Subjects judged to be non-compliant may continue in the study but should be counselled on the importance of taking their study medication as prescribed.

7.3.4.3 Drug Compliance

The first dose of each course will be given under supervision of study staff and the remaining 2 doses for days 2 and 3 will be taken at home by the study women. Women will be reminded through mobile phone contact to take the assigned tablets for the second and third dose. A sub-sample of women will be visited at home by random spot checks (some on Day 2 and other women on Day 3, with Day 1 being the first day of treatment) to assess adherence to the study drugs. In our previous trial in western Kenya documented adherence using this method was very high (>95%).[23]

7.3.5 Removal of participants from study

7.3.5.1 Removal of participants from therapy and/or assessment

Participants can discontinue from the study for any one of the following reasons:

1. Screening error resulting in incorrect enrolment (found that subject did not meet required inclusion / exclusion criteria)
2. Withdrawal of consent at any stage or subject not willing to continue in the study
3. Suspected or confirmed allergic reaction to the study drug (removal from therapy only)
4. Safety reasons as judged by the investigator, study safety monitor or DMEC (removal from therapy only)

Where feasible, the participants who discontinue from the study treatment or from the study entirely will be asked about the reason(s) for their discontinuation and the presence of adverse events. Participants have the right to unconditionally withdraw from participation in the study without providing a reason. If a subject discontinues, data captured will contribute to the analysis up to the day prior to discontinuation, unless the subject indicates that she withdraws consent for any data captured up to that point. Every effort will be made to follow-up participants who discontinue due to drug related adverse events to determine the birth outcome. If a subject discontinues due to drug-related adverse events, all the assessments that would have been carried out at the next scheduled visit will be conducted at day 28 where reasonably possible (unless consent is withdrawn). This will be recorded in the CRFs. The study drug will be returned by the subject. Subjects that have discontinued the study prematurely will not be replaced.

7.3.5.2 Discontinuation from storage of samples for future studies

The applicability of long term storage of study samples for future research shall be in respect to the regulations of the country specific ethics board. When a subject's consent for long-term storage is withdrawn, the stored sample will be destroyed and the withdrawal noted in the CRF. If the request is received after the dataset has been anonymised, the stored sample can no longer be withdrawn.

7.4 OUTCOMES

7.4.1 Primary outcome

Adverse pregnancy outcome

The primary outcome is 'Adverse pregnancy outcome' defined as the composite of foetal loss (spontaneous abortion or stillbirth), or singleton live births born small-for-gestational age (SGA), or with low birthweight (LBW), or preterm (PT) (SGA-LBW-PT), or subsequent neonatal death by day 28. Small for gestational age will be defined using the new INTERGROWTH population reference's 10th percentile.[71]

7.4.2 Key secondary outcome measures

7.4.2.1 efficacy

1. Composite of foetal loss and neonatal mortality
2. SGA-LBW-PT composite
3. The individual components of the above composites newborn outcome both as categorical and continuous variables
4. Neonatal length and stunting
5. Clinical malaria during pregnancy
6. Malaria infection during pregnancy detected by microscopy and PCR (not for point of care)
7. Placental malaria detected by microscopy, by molecular methods or by histology (past and active infection)
8. Individual components of the placental malaria composite
9. Maternal nutritional status
10. Maternal anaemia and haemoglobin concentration during pregnancy and delivery
11. Congenital anaemia
12. Congenital malaria infection

7.4.2.2 Safety

1. QTc-prolongation
2. Congenital malformations
3. Maternal mortality
4. Other SAEs and AEs

7.4.2.3 Tolerance

1. History of vomiting study drug
2. Dizziness
3. Gastrointestinal complaints

7.4.2.3 Antimicrobial activity and resistance

1. Molecular markers of drug resistance in *Plasmodium falciparum* infections during pregnancy and delivery

2. Presence of STIs/RTIs prior to delivery (syphilis, gonorrhoea, *Chlamydia trachomatis*, *Trichomonas vaginalis,* and bacterial vaginosis)
3. Changes in macrolide resistance in Pneumococcus detected in maternal nasopharyngeal samples
4. Changes in the colony composition of maternal vaginal microbiota, and intestinal microbiota of mother and infantPARTICIPANTS TIMELINE

7.5.1 Antenatal booking visit

If a woman makes first contact with the study team between 16 and 28 weeks gestation (inclusive), the first study visit will immediately follow eligibility screening. If a woman makes first contact before 16 weeks gestation, she will be invited to be enrolled into the study at the next clinic visit when she would have reached the eligible gestational age where all screening and enrolment procedures will be conducted. Additional informed consent shall be sought from 160 women (80 per DP intervention arm) for cardiac monitoring and safety evaluation at some sites.

7.5.1.1 Enrolment

After informed consent has been signed, participants will be randomly allocated (see section 8) to receive either IPTp-SP, IPTp-DP, or IPTp-DP+AZ (see section8, Methods: Assignment of interventions, page 39) and issued with a trial identifier with their unique study identification number.

At this stage study staff will ask the participant for a home or mobile telephone number if they have one, and home address or a description of the location of their home, and for their verbal consent to be visited at home whenever they do not attend scheduled appointments.

7.5.1.2 Baseline assessment

A baseline assessment will be conducted for each participant comprising demographic information, socioeconomic information, ITN and IRS use, medical and obstetric history, and clinical assessment. Any relevant information already collected during the screening process or at previous antenatal appointments for the same pregnancy will be copied from the antenatal cards onto the CRF, so that questions, tests and examinations are not unnecessarily duplicated.

Study-relevant information recorded at the first visit will include age; area of residence; prior use of ITNs; number of previous known pregnancies and number of previous births and corresponding pregnancy outcomes. Clinical assessment will include height and weight, mid-upper arm circumference, fundal height, reported date of last menstrual period and estimated gestation.

7.5.1.3 Biological samples

A venous (up to 10 mL) or finger prick whole blood sample (~250 µL) will be taken and used for both routine and study-specific testing. This will be obtained after consent for eligibility screening has been obtained but before consent for enrolment in the trial; women who have not been tested for HIV in their current pregnancy will be tested for HIV, and all women will be tested for anaemia and haemoglobin levels. Women not previously tested for syphilis in this pregnancy will be tested and treated if positive. The same blood sample will be prepared for study-specific diagnostic testing for malaria (e.g. not for point of care). A filter-paper sample will be prepared and stored for parasite genetic studies.

In a subgroup of 160 women enrolled in the nested cardiac monitoring study in the DP arms, an additional 2 mL of whole blood will be required for drug level assessment, full blood count, biochemistry for electrolytes, liver and renal function test, 4-6 hours after the 3rd dose of DP intervention.

In a subgroup of approximately 1,464 women in selected sites, women will be requested to provide a self-administered vaginal swab and a stool sample at enrolment for baseline reproductive tract and gut microbiota colonisation respectively. If no fresh stool sample can be produced on or just before the day of enrolment, the woman shall be requested to submit the stool sample the following day.

A sub-group of approximately 1,032 women will be asked to provide nasopharyngeal swabs for carriage macrolide resistant Streptococcus.

7.5.1.4 Study intervention
The first course of IPTp will be administered to asymptomatic women at the visit as described in section7.3.1, Trial Medications and interventions, page 27. If women have symptomatic malaria, they will be treated as described in section 7.3.2.1.: Symptomatic malaria.

7.5.1.5 Routine antenatal care and treatment of illness
Routine antenatal care and treatment of any illness identified at this visit will be provided as described in section 7.3.3.1, Routine care and permitted concomitant medication, page 30. This will include the provision of an ITN if the woman does not already have one, and advice on sleeping under the net for the entire duration of the pregnancy.

7.5.1.6 Recording of morbidity and medication taken
All participants will be asked about any symptoms or illnesses they have had in the last month, and any medication taken. These will be recorded in the adverse events section of the CRF. All AEs shall also be evaluated for seriousness and reported accordingly.

7.5.2 Interim monthly scheduled visits
Subsequent clinic visits will be scheduled monthly after the first visit.

7.5.2.1 Serial ultrasound scans
In selected sites, approximately 1,464 women shall be requested to undergo two more additional ultrasound scans at interim monthly visits at the approximate gestational ages of 25-28 weeks and 32-35 weeks (or otherwise at the nearest scheduled monthly visit).

7.5.2.2 Biological samples
A venous (up to 10 mL) or finger prick whole blood sample (~250 µL) will be taken for the same assays as the baseline sample, with the exception of syphilis testing which will only be conducted once at the initial visit. A second HIV test and nutritional biomarkers will be done once in third trimester only during one of the last scheduled visits prior to delivery. The samples taken for malaria diagnostics, including malaria smears will be taken for later diagnostic analysis and not for patient care. A standard malaria RDT will be taken from women with fever or a history of fever in the last 48 hours for patient care (see 7.3.2.1, Symptomatic malaria, page 29).

At each scheduled visit, the subgroup of 160 women enrolled in the nested cardiac monitoring study will have an additional 4mL of whole blood drawn for the assessment of drug levels, full blood count, and biochemistry for electrolytes, liver and renal function tests; This includes 2 mL taken just before the first dose of each course and 2 mL 4-6 hours after the 3rd dose of each course; i.e. just after the respective ECGs have been taken at these time points.

In the subgroup taken part in the micro-biota component in selected sites, the women will be requested to provide a further high vaginal swab and a stool sample for reproductive tract and gut microbiota colonisation respectively during one of the last scheduled visits prior to delivery.

Similarly, a nasopharyngeal swab will be requested again from the women taking part in the component that assesses the carriage rate of macrolide resistant Streptococcus during one of the last scheduled visits prior to delivery.

7.5.2.3 Study intervention
The IPTp will be administered to asymptomatic women at these visits as described in section 7.3.1, Trial Medications and interventions, page 27. If women have symptomatic malaria they will be treated as described in section 7.3.2.1.: Symptomatic malaria.

7.5.2.4 Routine antenatal care and treatment of illness
Routine antenatal care and treatment of any illness identified at this visit will be provided as described in section 7.3.2.1, Symptomatic malaria, page 29.

7.5.2.5 Recording of morbidity and medication
Participants will be asked about any symptoms or illnesses they have had since the start of pregnancy, and any medication taken, and these will be appropriately documented in the CRFs.

7.5.3 Unscheduled visits during pregnancy
Participants will be encouraged to visit the clinic if they feel unwell between scheduled appointments. Participants who present between appointments will be examined by study staff. Presenting symptoms, axillary temperature and blood pressure will be recorded. A finger-prick blood sample will be taken for Hb measurement (if clinically indicated) and malaria RDT for point of care, and a malaria smear and dried blood spot for later analysis of malaria. Any illness will be treated as appropriate and according to standard local care. The date of attendance, diagnosis and treatment will be recorded on the CRF, and any adverse events reported according to standard procedures.

7.5.4 Delivery visit
Women will be encouraged to deliver on the maternity wards of the participating clinics, in which case they will be assisted to delivery by regular clinic midwives, and a specially trained staff will visit them on the ward and perform relevant examinations shortly after delivery. Women who deliver at home will be identified using a network of community health workers such as health surveillance staff or village health volunteers/workers, and will be requested to report to the health facility within 48 hours, to collect, as far as possible, the same information.

7.5.4.1 Biological sampling mother
A similar venous (up to 10 mL) or finger prick whole blood sample (~250 µL) will be taken as for the scheduled follow-up visits for the same assays as described in Table 1: Summary Table of Study Design and Schedule of Assessment, page 15 and in more detail in Appendix VII. Description of other clinical and laboratory methods, page 84. Women taking part in the component that assesses the impact on the carriage rate of macrolide resistant Streptococcus will be asked to provide another nasopharyngeal swab (if the delivery timepoint is missed this can be taken at subsequent post-natal follow-up visits up to 6-8 weeks post-partum).

7.5.4.2 Placental sampling
A blood sample will be collected from the delivered placenta by making an incision on the maternal side and collecting the pooled blood. This sample will be tested for malaria using standard microscopy of blood smear and PCR, and drug levels.

A 2cm x 2cm x 1cm specimen of placental tissue will be taken from the maternal side for histopathology testing for current and past malarial infection. An amnion roll will be included in the collection of the placental sample for histological evaluation of chorio and/ or amnionitis.

7.5.4.3 Umbilical cord sampling

A 5 mL umbilical cord blood sample will be taken for haemoglobin level, malaria molecular diagnostics, antibody and cell mediated immune responses to malaria and other pathogens, and nutritional biomarkers including leptin, adiponectin, and drug levels. This will be a total of 7 mL for those contributing to the assessment of the impact on immunological outcomes: Maternal antibody, trans-placental antibody transfer and multi-pathogen neonatal cell mediated immune responses (page 88) for the additional assays on cell mediated immune responses involving peripheral blood mononuclear cell (PBMC).

7.5.4.4 Examination of the baby

All babies shall be examined for vital status, jaundice and the presence of congenital abnormalities. Birthweight, using digital scales, head circumference, length, and umbilical abdominal circumference shall be measured. In a subgroup, additional new-born anthropometric measurements including body composition, chest circumference, abdominal circumference below the xiphoid process, mid-upper arm circumference, skinfold (triceps, biceps, subscapular, quadriceps) measures will be taken.

7.5.5 Postnatal visits

7.5.5.1 Seven-day postnatal visit

Participants and their babies will be seen at the clinic by a study nurse around seven days after birth. The baby will be examined for the presence of jaundice and for any congenital anomalies that may have been missed at delivery. A heelprick sample (about 500 microL) will be taken in all babies with fever or other symptoms will have blood samples taken for testing by RDT, microscopy and DBS, and in a subgroup of asymptomatic babies for assays to assess immune responses to malaria and other infectious diseases. All treatment will be provided based on the RDT results. Standard postnatal advice and healthcare will be provided as needed for mother and baby, and any treatment and clinical findings for the baby recorded on the CRF.

Routine postnatal care will be provided for the women. In a sub-group of women, nutritional status postpartum shall be assessed through measurement of skinfold thickness, hip circumference, waist circumference and bioelectrical impedance analysis. Women who do not attend for this appointment will be visited at home if possible. Any infant deaths occurring before the visit will be recorded; the date and probable cause of death will be ascertained by verbal autopsy.

7.5.5.2 Six to eight -week postnatal visit

Participants and their babies will be seen again at the clinic around six to eight weeks after birth, at their baby's second vaccination visit. The baby will be examined for the presence of congenital anomalies that may have been missed at delivery or the first visit.

A similar heel prick sample (about 500 microL) as on day 7 will be taken again for malaria diagnosis and for immunological assays with treatment provided based on the RDT results. Standard postnatal advice and healthcare will be provided as needed for mother and baby, and any treatment and clinical findings for the baby recorded on the CRF. Routine postnatal care will be provided for the women. In a sub-group of women, nutritional status postpartum shall be assessed through measurement of skinfold thickness, hip circumference, waist circumference and bioelectrical impedance analysis. Women who do not attend the scheduled appointment will be visited at home

if possible. Any infant deaths occurring before the visit will be recorded; the date and probable cause of death will be ascertained by verbal autopsy.

A stool sample from the neonate will be collected to determine the gut microbiota. If not available on the day of visit, the mother shall be requested to submit the sample the following day. More detailed maternal and neonatal anthropometric measurements shall also be collected in a subgroup of approximately 1,464 children.

7.5.5.3 Unscheduled postnatal visits

Women will be encouraged to attend the clinic if they or their baby become unwell during the follow-up postnatal period. Physical examinations and relevant blood samples will be conducted to guide management. Any illness will be treated as appropriate and according to standard local care. The date of attendance, diagnosis and treatment will be recorded on the CRF, and any adverse events reported according to standard procedures. Clinical care will also be provided for women who attend because of their own illness, but this will not be recorded as part of the study.

7.6 SAMPLE SIZE

7.6.1 Primary endpoint

7.6.1.1 Rationale behind sample size calculations

The study is designed based on three main goals:

a) to provide 80% power as a standalone trial to detect ≥20% relative reduction (RR=0.80) by IPTp-DP in the primary endpoint from 21.3% in the SP arm, and
b) to provide at least 80% power to detect a further relative reduction of 25% by IPTp-DP+AZ relative to IPTp-DP alone, and
c) to take the existing evidence into account of the effect of IPTp-DP alone, such that on completion, a prospective meta-analysis combining the new evidence with the existing evidence on the effect of IPTp-DP versus IPTp-SP would have ≥80% power to detect a 17% relative reduction (RR=0.83) in the same primary composite endpoint. The 17% (95% CI -10 to 37) reduction is based on the pooled effect size obtained from a fixed-effects meta-analysis of the previous two trials from Kenya[23] and Uganda[27] conducted for this protocol.

7.6.1.2 Sample size for a standalone trial

The sample size calculations for the standalone trial were conducted using PASS software. The total sample size will be 4,680 women (1,560 per arm) across three countries pooled. A sample size of 4,020 women (1,340 per arm) will achieve 80% power trial to detect a ≥20% reduction in the primary endpoint from 21.3% with IPTp-SP to 17.0% (RR=0.80) (α=0.05) (90% power to detect a 23.0% reduction). 4,035 (1,345 per arm) women are required to allow for 1 interim analysis using the Lan-DeMets spending function with O'Brien-Fleming type boundaries to preserve the overall two-sided type I error rate for efficacy (see.10.1.3, page 45).

We will recruit 4,680 women (1,560 per arm) to allow for 13.7% loss to follow-up, which is approximately double the rate of loss to follow-up for this same primary composite endpoint in our recently completed MiP trials in Malawi (N=1873, across 2 arms[68]) and Kenya (N=1546, across 3 arms[23]). These trials involved similar sample sizes per country as in the proposed study, however the higher rate of loss to follow-up is used for our current sample size calculations since not all hospitals will have been part of the previous studies.

This same sample size of 1,340 completers per arm also provides 80% power to detect a further 22.8% reduction (RR=0.772) from 17.0% in the IPTp-DP alone arm to 13.1% in the IPTP-DP+AZ arm (α=0.05) (or 90% power to detect a 26.1% reduction [RR=0.739] from 17.0% to 12.6%). The 21.3% primary endpoint prevalence in the control arm is the observed mean prevalence in the IPTp-SP arms of our previous trials in the same sites in Kenya[23] and Malawi,[68] which also provided LLINs on enrolment, and in observational cohort studies in Tanzania.[72]

7.6.1.3 Power of updated prospective meta-analysis comparing IPTp-DP alone vs IPTp-SP

The power calculations for the updated meta-analysis were conducted using the Metapow package in STATA, which uses simulations to calculate the appropriate, 'evidence-based', sample size using the available information from previous trials.[73] In addition to the existing two trials, information from a third trial (PROMOTE-II) will become available in 2017/2018. This relatively small 2-arm trial (n=391 per arm) will be conducted in eastern Uganda using an identical design as the proposed study (Dorsey et al). The effect size of this PROMOTE-II trial was also set to 17% for the sample size calculation. An updated meta-analysis that takes the evidence from the 2 completed trials, the planned PROMOTE-II trial, as well as this new proposed trial into account, this same sample size of 1,340 completers per arm provides 84.9% power (95% CI 84.7-85.1) to detect a relative reduction of 17% in the same primary endpoint by IPTp-DP alone relative to the IPTp-SP arm (100,000 simulations, α=0.05).

7.6.2 Power calculations for key secondary endpoints

7.6.2.1 Foetal loss and neonatal mortality

An overall sample size of 1,340/arm also provides 80% power to detect a 43.8% reduction in foetal loss/neonatal mortality from 4.6% in the SP arm to 2.6% with DP (RR=0.562, α=0.05). The 4.6% estimate for this endpoint was the average observed in the SP arms in Kenya, Malawi and Tanzania.[23,68,72] The 43.8% reduction is similar to the pooled effect size of 57% obtained from fixed effects meta-analysis from the trials in Kenya[23] and Uganda.[27]

7.6.2.2 SGA-LBW-PT

This same sample size also provides 80% power to detect a 21.5% reduction in the composite outcome of SGA-LBW-PT, from 18.8% to 14.8% (RR=0.7852, α=0.05), again allowing for 13.7% loss to follow-up. The 18.8% estimate is the observed average in Kenya,[23] Malawi,[68] and Tanzania,[72] recalculated for this protocol using the new INTERGROWTH population reference's 10th percentile to define SGA.[71]

7.6.2.3 7.6.2.3. Other secondary endpoints

The sample size calculations for subgroups involved in the assessment of other secondary endpoints including immunological, nutritional endpoints are presented in Appendix VII. Description of other clinical , page 84.

7.6.3 Power calculations for cardiac monitoring and safety study

To detect a difference of 10 milliseconds (ms) in QTc prolongation observed between the first and last course of DP, a sample size of 68 women per arm are required (SD 25 ms [based on 1,002 observations in children], 90% power, two-sided α=0.05, paired test). To account for 15% loss to follow-up, 80 women per DP arm will be recruited (160 in total).

7.7 Recruitment and retention

7.7.1 Recruitment procedures

Eligible women who are resident in the study area will be recruited when they start attending for antenatal care in one of the ANC clinics in the participating hospitals. The study will provide dedicated study staff to deal with the additional workload. Only dedicated study staff will be involved with the recruitment and consent procedures. Where required, the trial will fund minor improvements in the infrastructure, such as the refurbishment of a dedicated study room, or pay rent to the hospital to use facilities.

7.7.2 Planned recruitment rate

Recruitment of 4,680 women (all gravidae) from 10 sites will require 17 months, with an additional 7 months to complete pregnancy plus infant follow-up. The recruitment rate of 280 HIV-negative women per month, or an average of 28 per site was the average recruitment in the most recent malaria in pregnancy interventions trials in Malawi[68] and Kenya,[23] conducted by the same teams. This corresponds to approximately 1 delivery per day per site, which is an adequate workload that helps ensure the teams are not overburdened and the data quality and completeness of follow-up is maintained to the highest standards.

7.7.3 Recruitment strategies for achieving target sample size

The enrolment of the target sample size is scheduled to be completed in an 18-month period, requiring an average of 260 participants per month. Each country has several proven study sites with high volume antenatal clinics that have met required sample sizes of similar magnitude before. Recruitment will be competitive between sites within a country. It is anticipated that any lag in recruitment by a site can be shifted to other sites, before being shifted to sites in the other participating countries.

8 Methods: Assignment of interventions

8.1 Allocation

Permuted block randomisation, stratified by site and two gravidity groups (paucigravidae [1st and 2nd pregnancies] and multigravidae) will be used to generate randomisation lists. This will be done by a statistician at LSTM who is not involved in the study. The randomisation assignments will be put in sequentially numbered, sealed, opaque envelopes, which will be opened sequentially upon enrolment of a study participant in each site. Stratification by gravidity is essential to ensure balanced distribution among study arms as this is the most important known effect modifier of susceptibility to malaria infection and the corresponding impact on pregnancy outcomes.

8.2 Blinding and unblinding

8.2.1 Blinding

The study will be a partially placebo-controlled involving a single placebo for AZ. To further minimise bias, we will use an objective primary outcome measure and mask all laboratory staff to the treatment assignment of individual women. The trial statistician will also be blinded regarding the treatment code when he develops the statistical analysis plan and writes the statistical programmes, which will be validated and completed using dummy randomisation codes. The actual allocation will only be provided to the study team after locking of the database and approval of the statistical

analysis plan by the independent Data Monitoring and Ethics Committee (DMEC) before they review any trial results. The study statistician conducting the interim analysis or sample size re-estimation will remain blinded throughout the analysis.

8.2.2 Emergency unblinding

To maintain the overall quality and legitimacy of the clinical trial, code breaks for the AZ arms will occur only in exceptional circumstances when knowledge of the actual treatment is absolutely essential for further management of the participant. Site investigators will be encouraged to discuss with the principal and chief investigators if he/she believes that unblinding is necessary. If unblinding is deemed to be necessary, the investigator will follow a standard operating procedures that will be developed specific for this trial describing the system for emergency unblinding. The unblinding shall be performed by the sponsor for that specific patient by the sponsor before notification of the SUSAR to the ethics committee and regulatory authority of all participating countries. As far as possible, the blinding shall be maintained for the trial staff, investigators and the biostatistician responsible for data analysis and interpretation. Consequently, the SUSAR and required unblinding should therefore be processed by a person assigned by the sponsor who is not involved in the conduct of the trial. The Data Monitoring and Ethics Committee (DMEC) may perform the duties required in case of unblinding to ensure that the sponsor and the trial team remain blinded.

Because the unblinding will be on an individual basis, the blinding of other participants is not affected. All Investigators are encouraged to maintain the blind as far as possible following the subsequent follow-up of the participant. The actual allocation will NOT be disclosed to the participant and/or other study personnel including other site personnel, monitors, corporate sponsors or project office staff; nor will there be any written or verbal disclosure of the code in any of the corresponding participant documents, with the exception of correspondence between the principal or chief investigator and the safety monitor or the DMEC chair. All code breaks (with reason) will be reported as they occur on a dedicated case report form. Unblinding will not necessarily be a reason for study drug discontinuation unless it is absolutely essential for the management of the participant.

9 METHODS DATA COLLECTION, MANAGEMENT AND ANALYSIS

9.1 PROCEDURES FOR ASSESSING EFFICACY AND SAFETY PARAMETERS

9.1.1 Efficacy

9.1.1.1 SGA-LBW-PT

New-borns will be weighed within 24 hours of delivery using digital scales (± 10 gr) and LBW defined as < 2,500g. Gestational age will be assessed using ultrasound dating at enrolment and preterm (PT) defined as <37 weeks' gestation. Small for gestational age (SGA) will be defined as birthweight below the tenth percentile for a given gestational age and sex, using the new INTERGROWTH reference population.[71]This will also allow the calculation of Z-scores.

9.1.1.2 Pregnancy outcome and neonatal death

Foetal loss will be assessed monthly at scheduled ANC visits. If women do not return they will be contacted by mobile phone and/or visited at home to document this. Neonatal death will be assessed at the 6-week follow-up visit.

9.1.1.3 Malaria infection
Malaria infection will be assessed at the scheduled monthly ANC visits and at delivery by taking dried blood spots for malaria diagnosis by molecular detection methods such as PCR or LAMP or Illumina®-based sequencing. Standard light microscopy will be used to evaluate malaria smears and RDTs will be used for point-of-care diagnosis in febrile women or women with a history of fever in the last 48 hours to measure the incidence of clinical malaria, with RDT-positivity defined as either pLDH or HRP2 antigen positivity. At delivery, samples will be taken for molecular diagnostics (henceforth referred to as PCR), microscopy, and placental histology.

9.1.2 Safety
The presence of congenital abnormalities and jaundice will be assessed at delivery, day 7, and at 6 to 8 weeks. The vital status of participants will be recorded at each scheduled and unscheduled visit, and through follow-up phone calls and home visits if women do not attend the scheduled visit. Maternal mortality will be defined as the death of a woman while pregnant or within 42 days of termination of pregnancy, irrespective of the duration and site of the pregnancy, from any cause related to or aggravated by the pregnancy or its management but not from accidental or incidental causes. A verbal autopsy questionnaire will attempt to determine the cause of death.

9.1.2.1 Procedures for cardiac monitoring
Cardiac monitoring will be conducted in 160 women; 80 per DP arm.

9.1.2.1.1 Electrocardiogram (ECG)y
ECGs will be conducted prior to the first dose and then again 4-6 hours after the 3rd dose of each course of IPTp containing DP by dedicated staff at the clinic and results read by local clinicians; All ECGs will also be read off-site by blinded clinicians and discrepancies resolved by an experienced cardiologist, such as those from Cardiabase, Banook Group, Nancy, France. Confirmed QTc values >480ms will be considered as contradiction for subsequent IPTp courses with DP. In such events, women will continue in the study but will received IPTp with SP instead of DP. The results of the external expert reading of ECGs will be available to the site PI or assigned clinical staff within hours of reading. ECGs and results will be available on a secure server (provided by the study or by the external experts, e.g. by Cardiabase). The external experts and the site PI will be alerted by e-mail as soon as the ECGs and results respectively have been uploaded onto the server. Feedback to participants will be provided on site. This will be within minutes after the ECG has been taken if the automated output from the ECG machine suggest the ECG is normal. If the output suggests any other variation, results will be provided within 1 to 2 hours following confirmatory reading by the study clinician. If the subsequent results of the expert reading disagree with the initial reading on site, and if these are of clinical relevance to the patient, results will be shared with the participant when she returns for her follow-up ECG 2 days later if it pertains to the baseline ECG prior to each course. If the expert results relate to ECGs taken after the 3rd dose of each course (Tmax), the results shall be provided to the participant at the next scheduled antenatal visit prior to the first dose for that visit. In the event of any findings with clinical significance, participants shall be traced and requested to report to the study clinic as soon as possible for communication of the findings and appropriate clinical management.

9.1.2.1.2 Plasma piperaquine levels, full blood count, renal function and liver function
A few minutes after the last ECG is taken (i.e. 4-6 hours after the last dose of each course), a 2 ml venous blood sample will be obtained for piperaquine and azithromycin drug levels to determine the relationship between drug level and magnitude of QTc prolongation. The sample will also be used to determine the impact of monthly dosing on full blood count, renal and hepatic function.

Furthermore, a at delivery, maternal placental blood and cord blood sample will be used for the same drug levels to quantify drug exposure to placental parasites and the neonate, respectively.

9.1.3 Laboratory and nutritional assays

Some laboratory assays will be conducted at collaborating institutions in Australia, United States of America, Canada, United Kingdom, and Europe. A detailed description of the assays and analyses are provided in Appendix VII. Description of other clinical and laboratory methods.

9.2 DATA COLLECTION METHODS & STORAGE

9.2.1 Data management

9.2.1.1 Methodologies for data collection / generation

A combination of paper-based and electronic record forms will be used. For the questionnaires that are administered in paper format, the study will utilise HP Teleform software to design the paper-based CRFs for semi-automated transcribing into an electronic database using scanning and Optical Character Recognition, intelligent document recognition and data validation using checksum algorithms, cross-field validation, and human verification of information against source documents. Once validated, the data will be transferred to the target database along with a PDF of the original image of the CRFs, such that there is an electronic copy of all paper-based documents. For the electronic-only data capture, such as home visits to administer drug adherence and bed net questionnaires, we will use tablets with integrated sim cards to transfer encrypted data to the ODK or similar servers where the electronic clinical data for the participants will be hosted.

9.2.1.2 Data quality and standards

The quality of questionnaire data collection and data entry will be maximised through training of field staff in the standardised questionnaire administration methodology. Field staff will be trained in the methodology for collecting data and will be expected to demonstrate competence before conducting fieldwork. All electronic CRFs and data validations processes for data captured through Teleform will incorporate range and consistency checks.

9.2.1.3 Managing, storing and curating data

Verified and validated data from all 3 countries will be stored via the cloud on a secure server at LSTM in Liverpool and/or KEMRI, Kisumu, Kenya. The PIs from each site will have access to their site data on the central server. Locally, data will be backed-up on a continuous basis on a secure off-site server and on encrypted standalone hard drives.

Once the data validation phase is completed by the central data manager, the database will be locked and transferred to a statistical programmer who will do further syntax driven consistency checks and syntax driven data cleaning (e.g. in Stata). The statistical programmer will have access to the PDF copies of the source data. He/she will then prepare the database for data analysis by the trial statistician by creating the final variables for data analysis, such as the creation of the composite endpoints. The final cleaned database will be available as SAS, STATA and in SPSS format, with embedded data dictionary.

9.2.1.4 Metadata standards and data documentation

The full study protocol, supporting documents and the full anonymised research database will be made publicly available once the study findings have been published. The data manager and statistical programmer will produce a document summarising the methods used to generate the

data with a full description of all procedures, analyses, data capture tools, coding and description of variables. This document will be available alongside the research database.

9.2.1.5 Data preservation strategy and standards

The majority of data collected will be captured using scannable paper-based forms. The scanned PDF copies will be used for archiving, and the original paper questionnaires kept in a dedicated lockable data storage room in country and then destroyed after a minimum period subject to the prevailing laws in each country. The research data will be stored for the long-term in the original electronic format, in a unified large database and a public database that contains all research data other than participant identifiable data. The public database will be updated when needed if software becomes obsolete to achieve long-term preservation. The data will be preserved in this way for 10 years or longer if still being accessed at that stage.

9.3 STATISTICAL METHODS

9.3.1 General principles

All statistical analyses will be described in detail in the Statistical Analysis Plan (SAP) and finalised and signed before unblinding the study. One interim analysis and/or one sample size re-estimation will be conducted as described in sections 7.6.1.2, page 37 and 10.1.3, page 45. The study is designed as a superiority trial, so all tests will be two-sided and *P*-values <0.05 will be used to define statistical significance. Analysis will be done using SAS and Stata.

9.3.1.1 Analyses populations

Primary analyses will be based on ITT population and secondary supportive analyses will be done on the PP population. Safety analysis will be performed on the safety population.

1. Intention-to-treat (ITT) population: This population consists of all randomised subjects who have a valid outcome;
2. Per-protocol (PP) population: This population is a subset of the ITT population. Subjects with major protocol deviations will be excluded from PP population. Major protocol deviations will be defined in the SAP.
3. Safety population: This population is a subset of the ITT population, consisting of all randomised subjects who receive at least one dose or partial dose of study drug.

9.3.2 Assessment of efficacy

9.3.2.1 Primary endpoint analyses

The generalised linear model (GLM) with the log-link function and binomial distribution (log-binomial regression) will be used to analyse the primary endpoint. The GLM model will have the treatment arm as the only predictor and country (Kenya, Malawi and Tanzania) and gravidity (pauci and multigravidae) as control variables, from which (unadjusted) risk ratio (RR) and its 95% confidence intervals (CI) of having a primary endpoint will be derived. Covariate-adjusted analyses for the primary endpoint will also be conducted by adding pre-specified covariates into the above unadjusted GLM analysis to derive the adjusted RR (95% CI). Imputation for baseline missing covariates will be made for covariate adjusted analysis.

In addition to the final analysis, the primary statistical hypothesis will be tested in an interim analysis when approximately 75% of women have enrolled as described in more detail in section 10.1.3, page 45.

9.3.2.2 Secondary endpoints analyses
These will also be analysed using GLMs with the treatment arm as the single predictor and country (Kenya, Malawi and Tanzania) and gravidity (pauci and multigravidae) as control variables. The point estimate of the treatment effects with 95% CI will be derived via specification of a GLM model for each secondary outcome depending on its distribution. For a binary outcome such as the components of primary endpoint, similar analysis to the primary endpoint analysis will be performed and the treatment effects measured as RR will be generated. For a continuous outcome like birthweight, a Gaussian distribution will be assumed and identity link function will be used. Correspondingly the mean difference and its 95%CI between two arms will be calculated. For a count outcome such as the number of SAEs, Poisson distribution and log link function will be used, from which incidence rate ratio (IRR) and its 95% CI will be computed.

9.3.2.3 Subgroup analyses
We will perform subgroup analyses of the primary endpoint and its components on the following baseline characteristics of women: study site, gravidity (1st, 2nd 3rd and 4th+), malaria status (positive-negative, based on a composite of microscopy and PCR), socio-economic status (terciles), season (terciles of average rainfall last 6 months before delivery), and site characteristics: malaria transmission intensity, expressed as prevalence of PCR positivity on enrolment across 3 arms; and degree of SP parasite resistance, expressed as prevalence of the 'quintuple' and 'sextuple' *dhfr/dhps* mutant parasites or other variations of resistance markers. We will also conduct subgroup analysis for the effect of the number of courses received (e.g. <4 vs 4+). The treatment effect within each category of the above selected variables will be estimated and the interaction effect between treatment and each variable assessed to explore effect modification. The modifying effects of bednets cannot be assessed as all women will receive LLINs on enrolment as part of the national policy.

9.3.3 Safety
For the safety analysis, women in the IPTp-DP arm will be considered overall and by number of DP courses, alone and combined with AZ, received to explore dose-responses using GLM.

9.3.4 Meta-analysis
Subject to the timely availability of individual datasets, a prospective individual participant data (IPD) meta-analysis is planned following the completion of this trial using data from all available trials comparing IPTp-DP with IPTp-SP in Africa. This is currently estimated to involve a total of 4 or 5 trials, including this proposed trial, the 2 completed trials[23,27], and 2 other smaller planned trials in Uganda and Malawi (see section 4.2, Other relevant research ongoing elsewhere, page 21). The statistics for each trial will be combined to estimate the pooled effect size and associated confidence intervals for the meta-analyses. The analysis will follow the new PRISMA-IPD statement guidelines.[74] All PIs have agreed to share their existing and prospective data bases for this purpose.

10 METHODS: MONITORING

10.1 DATA MONITORING AND TRIAL OVERSIGHT COMMITTEES

10.1.1 Data Monitoring and Ethics Committee (DMEC)
Since the study is a clinical trial an independent Data Monitoring and Ethics Committee (DMEC) will be set up. The DMEC will be critical to ensure that the subjects are protected from harm, while also

ensuring that the study integrity is not compromised. The DMEC will consist of 3 to 4 independent members knowledgeable in the conduct of clinical trials. They will meet regularly (e.g. twice yearly or more frequent if so required) during data collection period to provide a review of blinded (and if requested unblinded) data to ensure the safety, rights and well-being of trial participants. In addition, regular review of the quality of the study data will be conducted at each meeting of the DMEC.

The roles and membership of the DMEC are described in more detail in Appendix II. Terms of reference oversight committees, section 16.2, page 71.

10.1.2 Trial Steering Committee (TSC)

The trial will also have a Trial Steering Committee (TSC) which will advise on the study amendments, monitor progress and quality of the trial, ensure women's rights and safety are adhered to, review relevant new information from other sources and consider recommendations from the DMEC. The role and membership of the TSC are also described in more detail in Appendix II. Terms of reference oversight committees, section 16.2, page 71.

10.1.3 Interim sample size re-estimation and/or interim analyses and criteria for termination of the trial

10.1.3.1 Sample size re-estimation

One interim sample size-estimation will be conducted when approximately 50% of women have been recruited to verify if the trial is not under-powered based on potential differences between the observed event and dropout rate pooled across the three arms (i.e. blinded) and the estimates of these pooled rates used in the sample size calculations, which were based on historical data from the study areas. To maintain Type 1 error and trial integrity and safeguard the power of the trial, this will be conducted blinded; i.e. the effect size will not be taken into consideration as no details by arm will be provided to the statistician conducting the sample size re-estimation

10.1.3.2 Interim analysis

An interim analysis will be conducted on the primary endpoint when approximately 75% of participants have enrolled or at a time point suggested by the TSC or DSMB. The interim-analysis will be performed by an independent statistician, blinded for the treatment allocation. The statistician will report to the independent DMEC. The DMEC will have unblinded access to all data and will discuss or report the results of the interim-analysis with the Trial Steering Committee (TSC), e.g. in a joint meeting. The TSC decides on the continuation of the trial and will report to the ethics committees.

The Lan-DeMets spending function with O'Brien-Fleming type boundaries will be employed and the sample size inflated (see 7.6.1.2, page 37) to preserve the overall one-sided type I error rate for efficacy at the α=0.05 level at the final analysis. If the stopping boundary is crossed at the interim analysis and the RR is less than 1, i.e. the observed prevalence of the primary endpoint in the intervention arm is less than the expected prevalence under the null hypothesis, it will be concluded that the study has demonstrated that the efficacy of the intervention is superior to that of IPTp-SP in the prevention of the primary outcome. The trial recruitment can then be stopped unless the DMEC advises otherwise Statistics will not be the sole basis for the decision to stop or continue and the DMEC can advise to continue recruiting in the trial (e.g. in 2 of the 3 arms), or stop recruiting but continue to complete the intervention as per randomization in the remaining active women, even if statistically the stopping boundary is crossed, e.g. in order to continue collecting more safety

information or data for further sub-group analyses etc. The trial will not be stopped in case of futility, unless the DMEC during the course of safety monitoring advises otherwise.

A detailed plan for interim analysis, the provisional stopping rules and how the stopping rules will be applied, will be drawn up prior to the start of the interim analysis and documented in the study statistical analysis plan.

In addition, regular review of the quality of the study data will be conducted at each meeting of the DMEC.

10.2 Safety Monitoring and reporting

The principles of ICH GCP require that both investigators and sponsors follow specific procedures when notifying and reporting adverse events or reactions in clinical trials.

10.2.1 Definitions

The following definitions apply to this protocol:

10.2.1.1 Adverse Event (AE)

Any untoward medical occurrence in a patient or clinical trial subject administered a medicinal product and which does not necessarily have a causal relationship with this treatment.

10.2.1.2 Adverse Reaction (AR)

Any untoward and unintended response to an investigational medicinal product related to any dose administered.

Comment: All adverse events judged by either the reporting investigator or the sponsor as having a reasonable causal relationship to a medicinal product would qualify as adverse reactions. The expression ‘reasonable causal relationship’ means to convey, in general, that there is evidence or argument to suggest a causal relationship.

10.2.1.3 Serious Adverse Event (SAE) or Serious Adverse Reaction (SAR)

Any adverse event or adverse reaction that results in death, is life-threatening*, requires hospitalisation or prolongation of existing hospitalisation, results in persistent or significant disability or incapacity, or is a congenital anomaly or birth defect.

Comment: Medical judgement should be exercised in deciding whether an adverse event/reaction should be classified as serious in other situations. Important adverse events/reactions that are not immediately life-threatening or do not result in death or hospitalisation, but may jeopardise the subject or may require intervention to prevent one of the other outcomes listed in the definition above, should also be considered serious.

*Life-threatening in the definition of a serious adverse event or serious adverse reaction refers to an event in which the subject was at risk of death at the time of the event. It does not refer to an event which hypothetically might have caused death if it were more severe.

10.2.1.4 Suspected Unexpected Serious Adverse Reaction (SUSAR)

An adverse reaction that is both unexpected (not consistent with the applicable product information) and also meets the definition of a Serious Adverse Event/Reaction.

10.2.1.5 Intensity

The intensity of each AE recorded in the case report form should be assigned to a grade (1-5), which will be determined following the definitions set forth in the Common Terminology Criteria for Adverse Events v3.0 (CTCAE) (Cancer Therapy Evaluation Program, 2006). Use of these standardized guidelines will allow for uniform reporting. The grades are defined as follows:

- Grade 1: Mild AE
- Grade 2: Moderate AE
- Grade 3: Severe AE
- Grade 4: Life-threatening or disabling AE
- Grade 5: Death related to AE

10.2.2 Identifying, managing adverse events

Participants who develop adverse events as a consequence of the study interventions or other treatments will be identified at follow-up visits and referred to the designated hospital for evaluation and treatment according to local guidelines. Mild adverse events will be noted in the participant's case report form; no further action will be taken by study staff except in the case of vomiting, in which case the study medication may need to be re-administered. In the case of any SAE, subjects will be referred to the hospital for management. Transportation to the hospital will be provided where feasible or transport reimbursed. All participants with SAEs will undergo record review to identify potential adverse consequences of study participation.

10.2.3 Assessment of causality

The investigator is obligated to assess the relationship between investigational product and the occurrence of each AE/SAE. The investigator will use clinical judgment to determine the relationship. Alternative causes, such as natural history of the underlying diseases, concomitant therapy, other risk factors, and the temporal relationship of the event to the investigational product will be considered and investigated. The investigator will also consult the drug information and the DMEC as needed in the determination of his/her assessment.

There may be situations when an SAE has occurred and the investigator has minimal information to include in the initial report. However, it is very important that the investigator always make an assessment of causality for every event prior to transmission of the SAE report. The investigator may change his/her opinion of causality in light of follow-up information, amending the SAE case report form accordingly.

10.2.4 Reporting adverse event procedures

All SAEs will be reported to the in country principal investigator or an assigned representative within 24 hours of the staff becoming aware of it, using an SAE form, which should be completed and sent electronically. The SAE form asks for nature of event, date of onset, severity, corrective therapies given, outcome and causality (i.e. unrelated, unlikely, possible, probably, definitely). The responsible study clinician should assign the causality of the event.

10.2.4.1 Expedited reporting

SAEs that are unexpected and are at least 'possibly related' to the study drug require expedited reporting within 24 hours of the country principal investigator or assigned representative becoming aware of it (e-mail notification); i.e. this will be a maximum of 48 hours after the event occurred or the study team were made aware of the event (including the 24 hours required for the field staff to report to the principal investigator / representative). Additional information will be sent within 14 additional days (full SAE report) if the reaction had not resolved at the time of e-mail notification.

10.2.4.2 Scheduled reporting

Other SAEs and AEs will be reported annually (or more frequent if so required by the DMEC or ethics committees) in an aggregated report. AEs that will not be reported include common illnesses that do not result in hospitalization, including but not limited to clinical malaria, respiratory, gastrointestinal, and skin diseases, unless they are considered at least possibly related to the intervention.

10.2.4.3 Recipients of reports

The study will comply with local regulations pertaining to reporting of SAEs to their local Research Ethics Committee and/or Research & regulatory offices. In addition to the primary ethics committees, we will report safety data to the DMEC, and to the sponsor. In addition, and if so requested by the manufacturer, the sponsor will forward the reports to the manufacturers of DP (Sigma Tau) and to the manufacturer of AZ. A copy of the final study report will be provided to all study hospitals, ethics committees, TSC, DMEC, to local regulators, and, if so requested, to Sigma Tau and the manufacturer of AZ.

Figure 1: Safety reporting assessment flowchart[75]

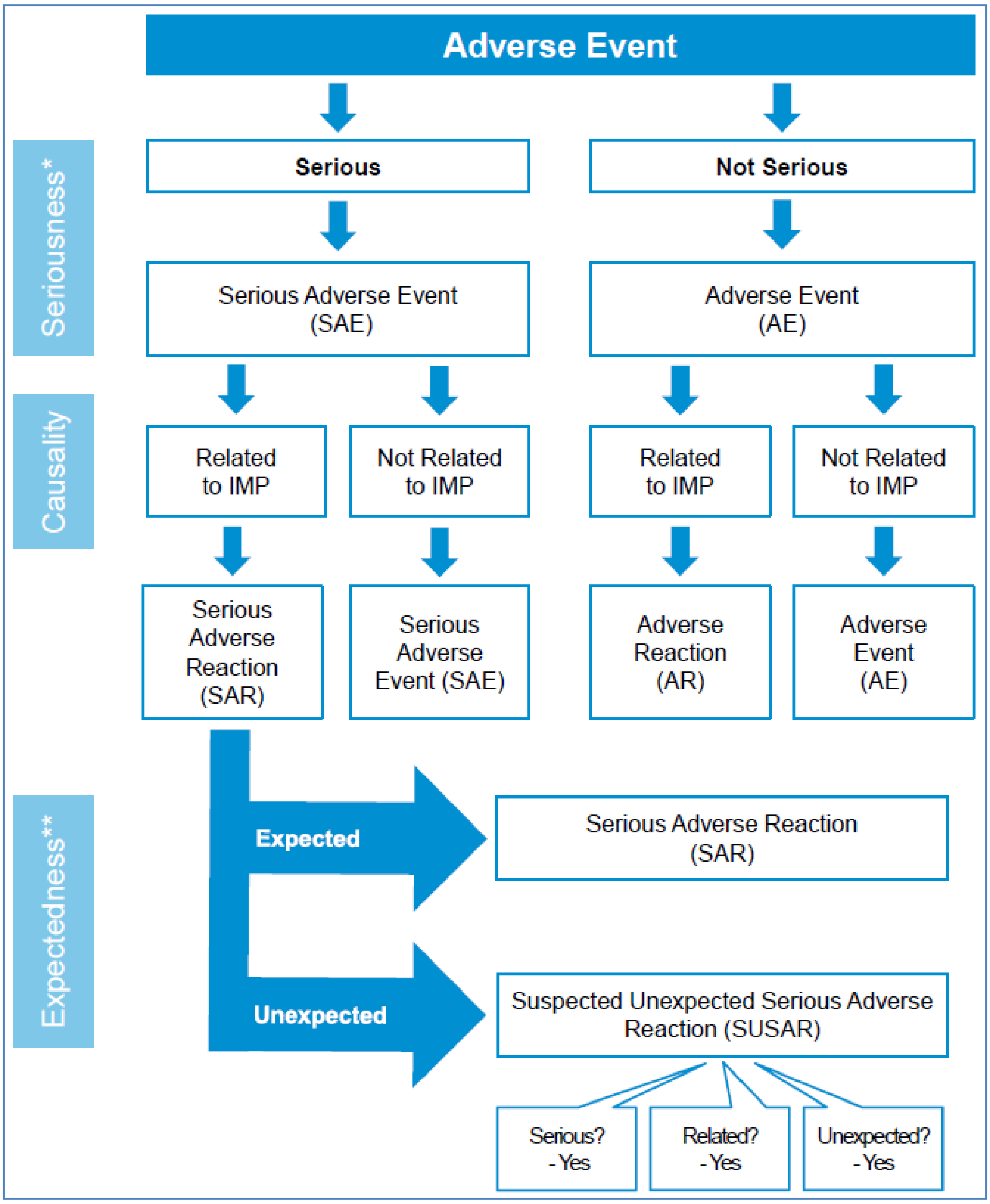

IMP: Investigational Medicinal Product
*See definition of SAE in section 10.2.1
**Assessed in line with the current approved Investigator's Brochure (IB)

10.3 Trial monitoring and auditing

10.3.1 Trial monitoring

External clinical trial monitoring visits are provided by the sponsor at trial initiation, and then regularly (at least yearly) thereafter and at trial close out, or more frequently if so required; e.g. if the trial field work is about 24 months, this means that each site is visited approximately 4 times by external monitors. The results from each monitoring visit will help inform whether more frequent or earlier repeat visits are required. The frequency of these visits appeared appropriate for our experienced study sites in recent trials and the feedback was highly appreciated by the study teams.

10.3.2 Auditing

The independent clinical monitoring process will be audited by a study staff from the sponsor's research office at LSTM in Liverpool, UK. The auditor will accompany the clinical monitor during at least one of the site visits. After this visit it will be determined by the sponsor if more auditing visits are required.

10.3.3 Role of sponsor

The sponsor reserves the right to suspend temporarily or prematurely discontinue this study at any time for reasons including, but not limited to, safety or ethical issues or severe non-compliance. If the sponsor determines such action is needed, it will discuss this with the investigator. When feasible, the sponsor will provide advance notification to the investigator of the impending action prior to it taking effect. The sponsor will promptly inform the ethics committees and provide the reason for the suspension or termination.

10.4 Other quality control measures

10.4.1 Safety monitors

A local paediatrician and/or obstetrician will act as trial safety monitors, one per country.

10.4.2 Internal monitoring

Each of the partner's institutions have their own internal quality control teams who will conduct internal monitoring on a regular basis, and also to help prepare for external monitoring visits.

10.4.3 Training

The country Principal Investigators are responsible for the conduct of the study at the study sites, including delegation of specified study responsibilities, and training of study staff. Each site will maintain a record of all individuals involved in the study (medical, nursing and other staff) and will ensure that all persons assisting with the trial receive the appropriate training about the protocol, the investigational product(s) and their trial-related duties and functions, including formal certified GCP training. During the study the regular spot checks will be conducted to assess the performance of study site staff members and re-training provided where necessary.

10.4.4 Quality assurance/control of laboratory tests

Regular audits of laboratory performance will be completed by experienced supervisors according to standard operating procedures. All malaria blood smears will be read by two different microscopists blinded to the allocation and each other's results, any significantly discordant results based on positive/negative results or difference in parasites above a defined threshold will be verified by a third expert microscopist. All malaria molecular analyses will include a set of positive *P. falciparum* controls and a set of randomly distributed negative controls on the PCR plates. If one or more of the

negative controls are PCR positive the PCR run will be discarded. Ten percent of all histopathology slides will undergo external validation.

11 ETHICS AND DISSEMINATION

11.1 DECLARATION OF HELSINKI

The trial will be conducted in compliance with the principles of the Declaration of Helsinki (1996) (See section 16.3, "Appendix III. Declaration of Helsinki", page 74), the principles of GCP and in accordance with all applicable regulatory requirements in Malawi, Tanzania and Kenya.

11.2 RESEARCH ETHICS AND REGULATORY APPROVAL

11.2.1 Review process

This protocol, the informed consent document, patient information sheets will be reviewed and approved by the Research Ethics Committees at KEMRI, Nairobi, Kenya (SERU), and the College of Medicine in Malawi (COMREC) (or if so requested at national level, by the Malawian National Health Science Research Committee), and by the National Health Research Ethics Committee (NatHREC) in Tanzania. In addition, the protocol will require approval from the Research and Ethics Committee of Liverpool School of Tropical Medicine, Liverpool (LSTM).

11.2.2 Protocol amendments

If it is necessary for the protocol to be amended, the protocol amendment will be submitted to the research ethics committees at LSTM (sponsor) and the primary ethics committees in each country for approval before implementation in that country. Any change to the informed consent form, with the exception of layout, spelling errors and formatting, must also be approved by the sponsor and the primary ethics committee in each country, before the revised form is used.

No change will be made to the approved protocol without the agreement of the sponsor. The Chief Investigator, or a delegated person, will distribute amendments on behalf of the sponsor to each principal investigator, who in turn is responsible for the distribution of these documents to the staff at his/her study site.

11.2.3 Sanctioning of protocol by hospitals

Where this is a requirement of participating hospitals, the approved protocol will subsequently be submitted to the participating hospitals review committees or representatives for subsequent sanctioning of the protocol and procedures by the hospital.

11.2.4 Regulatory approval

Regulatory approval will be sought from the national regulators in Kenya, Malawi and Tanzania form the Pharmacy and Poisons Board; Pharmacy, Medicines and Poisons Board; and Tanzania Food and Drugs Authority, respectively. All three drugs are currently approved under various brand names in each of the three countries as outlined below:

11.2.4.1 Dihydroartemisinin-piperaquine

Eurartesim®, from Sigma Tau is approved by the European Medicines Agency (EMA), the European regulator for the case-management of malaria. Dihydroartemisinin-piperaquine is also registered for use as case-management for clinical malaria in all three countries. The brand registered is DuoCotecxin (from Beijing Holley-Cotec Pharmaceutical Ltd, China). Eurartesim®, from Sigma Tau,

which is the only GMP formulation currently available and the brand that will be used in this study, is registered in Tanzania, but not yet in Kenya or Malawi. All three local regulators of participating countries have previously approved the use of Eurartesim® in recent studies of malaria in pregnancy in Kenya[24], Malawi,[66] and Tanzania.[74]

11.2.4.2 Azithromycin
Zithromax®, from Pfizer is approved by the US Food and Drug Administration (FDA), and azithromycin is also registered for use as antibiotic in all three countries.

11.2.4.3 Sulphadoxine-pyrimethamine
Sulphadoxine-pyrimethamine is approved in all three countries under various brand names.

11.3 INFORMED CONSENT PROCEDURES
Informed consent will be obtained before women are enrolled in the study. See section 7.5.1.1, page 33, and Appendix VI. Participant information sheets and informed consent , section 16.6, page 83, for further details.

11.3.1 Consent procedures
Written, informed consent will be obtained in the local vernacular language. The consent process shall be initiated at the time of enrolment into the study and shall continue throughout the participant's participation. Participants meeting the initial eligibility criteria will have the study explained to them by a member of the study team. If the participant meets the study enrolment criteria, the full consent process will follow, with a written consent form provided. A copy of the informed consent document will be given to the participant for their records, unless they state that they do not wish to have a copy.

For illiterate participants, an independent witness will be present during the informed consent process and will sign the consent form as a witness, while the participant will be asked to indicate consent by use of thumbprint. The participant may withdraw consent at any time throughout the course of the study, and this will be made clear in the informed consent process. A copy of the informed consent document will be given to the caregiver for their records, unless they state that they do not wish to have a copy.

All individuals will be informed that there is no requirement to join the study and that standard medical care will remain the same regardless of study enrolment. If the participant chooses not to be enrolled in the study, they will be allowed to continue with routine care provided by the health facility without any consequences to the expected level of care provided.

11.4 PROTECTION OF PRIVACY AND CONFIDENTIALITY

11.4.1 Privacy
Personal and medical information relating to research participants will be treated as confidential. The risk of disclosure will be minimized by secure storage of documents and use of linked data by replacing personal identifiers with a unique study code to conceal the identity of the participant.

11.4.2 Privacy of individual
Individual data such as RDT tests for malaria and anaemia will be reported to the participant at point of care, to relevant study staff and where appropriate will be recorded in the participants' medical record book in addition to study CRFs.

11.4.3 Confidentiality of data

All information regarding the participants will remain confidential to the extent allowed by law. Unique numerical identifiers will be used for data entry. All screening forms and case report forms will be kept in a secured location with access limited to authorized study staff. Unique numerical identifiers will be used for the computer-based data entry and blood samples. Publications will contain only aggregate data. No identifying information will be included to ensure individual patient anonymization of all data and results made public.

11.5 Declaration of interest

None of the principal investigators have paid consultancies with the pharmaceutical companies involved in the trial, or other competing interest for the overall trial or in each study site.

11.6 Access to source data/documents

In addition to the clinical monitors, authorised representatives of the sponsor/CRO, an IEC/IRB or regulatory authority may visit the study site to perform audits or inspections, including source data verification. The investigator agrees to allow the sponsor and CRO representatives, including the monitor and study safety monitor, the DMEC, the IRB/IEC and regulatory authority direct access to source data and other relevant documents.

11.7 Risks and benefits

11.7.1 Risks to Study Participants

11.7.1.1 Safety of the study drugs in pregnancy

All three drugs (SP, DP and AZ) are currently thought to be safe for the mother and foetus during the second and third trimesters of pregnancy, and have been used extensively in research settings with favourable safety profiles within the proposed doses to be used in the trial. However, adverse events, particularly those associated with the study medication, will be recorded and monitored throughout the trial. The trial may be stopped or temporarily suspended by the sponsor at any stage due to any arising safety concerns.

11.7.1.2 Potential interactions of DP and azithromycin when used for IPTp

There have been no previous IPTp trials with the combination of DP+AZ. The safety and tolerability of the combination was recently investigated as part of the first study of the pharmacokinetic properties of single dose co-administered of azithromycin (1g/day for 3 days) and piperaquine in pregnant women. The study showed that the combination was well tolerated, with no evidence for drug interaction or cardiotoxicity. The authors concluded that IPTp-DP+AZ is a suitable regimen for further assessment.[76]

11.7.1.3 Blood and tissue sampling

All examinations undertaken as part of this study will be non-invasive, with the exception of blood sampling and vaginal swabs. Wherever possible, blood sampling will be minimised and vaginal swabs self-administered by the participants. Blood samples of no more than 10 mL will be taken before enrolment, for eligibility screening/study-specific baseline testing. The sample will be taken at the screening stage because relatively few women are expected to be excluded from the trial based on blood test results (if HIV-positive or severely anaemic). A similar blood sample shall be collected at all scheduled visits, and aliquoted, where necessary, into respective sample tubes for study

investigations. An additional 2 ml venous blood sample taken from women in the DP intervention arms participating in the cardiac safety study on day 2, 4-6 hours after administration of the 3rd intervention dose, and again prior to and 4-6 hours after each course of IPTp.

A blood sample (no more than 10 mL) will be taken at scheduled visits and at birth, and where possible routine care and study-specific samples will be combined in a single blood draw.

Blood sampling may be inconvenient to the participants, and may cause minor discomfort and bruising. In some aspects of the trial, blood sampling has the potential to directly benefit the participants or their babies, as any malaria infection or anaemia detected as a result of the sampling will be treated. In other aspects of the trial, such as parasite genetics, SP and piperaquine resistance markers will not be of direct benefit to the individual woman, but the potential knowledge gained will eventually be used to benefit all pregnant women and their babies in areas with similar characteristics in sub-Saharan Africa.

The volume of blood collected from each participant will be small, a maximum of 90 ml per woman over the course of the study. Only well trained nursing and laboratory staff will be employed on the trial. Only new disposable needles and lancets will be used for blood taking procedures, and these will be safely discarded immediately after their use.

11.7.2 Benefits to study participants

By taking part in this trial, participants will receive either IPTp-SP, IPTp-DP or IPTp-DP+AZ on a monthly dose schedule. All these regimens have either been the routine standard of care (IPTp-SP) or have been shown in early trials to be superior (IPTp-DP) or have the potential to have significantly increased effectiveness with synergy between two safe and effective drugs (IPTp-DP+AZ). Participants who do not attend scheduled appointments will receive reminders and active follow-up. Participants experiencing illness between visits will be seen and treated free of charge as part of the study though this is already routine practice in some of the participating countries, such as in Malawi. As a minimum the participant will benefit from frequent reminders to attend the monthly ANC schedule, reduced waiting times, and ensured availability of study drugs and routine care.

11.7.3 Risk to the population

11.7.3.1 Macrolide resistance

A single dose of AZ (1g) clears ocular strains of chlamydia that cause trachoma. During the past two decades, over 500 million doses of AZ have been distributed in trachoma-endemicareas[37]as part of the WHO-led trachoma elimination programme.[38]Mass distribution has been shown also to reduce the incidence of respiratory infections,[39]diarrhoea,[40]and malaria.[41]These secondary benefits likely underlie the statistically significant reductions in all-cause childhood mortality reported in , where Ethiopia following trachoma treatment campaigns where odds were cut by one-half (OR=0.51; 95% CI: 0.29-0.90) in a clinical trial[42]and 35% (OR=0.35;95% CI: 0.17–0.74) in a cohortstudy.[43]However, there are concerns regarding clonal expansion of pneumococcal multi-locus sequence types following mass treatment in the same setting where four mass treatments of AZ were conducted.[44] Specifically, significant associations were reported between AZ dosing and *ermB*and *mefA/E*genetic resistant determinants responsible for the vast majority of pneumococcal macrolide resistance.

Whether this is of clinical consequence is unknown. Studies of mass treatment with follow-up periods of $\geq$6 months post AZ exposure have found that the prevalence of resistant isolates returns to pre-exposure levels.[45,46] Moreover, there is no evidence of noticeable increases in pneumococcal resistance over time in countries that have participated in mass campaigns.

11.7.3.2 DP resistance

Monthly DP will also exert some drug pressure on piperaquine. Artemisinin-based combination therapies, including DP, have demonstrable parasite resistance in South East Asia and there is concern that resistance will spread to or develop within Africa.[67] Importantly we will monitor for signs that drug pressure is selecting for resistant parasite strains as outlined in section 6.2.7, Rationale for testing parasite genetics and resistance-associate genes, page 24. We will determine if any mutations within these known drug resistance candidates increase as a result of monthly exposure to either SP or DP (piperaquine).

11.7.4 Other ethical considerations

11.7.4.1 Inclusion of young people under the age of 18

This study will include young women aged <18y. Young pregnant women, in tandem with the respective country laws of age of emancipation, will be considered emancipated, therefore being legally able to consent on their own behalf, and to be included in a clinical trial. It is important to include young women in the trial, as adolescents are known to be particularly susceptible to malaria in pregnancy, and are therefore one of the groups that may benefit the most from any improvements to practice to prevent adverse outcomes related to malaria in pregnancy.

11.7.4.2 Long term samples storage for future studies and shipment of samples to external laboratories

With respect to applicable country regulations, written informed consent will be sought from women participating in the trial for long term storage of their samples for future research. Informed consent shall be sought from all women for the shipping of their samples to external laboratories for relevant analyses.

11.8 ANCILLARY AND POST-TRIAL CARE

11.8.1 Health care during the trial

All care directly related to the proper and safe conduct of the trial, and the treatment of immediate adverse events related to trial procedures will be provided free of charge by the study in the study hospitals. The provision of ancillary care beyond that immediately required for conduct of the trial will not be covered by the trial.

11.8.2 Trial insurance

The sponsor will take out trial insurance such that participants enrolled into the study are covered by indemnity for negligent harm and non-negligent harm associated with the protocol. This will include cover for additional health care, compensation or damages whether awarded voluntarily by the Sponsor, or by claims pursued through the courts. The liability of the manufacturer of the trial drugs AZ and DP is limited to those claims arising from faulty manufacturing of the commercial product and not to any aspects of the conduct of the study.

11.8.3 Post-trial care

The study budget is not in a position to fund post-study care or implementation of IPTp-DP with or without AZ as policy. However, the investigators work in close collaboration with local and international policy makers (e.g. WHO) and funders (e.g. President's Malaria Initiative) to ensure that policy makers and funders are informed early of germane research findings.

11.9 EXPENSES REIMBURSEMENT AND INCENTIVES

The study will provide payment for all study drugs, study procedures, study-related visits and reasonable medical expenses that are incurred in study clinics or hospitals as a result of the study, including expenses for transport for any study related visits including unscheduled visits in between scheduled visits to study clinics. The study will not cover the costs of any non-malaria or non-study related events, including scheduled or unscheduled surgery or trauma related events (e.g. accidents, burns etc.) if this is not deemed to be related to the study by the principal investigators or their representative.

Table 3: Reimbursement of expenses and incentives provided by the study

To Who	What	Approximate Amount
Hospital	Improvement of infrastructure where required	up to ~$10,000/hospital
	Training of routine staff adult ward	~ $3,000/hospital
	Study procedure costs and study drugs and admission fees for inpatients	~ $100,000/hospital
Participant (mother or infant)	Travel expenses for participant	Up to ~$10 per round trip*
	Compensation for each day they are scheduled to come to the ANC, out-patient clinic or delivery unit and need to stay or wait for more than 4 hours	~$ 3 per day (excluding any travel expenses)

*In exceptional cases higher amounts of travel expenses can be reimbursed if distance requires. This would need to be decided on a case by case basis, courtesy of the site PI/coordinator.

11.10 DISSEMINATION AND APPLICATION OF THE RESULTS

11.10.1 Result dissemination and publication policy

11.10.1.1 Dissemination to local stakeholders, global policy makers, academic beneficiaries and the general public

Our research findings will be communicated to country level stakeholders including the study participants, reproductive health and malaria programmes/national Ministries of Health, and the research partner institutions in Kenya, Tanzania and Malawi, and to other national and international research partners, NGOs, technical agencies and implementing partners, donor organizations, WHO, UNICEF and the general public. We will use multiple communication strategies to reach each target audience as outlined below.

11.10.1.2 Local Stakeholders

Communications with stakeholders involved in the trials and nested studies (i.e. study participants, health providers and trial staff), Ministries of Health and their technical and implementing partners, and donors in Kenya, Malawi and Tanzania will be through face-to-face meetings and local forum presentations at village, district, regional, county and national levels as may be applicable. The results of the trial and nested studies will be communicated through a dissemination meeting to all national level stakeholders held in one of the three trial countries, preferably linking to the national interagency and multidisciplinary technical committee on malaria (see 11.10.2, Impact, page 57). Policy briefs describing the results in clear, jargon-free language targeting country policy makers will be disseminated to facilitate policy adoption and subsequent implementation, as appropriate.

11.10.1.3 Academic beneficiaries
Communication of trial results to academic beneficiaries will be achieved through standard academic channels including open-access, peer-reviewed journal publications, articles in science review magazines such as International Innovation, symposia, conferences, and the dissemination workshop to multidisciplinary malaria technical committees (which include academics) in Tanzania, Malawi and Kenya. The full de-identified individual patient level dataset from the trial will be made publicly accessible via the WWARN platform (http://www.wwarn.org/working-together/sharing-data/accessing-data). The trial publications will also be disseminated via institutional websites of partners, and to all partners of the LSTM-led Malaria in Pregnancy Consortium by e-mail, their website, and the MiP library, which is the most comprehensive and reliable database of literature on malaria in pregnancy currently available (http://library.mip-consortium.org/). The research will also be disseminated to all academics within each of the participating institutions through monthly and annual dissemination seminars that are held routinely for information sharing and also to encourage networking, cross disciplinary working, and development of new initiatives. The Chief Investigator and Dr Jenny Hill will be responsible for ensuring dissemination in the UK, Dr Pascal Magnussen in Denmark, Dr Robberstad in Norway, Drs Meghna Desai and Julie Gutman for dissemination in the US, and the country PIs in Kenya (Simon Kariuki), Malawi (Mwayiwawo Madanitsa, Victor Mwapasa, Ken Maleta) and Tanzania (Franklin Mosha, John Lusingu).

11.10.1.4 WHO and Roll Back Malaria (RBM) Partnership
Results of the trial will be presented to WHO at a WHO-convened ERG meeting in early 2019 for consideration for policy. If the intervention is recommended for global policy, the research team will support WHO to prepare and disseminate policy guidance information aimed at policy users in sub-Saharan Africa. The results will also be disseminated to the Roll Back Malaria (RBM) Partnership MiP Working Group, responsible for generating consensus among RBM Partners, including national malaria and reproductive health programmes, technical agencies and donors, on key strategic issues and best practices for ensuring effective delivery and scale-up of interventions for the prevention and control of MiP.

11.10.1.5 General Public
Communication to a broader audience and the general public will be via press releases and TV opportunities in the news media in the UK, Kenya, Tanzania and Malawi, news items on websites including partner websites and online networks such as Global Health Network https://globalhealthtrials.tghn.org, and publication portals including the MiP library.

11.10.2 Impact

11.10.2.1 Likelihood to result in major advances for the field of malaria control in pregnancy
The context for the trial is highly significant and timely. Several multi-centre trials for the control of malaria in pregnancy have been completed in the last decade. However, results of the first series of trials have been disappointing; neither amodiaquine, alone or combined with SP,[15] mefloquine (15 mg/kg),[16,18,19]or the fixed dose combination of chloroquine-azithromycin[20,77]are tolerated well enough to replace SP for IPTp in Africa. Furthermore, our recently completed trials with intermittent screening and treatment in pregnancy (ISTp) as alternative strategy to IPTp-SP, showed that ISTp was not superior to IPTp-SP in preventing malaria infections or improving foetal growth even in areas with very high SP resistance.[23,68] The lack of positive results from these trials is a cause of major concern as SP resistance has continued to increase, particularly in areas of East and Southern Africa.

However, the results of the recently completed exploratory trials of IPTp with DP in Kenya and Uganda[23,27]are promising and DP has now emerged as the leading, and so far only, suitable candidate to replace SP for use in IPTp.[26]

11.10.2.2 Improving public health, economic and societal impacts

The ultimate beneficiaries of this research will be mothers and their infants in Kenya, Malawi, Tanzania and other countries experiencing high levels of SP resistance, whose quality of life, health, and creative output will have been enhanced. Malaria poses a substantial economic and social burden in endemic countries, with costs arising at the household level through direct costs of seeking care and indirect (productivity) losses; and at the aggregate level by reducing economic growth. Malaria in pregnancy is estimated to be responsible for 35% of preventable low birth weight, which is a strong predictor of infant mortality, childhood morbidity and productivity in adult life.[13] Identifying more effective means of preventing malaria in pregnancy for these women can reduce part of this burden, and enhance the effectiveness of public investments in malaria control.

We estimate that each year approximately 6.2 million pregnancies will benefit from a switch to the new intervention if found to be superior to SP. These are the pregnancies that occur in highly SP resistant areas in east and southern Africa where the quintuple dhfr/dhps haplotype has reached saturation, representing approximately 24% of all pregnancies in malaria-endemic Africa. About one-third of these (2.0 million) occur in settings with appreciable levels of the sextuple haplotype which severely compromises the effectiveness of SP (Patrick Walker, Imperial College, unpublished observations).

11.10.2.3 Towards integrated strategies for malaria and other curable maternal co-infections

In addition to having the potential to set policy for malaria in pregnancy, the study also benefits from a novel component by determining whether combining DP with the broad spectrum antibiotic azithromycin, can further improve birth outcomes. If the results confirm previous promising findings from trials that combined IPTp-SP with intermittent AZ in pregnancy,[28,29,78] this could potentially pave the way for adoption of control strategies for integrated malaria and curable infections in pregnancy that employ combination treatment to improve birth outcomes. This could have a major impact on reproductive health as suggested by new research just published in *The Lancet* which suggests that malaria is the leading infectious cause of stillbirth worldwide and is responsible for 20%of all stillbirths in sub-Saharan Africa, an estimated 210,000 each year; with syphilis as a close second, responsible for another 200,000 stillbirths in the region.[79]

11.10.2.4 Contribution to strengthening the capacity in sub-Saharan Africa to conduct clinical trials

The development of research capacity of African institutions is central to the achievement of the research objectives and includes training in trial methodologies, including in Good Clinical Practice, and strengthening of the quality assurance/control procedures. The multi-centre trial in three countries will enhance the visibility of the four African research institutions and the potential to improve research quality and the rate at which pivotal research findings can be translated into policy, thereby increasing its impact. The trial will furthermore result in the training of a cohort of one Malawian Post-Doc and four African PhD students, contributing skilled research staff to the national workforce. Health worker capacity in the feasibility study sites in Kenya will also be strengthened through training, thereby advancing knowledge, improving skills and increasing the effectiveness of public health services for pregnant women.

11.10.3 Authorship and publications

Potential authors include all professionals that have participated in the trial for a minimum of four months. Authorship of any presentations or publications arising from this study will also be governed by the principles for authorship criteria of the International Committee of Medical Journal Editors has designed.[80] Disputes regarding authorship will be settled by the publications committee, with further involvement of the independent chair of the TSC if so required. The manufacturer of the study medication will be provided with a draft of the manuscript but will have no role in review, data interpretation, or writing of the article.

11.10.4 Data Sharing Statement

Biological samples and data will be shared using material and data transfer agreements with the collaborating institutions (see 2.2.4 Collaborators, page 7) to minimise the risk of unauthorised analysis beyond the scope of the agreed parameters.

The full protocol will be available on request to any interested professional and may be published in a peer reviewed journals or deposited in an online repository. Individual, de-identified participant data will be made available for meta-analyses as soon as the data analysis is completed, with the understanding that results of the meta-analysis will not be published prior to the results of the individual trial without prior agreement of the investigators. No later than five years after the publication of the trial a fully de-identified data set of the complete patient-level data will be available for sharing purposes, such as via the WWARN repository platform (http://www.wwarn.org/working-together/sharing-data/accessing-data). All requests for data for secondary analysis will be considered by a Data Access Committee to ensure that use of data is within the terms of consent and ethics approval.

12 TIMEFRAME AND DURATION OF THE STUDY

12.1 TIMELINE

The total duration of the project is 48 months, including 36 months for the trial, and 1 additional year for the PhD students to write up their theses. The 36 months trial period includes 9 months for trial preparation and 24 months field work (17 months recruitment plus 7 months of mother-infant follow-up until the child is 6 weeks old), and 3 months for completion of laboratory assays, data analysis and reporting. The 9 months for trial preparation are needed for ethical and regulatory clearances, trial insurance, trial registration, study drug shipment and packing, establishment of a DMEC, development of case-record forms (CRFs) and standard operating procedures (SOPs), and community sensitisation and staff training. The study will include 1 interim analysis of efficacy and safety data when approximately 75% of the patients have been enrolled.

12.1.1 Project gantt chart

	Year 1				Year 2				Year 3				Year 4*			
	Q 1	Q 2	Q 3	Q 4	Q 1	Q 2	Q 3	Q 4	Q 1	Q 2	Q 3	Q 4	Q 1	Q 2	Q 3	Q 4
**Activity**																
Protocol development	■															
IRB approval / Annual Review	■	■	■			■				■						

Regulatory approval/ Annual Review																
CRF and SOP development																
Drug and placebo preparation																
Drug shipment																
Enrolment																
Study								.								
Closure																
Analysis																
Manuscript and results dissemination												.				

13 REFERENCES

1. Desai M, ter Kuile FO, Nosten F, et al. Epidemiology and burden of malaria in pregnancy. *The Lancet Infectious diseases* 2007; **7**(2): 93-104.
2. Desai M, Gutman J, Taylor SM, et al. Impact of Sulfadoxine-Pyrimethamine Resistance on Effectiveness of Intermittent Preventive Therapy for Malaria in Pregnancy at Clearing Infections and Preventing Low Birth Weight. *Clinical infectious diseases : an official publication of the Infectious Diseases Society of America* 2016; **62**(3): 323-33.
3. Chico RM, Cano J, Ariti C, et al. Influence of malaria transmission intensity and the 581G mutation on the efficacy of IPT in pregnancy: systematic review and meta-analysis. *TM & IH* 2015.
4. Harrington WE, Mutabingwa TK, Muehlenbachs A, et al. Competitive facilitation of drug-resistant Plasmodium falciparum malaria parasites in pregnant women who receive preventive treatment. *Proceedings of the National Academy of Sciences of the United States of America* 2009; **106**(22): 9027-32.
5. Minja DT, Schmiegelow C, Mmbando B, et al. Plasmodium falciparum mutant haplotype infection during pregnancy associated with reduced birthweight, Tanzania. *Emerging infectious diseases* 2013; **19**(9).
6. Gutman J, Kalilani L, Taylor S, et al. The A581G Mutation in the Gene Encoding Plasmodium falciparum DHPS Reduces the Effectiveness of Sulfadoxine-Pyrimethamine Preventive Therapy in Malawian Pregnant Women. *The Journal of infectious diseases* 2015; **211**(12): 1997-2005.
7. Naidoo I, Roper C. Mapping 'partially resistant', 'fully resistant', and 'super resistant' malaria. *Trends in parasitology* 2013; **29**(10): 505-15.
8. Harrington WE, Mutabingwa TK, Kabyemela E, Fried M, Duffy PE. Intermittent treatment to prevent pregnancy malaria does not confer benefit in an area of widespread drug resistance. *Clinical infectious diseases : an official publication of the Infectious Diseases Society of America* 2011; **53**(3): 224-30.
9. Harrington WE, Morrison R, Fried M, Duffy PE. Intermittent preventive treatment in pregnant women is associated with increased risk of severe malaria in their offspring. *PloS one* 2013; **8**(2): e56183.
10. Harrington W, McGready R, Muehlenbachs A, Fried M, Nosten F, Duffy P. Intermittent preventive treatment in pregnancy with sulfadoxine-pyrimethamine: the times they are a-changin'. *Clinical infectious diseases : an official publication of the Infectious Diseases Society of America* 2012; **55**(7): 1025-6; author reply 6-7.
11. Harrington WE, Fried M, Duffy PE. Defending the Use of Sulfadoxine-Pyrimethamine for Intermittent Preventive Treatment for Malaria in Pregnancy: A Short-Sighted Strategy. *The Journal of infectious diseases* 2016; **213**(3): 496-7.
12. Gutman J, Taylor S, Meshnick SR, Ter Kuile FO. Reply to Harrington et al. *The Journal of infectious diseases* 2016; **213**(3): 497-8.
13. Gutman J, Kalilani L, Taylor S, et al. The A581G Mutation in the Gene Encoding Plasmodium falciparum Dihydropteroate Synthetase Reduces the Effectiveness of Sulfadoxine-Pyrimethamine Preventive Therapy in Malawian Pregnant Women. *J Infect Dis* 2015; **211**(12): 1997-2005.
14. Nosten F, McGready R. Intermittent presumptive treatment in pregnancy with sulfadoxine-pyrimethamine: a counter perspective. *Malaria journal* 2015; **14**: 248.
15. Clerk CA, Bruce J, Affipunguh PK, et al. A randomized, controlled trial of intermittent preventive treatment with sulfadoxine-pyrimethamine, amodiaquine, or the combination in pregnant women in Ghana. *The Journal of infectious diseases* 2008; **198**(8): 1202-11.
16. Briand V, Bottero J, Noel H, et al. Intermittent treatment for the prevention of malaria during pregnancy in Benin: a randomized, open-label equivalence trial comparing sulfadoxine-pyrimethamine with mefloquine. *The Journal of infectious diseases* 2009; **200**(6): 991-1001.
17. Gonzalez R, Hellgren U, Greenwood B, Menendez C. Mefloquine safety and tolerability in pregnancy: a systematic literature review. *Malaria journal* 2014; **13**: 75.

18. Gonzalez R, Desai M, Macete E, et al. Intermittent preventive treatment of malaria in pregnancy with mefloquine in HIV-infected women receiving cotrimoxazole prophylaxis: a multicenter randomized placebo-controlled trial. *PLoS Med* 2014; **11**(9): e1001735.
19. Gonzalez R, Mombo-Ngoma G, Ouedraogo S, et al. Intermittent preventive treatment of malaria in pregnancy with mefloquine in HIV-negative women: a multicentre randomized controlled trial. *PLoS Med* 2014; **11**(9): e1001733.
20. Chandra RS, Orazem J, Ubben D, Duparc S, Robbins J, Vandenbroucke P. Creative solutions to extraordinary challenges in clinical trials: methodology of a phase III trial of azithromycin and chloroquine fixed-dose combination in pregnant women in Africa. *Malaria journal* 2013; **12**: 122.
21. Kimani J, Phiri K, Kamiza S, et al. Efficacy and Safety of Azithromycin/Chloroquine Versus Sulfadoxine/Pyrimethamine for Intermittent Preventive Treatment of Plasmodium falciparum Malaria Infection in Pregnant Women in Africa: an Open-Label, Randomized Trial. *PloS one* 2016 (in press).
22. Madanitsa M, Kalilani M, L., Mwapasa V, et al. Control of malaria in pregnancy with scheduled intermittent screening with rapid diagnostic tests and treatment with dihydroartemisinin-piperaquine versus intermittent preventive therapy with sulphadoxine-pyrimethamine in Malawi: a randomized clinical trial. Submitted.
23. Desai M, Gutman J, L'Lanziva A, et al. Intermittent screening and treatment or intermittent preventive treatment with dihydroartemisinin-piperaquine versus intermittent preventive treatment with sulfadoxine-pyrimethamine for the control of malaria during pregnancy in western Kenya: an open-label, three-group, randomised controlled superiority trial. *Lancet* 2015; **386**(10012): 2507-19.
24. Group PS, Pekyi D, Ampromfi AA, et al. Four Artemisinin-Based Treatments in African Pregnant Women with Malaria. *N Engl J Med* 2016; **374**(10): 913-27.
25. Tarning J. Treatment of Malaria in Pregnancy. *N Engl J Med* 2016; **374**(10): 981-2.
26. Chico RM, Moss WJ. Prevention of malaria in pregnancy: a fork in the road? *Lancet* 2015.
27. Kakuru A, Jagannathan P, Muhindo MK, et al. Dihydroartemisinin-Piperaquine for the Prevention of Malaria in Pregnancy. *N Engl J Med* 2016; **374**(10): 928-39.
28. Unger HW, Ome-Kaius M, Wangnapi RA, et al. Sulphadoxine-pyrimethamine plus azithromycin for the prevention of low birthweight in Papua New Guinea: a randomised controlled trial. *BMC medicine* 2015; **13**(1): 9.
29. Luntamo M, Kulmala T, Cheung YB, Maleta K, Ashorn P. The effect of antenatal monthly sulphadoxine-pyrimethamine, alone or with azithromycin, on foetal and neonatal growth faltering in Malawi: a randomised controlled trial. *Tropical medicine & international health : TM & IH* 2013; **18**(4): 386-97.
30. van den Broek NR, White SA, Goodall M, et al. The APPLe study: a randomized, community-based, placebo-controlled trial of azithromycin for the prevention of preterm birth, with meta-analysis. *PLoS Med* 2009; **6**(12): e1000191.
31. Heppner DG, Jr., Walsh DS, Uthaimongkol N, et al. Randomized, controlled, double-blind trial of daily oral azithromycin in adults for the prophylaxis of Plasmodium vivax malaria in Western Thailand. *The American journal of tropical medicine and hygiene* 2005; **73**(5): 842-9.
32. Taylor WR, Richie TL, Fryauff DJ, et al. Malaria prophylaxis using azithromycin: a double-blind, placebo-controlled trial in Irian Jaya, Indonesia. *Clinical infectious diseases : an official publication of the Infectious Diseases Society of America* 1999; **28**: 74–81.
33. van Eijk AM, Terlouw DJ. Azithromycin for treating uncomplicated malaria. *The Cochrane database of systematic reviews* 2011; (2): CD006688.
34. Chico RM, Hack BB, Newport MJ, Ngulube E, Chandramohan D. On the pathway to better birth outcomes? A systematic review of azithromycin and curable sexually transmitted infections. *Expert review of anti-infective therapy* 2013; **11**(12): 1303-32.
35. Chico RM, Mayaud P, Ariti C, Mabey D, Ronsmans C, Chandramohan D. Prevalence of malaria and sexually transmitted and reproductive tract infections in pregnancy in sub-Saharan Africa: a systematic review. *Jama* 2012; **307**(19): 2079-86.

36. World Health Organization. Prevalence and Incidence of Selected Curable Sexually Transmitted Infections: Overview and Estimates. . Geneva, 2001.
37. Global Partners Announce Donation of 500 Millionth Dose of Azithromycin, Marking Exceptional Progress to Help Alleviate the Suffering from Trachoma. Reuters News. 2015 16 November.
38. World Health Organization. Prevention of blindness and deafness. Global initiative for the elimination of avoidable blindness. Geneva, Switerland: WHO; 2000.
39. Coles CL, Levens J, Seidman JC, Mkocha H, Munoz B, West S. Mass distribution of azithromycin for trachoma control is associated with short-term reduction in risk of acute lower respiratory infection in young children. *The Pediatric infectious disease journal* 2012; **31**(4): 341-6.
40. Coles CL, Seidman JC, Levens J, Mkocha H, Munoz B, West S. Association of mass treatment with azithromycin in trachoma-endemic communities with short-term reduced risk of diarrhea in young children. *The American journal of tropical medicine and hygiene* 2011; **85**(4): 691-6.
41. Schachterle SE, Mtove G, Levens JP, et al. Short-term malaria reduction by single-dose azithromycin during mass drug administration for trachoma, Tanzania. *Emerging infectious diseases* 2014; **20**(6): 941-9.
42. Porco TC, Gebre T, Ayele B, et al. Effect of mass distribution of azithromycin for trachoma control on overall mortality in Ethiopian children: a randomized trial. *Jama* 2009; **302**(9): 962-8.
43. Keenan JD, Ayele B, Gebre T, et al. Childhood mortality in a cohort treated with mass azithromycin for trachoma. *Clinical infectious diseases : an official publication of the Infectious Diseases Society of America* 2011; **52**(7): 883-8.
44. Keenan JD, Klugman KP, McGee L, et al. Evidence for clonal expansion after antibiotic selection pressure: pneumococcal multilocus sequence types before and after mass azithromycin treatments. *The Journal of infectious diseases* 2015; **211**(6): 988-94.
45. Leach AJ, Shelby-James TM, Mayo M, et al. A prospective study of the impact of community-based azithromycin treatment of trachoma on carriage and resistance of Streptococcus pneumoniae. *Clinical infectious diseases : an official publication of the Infectious Diseases Society of America* 1997; **24**(3): 356-62.
46. Batt SL, Charalambous BM, Solomon AW, et al. Impact of azithromycin administration for trachoma control on the carriage of antibiotic-resistant Streptococcus pneumoniae. *Antimicrobial agents and chemotherapy* 2003; **47**(9): 2765-9.
47. WHO Malaria Policy Advisory Committee. Malaria Policy Advisory Committee to the WHO: conclusions and recommendations of eighth biannual meeting (September 2015). *Malaria journal* 2016; **15**(1): 117.
48. Benjamin JM, Moore BR, Salman S, et al. Population pharmacokinetics, tolerability, and safety of dihydroartemisinin-piperaquine and sulfadoxine-pyrimethamine-piperaquine in pregnant and nonpregnant Papua New Guinean women. *Antimicrobial agents and chemotherapy* 2015; **59**(7): 4260-71.
49. Moore BR, Salman S, Benjamin J, et al. Pharmacokinetics of piperaquine transfer into the breast milk of Melanesian mothers. *Antimicrobial agents and chemotherapy* 2015; **59**(7): 4272-8.
50. Moore KA, Simpson JA, Paw MK, et al. Safety of artemisinins in first trimester of prospectively followed pregnancies: an observational study. *The Lancet Infectious diseases* 2016.
51. Tarning J, Rijken MJ, McGready R, et al. Population pharmacokinetics of dihydroartemisinin and piperaquine in pregnant and nonpregnant women with uncomplicated malaria. *Antimicrobial agents and chemotherapy* 2012; **56**(4): 1997-2007.
52. Rijken MJ, McGready R, Phyo AP, et al. Pharmacokinetics of dihydroartemisinin and piperaquine in pregnant and nonpregnant women with uncomplicated falciparum malaria. *Antimicrobial agents and chemotherapy* 2011; **55**(12): 5500-6.
53. Rijken MJ, McGready R, Boel ME, et al. Dihydroartemisinin-piperaquine rescue treatment of multidrug-resistant Plasmodium falciparum malaria in pregnancy: a preliminary report. *The American journal of tropical medicine and hygiene* 2008; **78**(4): 543-5.

54. Mytton OT, Ashley EA, Peto L, et al. Electrocardiographic safety evaluation of dihydroartemisinin piperaquine in the treatment of uncomplicated falciparum malaria. *The American journal of tropical medicine and hygiene* 2007; **77**(3): 447-50.
55. Keating G. Dihydroartemisinin/Piperaquine: A Review of its Use in the Treatment of Uncomplicated Plasmodium falciparum Malaria. *Drugs* 2012; **72**(7): 937-61.
56. Desai M, Gutman J, L'Lanziva A, et al. Intermittent screening and treatment or IPT with dihydroartemisinin-piperaquine versus IPT with SP for the control of malaria during pregnancy in western Kenya: an open-label, three-group, randomised controlled superiority trial. *Lancet* 2015; **386**(10012): 2507-19.
57. Kakuru A, Jagannathan P, Muhindo MK, et al. Dihydroartemisinin-piperaquine for the prevention of malaria in pregnancy. *New Engl J Med* 2016; **374**(10): 928-39.
58. Ter Kuile FO. ISRCTN34010937 DOI 10.1186/ISRCTN34010937 STOPMiP: Intermittent Screening and Treatment Or intermittent Preventive therapy for the control of Malaria in Pregnancy in Indonesia (http://www.isrctn.com/ISRCTN34010937). 20132013).
59. Bigira V, Kapisi J, Clark TD, et al. Protective efficacy and safety of three antimalarial regimens for the prevention of malaria in young ugandan children: a randomized controlled trial. *PLoS Med* 2014; **11**(8): e1001689.
60. Capan M, Mombo-Ngoma G, Makristathis A, Ramharter M. Anti-bacterial activity of intermittent preventive treatment of malaria in pregnancy: comparative in vitro study of sulphadoxine-pyrimethamine, mefloquine, and azithromycin. *Malaria journal* 2010; **9**(1): 303.
61. Prendergast A, Kelly P. Enteropathies in the developing world: neglected effects on global health. *Am J Trop Med Hyg* 2012; **86**(5): 756-63.
62. Cho I, Yamanishi S, Cox L, et al. Antibiotics in early life alter the murine colonic microbiome and adiposity. *Nature* 2012; **488**(7413): 621-6.
63. Oliver RS, Lamont RF. Infection and antibiotics in the aetiology, prediction and prevention of preterm birth. *J Obstet Gynaecol* 2013; **33**(8): 768-75.
64. ter Kuile FO, van Eijk AM, Filler SJ. Effect of sulfadoxine-pyrimethamine resistance on the efficacy of intermittent preventive therapy for malaria control during pregnancy: a systematic review. *Jama* 2007; **297**(23): 2603-16.
65. Gamble C, Ekwaru JP, ter Kuile FO. Insecticide-treated nets for preventing malaria in pregnancy. *The Cochrane database of systematic reviews* 2006; (2): CD003755.
66. Gimnig JE, MacArthur JR, M'Bang'ombe M, et al. Severe cutaneous reactions to sulfadoxine-pyrimethamine and trimethoprim-sulfamethoxazole in Blantyre District, Malawi. *The American journal of tropical medicine and hygiene* 2006; **74**(5): 738-43.
67. Ashley EA, Dhorda M, Fairhurst RM, et al. Spread of artemisinin resistance in Plasmodium falciparum malaria. *N Engl J Med* 2014; **371**(5): 411-23.
68. Madanitsa M, Kalilani M, L., Mwapasa V, et al. Control of malaria in pregnancy with scheduled intermittent screening with rapid diagnostic tests and treatment with dihydroartemisinin-piperaquine versus intermittent preventive therapy with sulphadoxine-pyrimethamine in Malawi: a randomized clinical trial. Submitted.
69. Baraka V, Ishengoma DS, Fransis F, et al. High-level Plasmodium falciparum sulfadoxine-pyrimethamine resistance with the concomitant occurrence of septuple haplotype in Tanzania. *Malaria journal* 2015; **14**(1): 439.
70. World Health Organization. Guidelines for the treatment of malaria, third edition: World Health Organization,; 2015.
71. Villar J, Cheikh Ismail L, Victora CG, et al. International standards for newborn weight, length, and head circumference by gestational age and sex: the Newborn Cross-Sectional Study of the INTERGROWTH-21st Project. *Lancet* 2014; **384**(9946): 857-68.
72. Schmiegelow C, Minja D, Oesterholt M, et al. Malaria and fetal growth alterations in the 3(rd) trimester of pregnancy: a longitudinal ultrasound study. *PloS one* 2013; **8**(1): e53794.

73. Sutton AJ, Cooper NJ, Jones DR, ..., Thompson JR, Abrams KR. Evidence-based sample size calculations based upon updated meta-analysis. *Stat Med* 2007; **26**(12): 2479-500.
74. Stewart LA, Clarke M, Rovers M, et al. Preferred Reporting Items for Systematic Review and Meta-Analyses of individual participant data: the PRISMA-IPD Statement. *Jama* 2015; **313**(16): 1657-65.
75. Research NIfH. CT-toolokit. 2014. http://www.ct-toolkit.ac.uk/glossary/suspected-unexpected-serious-adverse-reactions-susar (accessed 02 July 2016 2016).
76. Moore BR, Benjamin JM, Auyeung SO, et al. Safety, tolerability and pharmacokinetic properties of co-administered azithromycin and piperaquine in pregnant Papua New Guinean women. *Br J Clin Pharmacol* 2016.
77. Kimani J, Phiri K, Kamiza S, et al. Azithromycin/chloroquine (AZCQ) versus sulfadoxine/pyrimethamine (SP) in intermittent preventive treatment of falciparum malaria infection in pregnant women (IPTp) in Sub-Saharan Africa: an open-label randomized trial. ePoster EP168. European Congress of Clinical Microbiology and Infectious Diseases. Copenhagen, Denmark; 2015.
78. Luntamo M, Kulmala T, Mbewe B, Cheung YB, Maleta K, Ashorn P. Effect of repeated treatment of pregnant women with sulfadoxine-pyrimethamine and azithromycin on preterm delivery in Malawi: a randomized controlled trial. *The American journal of tropical medicine and hygiene* 2010; **83**(6): 1212-20.
79. Lawn JE, Blencowe H, Waiswa P, et al. Stillbirths: rates, risk factors, and acceleration towards 2030. *Lancet* 2016; **387**(10018): 587-603.
80. Uniform requirements for manuscripts submitted to biomedical journals: Writing and editing for biomedical publication. *Journal of Pharmacology & Pharmacotherapeutics* 2010; **1**(1): 42-58.
81. Unger HW, Wangnapi RA, Ome-Kaius M, et al. Azithromycin-containing intermittent preventive treatment in pregnancy affects gestational weight gain, an important predictor of birthweight in Papua New Guinea - an exploratory analysis. *Matern Child Nutr* 2016; **12**(4): 699-712.
82. Ledger WJ, Blaser MJ. Are we using too many antibiotics during pregnancy? *BJOG : an international journal of obstetrics and gynaecology* 2013; **120**(12): 1450-2.
83. Mueller NT, Rifas-Shiman SL, Blaser MJ, Gillman MW, Hivert MF. Association of prenatal antibiotics with foetal size and cord blood leptin and adiponectin. *Pediatr Obes* 2016.
84. Black RE, Victora CG, Walker SP, et al. Maternal and child undernutrition and overweight in low-income and middle-income countries. *Lancet* 2013; **382**(9890): 427-51.
85. Christian P, Mullany LC, Hurley KM, Katz J, Black RE. Nutrition and maternal, neonatal, and child health. *Seminars in perinatology* 2015; **39**(5): 361-72.
86. Garner P, Smith T, Baea M, Lai D, Heywood P. Maternal nutritional depletion in a rural area of Papua New Guinea. *Tropical and geographical medicine* 1994; **46**(3): 169-71.
87. Khlangwiset P, Shephard GS, Wu F. Aflatoxins and growth impairment: a review. *Critical reviews in toxicology* 2011; **41**(9): 740-55.
88. CDC. National Health and Nutrition Examination Survey (NHANES) - Anthropometry Procedures Manual. CDC; 2007.
89. Fattah C, Farah N, Barry SC, O'Connor N, Stuart B, Turner MJ. Maternal weight and body composition in the first trimester of pregnancy. *Acta Obstet Gynecol Scand* 2010; **89**(7): 952-5.
90. Scholl PF, Turner PC, Sutcliffe AE, et al. Quantitative Comparison of Aflatoxin B₁ Serum Albumin Adducts in Humans by Isotope Dilution Mass Spectrometry and ELISA. *Cancer Epidemiology Biomarkers & Prevention* 2006; **15**(4): 823.
91. Wooden J, Kyes S, Sibley CH. PCR and strain identification in Plasmodium falciparum. *Parasitology today (Personal ed)* 1993; **9**(8): 303-5.
92. Hofmann N, Mwingira F, Shekalaghe S, Robinson LJ, Mueller I, Felger I. Ultra-sensitive detection of Plasmodium falciparum by amplification of multi-copy subtelomeric targets. *PLoS Med* 2015; **12**(3): e1001788.
93. Organisation WH. Recommended Genotyping Procedures (RGPs) to identify parasite populations, 2007.

94. Nag S DMD, Kofoed PE, Ursing J, O'Brien.L, Aarestrup FM, et al. High through-put resistance profiling of P. falciparum infections based on filter paper samples, custom dual indexing and Illumina NGS-technology reveals increase in molecular markers of resistance towards sulfadoxine-pyrimethamine, lumefantrine and quinine in Guinea-Bissau. 2016.
95. Alifrangis M, Enosse S, Pearce R, et al. A simple, high-throughput method to detect Plasmodium falciparum single nucleotide polymorphisms in the dihydrofolate reductase, dihydropteroate synthase, and P. falciparum chloroquine resistance transporter genes using polymerase chain reaction- and enzyme-linked immunosorbent assay-based technology. *The American journal of tropical medicine and hygiene* 2005; **72**(2): 155-62.
96. Lozupone C, Knight R. UniFrac: a new phylogenetic method for comparing microbial communities. *Applied and environmental microbiology* 2005; **71**(12): 8228-35.
97. Chico RM, Mayaud P, Ariti C, Mabey D, Ronsmans C, Chandramohan D. Prevalence of Malaria and Sexually Transmitted and Reproductive Tract Infections in Pregnancy in Sub-Saharan Africa: A Systematic Review. *Journal of the American Medical Association (JAMA)* 2012; **307**(19): 2079-86.
98. Recommendations for the laboratory-based detection of Chlamydia trachomatis and Neisseria gonorrhoeae--2014. *MMWR Recommendations and reports : Morbidity and mortality weekly report Recommendations and reports / Centers for Disease Control* 2014; **63**(Rr-02): 1-19.
99. Nugent RP, Krohn MA, Hillier SL. Reliability of diagnosing bacterial vaginosis is improved by a standardized method of gram stain interpretation. *J Clin Microbiol* 1991; **29**(2): 297-301.
100. Meehan M, Wawer M, Swerwadda D, Gray R, Quinn T. Laboratory methods for the diagnosis of reprodutive tract infections and selecged conditions in population-based studies. In: Jejeebhoy S, Koenig M, Elias C, eds. Reproductive Tract Infections and other Gynaecological Disorders. Cambridge: Cambridge University Press; 2003: 261-82.
101. Arthur JC, Perez-Chanona E, Muhlbauer M, et al. Intestinal inflammation targets cancer-inducing activity of the microbiota. *Science (New York, NY)* 2012; **338**(6103): 120-3.
102. Faith JJ, Guruge JL, Charbonneau M, et al. The long-term stability of the human gut microbiota. *Science (New York, NY)* 2013; **341**(6141): 1237439.
103. Underwood MA, Kalanetra KM, Bokulich NA, et al. A comparison of two probiotic strains of bifidobacteria in premature infants. *The Journal of pediatrics* 2013; **163**(6): 1585-91.e9.
104. Knights D, Parfrey LW, Zaneveld J, Lozupone C, Knight R. Human-associated microbial signatures: examining their predictive value. *Cell host & microbe* 2011; **10**(4): 292-6.
105. Conklin LM, Bigogo G, Jagero G, et al. High Streptococcus pneumoniae colonization prevalence among HIV-infected Kenyan parents in the year before pneumococcal conjugate vaccine introduction. *BMC Infect Dis* 2016; **16**: 18.
106. Atwell JE, Thumar B, Robinson LJ, et al. Impact of Placental Malaria and Hypergammaglobulinemia on Transplacental Transfer of Respiratory Syncytial Virus Antibody in Papua New Guinea. *The Journal of infectious diseases* 2016; **213**(3): 423-31.
107. Moro L, Bardaji A, Nhampossa T, et al. Malaria and HIV infection in Mozambican pregnant women are associated with reduced transfer of antimalarial antibodies to their newborns. *The Journal of infectious diseases* 2015; **211**(6): 1004-14.
108. Ogolla S, Daud, II, Asito AS, et al. Reduced Transplacental Transfer of a Subset of Epstein-Barr Virus-Specific Antibodies to Neonates of Mothers Infected with Plasmodium falciparum Malaria during Pregnancy. *Clinical and vaccine immunology : CVI* 2015; **22**(11): 1197-205.
109. Piriou E, Kimmel R, Chelimo K, et al. Serological evidence for long-term Epstein-Barr virus reactivation in children living in a holoendemic malaria region of Kenya. *Journal of medical virology* 2009; **81**(6): 1088-93.
110. Chung AW, Kumar MP, Arnold KB, et al. Dissecting Polyclonal Vaccine-Induced Humoral Immunity against HIV Using Systems Serology. *Cell* 2015; **163**(4): 988-98.
111. Su LF, Han A, McGuire HM, Furman D, Newell EW, Davis MM. The promised land of human immunology. *Cold Spring Harbor symposia on quantitative biology* 2013; **78**: 203-13.

112. Tippett E, Fernandes LA, Rogerson SJ, Jaworowski A. A novel flow cytometric phagocytosis assay of malaria-infected erythrocytes. *J Immunol Methods* 2007.
113. Burton GJ, Charnock-Jones DS, Jauniaux E. Regulation of vascular growth and function in the human placenta. *Reproduction (Cambridge, England)* 2009; **138**(6): 895-902.
114. Karimu AL, Burton GJ. The distribution of microvilli over the villous surface of the normal human term placenta is homogenous. *Reproduction, fertility, and development* 1995; **7**(5): 1269-73.
115. Chisholm KM, Heerema-McKenney A, Tian L, et al. Correlation of preterm infant illness severity with placental histology. *Placenta* 2016; **39**: 61-9.
116. Ogunyemi D, Murillo M, Jackson U, Hunter N, Alperson B. The relationship between placental histopathology findings and perinatal outcome in preterm infants. *The journal of maternal-fetal & neonatal medicine : the official journal of the European Association of Perinatal Medicine, the Federation of Asia and Oceania Perinatal Societies, the International Society of Perinatal Obstet* 2003; **13**(2): 102-9.
117. Conroy AL, Silver KL, Zhong K, et al. Complement activation and the resulting placental vascular insufficiency drives fetal growth restriction associated with placental malaria. *Cell host & microbe* 2013; **13**(2): 215-26.

14 Financial aspects and conflict of interest

14.1 Funding of the trial

Funding to conduct the trial is provided by:

1. The Joint Global Health Trials Scheme of the UK Medical Research Council (MRC/ Wellcome Trust of Great Britain (WT) and the UK Department of International Development (DFID).
2. The European and Developing Countries Clinical trials Partnership (EDCTP 2) which is funded by the European Union (EU), European Participating States and third parties including Product Development Partners (PDPs), Private Sector Industry, and International Development Partners.

The funders had no role in the design of this trial and will not have any during the execution, analysis, interpretation of the data, or decision to submit the results.

14.2 Provision of study drugs

DP and DP placebo will be provided free of charge by Sigma Tau. AZ and matching placebo will be purchased or donated free of charge by Pfizer or another GMP manufacturer of the generic formulation. SP will be obtained from a GMP manufacturer identified in section 7.3.1.3. When so requested by manufacturers, the study will provide copies of safety reports of SAEs and AEs to the manufacturers (expedited where required). The manufacturers will not be involved in the design of the trial.

15 Budget & budget Justification

See Appendix IV. Budget and budget justification.

16 APPENDICES

16.1 APPENDIX I. ROLE OF INVESTIGATORS AND COLLABORATORS

16.1.1 Definition investigator and collaborator

16.1.1.1 Investigators

Investigators are directly involved with the field research and, with the exception of the trial statisticians, live and work in Kenya, Malawi or Tanzania.

16.1.1.2 Collaborators

Collaborators are not involved with any of the field work and are either PhD supervisors living overseas, or support the study with the laboratory assays in their overseas laboratories. Each home institutions will sign MOUs in the form of MTA and/or DTAs with the overseas collaborators before samples or data can be shipped overseas.

16.1.2 Protocol development: authors' contributions

Mwayiwawo Madanitsa (MM) and FtK wrote the first draft of the protocol. All investigators contributed to the refinement of the study protocol and approved the final versions.

16.1.3 Role Investigators

16.1.3.1 Chief Investigator

Prof Feiko Ter Kuile (MD, PhD), is a Professor of Tropical Epidemiology from the Liverpool School of Tropical Medicine (LSTM) based at KEMRI in Kisumu. He is the Chief Investigator and grant holder and will carry overall responsibility for the coordination of the trial and for the linkages with the sponsor, funders and with international partners involved. He will also liaise with WHO.

16.1.3.2 Co-principal investigators

Dr Mwayiwawo Madanitsa (MD, PhD), is a postdoctoral research fellow with COM and LSTM, based at COM in Blantyre, Malawi. He will act as the co-Principal Investigator for Malawi and as overall trial Manager. He will a l s o liaise with the local hospitals, the ethics committees, the regulators and the MoH in country.

He will be supervised locally by Professor Victor Mwapasa and internationally by FtK.

Dr Simon Kariuki (PhD), is the KEMRI/CDC Malaria Branch Chief and will be the co-Principal Investigator for Kenya. He will a l s o liaise with the local hospitals, the ethics committees, the regulators and the MoH in country.

Prof John Lusingu (MD, PhD) is a Visiting Professor of the University of Copenhagen, Denmark and a Principal Research Scientist at the National Institute for Medical Research in Tanzania. He will be the co-Principal investigator of two of the four sites in Tanzania at the NIMR Tanga Center. He willa l s o liaise with the local hospitals, the ethics committees, the regulators and the Ministry of Health, Gender, Children and Elderly (MoH) in Tanzania. He will receive local support by Drs Christentze Schmiegelow and Daniel Minja.

Prof Frank Mosha (PhD) will be the principal investigator for 2 sites in Tanzania at the KCMC. He will also liaise with the local hospitals, the ethics committees, the regulators and the MoH in country. He will be supported by Assistant Professor Matthew Chico.

16.1.3.3 Co-investigators

Prof Duolao Wang, PhD [DW] is Professor of Medical Statistics at the Liverpool School of Tropical Medicine and will be the trial statistician.

Prof Victor Mwapasa (MD,MPH, PhD), is a Professor of Public Health and Dean of Research and Post-graduate Studies at the CoM. He will serve as the co-investigator in Malawi and provide site co-leadership and liaison with administrative and policy stakeholders of the trial, and co-lead the capacity development activities at the College of Medicine, University of Malawi.

Prof Kamija Phiri (MD, PhD), is Professor in Clinical Epidemiology, Public Health Department, and Dean of the School of Public Health & Family Medicine, and Director, of the Training & Research Unit of Excellence (TRUE), College of Medicine, University of Malawi. He has over 10 years' experience in clinical research and provide site co-leadership for the study site in Zomba, Malawi.

Prof Ken Maleta (MD PhD) is Professor in Community Health, College of Medicine, University of Malawi. He is nutritional and growth expert and will support the activities in the Mangochi site in Malawi.

Dr Dianna Terlouw (MD, PhD), is a lecturer in malaria epidemiology from the LSTM, based at the Malawi-Liverpool Wellcome Trust Clinical Research Programme, Malawi. She will support the activities in the Chikwawa site in Malawi and linkages with the MLW program.

Dr Daniel Minja (PhD) is a research scientist with the NIMR Tanzania. He will support Prof John Lusingu at the NIMR Tanga Centre sites

Dr Christentze Schmiegelow (MD, PhD) is a postdoctoral research fellow with the University of Copenhagen. She will support Prof John Lusingu at the NIMR Tanga Centre sites.

Dr Jacklin Mosha (MD, MSc) is an epidemiologist at the NIMR, Tanzania. She will coordinate the research activities that fall under the mandate of the Kilimanjaro Christian Medical Centre, Tanzania.

16.1.4 Collaborators

Non-engaged collaborators are not classified as investigators and have an advisory and supporting role and are not directly involved with the research activities on site.

16.2 Appendix II. Terms of reference oversight committees

16.2.1 Trial Management Group (TMG)

16.2.1.1 Purpose
The TMG is responsible for the administrative management and day to day running of the trial.

16.2.1.2 Membership
1. Overall trial manager (Dr Mwayi Madanitsa)
2. Other co-Principal Investigators (3x) or representatives
3. Country trial Coordinators (4x)
4. Lead Data manager
5. Lead administrators
6. Chief Investigator (ad hoc)
7. Others who are involved in the day to day running of the trial are invited ad hoc

16.2.1.3 Responsibilities:
- Study planning
- Organisation of Trial Steering Committee and Data Monitoring and Ethics Committee (DMEC) meetings
- Provide risk report to regulators, manufactures and ethics committees
- SUSAR [Serious unexpected suspected adverse events] reporting
- Responsible for trial master file
- Budget administration and contractual issues
- Advice for lead investigators
- Organisation of central data management and sample collection

16.2.2 Trial Steering Committee (TSC)

16.2.2.1 Membership TSC

16.2.2.1.1 Independent members
1. Chair: Associate Professor Miriam Laufer, (PhD)
University of Maryland, School of Medicine, Baltimore, USA (paediatrician, and malaria epidemiologist and public health expert with specific expertise in pregnancy, WHO ERG member);
2. Professor Grant Dorsey, MD, PhD University of California, San Francisco (malaria epidemiologist and expert on intermittent DP, CI of previous trial in Uganda[27] and current trial in Uganda).
3. Professor Per Ashorn, MD, PhD, Department of Maternal, Child, and Adolescent Health, World Health Organization, Geneva, Switzerland (paediatrician, infectious disease and growth and nutrition expert and CI of previous intervention trials in pregnancy women using intermittent azithromycin combined with IPTp).
4. Dr Matt Cairns, from LSHTM, will be TSC statistician.
5. Professor Alex Sutton, statistician, will be ad hoc advisor for the simulation based sample size calculation/meta-analysis.

16.2.2.1.2 Trial members
- Prof Feiko ter Kuile, chief Investigator

- Dr Mwayi Madanitsa, representing the co-principal investigators
- Prof Duolao Wang, trial statistician
- The other co-PIs, co-investigators, collaborators and the trial statistician will attend the meetings if and when required.

16.2.2.1.3 Observers

- EDCTP
- MRC

16.2.2.2 Roles and Responsibilities TSC

The TSC is a trial governing body which includes a majority of its members who are independent of the trial management group. The TSC concentrates on the progress of the trial and ensures that the trial is conducted to the standards set out in the Guidelines for Good Clinical Practice with consideration given to participant safety and provision of informed consent.

- To evaluate the progress of the trial in relation to timeliness, data quality and other factors that can affect the overall objectives of the trial
- To ensure participant rights and safety are adhered to and that the protocol demands freely given informed consent
- To review relevant information from other sources
- To consider the recommendations of the Data Monitoring and Ethics Committee (DMEC) and in light of it to inform the Chief Investigator and TMG the need to changes to the trial protocol
- To ensure that the trial results are disseminated appropriately and consideration be given to the implementation of the results into policy

16.2.2.3 Operational TSC

The CI will present the full protocol to the TSC as an agenda before the start-up of data collection. The TSC members shall review the timeline set out in the protocol for participant recruitment, informed consent documents and plans for data safety monitoring.

The TSC shall see that the finalised protocol is sent to the sponsor and funders before the start of participant recruitment and data collection.

The TSC in its first meeting shall approve the nominated members of the DMEC and establish the DMEC which shall meet regularly to review and report on the data quality and the results of interim analyses.

In all their deliberations the TSC should consider any deviations from the trial protocol, participant safety and information provided to the participants and consenting procedures.

16.2.2.4 Frequency of Meetings

The TSC shall have an initial face to face start up meeting to discuss the protocol and establish the DMEC. A second meeting shall take place before the initiation of the trial to finalise the protocol and approve the commencement of the trial. Thereafter the TSC will normally meet once a year in the life span of the trial and one meeting at the closure of the trial.

The Chair and at least 2 of the three independent members together with the CI and trial co-ordinator shall constitute the quorum. If so required, in addition a member of the funder can be invited to attend the meetings.

16.2.2.5 Trial Reports and actions TSC meeting
The TSC shall provide at each meeting a summary report of their findings and recommendations which must be submitted to the funder, the Sponsor and the TMG.

If the TSC makes a recommendation that the trial should be stopped or suspended, the Sponsor will take the necessary action to ensure that new recruitment to the trial is stopped whilst the TSC report is evaluated and the Research Ethics Committee is informed.

16.2.3 Data Monitoring and Ethics Committee (DMEC)

16.2.3.1 Membership DMEC (preliminary)
1. Professor Andy Stergachis, Professor of Pharmacy and Global Health, and pharmaco-epidemiologist, University of Washington will chair the DMEC. He chairs the safety working group of the Malaria in Pregnancy Consortium since 2008 and chaired several DMECs related to similar malaria in pregnancy trials.
2. Professor Ib Christian Bygbjerg, from the Institute for Public Health, University of Copenhagen, Denmark
3. Dr Billy Ngasala, from Department of Parasitology and Medical Entomolgy, Muhimbili University of Health and Allied Sciences (MUHAS), Tanzania
4. A DMEC statistician will be identified.

16.2.3.2 Role DMEC
The DMEC consist of 3-4 members (including one or more clinicians and one statistician, all with experience in clinical trials).

The DMEC shall assess the data regularly (before the annual TSC meeting) to review the data and the interim analysis. The assessment could be via email or other electronic medium annually prior to the TSC meeting. In the first year of recruitment more frequent assessment (bi-annually) is recommended for this trial with one face to face meeting at least once during the trial.

The members should be the only personnel to see the results separated by treatment group during the trial. They are independent and look at the trial from an ethical point of view of the participant safety, future participants and society in general. It is their responsibility to prevent participants being exposed to any excess risks by recommending to the Trial Steering Committee (TSC) for the trial suspension or termination early if the safety or efficacy results are sufficiently convincing. The trial statistician is usually invited to attend part of the DMEC meeting to present the most current data from the trial. This will be blinded, unless the DMEC specifically requests for an unblinded analysis.

16.2.3.3 Responsibilities DMEC
- To determine how frequently interim analysis of trial data should be undertaken.
- To consider the blinded or unblinded interim data from the trial and relevant information from other sources.
- To consider any requests for unblinding and release of interim trial data and to recommend to the TSC on the importance of this.
- To report (following each DMEC meeting) to the TSC and to recommend whether the trial should continue, the protocol be modified or the trial be stopped.

A full confidence report should be submitted in writing to the TSC at the end of each DMEC meeting

16.3 Appendix III. Declaration of Helsinki

WORLD MEDICAL ASSOCIATION DECLARATION OF HELSINKI

Recommendations guiding physicians in biomedical research involving human subjects. Adopted by the 18th World Medical Assembly, Helsinki, Finland, June 1964. Amended by the 29th World Medical Assembly, Tokyo, Japan, October 1975, 35th World Medical Assembly, Venice, Italy, October 1983, 41st World Medical Assembly Hong Kong, September 1989 and the 48th General Assembly, Somerset West, Republic of South Africa, October 1996

INTRODUCTION

It is the mission of the physician to safeguard the health of the people. His or her knowledge and conscience are dedicated to the fulfillment of this mission.

The Declaration of Geneva of the World Medical Association binds the physician with the words, "The health of my patient will be my first consideration", and the International Code of Medical Ethics declares that, "A physician shall act only in the patient's interest when providing medical care which might have the effect of weakening the physical and mental condition of the patient."

The purpose of biomedical research involving human subjects must be to improve diagnostic, therapeutic and prophylactic procedures and the understanding of the aetiology and pathogenesis of disease.

In current medical practice most diagnostic, therapeutic or prophylactic procedures involve hazards. This applies especially to biomedical research.

Medical progress is based on research which ultimately must rest in part on experimentation involving human subjects.

In the field of biomedical research a fundamental distinction must be recognized between medical research in which the aim is essentially diagnostic or therapeutic for a patient, and medical research, the essential object of which is purely scientific and without implying direct diagnostic or therapeutic value to the person subjected to the research.

Special caution must be exercised in the conduct of research which may affect the environment, and the welfare of animals used for research must be respected.

Because it is essential that the results of laboratory experiments be applied to human beings to further scientific knowledge and to help suffering humanity, the World Medical Association has prepared the following recommendations as a guide to every physician in biomedical research involving human subjects. They should be kept under review in the future. It must be stressed that the standards as drafted are only a guide to physicians all over the world. Physicians are not relieved from criminal, civil and ethical responsibilities under the laws of their own countries.

Basic principles

1. Biomedical research involving human subjects must conform to generally accepted scientific principles and should be based on adequately performed laboratory and animal experimentation and on a thorough knowledge of the scientific literature.
2. The design and performance of each experimental procedure involving human subjects should be clearly formulated in an experimental protocol which should be transmitted for consideration, comment and guidance to a specially appointed committee independent of the

investigator and the sponsor provided that this independent committee is in conformity with the laws and regulations of the country in which the research experiment is performed.

3. Biomedical research involving human subjects should be conducted only by scientifically qualified persons and under the supervision of a clinically competent medical person. The responsibility for the human subject must always rest with a medically qualified person and never rest on the subject of the research, even though the subject has given his or her consent.
4. Biomedical research involving human subjects cannot legitimately be carried out unless the importance of the objective is in proportion to the inherent risk to the subject.
5. Every biomedical research project involving human subjects should be preceded with careful assessment of predictable risks in comparison with foreseeable benefits to the subject or to others. Concern for the interests of the subject must always prevail over the interests of science and society.
6. The right of the research subject to safeguard his or her integrity must always be respected. Every precaution should be taken to respect the privacy of the subject and to minimize the impact of the study on the subject's physical and mental integrity and on the personality of the subject.
7. Physicians should abstain from engaging in research projects involving human subjects unless they are satisfied that the hazards involved are believed to be predictable. Physicians should cease any investigation if the hazards are found to outweigh the potential benefits.
8. In publication of the results of his or her research, the physician is obliged to preserve the accuracy of the results. Reports of experimentation not in accordance with the principles laid down in this Declaration should not be accepted for publication.
9. In any research on human beings, each potential subject must be adequately informed of the aims, methods, anticipated benefits and potential hazards of the study and the discomfort it may entail. He or she should be informed that he or she is at liberty to abstain from participation in the study and that he or she is free to withdraw his or her consent to participation at any time. The physician should then obtain the subject's freely-given informed consent, preferably in writing.
10. When obtaining informed consent for the research project, the physician should be particularly cautious if the subject is in a dependent relationship to him or her or may consent under duress. In that case the informed consent should be obtained by a physician who is not engaged in the investigation and who is completely independent of this official relationship.
11. In case of legal incompetence, informed consent should be obtained from the legal guardian in accordance with national legislation. Where physical or mental incapacity makes it impossible to obtain informed consent, or when the subject is a minor, permission from the responsible relative replaces that of the subject in accordance with national legislation. Whenever the minor child is in fact able to give a consent, the minor's consent must be obtained in addition to the consent of the minor's legal guardian.
12. The research protocol should always contain a statement of the ethical considerations involved and should indicate that the principles enunciated in the present Declaration are complied with.

Medical research combined with professional care (Clinical Research)

1. In the treatment of the sick person, the physician must be free to use a new diagnostic and therapeutic measure, if in his or her judgement it offers hope of saving life, re-establishing health or alleviating suffering.
2. The potential benefits, hazards and discomfort of a new method should be weighed against the advantages of the best current diagnostic and therapeutic methods.

3. In any medical study, every patient - including those of a control group, if any - should be assured of the best proven diagnostic and therapeutic method. This does not exclude the use of inert placebo in studies where no proven diagnostic or therapeutic method exists.
4. The refusal of the patient to participate in a study must never interfere with the physician-patient relationship.
5. If the physician considers it essential not to obtain informed consent, the specific reasons for this proposal should be stated in the experimental protocol for transmission to the independent committee (I,2).
6. The physician can combine medical research with professional care, the objective being the acquisition of new medical knowledge, only to the extent that medical research is justified by its potential diagnostic or therapeutic value for the patient.

Non-therapeutic biomedical research involving human subjects (Non-clinical biomedical research)

1. In the purely scientific application of medical research carried out on a human being, it is the duty of the physician to remain the protector of the life and health of that person on whom biomedical research is being carried out.
2. The subjects should be volunteers -- either healthy persons or patients for whom the experimental design is not related to the patient's illness.
3. The investigator or the investigating team should discontinue the research if in his/her or their judgement it may, if continued, be harmful to the individual.
4. In research on man, the interest of science and society should never take precedence over considerations related to the well-being of the subject.

16.4 Appendix IV. Budget and budget justification

16.4.1 Budget

Generic site budget estimates and summaries

Item Description	Total Cost Euro	Total Cost USD*
**PERSONNEL**		
Actual costs	€ 728,900	$ 819,400
Unit costs	€ 153,100	$ 172,100
Subtotal cost Personnel	€ 882,000.00	$ 991,500
**GOODS AND SERVICES**		
Certificates on Financial Statements	€ 10,000.00	$ 11,200
Equipment	€ 6,500	$ 7,300
Consumables and supplies	€ 72,600	$ 81,600
Training	€ 3,500	$ 4,000
Subtotal cost Goods and Services	€ 92,600	$ 104,100
**PATIENT ADMINISTRATION**		
Patient costs	€ 190,300	$ 213,900
Subtotal costs Patient Administration	€ 190,300	$ 213,900
**TRAVEL**		
In-country work-related travel	€ 65,000.00	$ 73,000
Subtotal Travel	€ 65,000.00	$ 73,000
**TOTAL DIRECT COST**		
Total Direct Costs	€ 1,229,900	$ 1,382,500
Indirect Costs (25%)	€ 307,400	$ 345,600
**TOTAL COSTS**	€ 1,537,300	$ 1,728,100

*The original budget is in Euros and subject to exchange rate fluctuations with the US Dollar and local currency
* The above are generic budget estimates across countries and specific costs will be subject to minimal variation between countries

16.4.2 Budget Justification

Funding has been approved for 3 years of field work starting in 2017 and an additional year to support completion of capacity development training. The overall budget includes central sponsorship support, trial insurance and trial monitoring, a central safety register, data management support and for trial coordination, international travel and academic capacity training. The budget also includes 25% overheads as indirect costs.

16.5 Appendix V. SPIRIT 2013 checklist clinical trial protocol

SPIRIT 2013 Checklist: Recommended items to address in a clinical trial protocol and related documents*

Section/item	Item No	Description	Addressed on page number
Administrative information			
Title	1	Descriptive title identifying the study design, population, interventions, and, if applicable, trial acronym	1
Trial registration	2a	Trial identifier and registry name. If not yet registered, name of intended registry	1, 8
	2b	All items from the World Health Organization Trial Registration Data Set	8
Protocol version	3	Date and version identifier	1
Funding	4	Sources and types of financial, material, and other support	1, 11, 28,61, 68
Roles and responsibilities	5a	Names, affiliations, and roles of protocol contributors	1 and 69
	5b	Name and contact information for the trial sponsor	1, 61
	5c	Role of study sponsor and funders, if any, in study design; collection, management, analysis, and interpretation of data; writing of the report; and the decision to submit the report for publication, including whether they will have ultimate authority over any of these activities	68
	5d	Composition, roles, and responsibilities of the coordinating centre, steering committee, endpoint adjudication committee, data management team, and other individuals or groups overseeing the trial, if applicable (see Item 21a for data monitoring committee)	71 to 73
Introduction			

Background and rationale	6a	Description of research question and justification for undertaking the trial, including summary of relevant studies (published and unpublished) examining benefits and harms for each intervention	18 to 21
	6b	Explanation for choice of comparators	22
Objectives	7	Specific objectives or hypotheses	22 to 22
Trial design	8	Description of trial design including type of trial (eg, parallel group, crossover, factorial, single group), allocation ratio, and framework (eg, superiority, equivalence, noninferiority, exploratory)	23
Methods: Participants, interventions, and outcomes			
Study setting	9	Description of study settings (eg, community clinic, academic hospital) and list of countries where data will be collected. Reference to where list of study sites can be obtained	25
Eligibility criteria	10	Inclusion and exclusion criteria for participants. If applicable, eligibility criteria for study centres and individuals who will perform the interventions (eg, surgeons, psychotherapists)	26
Interventions	11a	Interventions for each group with sufficient detail to allow replication, including how and when they will be administered	17, 27
	11b	Criteria for discontinuing or modifying allocated interventions for a given trial participant (eg, drug dose change in response to harms, participant request, or improving/worsening disease)	29
	11c	Strategies to improve adherence to intervention protocols, and any procedures for monitoring adherence (eg, drug tablet return, laboratory tests)	32
	11d	Relevant concomitant care and interventions that are permitted or prohibited during the trial	28,30
Outcomes	12	Primary, secondary, and other outcomes, including the specific measurement variable (eg, systolic blood pressure), analysis metric (eg, change from baseline, final value, time to event), method of aggregation (eg, median, proportion), and time point for each outcome. Explanation of the clinical relevance of chosen efficacy and harm outcomes is strongly recommended	32, 32
Participant timeline	13	Time schedule of enrolment, interventions (including any run-ins and washouts), assessments, and visits for participants. A schematic diagram is highly recommended (see Figure)	33 to 37

Sample size	14	Estimated number of participants needed to achieve study objectives and how it was determined, including clinical and statistical assumptions supporting any sample size calculations	37 to 38
Recruitment	15	Strategies for achieving adequate participant enrolment to reach target sample size	39
Methods: Assignment of interventions (for controlled trials)			
Allocation:			
Sequence generation	16a	Method of generating the allocation sequence (eg, computer-generated random numbers), and list of any factors for stratification. To reduce predictability of a random sequence, details of any planned restriction (eg, blocking) should be provided in a separate document that is unavailable to those who enrol participants or assign interventions	11, 23
Allocation concealment mechanism	16b	Mechanism of implementing the allocation sequence (eg, central telephone; sequentially numbered, opaque, sealed envelopes), describing any steps to conceal the sequence until interventions are assigned	23
Implementation	16c	Who will generate the allocation sequence, who will enrol participants, and who will assign participants to interventions	23
Blinding (masking)	17a	Who will be blinded after assignment to interventions (eg, trial participants, care providers, outcome assessors, data analysts), and how	11, 23, 28, 28,40, 50,
	17b	If blinded, circumstances under which unblinding is permissible, and procedure for revealing a participant's allocated intervention during the trial	40
Methods: Data collection, management, and analysis			
Data collection methods	18a	Plans for assessment and collection of outcome, baseline, and other trial data, including any related processes to promote data quality (eg, duplicate measurements, training of assessors) and a description of study instruments (eg, questionnaires, laboratory tests) along with their reliability and validity, if known. Reference to where data collection forms can be found, if not in the protocol	40
	18b	Plans to promote participant retention and complete follow-up, including list of any outcome data to be collected for participants who discontinue or deviate from intervention protocols	32, 39

Data management	19	Plans for data entry, coding, security, and storage, including any related processes to promote data quality (eg, double data entry; range checks for data values). Reference to where details of data management procedures can be found, if not in the protocol	40 to 43
Statistical methods	20a	Statistical methods for analysing primary and secondary outcomes. Reference to where other details of the statistical analysis plan can be found, if not in the protocol	43 to 44
	20b	Methods for any additional analyses (eg, subgroup and adjusted analyses)	44
	20c	Definition of analysis population relating to protocol non-adherence (eg, as randomised analysis), and any statistical methods to handle missing data (eg, multiple imputation)	43 to 44
Methods: Monitoring			
Data monitoring	21a	Composition of data monitoring committee (DMC); summary of its role and reporting structure; statement of whether it is independent from the sponsor and competing interests; and reference to where further details about its charter can be found, if not in the protocol. Alternatively, an explanation of why a DMC is not needed	73
	21b	Description of any interim analyses and stopping guidelines, including who will have access to these interim results and make the final decision to terminate the trial	45
Harms	22	Plans for collecting, assessing, reporting, and managing solicited and spontaneously reported adverse events and other unintended effects of trial interventions or trial conduct	47 to 48
Auditing	23	Frequency and procedures for auditing trial conduct, if any, and whether the process will be independent from investigators and the sponsor	50
Ethics and dissemination			
Research ethics approval	24	Plans for seeking research ethics committee/institutional review board (REC/IRB) approval	51
Protocol amendments	25	Plans for communicating important protocol modifications (eg, changes to eligibility criteria, outcomes, analyses) to relevant parties (eg, investigators, REC/IRBs, trial participants, trial registries, journals, regulators)	51

Consent or assent	26a	Who will obtain informed consent or assent from potential trial participants or authorised surrogates, and how (see Item 32)	52
	26b	Additional consent provisions for collection and use of participant data and biological specimens in ancillary studies, if applicable	32, 83
Confidentiality	27	How personal information about potential and enrolled participants will be collected, shared, and maintained in order to protect confidentiality before, during, and after the trial	52
Declaration of interests	28	Financial and other competing interests for principal investigators for the overall trial and each study site	53
Access to data	29	Statement of who will have access to the final trial dataset, and disclosure of contractual agreements that limit such access for investigators	53
Ancillary and post-trial care	30	Provisions, if any, for ancillary and post-trial care, and for compensation to those who suffer harm from trial participation	55
Dissemination policy	31a	Plans for investigators and sponsor to communicate trial results to participants, healthcare professionals, the public, and other relevant groups (eg, via publication, reporting in results databases, or other data sharing arrangements), including any publication restrictions	56
	31b	Authorship eligibility guidelines and any intended use of professional writers	59
	31c	Plans, if any, for granting public access to the full protocol, participant-level dataset, and statistical code	59
Appendices			
Informed consent materials	32	Model consent form and other related documentation given to participants and authorised surrogates	83 onwards
Biological specimens	33	Plans for collection, laboratory evaluation, and storage of biological specimens for genetic or molecular analysis in the current trial and for future use in ancillary studies, if applicable	32 to 36

16.6 Appendix VI. Participant information sheets and informed consent statements

16.6.1 Participant Information Sheet: Main trial (all women) (English)
See separate document for participant information sheets and informed consent statements

16.6.2 Consent statement for screening and participation in the trial (all women) (English)
See separate document for participant information sheets and informed consent statements

16.6.3 Consent Statement for long term storage of samples and use for future research (all women) (English)
See separate document for participant information sheets and informed consent statements

16.6.4 Consent Statement for vaginal swab and stool samples (sub-group) (English)
See separate document for participant information sheets and informed consent statements

16.6.5 Consent Statement for nasopharyngeal swab sample (sub-group) (English)
See separate document for participant information sheets and informed consent statements

16.6.6 Participant Information Sheet for cardiac safety monitoring (subgroup) (English)
See separate document for participant information sheets and informed consent statements

16.6.7 Consent Statement for cardiac safety monitoring (sub-group) (English)
See separate document for participant information sheets and informed consent statements

16.6.8 Participant Information Sheet for assessment of nutritional endpoints (sub-group) (English)
See separate document for participant information sheets and informed consent statements

16.6.9 Consent Statement for assessment of nutritional endpoints (sub-group) (English)
See separate document for participant information sheets and informed consent statements

16.7 Appendix VII. Description of other clinical and laboratory methods

5. This section 'Appendix VII. Description of other clinical and laboratory methods' of the appendix, describes the clinical and laboratory based assays for some secondary endpoints conducted in subgroups of women in specific study sites or otherwise in a random sample of women in all sites. These volume and timing of these samples is described in 'Table 1: Summary Table of Study Design and Schedule of Assessment' and in sections 4, page 15, and in section 4 'Participants timeline

', page 33 onwards. The purpose if this appendix text is to describe the assays and measures of the procedures in more detail.

16.8.1 Impact on maternal and neonatal anthropometric and biochemical markers of nutritional status

COLLABORATING INSTITUTIONS AND CONTACT PERSONS: Prof Ken Maleta (COM, University of Malawi); Dr Ulla Ashorn (University of Tampere, Finland); Dr Holger Unger and Prof Stephen Rogerson (University of Melbourne, Australia)

OBJECTIVE: To evaluate the impact of adding AZ to IPTp-DP on maternal and neonatal anthropometric and biochemical markers of nutritional status and maternal intestinal inflammation.

RATIONALE: Previous trials showed that AZ results in general effects on maternal health including gains in gestational weight, maternal body mass index (BMI) and mid-upper arm circumference (MUAC).[81] If this is confirmed in the current trial, it will be important to examine whether this is through improved intestinal nutrient absorption, for example through reduction in gut inflammation or effects on gut microbe populations, or whether this simply reflects the reduced rate of infections which reduces maternal energy expenditure. Neonatal antibiotic use has been associated with the development of obesity in later life, possibly through effects on the neonatal intestinal microbiome.[82] Less is known about the impact of gestational antibiotic use on maternal and neonatal nutritional status.[83]

Improved maternal and neonatal nutritional status as a result of gestational antibiotic exposure could be beneficial, given maternal undernutrition and low birthweight remain enormous health problems, in particular in developing countries.[84,85] Furthermore, nutritional depletion as a result of pregnancy (and e.g. pregnancy-associated infections) remains an issue in low-resource settings, and could potentially be averted through secondary effects of gestational antimalarial/antibiotic use.[86] More epidemiological and mechanistic evidence for a role of gestational antibiotic use in shaping short- and long-term maternal and neonatal nutritional status is urgently required in order to rule out adverse effects such as later-life obesity (both mother and neonate) and to firmly establish benefits such as improvements in maternal nutritional status that will benefit the fetus and neonate. We will also measure aflatoxin levels as potential confounder because recent evidence from both animal and human studies has shown a significant association between aflatoxin and impaired growth.[87]

METHODS: *Nutritional assessment methods (anthropometric, other):* Detailed assessments of maternal and neonatal body composition will be done in a subset of women. The following routine anthropometric measurements will be taken from the mother: weight, height, MUAC, skinfold thickness (SFT) with standard Harpenden calipers,[88] hip and waist circumference, and from the newborn: weight, head circumference, abdominal circumference, length, SFT). SFT will be measured used (Holtain, UK). In addition, mothers' postpartum body composition, particularly % body fat, will

be evaluated using the non-invasive technique of segmental multifrequency bioelectrical impedance analysis (BIA) using Tanita body composition monitors (Tanita, Japan).[89] Newborn/infant % body fat will be assessed using the non-invasive technique of Near Infrared Interactance device to determine body composition. Postpartum maternal and neonatal nutritional composition will further be evaluated using a stable isotope techniques have been used in studies of human nutrition for over 50 years. Deuterium is a stable (non-radioactive) isotope of hydrogen. It is given orally as deuterium oxide (2^H2O or 'labeled water'), and after mixing with body water, it is eliminated from the body in urine, saliva, sweat and human milk. Deuterium oxide is handled in the body in the same way as water, and is dispersed through the body water within a matter of hours. Through measurement of 2^H2O excretion (saliva samples) it is possible to determine body tissue composition. It is estimated that approximately 1,400 women (700 IPTp-DP and 700 IPTp-DPAZ) will provide >90% to detect differences of 60g/week in gestational, weight gain, 0.4 kg m2 in BMI, and 0.3 cm in post-partum MUAC.

Laboratory assays: We measure levels of maternal faecal calprotectin, a marker of enteric inflammation, in the third trimester, by freezing a small stool sample from the mother (-20 °C). Samples will be analysed within approximately 1 year of collection by suspending 0.1 g of thawed faeces in 5 mL extraction buffer and testing the supernatant using a standard enzyme-linked immunosorbent assay (ELISA) technique (CALPRO calprotectin ELISA Test, Calpro AS, Norway). We will also assess blood adipokines, specifically leptin and adiponectin (secretion correlates with fatty tissue mass) in the mother (from the blood sample collected at the scheduled third trimester visit) and newborn (cordblood). Maternal and cord EDTA plasma samples for leptin and adiponectin will be stored at -80°C prior to testing and measured by ELISA (R&D Systems USA). It is estimated that approximately 800 women (400 IPTp-DP, 400 IPTp-DPAZ) will provide >90% to detect differences of 100 µg/g and 2.28 ng/mL in calprotectin and leptin. Plasma aflatoxin-albumin adduct levels (AF-alb), will be measured using competitive binding ELISA,[90] which is a sensitive and specific method for quantification of biologically effective aflatoxin in plasma reflecting exposure during the past 2-3 months.

16.8.2 Malaria detection and molecular markers of antimalarial resistance

COLLABORATING INSTITUTIONS AND CONTACT PERSONS: Ass. Prof Dr Michael Alifrangis, University of Copenhagen

OBJECTIVE: 1. To evaluate the contribution of recrudescence and reinfection to prevalent and incidence of malaria infection over the course of pregnancy; 2. To investigate the prevalence of molecular antimalarial resistance markers in parasite isolates

RATIONALE: The use of DP, a slowly eliminated antimalarial, for IPTp may pose as a source of drug pressure that may drive the emergence of piperaquine resistance in sub-Saharan Africa. To evaluate this, we will conduct molecular studies to detect malaria infections from enrolment to delivery, differentiate these as new or recrudescent infections and determine their molecular resistance status to artemisinins and piperaquine.

METHODS

Diagnostic qPCR: Dry blood spots or redcell pellets will be prepared at enrolment, all unscheduled (sick) visits and at delivery from all women and from a random sub-sample of approximately 1,464 asymptomatic women at each scheduled monthly follow-up visit. DNA will be extracted using chelex-100 extraction[91] or Qiagen DNA extraction for further qPCR targeting the high-copy telomere-associated repetitive element 2 (limit of detection 0.15 parasites/µl blood).[92]

Genotyping distinguish recrudescent and re-infections: qPCR positive samples from women at two sequential monthly visits will be used for PCR genotyping of the polymorphic *P.falciparum* genes MSP2, GLURP and MSP1; interpretation of results will be performed using standardised methodologies.[93]

Molecular markers of antimalarial drug resistance: All *P. falciparum*-qPCR positive samples collected will be used for the evaluation of artemisinin, piperaquine, and SP resistance molecular markers. All PCR positive samples at enrolment will be examined for SNPs in the genes *Pfdhfr*, *Pfdhps (SP arm)*, *K13* and *Pfmdr*-1 and plasmepsin 2,3 (DP arms). A high-throughput next-generation platform for targeted sequencing using Illumina®-basedtechnology[94]will be applied as the main method. After index PCR, the gene fragments will be sequenced using the Illumina Miseq® platform available through collaboration with researchers at the Danish Technical University, Denmark. In addition, direct Sanger sequencing methods will be applied to a subset of samples (approximately 200) targeting mainly K13 to confirm novel findings and as a gold standard comparison using published PCR methods [6] and after PCR, also send for commercial sequencing. A PCR sequence-specific oligonucleotide probe (SSOP)-ELISA[95] will be deployed as a reference method to the Illumina methodology regarding Pfdhfr and Pfdhps data.

Data analysis: The degree of SP resistance in the parasite population will be expressed as the frequency of the *Pfdhps*-A437G, K540E, or A581G mutations, which predict the frequency of quadruple, quintuple, and sextuple *Pfdhfr–Pfdhps* mutant haplotypes, respectively. In addition, we will assess prevalence of molecular markers associated with tolerance/resistance to artemisinin and piperaquine (*K13* and *Pfmdr1,* plasmepsin-2). These molecular data will be used as potential effect modifiers in the impact analysis of the main trial.

16.8.3 Antimicrobial activity and azithromycin resistance

COLLABORATING INSTITUTIONS AND CONTACT PERSONS: Ass Prof Matt Chico, London School of Hygiene and Tropical Medicine, UK, Prof Nigel Klein, University College London, UK, Dr Ulla Ashorn, University of Tampere, Finland, Dr Julie Gutman, CDC, USA.

OBJECTIVE: 1. To determine the efficacy of IPTp with DP+AZ on curable STIs/RTIs) relative to IPTp with SP and DP alone. 2. To determine the effect of SP and AZ on the intestinal and vaginal microbiomes of mothers, environmental enteric dysfunction, and the intestinal microbiomes of neonates relative to DP alone. 3. To determine the effect of multiple doses of DP-AZ on the prevalence of macrolide resistance in medically important pathogens

RATIONALE: Assessment of these endpoints involve the analysis of biological samples to provide key evidence of antimicrobial effect conferred by study drug on medically important pathogens and changes in the microbiota and gut inflammation that result from study drug exposure among pregnant women. Objective-1 will measure the effect of study treatment against curable STIs/RTIs. Objective-2 will investigate the effect of study treatment on the vaginal microbiome using MiSeq sequencing analysis from vaginal swab samples collected at enrolment (pre-dose) and, again, at week 32-35 approximately (or otherwise at the nearest scheduled monthly visit). The same relationship and gut inflammation will be assessed between the intestinal microbiome of pregnant women with stool samples collected at enrolment (pre-dose) and, again, at week 32-35 approximately (or otherwise at the nearest scheduled monthly visit), and in their new-borns at 6–8 weeks postpartum. In all instances, analysis of similarities (ANOSIM) will be used to estimate distances (degree of similarity) between samples. The phylogeny-based UniFrac metric where distance is calculated based on the degree to which any two communities share branch length on a

bacterial tree of life.[96] Beta-diversity metrics will be used to estimate statistical significance. Objective-3 will investigate whether multiple doses of AZ changes the prevalence of colonization and carriage of macrolide resistance in colonies of *Pneumococcus* resident in the nasopharynx.

STIs/RTIs assessment methods: Syphilis testing with rapid plasma reagin (RPR) that will be provided per national norms. RPR positive women at enrolment (3%; n=140)[97] will receive treatment (2.4 MU benzathine penicillin G) and a confirmatory TPHA. If rapid point of care testing for syphilis is the national norm, confirmatory TPHA will still be conducted. Other STIs/RTIs will also be managed per national norms. Retrospective batch analyses will be conducted on samples from in all three countries (n~1,464) . Vaginal samples will be collected at enrolment and between ~32-35 weeks approximately (or otherwise at the nearest scheduled monthly visit). Gonorrhoea and chlamydia will be detected by NAAT tests[98]. Bacterial vaginosis will be diagnosed by Nugent scoring[99] alongside In Pouch culture of cervical samples.[100] Trichomonas testing will involve In Pouch culture of cervical samples collected on day 28 post-partum. Using the composite endpoint – any STI/RTI detected between approximately 32-35 weeks and 4 weeks post-partum – a sample of 432 per arm (1,296 total) provides 80% power to detect a 25% reduction from 40% in the DP-alone arm to 30% in the SP or in the DP-AZ arm, at a significance level of 2.5% to allow for 2 comparisons, and to detect a further 27.8% reduction from 30% in the SP-arm to 21% in DP-AZ arm (P = 0.05; 1 comparison). To allow for loss-to-follow-up, a sample size of 1,464 is included.

Microbiome: We will assess the impact on the intestinal and vaginal microbiome using Illumina MiSeq sequencing with V3 cassettes – including coverage of V1, V2, V5 and V6 regions of the 16S rDNA gene[101] – with stool and vaginal-swab samples collected among women from selected sites at enrolment and, again, in the third trimester; stool samples from the newborns of these same women will be collected at 6 to 8 weeks post-partum. A random selection of 20 samples will have 87% power to detect a 10% difference in alpha-diversity (5 vs 4.5) (SD 0.5, α=0.05). Thus, 300 total samples collected across all three treatment groups at each of the five time points will be randomly selected and run with mixed mock community controls to permit improved genus quantification across MiSeq runs, and for diversity and precision-sensitivity analyses.[102,103] Additional panels of Real-Time PCRs will be run. Sequences from microbiota samples will be binned in operational taxonomic units sharing ≥97% nucleotide sequence identity, and assigned a taxonomy based on the naïve Bayesian RDP classifier. Analyses will then determine whether exposure to study drug during pregnancy alters the composition of the microbiota or the relationship between maternal and infant microbiota structure. Degrees of similarity between pairs of maternal-infant faecal microbiota samples will be computed using the phylogeny-based UniFrac metric.[96] In addition to whole community similarity between mother and infant, computational methods will be applied to identify bacterial species shared between mothers and infants, and the degree to which this varies by treatment group.[104] Microbiota analyses will consist of a semi-quantitative indication of the relative amounts of bacterial species. Pooled DNA will be used to screen for antimicrobial resistance with a multiplexed real-time PCR to detect *msr(A)*, *mef(A)*, 23S rRNA variation by identifying *erm* genes and mutations in the *mtrR* promoter regions and *ere(A)* and *ere(B)* genes. Markers of gut inflammation will be measured in maternal stool and sera (e.g. calprotectin).

Macrolide resistance: This component will assess the effects of repeated AZ dosing on macrolide resistance. Naso-pharyngeal samples will be collected at enrolment, near delivery (collected at the last scheduled visit around 36-40 weeks gestational age or at delivery, or 6 to 8 weeks post-delivery and at 6 month or later. The prevalence of pneumococcal carriage in the general population in western Kenya is 27-43%.[105] Using a conservative estimate that 25% of women are colonized, and allowing for 13.7% lost to follow-up, 344 women per arm, or 1,032 women in total will have 80%

power to detect a 10%- point difference in the incidence of resistance between baseline and follow-up, assuming that 5% of women have macrolide resistant Pneumococcus at baseline, and no increase is seen in the non-azithromycin arms. If only 10% or 15% of women are colonized with pneumococcus, this sample size will be sufficient to detect a 22.3% and 16.0% difference. Nasopharyngeal samples will be collected on FLOQSwabs™ (Copan) and stored at -80°C in STGG media until processing. Culture methods will be used to identify *Pneumococcus*; e-test or broth microdilution will be used to assess changes in resistance patterns associated with SP and AZ.

16.8.4 *Maternal antibody, trans-placental antibody transfer and multi-pathogen neonatal cell mediated immune responses*

COLLABORATING INSTITUTIONS AND CONTACT PERSONS: Ass Prof Ann Moormann, University of Massachusetts, Worcester, USA and Prof Stephen Rogerson, University of Melbourne, Australia.

OBJECTIVE: To determine if antimalarial treatment of women during pregnancy is associated with changed maternal immunity, robust transfer of maternal antibodies and the development of cell-mediate immunity in infants to other pathogens, in addition to malaria.

RATIONALE: Maternal antibodies may protect pregnant women from malaria, but placental malaria has been associated with impaired transfer of maternal antibodies and hypergammaglobulinemia.[106,107] This negatively impacts the new-born's ability to be protected against malaria as well as viral infections.[106,108] The scope of this lack of maternal antibody protection tends to be studied one or two infections at a time. Using multiplex methods,[109,110] we will be able to simultaneously measure a panel of antibody titres and cell-mediated immune responses to multiple pathogens within the same study participants using extremely low volumes of peripheral blood.

METHODS

a) Serology profiling: Using a Luminex, multiplex suspension bead-based assay to simultaneously measure antibody titers to a panel of individual antigens or immunologic peptides against malaria (ie MSP1, AMA1, CSP, LSA1, etc), EBV, CMV, measles virus, tetanus toxoid, arboviruses etc.[109] We will use a Systems Serology approach to identify protective antibodies.[110]

b) Cell-mediated immunity: Using multi-parameter flow cytometry and potentially single cell RNAseq[111] we will efficiently and comprehensively interrogate immunity specific to pathogens and EPI vaccine-induced responses.

Sample size: ~1,500, all scheduled clinic visits, delivery and 1, and 6-8 weeks infant visit (heel prick)

16.8.5 *IPTp and maternal immune modulation*

COLLABORATING INSTITUTIONS AND CONTACT PERSONS: Prof Stephen Rogerson, University of Melbourne

OBJECTIVE: To examine effects of IPTp with DP, DPAZ and SP on maternal immune activation and pathogen clearance.

RATIONALE: The beneficial effect of AZ extends beyond malaria control. To explain our surprising and important results, we will test new hypotheses regarding effects of AZ and SP on immunomodulation. We postulate that AZ will decrease cytokine elicitation, especially of macrophage derived inflammatory cytokines, from PBMCs.

METHODS: *a) Clinical sampling.* 100 study participants, one third from each arm, matched for gravidity. Peripheral blood (as part of the standard study sample described in the main text) will be

collected at enrolment, at second treatment visit and at the last scheduled visit (around) 36 weeks' gestation for PBMCs, whole blood and plasma CRP. Stool, PBMC, plasma and RBC will be frozen.

b) Modulating maternal innate immune responses to malaria and other infections. PBMC cytokine responses to infected erythrocytes (IE) and to LPS will be compared between the three arms. IE (CS2 line, associated with placental malaria) will be added at a 3:1 ratio to PBMCs, and LPS at 100 ng/ml. PHA is used as viability control.

c) Altered phagocytic clearance. To determine whether AZ and/or SP augments the clearance of IE and bacteria, we will use our whole blood assay.[112] Fresh whole blood will be analysed by flow cytometer and 1.5 x 10^5 phagocytes will be suspended in 100 µl with ethidium bromidelabeled IE (25:1) for 30 mins, or with 2 x 10^8*E. coli* pHRODO for 10 mins at 37°C, or 4°C (control). After counterstaining with FITC-CD14 and CD16-APC, proportion of monocytes taking up IE or *E coli* is determined. Monocyte subsets (classical, intermediate and non-classical) will be determined. We hypothesise that azithromycin, through its effect on phagocytic function, alters the uptake of parasites and/or bacteria, potentially affecting clearance of these infections.

Data analysis and sample sizes: Paired samples will be collected at enrolment, the next monthly and the last monthly schedule visit during pregnancy. We will perform paired (within subject) and between-subject (SP, DHA-PQ or DHA-PQ + AZ) unmatched comparisons. Based on previous observations the study has approximately 80% power to detect a 50% reduction in cytokine output between groups.

16.8.6 *Biomarkers of placental function and adverse birth outcomes*

COLLABORATING INSTITUTIONS AND CONTACT PERSONS: Prof Kevin Kaine, University of Toronto, Canada

OBJECTIVE: To evaluate biomarkers and pathways regulating placental development and function and that are predictive of adverse birth outcomes (e.g. fetal growth restriction (FGR), preterm birth (PTB)) in a prospective study of pregnant women at high risk of malaria.

RATIONALE: Normal placental development is essential to support the growth of the fetus throughout pregnancy. Vasculogenic processes in the first trimester regulate de novo formation and growth of blood vessels.[113] Angiogenic processes beginning in the second trimester induce remodeling of the underlying architecture of the placenta to allow for increased blood flow and surface area for nutrient exchange required for exponential fetal growth in the 3rd trimester.[113,114] Healthy birth outcomes require tight regulation of these vasculogenic, angiogenic, and inflammatory pathways and disruptions to these critical axes that can result in placental dysfunction and poor birth outcomes. For example, placental vascular dysfunction is associated with multiple pregnancy pathologies including gestational diabetes, preeclampsia, and malaria in pregnancy.[115-117] The early identification of pregnancies at risk of adverse birth outcomes would enable risk stratification for increased monitoring and interventions to improve outcome. We propose to externally validate a panel of biomarkers to examine these markers in the context of risk factors contributing to FGR and PTB, including malaria infection in pregnancy, and how these markers would be modified with antimalarial treatment regimen for IPTp.

METHODS Venous blood samples (minimum required volume of 250 µL) collected from participants at study enrolment, scheduled antenatal visits and delivery will be processed via a custom multiplex Luminex®-based assay to quantify plasma levels of markers of placental function, inflammation and development including Angiopoietin-1 (Ang-1), Angiopoietin-2 (Ang-2), Angiopoietin-Like 3 (AngptL3), Vascular Endothelial Growth Factor (VEGF), Soluble fms-like tyrosine kinase 1 (sFlt-1),

soluble Tumor Necrosis Factor Receptor 2 (sTNFR2), Placental Growth Factor (PGF), Interleukin-18 Binding Protein (IL-18BP), soluble Intercellular Adhesion Molecule-1 (sICAM-1), soluble Endoglin (sEndoglin), C-reactive protein (CRP), Chitinase-3-Like Protein-1 (CHI3L1), complement components including C5a (C5a), and brain derived neurotrophic factor (BDNF), and inflammatory biomarkers such as C-reactive protein (CRP). Proteins were selected based on evidence of a critical role of each protein in inflammatory pathways and placental vasculogenesis, as well as associations with birth and fetal outcomes.

Sample size: A sample size of 1,500 women will provide >80% power to detect clinically and statistically significant differences in at least four biomarkers between women who deliver preterm vs term birth (>=37 weeks). These power calculations were based on detectable differences observed in a previous study conducted in Tanzania, and based on a t-test and boosted by 15% to account for non-normal distribution of biomarkers (alpha=0.05).

16.8.7 Performance of a highly sensitive diagnostic test for malaria

COLLABORATING INSTITUTIONS AND CONTACT PERSONS: Christine Bachman, from Intellectual Ventures Limited, Washington, USA; and Dr Gonzalo Domingo, from PATH, Seattle, Washington, USA, and Dr Iveth Gonzalez from FIND, Geneva, Switzerland, Dr Emily Adams, LSTM, UK

OBJECTIVE: To evaluate the diagnostic performance of novel diagnostic devices and highly sensitive diagnostic test for malaria in pregnant women.

RATIONALE: Current RDT are inadequate for the diagnosis of placental malaria in asymptomatic pregnant women. There is a new generation of diagnostic tests currently under development that have a lower limit of detection, including, but not limited to, use of a photo-thermal reader using a standard RDT and an experimental, highly-sensitive RDT (HSRDT). A reformatted RDT will also be trialled to enhance visual read and provide further enhancement with the reader. They have not yet been evaluated in asymptomatic pregnant women. We will determine the diagnostic performance of new diagnostic tests in sample taken from the pregnant women included in the trial and compare them against the PCR results obtained in the trial as reference standard. Since these new diagnostics tests are still experimental and under development and can be used for research only, but not yet for clinical care.

METHODS: The reformatted RDT and HSRDT will be run on site on a 5 to 100 microlitre taken from the blood samples collected from participants. The RDTs will be read visually and also by placing the cartridge into the reader. The reader will take approximately 1-3 minutes to complete the analysis. The results from the RDT Reader will be blinded to the health care worker and because these involve proto-types they cannot be used for patient care.

In addition, a 75 microlitre aliquot of blood will be stored frozen in cryovials for a later multiplex quantitative ELISA (Q-ELISA) and standard RDTs (not for patient care) at PATH (Seattle, USA). The Q-ELISA assay, contains spots for HRP2, and pan-malaria LDH. The capture and detection antibodies are orthologous to those on the RDTs and therefore allow confirmation of antigenemia. All experimental assays shall be conducted for research purposes only within the clinical setting of the trial but will not inform point of care.

A total of 1,500 samples will be tested using the HSRDT and Q-Elisa providing 0.06 confidence interval at 0.95 confidence level for a 90% expected sensitivity, and a 1,500 sample for the comparison against the developmental readers. The primary outcome will be the sensitivity of the various diagnostic methods compared to qPCR or standard RDTs, including the threshold of

detection of the experimental assays. Secondary outcomes will include other standard measures of diagnostic accuracy (specificity, positive predictive value, and negative predictive value) against PCR positivity.

16.9 APPENDIX VIII. PRODUCT CHARACTERISTICS

16.9.1 Dihydroartemisinin-piperaquine product insert (Sigma-Tau)

PACKAGE LEAFLET: INFORMATION FOR THE USER

Eurartesim 320 mg/40 mg film-coated tablets
Piperaquine tetraphosphate/dihydroartemisinin

Read all of this leaflet carefully before you start using this medicine
- Keep this leaflet. You may need to read it again.
- If you have any further questions, ask your doctor or pharmacist.
- This medicine has been prescribed for you. Do not pass it on to others. It may harm them, even if their symptoms are the same as yours.
- If any of the side effects gets serious, or if you notice any side effects not listed in this leaflet, please tell your doctor or pharmacist.

In this leaflet:

1. What Eurartesim is and what it is used for
2. Before you or your child takes Eurartesim
3. How to take Eurartesim
4. Possible side effects
5. How to store Eurartesim
6. Further information

1. WHAT EURARTESIM IS AND WHAT IT IS USED FOR

Eurartesim contains the ingredients piperaquine tetraphosphate and dihydroartemisinin. It is used to treat uncomplicated malaria when use of a medicine given by mouth is appropriate.

Malaria is caused by infection with a parasite called *Plasmodium*, spread by the bite of an infected mosquito.There are different types of *Plasmodium* parasite. Eurartesim kills the *Plasmodium falciparum* parasite.

The medicine can be taken by adults, children and infants over 6 months old who weigh 5 kilograms or more.

2. BEFORE YOU OR YOUR CHILD TAKE EURARTESIM

Do not take Eurartesim if you or your child:
- is allergic (hypersensitive) to the active substances, piperaquine tetraphosphate or dihydroartemisinin, or to any of the other ingredients of Eurartesim (see section 6 for a list of these);
- has a severe type of malaria infection which has affected parts of your body such as the brain, lungs or kidneys;
- has a heart condition, such as changes to the rhythm or rate of your heart beat, or heart disease;
- knows that any member of your family (parents, grandparents, brothers or sisters) died suddenly due to a heart problem or was born with heart problems;
- suffers from changes to the levels of salts in your body (electrolyte imbalances);
- is taking other medicines that can have an effect on heart rhythm, such as:
  - quinidine, disopyramide, procainamide, amiodarone, dofetilide, ibutilide, hydroquinidine or sotalol;
  - medicines used to treat depression;
  - medicines used to treat mental health problems such as phenothiazines, sertindole, sultopride, chlorpromazine, haloperidol, mesoridazine, pimozide, or thioridazine;

48

- medicines used to treat infections. These include some of the types of medicines used to treat bacterial infections (macrolides [such as erythromycin or clarithromycin] and fluoroquinolones [such as moxifloxacin and sparfloxacin]) or fungal infections (including fluconazole and imidazole) as well as pentamidine (used to treat a specific type of pneumonia) and saquinavir (for treatment of HIV);
- antihistamines used to treat allergies or inflammation such as terfenadine, astemizole or mizolastine;
- certain medicines used to treat stomach problems such as cisapride, domperidone or droperidol;
- other medicines such as vinca alkaloids and arsenic trioxide (used to treat certain cancers), bepridil (used to treat angina), diphemanil (used to treat stomach disturbances), levomethadyl and methadone (used to treat drug addiction), and probucol (used to treat high blood cholesterol levels).

• has recently (for example within about one month) been treated for malaria with certain medicines or has taken certain medicines to prevent malaria. These medicines include: mefloquine, halofantrine, lumefantrine, chloroquine or quinine

If any of the above applies to you or your child or if you are unsure, tell your doctor or pharmacist before taking or giving Eurartesim.

Take special care with Eurartesim
Check with your doctor or pharmacist before taking this medicine if you or your child:

- has liver or kidney problems;
- has a malaria infection caused by a parasite other than *Plasmodium falciparum*;
- is taking or has taken any other medicines for the treatment of malaria (other than those mentioned above);
- is pregnant or breastfeeding (see below);
- is female, elderly (over 65 years) or vomiting;
- is taking certain other medicines which could cause possible metabolic interactions. Examples are listed in the section "Taking other medicines".

If you are not sure about any of the above, please ask your doctor or pharmacist.

Use in children
Do not give this medicine to infants under 6 months or below 5 kg in weight.

Taking other medicines
Please tell your doctor or pharmacist if you or your child is taking or has recently taken any other medicines, including medicines obtained without a prescription. Some medicines can affect the way Eurartesim works and your doctor may decide that Eurartesim is not suitable or that extra checks are needed while you or your child is taking the medicinal products which could cause possible interactions. Examples are listed below (but there are several others):

- some medicines used to treat high cholesterol in the blood (such as atorvastatin, lovastatin, simvastatin);
- medicines used to treat hypertension and heart problems (such as diltiazem, nifedipine, nitrendipine, verapamil, felodipine, amlodipine);
- some medicines used to treat HIV (antiretroviral medicinal products): protease inhibitors (such as amprenavir, atazanavir, indinavir, nelfinavir, ritonavir), non-nucleoside reverse transcriptase inhibitors (such as efavirenz, nevirapine);
- some medicines used to treat microbial infections (such as telithromycin, rifampicin, dapsone);
- medicines used to help you fall asleep: benzodiazepines (such as midazolam, triazolam, diazepam, alprazolam), zaleplon, zolpidem;
- medicines used to prevent/treat epileptic seizures: barbiturates (such as phenobarbital), carbamazepine or phenytoin;
- medicines used after organ transplantation and in autoimmune diseases (such as cyclosporin, tacrolimus);

- sex hormones, including those contained in hormonal contraceptives (such as gestodene, progesterone, estradiol), testosterone;
- glucocorticoids (hydrocortisone, dexamethasone);
- omeprazole (used to treat diseases related to gastric acid production);
- paracetamol (used to treat pain and fever);
- theophylline (used to improve bronchial air flow);
- nefazodone (used to treat depression);
- aprepitant (used to treat nausea);
- some gases (such as enflurane, halothane and isoflurane) used to give a general anaesthetic.

Taking Eurartesim without food and drink
You should take Eurartesim tablets with water only.
You should take this medicine on an empty stomach. You should take each dose no less than 3 hours after the last food intake, and no food should be taken within 3 hours after each dose of Eurartesim. You can drink water at any time. You should not take Eurartesim with grapefruit juice due to possible interactions.

Pregnancy and breast-feeding
Tell your doctor if you are pregnant, think you may be pregnant or become pregnant, or if you are breast-feeding.

Eurartesim must not be used in pregnancy if your doctor can give you an alternative medicine. If you receive Eurartesim while pregnant, please note that a pregnancy registry is in place to monitor the pregnancy outcomes.

You should not breast-feed your baby while taking this medicine.

If you are taking folate supplements to prevent possible neural tube birth defects, you can continue taking them at the same time as Eurartesim.

Ask your doctor or pharmacist for advice before taking any medicine during pregnancy or breast-feeding.

Driving and using machines
You can drive or use machines after taking Eurartesim.

3. HOW TO TAKE EURARTESIM

Always take Eurartesim exactly as your doctor has told you to. You should check with your doctor or pharmacist if you are not sure.

Take this medicine with water and on an empty stomach. You or your child should take each dose at least 3 hours after your last meal. You should also avoid eating until 3 hours after taking Eurartesim. You can drink water at any time.
If the tablets are difficult to swallow, you can crush and mix them with water; drink the mixture immediately.

A course of Eurartesim lasts 3 consecutive days. Take one dose on each day. You should try to take the dose at about the same time on each of the three days.

The daily dose depends on the patient's **body weight**. Your doctor should have prescribed a dose that is appropriate for your weight or your child's weight as follows:

Body weight (kg)	Daily dose (mg)	Total number of tablets for treatment
5 to less than 7	Half 160 mg/20 mg tablet a day	1.5 tablet
7 to less than 13	One 160 mg/20 mg tablet a day	3 tablets
13 to less than 24	One 320 mg/40 mg tablet a day	3 tablets
24 to less than 36	Two 320 mg/40 mg tablets a day	6 tablets
36 to less than 75	Three 320 mg/40 mg tablets a day	9 tablets
75 to 100	Four 320 mg/40 mg tablets a day	12 tablets

If you weigh more than 100 kg then follow the dose that your doctor has prescribed.

Vomiting when taking this medicine
If this happens within:

- 30 minutes of taking Eurartesim, the whole dose must be taken again.
- 31-60 minutes, half the dose must be taken again.

If you or your child vomit also the second dose, do not take or give your child another dose. Contact your doctor urgently to obtain an alternative treatment for malaria.

Taking this medicine, if the malaria infection returns

- If you or your child gets another attack of malaria you may take a second course of Eurartesim within one year if your doctor thinks this is a suitable treatment. You or your child must not take more than two courses within one year. If this happens, talk to your doctor. You or your child should not take a second course of Eurartesim within 2 months of the first course.
- If you or your child is infected more than twice in a year, your doctor will prescribe an alternative treatment.

If you or your child takes more Eurartesim tablets than you should
If you or your child takes more than the recommended dose, tell your doctor. Your doctor may suggest special monitoring for you or your child because doses higher than those recommended may have an unwanted, severe effect on your heart (see also section 4).

If you or your child forgets to take Eurartesim
If you or your child forgets to take the second dose of Eurartesim at the right time, take it as soon as you remember. Then take the third (last) dose approximately 24 hours after the second dose.
If you or your child forgets to take the third (last) dose at the right time, take it as soon as you remember.
Never take more than one dose on the same day to make up for a missed dose.
Check with your doctor or pharmacist if you are not sure.

If you or your child stops taking Eurartesim
For the medicine to work effectively, you or your child should take the tablets as instructed and should complete the 3 days course of treatment. If you or your child is not able to do this, talk to your doctor or pharmacist.

If you have any further questions on the use of this medicine, ask your doctor or pharmacist.

4. POSSIBLE SIDE EFFECTS

Like all medicines, Eurartesim can cause side effects, although not everybody gets them. Most of the side effects are not severe and normally disappear within a few days or weeks after treatment.

Do not use Eurartesim if you notice the blister package is open.

Medicines should not be disposed of via wastewater or household waste. Ask your pharmacist how to dispose of medicines no longer required. These measures will help to protect the environment.

6. FURTHER INFORMATION

What Eurartesim contains
Each film-coated tablet contains 320 mg piperaquine tetraphosphate (as the tetrahydrate) and 40 mg dihydroartemisinin.

The other ingredients are:
Tablet core: pre-gelatinised starch, dextrin, hypromellose (E464), croscarmellose sodium, magnesium stearate (E572).
Film coating: hypromellose, titanium dioxide (E171), macrogol 400.

What Eurartesim looks like and contents of the pack
Eurartesim are white film-coated tablets, embossed and with a break line along the middle.

The 320 mg/40 mg tablets have two 'σ' letters on one side and come in blister strips containing 3, 6, 9 or 12 tablets.

Marketing Authorisation Holder

Sigma-Tau Industrie Farmaceutiche Riunite s.p.a.
Viale Shakespeare 47
00144 Rome
Italy

Tel: +39 06 5926443
Fax: +39 06 5926600
Email: sigmatauinfo@sigma-tau.it

Manufacturer

Sigma-Tau Industrie Farmaceutiche Riunite s.p.a.
Via Pontina km. 30,400
00040 Pomezia (Rome)
Italy

Tel: +39 06 91391
Fax: +39 06 91166976
Email: sigmatauinfo@sigma-tau.it

For any information about this medicine, please contact the local representative of the Marketing Authorisation Holder:

België/Belgique/Belgien
MPCA bvba
Tél/Tel: + 32 9 344 53 38

Luxembourg/Luxemburg
MPCA bvba
Belgique/Belgien
Tél/Tel: + 32 9 344 53 38

Nederland
Sigma-Tau BV
Tel: +31 30 6702020
info@sigma-tau.nl

Deutschland
Sigma-Tau Arzneimittel GmbH
Tel.: +49 211 687717 0
info@sigma-tau.de

España
Sigma-Tau España, S.A.
Tel: +34 91 888 36 00
sigmatau@sigma-tau.es

Portugal
Defiante Farmacêutica, S.A.
Tel: +351 291 214 090
info@defiante.com

France
Sigma-Tau France
Tél: + 33 1 45 21 02.69

United Kingdom
Sigma-Tau Pharma Limited UK
Tel: +44 (0) 8000431268
medical.information@sigma-tau.co.uk

Ireland
Sigma-Tau Pharma Limited UK
United Kingdom
Tel: +44 (0) 8000431268
medical.information@sigma-tau.co.uk

България, Magyarország, Ceská republika, Malta, Danmark, Norge, Eesti, Österreich, Ελλάδα, Polska, România, Slovenija, Ísland, Slovenská republika, Italia, Suomi/Finland, Κύπρος, Sverige,Latvija, Lietuva

Sigma-Tau Industrie Farmaceutiche Riunite s.p.a.
Италия, Olaszország, Itàlie, Italja, Italien, Italia, Itaalia, Ιταλία, Włochy, Italija, Ítalía, taliansko, Itālija
Тел/Tel/Tlf/Τηλ/Sími/Puh: +39 06 9139.3796
Daniela.Campanelli@sigma-tau.it

This leaflet was last revised in

Detailed information on this medicine is available on the European Medicines Agency web site: http://www.ema.europa.eu

16.9.2 Azithromycin (as Zithromax®, Pfizer)

The summary of product characterisitics is provided as the generic drug below. Further detailed product charactersitics of the intended brand of choice may be found at www.accessdata.fda.gov/

Azithromycin

Antibiotic Class:
Macrolide

Antimicrobial Spectrum:
Staphylococcus aureus, Bacillus cereus, Bordetella pertussis, Chlamydia trachomatis, Corynebacterium diphtheriae, Gardnerella vaginalis, H. influenzae, Legionella pneumophila, Moraxella catarrhalis, Mycobacterium spp., Mycoplasma pneumoniae, Pasteurella multocida, S. pneumoniae, S. pyogenes.

Mechanism of Action:
Macrolides inhibit protein synthesis. They impair the elongation cycle of the peptidyl chain by specifically binding to the 50 S subunit of the ribosome.

Pharmacodynamics:
Macrolides produce time-dependent killing

Pharmacokinetics:
500mg dose: Cmax: 0.4mg/L; Half-life: 35-40 hours[illegible]lume of distribution: 23-31 L/kg; Table 3

Adverse Effects:
Gastrointestinal: abdominal cramps, nausea, diarrhea, anorexia, pancreatitis
Genitourinary: vulvovaginal candidiasis, renal failure
Cardiovascular System: prolongation of QT interval
Hepatic: hepatotoxicity, jaundice
Hematologic: eosinophilia, thrombocytosis, lymphopenia
Central Nervous System: headache, fatigue
Endocrine/Metabolic: hyperglycemia
Dermatologic: itching, nail discoloration

Dosage:
Oral: 250mg, 600mg tablet
1gram packet
100mg/5ml, 200mg/5ml powder for reconstitution to suspension
IV: 500mg vial

Dosing in adults:
Acute bacterial exacerbations of COPD: 500mg PO q24h x 3 days, or 500mg on day 1, 250 mg q24h on days 2-5
Acute bacterial sinusitis 500mg PO q24h x 3 days
Cervicitis due to *Chlamydia trachomatis:* 1 gram PO x 1 dose
Cervicitis due to *Neisseria gonorrhoeae:* 2 gram PO x 1 dose
Chancroid (genital ulcer disease due to *Haemophilus ducreyi*): 1 gram PO x 1
Mycobacterium avium complex, prophylaxis: 1200mg PO q weekly
Mycobacterium avium complex, treatment: 500 mg PO q24h (in combination with ethambutol)

Pharyngitis/tonsillitis: 500mg PO day 1, then 250mg q24h on days 2-5
Community acquired pneumonia (mild severity): 500mg PO day 1, then 250mg q24h on days 2-5
Skin and skin structure infections (uncomplicated): 500mg PO day 1, 250mg q24h on days 2-5
Urethritis due to *Chlamydia trachomatis*: 1 gram PO x 1
Urethritis due to *Neisseria gonorrhoeae*: 2 gram PO x 1
Pelvic inflammatory disease: 500mg IV q24h for at least 2 days, then 250 mg PO q24h x 7 days total
Community acquired pneumonia: 500mg IV q24h x at least 2 days, followed by 500mg PO q24h x 7-10 days total.

Dosing in pediatrics:
Acute bacterial sinusitis (≥ 6 months): 10 mg/kg PO q24h x 3 days
Mycobacterium avium complex disease, primary prevention: 20 mg/kg PO q weekly (maximum per dose 1200 mg)
Mycobacterium avium complex disease, secondary prevention: 5 mg/kg q24h (maximum dose 250mg) PO combined with ethambutol
Mycobacterium avium complex disease, treatment: 5 mg/kg up to 20 mg/kg PO x 1 month or longer
Otitis media, acute (≥ 6 months): 30mg/kg PO x 1, or 10mg/kg PO q24h x 3 days, or 10 mg/kg PO on day 1 followed by 5 mg/kg PO q24h on days 2-5
Pharyngitis, tonsillitis (≥2 years old): 12 mg/kg PO q24h x 5 days
Pharyngitis/tonsillitis (second-line therapy): (≥16 years) 500 mg PO day 1, then 250 mg q24h on days 2-5
Pneumonia, community-acquired (≥6 months): 10 mg/kg PO day 1, then 5 mg/kg q24h on days 2-5
Pneumonia, community-acquired (mild severity): (≥16 years) 500mg PO day 1, then 250 mg q24h on days 2-5
Skin and skin structure infections (uncomplicated): (≥16 years) 500mg PO on day 1, then 250mg q24h on days 2-5

Disease state based dosing:
Hepatic failures: No adjustment necessary
Renal failures: No adjustment necessary, however should be used with caution in patients with CrCl < 10ml/min. No supplemental doses needed after dialysis.

Contraindications/Warnings/Precautions:
Contraindicated: Coadministration with astemizole, cisapride, ergotamine, terfenadine
Precautions: May prolong the QTc interval

Drug Interactions:
Due to its hepatic metabolism, caution should be exercised when administering this agent with other drugs metabolized in the liver. The following drug interactions are clinically relevant but do not represent the comprehensive list of documented or potential drug-drug interactions.

Amiodarone: Incereased risk of cardiotoxicity (QTc prolongation)
Cyclosporine: Concomitant administration may increase cyclosporine levels. Close monitoring of cyclosporine levels is recommended
Nelfinavir: Coadministration may lead to increased azithromycin levels
Phenytoin: Concomitant administration may increase phenytoin levels. Close monitoring of phenytoin levels is recommended

Pregnancy:
Category B: No evidence of risk in humans but studies inadequate.

Monitoring Requirements:
Therapeutic: Periodic WBC, chest X-ray if pneumonia, cultures, temperature

Brand names/Manufacturer:
ZITHROMAX (Pfizer – MALAYSIA, THAILAND, SINGAPORE, FINLAND, NEW ZEALAND, ISRAEL, GREECE, CHILE, UNITED KINGDOM, USA, FRANCE, NETHERLANDS, AUSTRIA, AUSTRALIA, GERMANY, SWITZERLAND, SOUTH AFRICA, IRELAND, CANADA, PORTUGAL, HONG KONG)
For a complete manufacturer's list, please click

16.9.3 Sulphadoxine-pyrimethamine

The summary of product characterisitics is provided below. Further detailed product charactersitics of the intended brand of choice may be found at www.accessdata.fda.gov/

HLR 052803

FANSIDAR®

brand of

sulfadoxine and pyrimethamine

TABLETS

R_x only

WARNING: FATALITIES ASSOCIATED WITH THE ADMINISTRATION OF FANSIDAR HAVE OCCURRED DUE TO SEVERE REACTIONS, INCLUDING STEVENS-JOHNSON SYNDROME AND TOXIC EPIDERMAL NECROLYSIS. FANSIDAR PROPHYLAXIS MUST BE DISCONTINUED AT THE FIRST APPEARANCE OF SKIN RASH, IF A SIGNIFICANT REDUCTION IN THE COUNT OF ANY FORMED BLOOD ELEMENTS IS NOTED, OR UPON THE OCCURRENCE OF ACTIVE BACTERIAL OR FUNGAL INFECTIONS.

DESCRIPTION

Fansidar is an antimalarial agent, each tablet containing 500 mg N^1-(5,6-dimethoxy-4-pyrimidinyl) sulfanilamide (sulfadoxine) and 25 mg 2,4-diamino-5-(p-chlorophenyl)-6-ethylpyrimidine (pyrimethamine). Each tablet also contains cornstarch, gelatin, lactose, magnesium stearate and talc.

Sulfadoxine

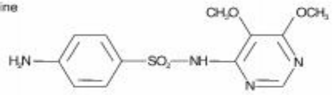

Pyrimethamine

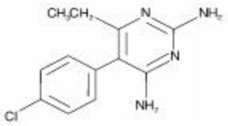

HLR 052803

FANSIDAR® (sulfadoxine and pyrimethamine)

CLINICAL PHARMACOLOGY

Microbiology

Mechanism of Action: Sulfadoxine and pyrimethamine, the constituents of Fansidar, are folic acid antagonists. Sulfadoxine inhibits the activity of dihydropteroate synthase whereas pyrimethamine inhibits dihydrofolate reductase.

Activity *in vitro*: Sulfadoxine and pyrimethamine are active against the asexual erythrocytic stages of *Plasmodium falciparum*. Fansidar may also be effective against strains of *P. falciparum* resistant to chloroquine.

Drug Resistance: Strains of *P. falciparum* with decreased susceptibility to sulfadoxine and /or pyrimethamine can be selected *in vitro* or *in vivo*. *P. falciparum* malaria that is clinically resistant to Fansidar occurs frequently in parts of Southeast Asia and South America, and is also prevalent in East and Central Africa. Therefore, Fansidar should be used with caution in these areas. Likewise, Fansidar may not be effective for treatment of recrudescent malaria that develops after prior therapy (or prophylaxis) with Fansidar.

PHARMACOKINETICS

Absorption

After administration of 1 tablet, peak plasma levels for pyrimethamine (approximately 0.2 mg/L) and for sulfadoxine (approximately 60 mg/L) are reached after about 4 hours.

Distribution

The volume of distribution for sulfadoxine and pyrimethamine is 0.14 L/kg and 2.3 L/kg, respectively.

Patients taking 1 tablet a week (recommended adult dose for malaria prophylaxis) can be expected to have mean steady state plasma concentrations of about 0.15 mg/L for pyrimethamine after about four weeks and about 98 mg/L for sulfadoxine after about seven weeks. Plasma protein binding is about 90% for both pyrimethamine and sulfadoxine. Both pyrimethamine and sulfadoxine cross the placental barrier and pass into breast milk.

Metabolism

About 5% of sulfadoxine appears in the plasma as acetylated metabolite, about 2 to 3% as the glucuronide. Pyrimethamine is transformed to several unidentified metabolites.

Elimination

A relatively long elimination half-life is characteristic of both components. The mean values are about 100 hours for pyrimethamine and about 200 hours for sulfadoxine. Both pyrimethamine and sulfadoxine are eliminated mainly via the kidneys.

HLR 052803

FANSIDAR® (sulfadoxine and pyrimethamine)

Characteristics in Patients

In malaria patients, single pharmacokinetic parameters may differ from those in healthy subjects, depending on the population concerned. In patients with renal insufficiency, delayed elimination of the components of Fansidar must be anticipated.

INDICATIONS AND USAGE

Treatment of Acute Malaria

Fansidar is indicated for the treatm[illegible]f acute, uncomplicated *P. falciparum* malaria for those patients in whom chloroquine resistance is suspected. However, strains of *P. falciparum* (see **CLINICAL PHARMACOLOGY: Microbiology**) may be encountered which have developed resistance to Fansidar, in which case alternative treatment should be administered.

Prevention of Malaria

Malaria prophylaxis with Fansidar is not routinely recommended and should only be considered for travelers to areas where chloroquine-resistant *P. falciparum* malaria is endemic and sensitive to Fansidar, and when alternative drugs are not available or are contraindicated (see **CONTRAINDICATIONS**). However, strains of *P. falciparum* may be encountered which have developed resistance to Fansidar.

CONTRAINDICATIONS

- Repeated prophylactic use of Fansidar is contraindicated in patients with renal or hepatic failure or with blood dyscrasias;
- Hypersensitivity to pyrimethamine, sulfonamides, or any other ingredient of Fansidar;
- Patients with documented megaloblastic anemia due to folate deficiency;
- Infants less than 2 months of age;
- Prophylactic use of Fansidar in pregnancy at term and during the nursing period.

HLR 052803

FANSIDAR® (sulfadoxine and pyrimethamine)

WARNINGS

FATALITIES ASSOCIATED WITH THE ADMINISTRATION OF FANSIDAR HAVE OCCURRED DUE TO SEVERE REACTIONS, INCLUDING STEVENS-JOHNSON SYNDROME AND TOXIC EPIDERMAL NECROLYSIS. FANSIDAR PROPHYLAXIS MUST BE DISCONTINUED AT THE FIRST APPEARANCE OF SKIN RASH, IF A SIGNIFICANT REDUCTION IN THE COUNT OF ANY FORMED BLOOD ELEMENTS IS NOTED, OR UPON THE OCCURRENCE OF ACTIVE BACTERIAL OR FUNGAL INFECTIONS.

Fatalities associated with the administration of sulfonamides, although rare, have occurred due to severe reactions, including fulminant hepatic necrosis, agranulocytosis, aplastic anemia and other blood dyscrasias. Fansidar prophylactic regimen has been reported to cause leukopenia during a treatment of 2 months or longer. This leukopenia is generally mild and reversible.

PRECAUTIONS

General

Oral Fansidar has not been evaluated for the treatment of cerebral malaria or other severe manifestations of complicated malaria, including hyperparasitemia, pulmonary edema or renal failure. Patients with severe malaria are not candidates for oral therapy. In the event of recrudescent *P. falciparum* infections after treatment with Fansidar or failure of chemoprophylaxis with Fansidar, patients should be treated with a different blood schizonticide.

Fansidar should be given with caution to patients with impaired renal or hepatic function, to those with possible folate deficiency and to those with severe allergy or bronchial asthma. As with some sulfonamide drugs, in glucose-6-phosphate dehydrogenase-deficient individuals, hemolysis may occur. Urinalysis with microscopic examination and renal function tests should be performed during therapy of those patients who have impaired renal function. Excessive sun exposure should be avoided.

Information for the Patient

Patients should be warned that at the first appearance of a skin rash, they should stop use of Fansidar and seek medical attention immediately. Adequate fluid intake must be maintained in order to prevent crystalluria and stone formation.

Patients should also be warned that the appearance of sore throat, fever, arthralgia, cough, shortness of breath, pallor, purpura, jaundice or glossitis may be early indications of serious disorders which require prophylactic treatment to be stopped and medical treatment to be sought.

Females should be cautioned against becoming pregnant and should not breastfeed their infants during Fansidar therapy or prophylactic treatment.

Patients should be warned to keep Fansidar out of reach of children.

Patients also should be advised:

HLR 052803

FANSIDAR® (sulfadoxine and pyrimethamine)

- that malaria can be a life-threatening infection;
- that Fansidar is being prescribed to help prevent or treat this serious infection;
- that no chemoprophylactic regimen is 100% effective, and protective clothing, insect repellents, and bednets are important components of malaria prophylaxis;
- to seek medical attention for any febrile illness that occurs after return from a malarious area and inform their physician that they may have been exposed to malaria;
- that in a small percentage of cases, patients are unable to take this medication because of side effects, and it may be necessary to change medications;
- that when used as prophylaxis, the first dose of Fansidar should be taken 1 or 2 days prior to arrival in an endemic area;
- that if the patient experiences any symptom that may affect the patient's ability to take this drug as prescribed, the physician should be contacted and alternative antimalarial medication should be considered.

Laboratory Tests

Regularly scheduled complete blood counts, liver enzyme tests and analysis of urine for crystalluria should be performed whenever Fansidar is administered for more than three months.

Drug Interactions

There have been reports which may indicate an increase in incidence and severity of adverse reactions when chloroquine is used with Fansidar as compared to the use of Fansidar alone. Fansidar is compatible with quinine and with antibiotics. However, antifolic drugs such as sulfonamides, trimethoprim, or trimethoprim-sulfamethoxazole combinations should not be used while the patient is receiving Fansidar for antimalarial prophylaxis. Fansidar has not been reported to interfere with antidiabetic agents.

If signs of folic acid deficiency develop, Fansidar should be discontinued. When recovery of depressed platelets or white blood cell counts in patients with drug-induced folic acid deficiency is too slow, folinic acid (leucovorin) may be administered in doses of 5-15 mg intramuscularly daily for 3 days or longer.

Carcinogenesis, Mutagenesis, Impairment of Fertility

Pyrimethamine was not found carcinogenic in female mice or in male and female rats. The carcinogenic potential of pyrimethamine in male mice could not be assessed from the study because of markedly reduced life-span. Pyrimethamine was found to be mutagenic in laboratory animals and also in human bone marrow following 3 or 4 consecutive daily doses totaling 200 mg to 300 mg. Pyrimethamine was not found mutagenic in the Ames test. Testicular changes have been observed in rats treated with 105 mg/kg/day of Fansidar and with 15 mg/kg/day of pyrimethamine alone. Fertility of male rats and the ability of male or female rats to mate were not adversely affected at dosages of up to 210

mg/kg/day of Fansidar. The pregnancy rate of female rats was not affected following their treatment with 10.5 mg/kg/day, but was significantly reduced at dosages of 31.5 mg/kg/day or higher, a dosage approximately 30 times the weekly human prophylactic dose or higher.

Pregnancy

Teratogenic Effects: Pregnancy Category C. Fansidar has been shown to be teratogenic in rats when given in weekly doses approximately 12 times the weekly human prophylactic dose. Teratology studies with pyrimethamine plus sulfadoxine (1:20) in rats showed the minimum oral teratogenic dose to be approximately 0.9 mg/kg pyrimethamine plus 18 mg/kg sulfadoxine. In rabbits, no teratogenic effects were noted at oral doses as high as 20 mg/kg pyrimethamine plus 400 mg/kg sulfadoxine.

There are no adequate and well-controlled studies in pregnant women. However, due to the teratogenic effect shown in animals and because pyrimethamine plus sulfadoxine may interfere with folic acid metabolism, Fansidar therapy should be used during pregnancy only if the potential benefit justifies the potential risk to the fetus. Women of childbearing potential who are traveling to areas where malaria is endemic should be warned against becoming pregnant, and should be advised to practice contraception during prophylaxis with Fansidar and for three months after the last dose.

Nonteratogenic Effects

See **CONTRAINDICATIONS**.

Nursing Mothers

See **CONTRAINDICATIONS**.

Pediatric Use

Fansidar should not be given to infants less than 2 months of age because of inadequate development of the glucuronide-forming enzyme system.

Geriatric Use

Clinical studies of Fansidar did not include sufficient numbers of subjects aged 65 and over to determine whether they respond differently from younger subjects. Other reported clinical experience has not identified differences in responses between the elderly and younger patients. In general, dose selection for an elderly patient should be cautious, usually starting at the low end of the dosing range, reflecting the greater frequency of decreased hepatic, renal or cardiac function, and of concomitant disease or other drug therapy. This drug is known to be substantially excreted by the kidney, and the risk of toxic reactions to this drug may be greater in patients with impaired renal function. Because elderly patients are more likely to have decreased renal function, care should be taken in dose selection, and it may be useful to monitor renal function.

HLR 052803

FANSIDAR® (sulfadoxine and pyrimethamine)

ADVERSE REACTIONS

For completeness, all major reactions to sulfonamides and to pyrimethamine are included below, even though they may not have been reported with Fansidar (see **WARNINGS** and **PRECAUTIONS: Information for the Patient**).

Hematological Changes

Agranulocytosis, aplastic anemia, megaloblastic anemia, thrombocytopenia, leukopenia, hemolytic anemia, purpura, hypoprothrombinemia, methemoglobinemia, and eosinophilia.

Skin and Miscellaneous Sites Allergic Reactions

Erythema multiforme, Stevens-Johnson syndrome, generalized skin eruptions, toxic epidermal necrolysis, urticaria, serum sickness, pruritus, exfoliative dermatitis, anaphylactoid reactions, periorbital edema, conjunctival and scleral injection, photosensitization, arthralgia, allergic myocarditis, slight hair loss, Lyell's syndrome, and allergic pericarditis.

Gastrointestinal Reactions

Glossitis, stomatitis, nausea, emesis, abdominal pains, hepatitis, hepatocellular necrosis, diarrhea, pancreatitis, feeling of fullness, and transient rise of liver enzymes.

Central Nervous System Reactions

Headache, peripheral neuritis, mental depression, convulsions, ataxia, hallucinations, tinnitus, vertigo, insomnia, apathy, fatigue, muscle weakness, nervousness, and polyneuritis.

Respiratory Reactions

Pulmonary infiltrates resembling eosinophilic or allergic alveolitis.

Genitourinary

Renal failure, interstitial nephritis, BUN and serum creatinine elevation, toxic nephrosis with oliguria and anuria, and crystalluria.

Miscellaneous Reactions

Drug fever, chills, periarteritis nodosa and LE phenomenon have occurred.

The sulfonamides bear certain chemical similarities to some goitrogens, diuretics (acetazolamide and the thiazides), and oral hypoglycemic agents. Diuresis and hypoglycemia have occurred rarely in patients receiving sulfonamides. Cross-sensitivity may exist with these agents. Rats appear to be especially susceptible to the goitrogenic effects of sulfonamides, and long-term administration has produced thyroid malignancies in the species.

HLR 052803

FANSIDAR® (sulfadoxine and pyrimethamine)

OVERDOSAGE

Acute intoxication may be manifested by headache, nausea, anorexia, vomiting and central nervous system stimulation (including convulsions), followed by megaloblastic anemia, leukopenia, [illegible]mbocytopenia, glossitis and crystalluria. In acute intoxication, emesis and gastric lavage followed by purges may be of benefit. The patient should be adequately hydrated to prevent renal damage. The renal, hepatic, and hematopoietic systems should be monitored for at least 1 month after an overdosage. If the patient is having convulsions, the use of parenteral diazepam or a barbiturate is indicated. For depressed platelet or white blood cell counts, folinic acid (leucovorin) should be administered in a dosage of 5 mg to 15 mg intramuscularly daily for 3 days or longer.

DOSAGE AND ADMINISTRATION (See INDICATIONS AND USAGE)

The dosage should be swallowed whole, and not chewed, with plenty of fluids after a meal.

Treatment of Acute Malaria

Adults	2 to 3 tablets taken as a single dose.
Pediatric patients (>2 months to 18 years)	The dosage for treatment of malaria in children is based upon body weight:

Weight (kg)	Number of Tablets Taken as a Single Dose
>45	3
31 to 45	2
21 to 30	1 ½
11 to 20	1
5 to 10	½

Prevention of Malaria

The malaria risk must be carefully weighed against the risk of serious adverse drug reactions (see **INDICATIONS AND USAGE**). If Fansidar is prescribed for prophylaxis, it is important that the physician inquires about sulfonamide intolerance and points out the risk and the need for immediate drug withdrawal if skin reactions do occur.

The first dose of Fansidar should be taken 1 or 2 days before arrival in an endemic area; administration should be continued during the stay and for 4 to 6 weeks after return.

HLR 052803

FANSIDAR® (sulfadoxine and pyrimethamine)

	Once Weekly	Once Every 2 Weeks
Adults	1 tablet	2 tablets

Pediatric patients (>2 months to 18 years) — The dosage for prevention of malaria in children is based upon body weight:

Weight (kg)	Number of Tablets Taken Once Weekly
>45	1 ½
31 to 45	1
21 to 30	¾
11 to 20	½
5 to 10	¼

Prophylaxis with Fansidar should not be continued for more than two years, since no experience of more prolonged administration is available to date.

HOW SUPPLIED

Scored tablets, containing 500 mg sulfadoxine and 25 mg pyrimethamine — unit dose packages of 25 (NDC-0004-0161-03). Imprint on tablets: FANSIDAR ((ROCHE LOGO)) ROCHE.

Manufactured by:
F. Hoffmann-La Roche Ltd.
Basel, Switzerland

Distributed by:

Roche Pharmaceuticals

Roche Laboratories Inc.
340 Kingsland Street
Nutley, New Jersey 07110-1199

XXXXXXXX

Revised: XXXX XXXX

Copyright © 1996-200X by Roche Laboratories Inc. All rights reserved.

9

16.10 Appendix IX. Questionnaires

The questionnaires are provided as a separate document entitled Appendix IX. Questionnaires
